# Supplementary material for: Gypsum, crop rotation, and cover crop impacts on soil organic carbon and biological dynamics in rainfed transitional no-till corn-soybean systems
Source: PLoS One. 2022 Sep 27;17(9):e0275198. doi: 10.1371/journal.pone.0275198 (PMC9514652; doi:10.1371/journal.pone.0275198)
Supplement: S1 File — (PDF) [file pone.0275198.s001.pdf]

Soil microbial biomass and organic carbon pools and lability

Obs	Site	Gypsum	CR	CC	Depth	Rep	SMB (mg/kg)	qR (%)
1	Indiana	1000	SS	No	0	1	478.4	3.70
2	Indiana	1000	SS	No	15	1	153.6	2.30
3	Indiana	1000	SS	Yes	0	1	353.6	2.60
4	Indiana	1000	SS	Yes	15	1	172.8	2.40
5	Indiana	0	SS	Yes	0	1	156.6	1.50
6	Indiana	0	SS	Yes	15	1	86.2	0.70
7	Indiana	0	SS	No	0	1	322.5	3.10
8	Indiana	0	SS	No	15	1	66.9	1.40
9	Indiana	0	SS	No	0	2	392.6	3.00
10	Indiana	0	SS	No	15	2	167.9	2.50
11	Indiana	0	SS	Yes	0	2	486.9	3.30
12	Indiana	0	SS	Yes	15	2	229.3	3.00
13	Indiana	1000	SS	Yes	0	2	413.3	3.00
14	Indiana	1000	SS	Yes	15	2	162.2	2.50
15	Indiana	2000	SS	No	0	1	518.4	4.30
16	Indiana	2000	SS	No	15	1	413.6	6.70
17	Indiana	2000	SS	No	0	2	279.2	2.30
18	Indiana	2000	SS	No	15	2	138.5	2.30
19	Indiana	2000	SS	Yes	0	1	466.4	3.60
20	Indiana	2000	SS	Yes	15	1	151.5	2.30
21	Indiana	2000	SS	Yes	0	2	396.5	3.30
22	Indiana	2000	SS	Yes	15	2	148.7	2.50
23	Indiana	1000	SS	No	0	2	164.5	1.50
24	Indiana	1000	SS	No	15	2	64.8	0.90
25	Indiana	1000	SS	Yes	0	3	302.8	2.30
26	Indiana	1000	SS	Yes	15	3	230.8	1.70
27	Indiana	2000	SS	No	0	3	288.1	2.70
28	Indiana	2000	SS	No	15	3	193.6	1.90
29	Indiana	0	SS	No	0	3	224.7	1.80
30	Indiana	0	SS	No	15	3	172.0	1.70
31	Indiana	1000	SS	Yes	0	4	315.5	2.30
32	Indiana	1000	SS	Yes	15	4	215.6	2.00
33	Indiana	0	SS	Yes	0	3	299.7	2.40
34	Indiana	0	SS	Yes	15	3	258.5	3.60
35	Indiana	0	SS	No	0	4	300.6	2.10
36	Indiana	0	SS	No	15	4	172.0	1.50
37	Indiana	2000	SS	No	0	4	293.8	1.50
38	Indiana	2000	SS	No	15	4	207.0	1.40
39	Indiana	0	SS	Yes	0	4	180.4	1.00
40	Indiana	0	SS	Yes	15	4	59.0	0.30
41	Indiana	2000	SS	Yes	0	3	615.8	4.50
42	Indiana	2000	SS	Yes	15	3	349.1	4.00

43	Indiana	1000	SS	No	0	3	115.4	0.90
44	Indiana	1000	SS	No	15	3	64.4	0.60
45	Indiana	1000	SS	No	0	3	365.2	2.00
46	Indiana	1000	SS	No	15	3	238.4	1.50
47	Indiana	2000	SS	Yes	0	4	322.5	2.00
48	Indiana	2000	SS	Yes	15	4	242.2	1.60
49	Indiana	1000	SC	No	0	1	174.1	1.60
50	Indiana	1000	SC	No	15	1	104.7	2.00
51	Indiana	1000	SC	Yes	0	1	151.3	1.40
52	Indiana	1000	SC	Yes	15	1	86.5	1.30
53	Indiana	0	SC	Yes	0	1	156.2	1.20
54	Indiana	0	SC	Yes	15	1	106.4	1.20
55	Indiana	0	SC	No	0	1	116.8	1.10
56	Indiana	0	SC	No	15	1	71.5	0.80
57	Indiana	0	SC	No	0	2	96.5	0.80
58	Indiana	0	SC	No	15	2	37.2	0.40
59	Indiana	0	SC	Yes	0	2	101.3	0.80
60	Indiana	0	SC	Yes	15	2	94.8	1.30
61	Indiana	1000	SC	Yes	0	2	215.5	1.70
62	Indiana	1000	SC	Yes	15	2	190.0	1.80
63	Indiana	2000	SC	No	0	1	390.6	3.00
64	Indiana	2000	SC	No	15	1	236.0	2.70
65	Indiana	2000	SC	No	0	2	193.1	1.70
66	Indiana	2000	SC	No	15	2	92.2	1.30
67	Indiana	2000	SC	Yes	0	1	387.9	2.90
68	Indiana	2000	SC	Yes	15	1	367.7	3.80
69	Indiana	2000	SC	Yes	0	2	377.9	2.10
70	Indiana	2000	SC	Yes	15	2	236.6	1.70
71	Indiana	1000	SC	No	0	2	186.6	1.10
72	Indiana	1000	SC	No	15	2	154.8	1.10
73	Indiana	1000	SC	Yes	0	3	222.6	1.50
74	Indiana	1000	SC	Yes	15	3	160.9	1.40
75	Indiana	2000	SC	No	0	3	297.2	1.80
76	Indiana	2000	SC	No	15	3	224.2	1.60
77	Indiana	0	SC	No	0	3	114.2	0.70
78	Indiana	0	SC	No	15	3	92.5	0.60
79	Indiana	1000	SC	Yes	0	4	295.0	1.70
80	Indiana	1000	SC	Yes	15	4	174.0	1.20
81	Indiana	0	SC	Yes	0	3	152.2	1.00
82	Indiana	0	SC	Yes	15	3	108.2	0.90
83	Indiana	0	SC	No	0	4	153.5	0.90
84	Indiana	0	SC	No	15	4	102.2	0.70
85	Indiana	2000	SC	No	0	4	296.9	2.20
86	Indiana	2000	SC	No	15	4	181.4	1.50
87	Indiana	0	SC	Yes	0	4	128.1	1.50
88	Indiana	0	SC	Yes	15	4	58.9	1.10
89	Indiana	2000	SC	Yes	0	3	398.7	3.10

90	Indiana	2000	SC	Yes	15	3	241.5	2.50
91	Indiana	1000	SC	No	0	3	294.5	2.60
92	Indiana	1000	SC	No	15	3	247.9	5.70
93	Indiana	1000	SC	No	0	3	280.7	2.40
94	Indiana	1000	SC	No	15	3	223.9	3.70
95	Indiana	2000	SC	Yes	0	4	406.5	3.20
96	Indiana	2000	SC	Yes	15	4	353.1	4.20
97	Indiana	1000	CS	No	0	1	311.1	2.70
98	Indiana	1000	CS	No	15	1	144.6	1.50
99	Indiana	1000	CS	Yes	0	1	250.9	2.00
100	Indiana	1000	CS	Yes	15	1	209.9	2.70
101	Indiana	1000	CS	Yes	0	1	223.3	1.30
102	Indiana	1000	CS	Yes	15	1	172.8	1.80
103	Indiana	2000	CS	No	0	1	287.2	1.90
104	Indiana	2000	CS	No	15	1	248.3	1.90
105	Indiana	0	CS	Yes	0	2	151.1	0.80
106	Indiana	0	CS	Yes	15	2	105.8	0.80
107	Indiana	0	CS	No	0	2	125.7	0.70
108	Indiana	0	CS	No	15	2	102.3	0.80
109	Indiana	0	CS	No	0	2	163.3	0.80
110	Indiana	0	CS	No	15	2	84.2	0.60
111	Indiana	1000	CS	Yes	0	1	209.9	1.10
112	Indiana	1000	CS	Yes	15	1	192.7	1.40
113	Indiana	0	CS	No	0	2	125.2	1.00
114	Indiana	0	CS	No	15	2	100.5	1.50
115	Indiana	0	CS	Yes	0	1	90.9	0.60
116	Indiana	0	CS	Yes	15	1	42.8	0.60
117	Indiana	0	CS	Yes	0	2	106.0	0.60
118	Indiana	0	CS	Yes	15	2	48.2	0.30
119	Indiana	0	CS	No	0	2	139.7	0.80
120	Indiana	0	CS	No	15	2	59.2	0.40
121	Indiana	1000	CS	Yes	0	3	154.7	0.90
122	Indiana	1000	CS	Yes	15	3	126.7	1.00
123	Indiana	2000	CS	No	0	3	285.0	1.70
124	Indiana	2000	CS	No	15	3	193.6	1.50
125	Indiana	2000	CS	No	0	3	196.8	1.10
126	Indiana	2000	CS	No	15	3	145.4	0.90
127	Indiana	0	CS	Yes	0	4	129.1	0.60
128	Indiana	0	CS	Yes	15	4	40.5	0.30
129	Indiana	2000	CS	No	0	3	310.7	2.50
130	Indiana	2000	CS	No	15	3	247.0	4.10
131	Indiana	2000	CS	Yes	0	4	362.3	2.80
132	Indiana	2000	CS	Yes	15	4	229.8	2.70
133	Indiana	2000	CS	Yes	0	4	353.1	2.10
134	Indiana	2000	CS	Yes	15	4	270.5	2.10
135	Indiana	1000	CS	No	0	4	253.8	1.40
136	Indiana	1000	CS	No	15	4	151.0	1.00

137	Indiana	2000	CS	Yes	0	3	424.7	2.20
138	Indiana	2000	CS	Yes	15	3	309.6	2.50
139	Indiana	1000	CS	No	0	3	264.5	1.50
140	Indiana	1000	CS	No	15	3	148.4	1.40
141	Indiana	1000	CS	No	0	3	208.8	1.10
142	Indiana	1000	CS	No	15	3	128.9	0.90
143	Indiana	2000	CS	Yes	0	4	425.7	1.90
144	Indiana	2000	CS	Yes	15	4	215.6	1.50
145	Alabama	1000	SS	No	0	1	134.2	2.10
146	Alabama	1000	SS	No	15	1	77.0	3.00
147	Alabama	0	SS	No	0	1	182.9	2.20
148	Alabama	0	SS	No	15	1	175.3	5.70
149	Alabama	2000	SS	No	0	1	144.0	2.00
150	Alabama	2000	SS	No	15	1	60.5	2.00
151	Alabama	1000	SS	Yes	0	1	109.2	1.80
152	Alabama	1000	SS	Yes	15	1	66.1	1.70
153	Alabama	0	SS	Yes	0	1	93.0	1.10
154	Alabama	0	SS	Yes	15	1	67.7	1.30
155	Alabama	2000	SS	Yes	0	1	114.2	1.20
156	Alabama	2000	SS	Yes	15	1	61.5	0.90
157	Alabama	2000	SS	Yes	0	2	136.7	2.70
158	Alabama	2000	SS	Yes	15	2	106.3	3.70
159	Alabama	0	SS	Yes	0	2	167.1	2.00
160	Alabama	0	SS	Yes	15	2	105.4	6.50
161	Alabama	1000	SS	Yes	0	2	189.5	2.70
162	Alabama	1000	SS	Yes	15	2	164.2	10.30
163	Alabama	2000	SS	No	0	2	117.0	1.90
164	Alabama	2000	SS	No	15	2	124.2	3.90
165	Alabama	0	SS	No	0	2	132.8	2.40
166	Alabama	0	SS	No	15	2	96.7	2.40
167	Alabama	1000	SS	No	0	2	183.8	1.70
168	Alabama	1000	SS	No	15	2	87.5	1.50
169	Alabama	0	SS	Yes	0	3	167.5	2.30
170	Alabama	0	SS	Yes	15	3	141.8	5.10
171	Alabama	1000	SS	Yes	0	3	177.7	2.60
172	Alabama	1000	SS	Yes	15	3	80.7	7.20
173	Alabama	2000	SS	Yes	0	3	186.5	4.60
174	Alabama	2000	SS	Yes	15	3	140.9	7.80
175	Alabama	0	SS	No	0	3	191.4	3.00
176	Alabama	0	SS	No	15	3	121.2	5.90
177	Alabama	2000	SS	No	0	3	134.6	2.40
178	Alabama	2000	SS	No	15	3	92.6	3.30
179	Alabama	1000	SS	No	0	3	100.9	1.30
180	Alabama	1000	SS	No	15	3	91.6	4.50
181	Alabama	0	SS	No	0	4	106.2	2.10
182	Alabama	0	SS	No	15	4	98.3	4.60
183	Alabama	2000	SS	No	0	4	209.3	6.10

184	Alabama	2000	SS	No	15	4	139.8	4.40
185	Alabama	1000	SS	No	0	4	173.6	3.70
186	Alabama	1000	SS	No	15	4	111.5	3.60
187	Alabama	1000	SS	Yes	0	4	105.4	2.40
188	Alabama	1000	SS	Yes	15	4	101.3	8.00
189	Alabama	0	SS	Yes	0	4	149.6	2.90
190	Alabama	0	SS	Yes	15	4	95.6	4.30
191	Alabama	2000	SS	Yes	0	4	190.6	2.80
192	Alabama	2000	SS	Yes	15	4	112.5	3.60
193	Alabama	1000	SC	No	0	1	183.8	4.10
194	Alabama	1000	SC	No	15	1	137.9	3.50
195	Alabama	0	SC	No	0	1	119.9	1.80
196	Alabama	0	SC	No	15	1	70.0	1.40
197	Alabama	2000	SC	No	0	1	85.9	1.20
198	Alabama	2000	SC	No	15	1	81.0	1.40
199	Alabama	1000	SC	Yes	0	1	160.7	1.70
200	Alabama	1000	SC	Yes	15	1	76.1	1.00
201	Alabama	0	SC	Yes	0	1	124.1	1.60
202	Alabama	0	SC	Yes	15	1	104.0	3.70
203	Alabama	2000	SC	Yes	0	1	115.3	1.00
204	Alabama	2000	SC	Yes	15	1	98.7	3.00
205	Alabama	2000	SC	Yes	0	2	144.0	2.40
206	Alabama	2000	SC	Yes	15	2	60.9	2.60
207	Alabama	0	SC	Yes	0	2	127.9	2.30
208	Alabama	0	SC	Yes	15	2	111.9	4.00
209	Alabama	1000	SC	Yes	0	2	86.2	1.20
210	Alabama	1000	SC	Yes	15	2	79.8	1.90
211	Alabama	2000	SC	No	0	2	141.0	1.80
212	Alabama	2000	SC	No	15	2	74.9	2.40
213	Alabama	0	SC	No	0	2	171.5	1.70
214	Alabama	0	SC	No	15	2	57.0	1.00
215	Alabama	1000	SC	No	0	2	88.5	1.00
216	Alabama	1000	SC	No	15	2	74.4	0.90
217	Alabama	0	SC	Yes	0	3	108.0	1.20
218	Alabama	0	SC	Yes	15	3	54.0	1.10
219	Alabama	1000	SC	Yes	0	3	165.4	1.70
220	Alabama	1000	SC	Yes	15	3	50.2	1.10
221	Alabama	2000	SC	Yes	0	3	172.6	2.10
222	Alabama	2000	SC	Yes	15	3	72.6	0.90
223	Alabama	0	SC	No	0	3	136.6	1.60
224	Alabama	0	SC	No	15	3	105.9	2.30
225	Alabama	2000	SC	No	0	3	168.0	1.80
226	Alabama	2000	SC	No	15	3	109.0	2.20
227	Alabama	1000	SC	No	0	3	123.8	1.00
228	Alabama	1000	SC	No	15	3	113.7	1.80
229	Alabama	0	SC	No	0	4	87.2	1.10
230	Alabama	0	SC	No	15	4	37.0	0.70

231	Alabama	2000	SC	No	0	4	260.1	4.30
232	Alabama	2000	SC	No	15	4	36.8	0.90
233	Alabama	1000	SC	No	0	4	168.4	2.70
234	Alabama	1000	SC	No	15	4	55.5	1.50
235	Alabama	1000	SC	Yes	0	4	127.2	1.30
236	Alabama	1000	SC	Yes	15	4	58.6	1.30
237	Alabama	0	SC	Yes	0	4	153.6	1.70
238	Alabama	0	SC	Yes	15	4	65.0	1.20
239	Alabama	2000	SC	Yes	0	4	188.7	1.60
240	Alabama	2000	SC	Yes	15	4	113.1	1.20
241	Alabama	1000	CS	No	0	1	219.1	2.60
242	Alabama	1000	CS	No	15	1	101.2	2.20
243	Alabama	0	CS	No	0	1	109.7	1.60
244	Alabama	0	CS	No	15	1	28.0	0.50
245	Alabama	2000	CS	No	0	1	172.4	2.40
246	Alabama	2000	CS	No	15	1	165.9	3.10
247	Alabama	1000	CS	Yes	0	1	126.8	1.60
248	Alabama	1000	CS	Yes	15	1	176.2	7.00
249	Alabama	0	CS	Yes	0	1	92.7	1.10
250	Alabama	0	CS	Yes	15	1	48.1	1.00
251	Alabama	2000	CS	Yes	0	1	200.6	1.80
252	Alabama	2000	CS	Yes	15	1	136.9	2.80
253	Alabama	2000	CS	Yes	0	2	230.2	2.60
254	Alabama	2000	CS	Yes	15	2	146.8	2.20
255	Alabama	0	CS	Yes	0	2	97.2	0.90
256	Alabama	0	CS	Yes	15	2	91.6	1.20
257	Alabama	1000	CS	Yes	0	2	132.8	1.20
258	Alabama	1000	CS	Yes	15	2	89.2	1.20
259	Alabama	2000	CS	No	0	2	172.0	1.60
260	Alabama	2000	CS	No	15	2	122.1	4.50
261	Alabama	0	CS	No	0	2	78.3	0.80
262	Alabama	0	CS	No	15	2	73.5	1.30
263	Alabama	1000	CS	No	0	2	118.2	1.30
264	Alabama	1000	CS	No	15	2	124.6	3.90
265	Alabama	0	CS	Yes	0	3	51.2	0.50
266	Alabama	0	CS	Yes	15	3	47.2	0.70
267	Alabama	1000	CS	Yes	0	3	149.2	1.40
268	Alabama	1000	CS	Yes	15	3	53.6	0.70
269	Alabama	2000	CS	Yes	0	3	179.3	1.50
270	Alabama	2000	CS	Yes	15	3	124.5	1.90
271	Alabama	0	CS	No	0	3	111.6	1.00
272	Alabama	0	CS	No	15	3	21.4	0.40
273	Alabama	2000	CS	No	0	3	234.9	1.80
274	Alabama	2000	CS	No	15	3	100.5	2.10
275	Alabama	1000	CS	No	0	3	151.7	1.60
276	Alabama	1000	CS	No	15	3	50.0	1.90
277	Alabama	0	CS	No	0	4	128.8	1.40

278	Alabama	0	CS	No	15	4	44.4	0.80
279	Alabama	2000	CS	No	0	4	206.1	2.10
280	Alabama	2000	CS	No	15	4	106.1	1.10
281	Alabama	1000	CS	No	0	4	181.9	1.70
282	Alabama	1000	CS	No	15	4	173.0	1.80
283	Alabama	1000	CS	Yes	0	4	132.6	1.10
284	Alabama	1000	CS	Yes	15	4	34.6	1.00
285	Alabama	0	CS	Yes	0	4	114.4	1.10
286	Alabama	0	CS	Yes	15	4	99.3	1.90
287	Alabama	2000	CS	Yes	0	4	218.4	2.10
288	Alabama	2000	CS	Yes	15	4	93.0	2.10
289	Piketon	2000	SS	Yes	0	1	238.0	2.30
290	Piketon	2000	SS	Yes	15	1	110.1	1.50
291	Piketon	1000	SS	No	0	2	142.3	1.50
292	Piketon	1000	SS	No	15	2	94.8	2.40
293	Piketon	1000	SS	No	0	3	208.3	1.80
294	Piketon	1000	SS	No	15	3	135.6	4.00
295	Piketon	2000	SS	Yes	0	4	262.9	2.50
296	Piketon	2000	SS	Yes	15	4	110.2	3.20
297	Piketon	0	SS	Yes	0	4	212.6	1.70
298	Piketon	0	SS	Yes	15	4	78.4	1.30
299	Piketon	2000	SS	No	0	3	232.0	2.30
300	Piketon	2000	SS	No	15	3	83.5	1.20
301	Piketon	0	SS	No	0	2	91.1	0.80
302	Piketon	0	SS	No	15	2	102.8	2.00
303	Piketon	0	SS	Yes	0	1	117.5	1.00
304	Piketon	0	SS	Yes	15	1	112.0	2.20
305	Piketon	1000	SS	Yes	0	1	107.8	1.10
306	Piketon	1000	SS	Yes	15	1	121.0	4.70
307	Piketon	2000	SS	No	0	2	197.4	2.20
308	Piketon	2000	SS	No	15	2	89.3	2.10
309	Piketon	0	SS	No	0	3	422.5	4.70
310	Piketon	0	SS	No	15	3	222.1	9.70
311	Piketon	1000	SS	Yes	0	4	320.8	4.00
312	Piketon	1000	SS	Yes	15	4	164.4	5.40
313	Piketon	1000	SS	No	0	4	234.3	2.80
314	Piketon	1000	SS	No	15	4	225.2	7.70
315	Piketon	2000	SS	Yes	0	3	348.5	3.40
316	Piketon	2000	SS	Yes	15	3	144.1	3.60
317	Piketon	1000	SS	Yes	0	2	315.7	4.30
318	Piketon	1000	SS	Yes	15	2	143.3	5.90
319	Piketon	2000	SS	No	0	1	404.6	3.50
320	Piketon	2000	SS	No	15	1	189.2	6.60
321	Piketon	0	SS	No	0	1	353.3	4.40
322	Piketon	0	SS	No	15	1	202.3	7.50
323	Piketon	0	SS	Yes	0	2	258.4	3.40
324	Piketon	0	SS	Yes	15	2	99.5	2.70

325	Piketon	1000	SS	Yes	0	3	85.6	1.10
326	Piketon	1000	SS	Yes	15	3	98.5	4.40
327	Piketon	2000	SS	No	0	4	212.4	3.30
328	Piketon	2000	SS	No	15	4	134.5	4.30
329	Piketon	0	SS	No	0	4	250.2	2.90
330	Piketon	0	SS	No	15	4	73.6	3.40
331	Piketon	0	SS	Yes	0	3	157.8	2.20
332	Piketon	0	SS	Yes	15	3	89.6	4.40
333	Piketon	2000	SS	Yes	0	2	198.6	2.60
334	Piketon	2000	SS	Yes	15	2	54.5	3.50
335	Piketon	1000	SS	No	0	1	120.2	1.80
336	Piketon	1000	SS	No	15	1	113.9	7.10
337	Piketon	2000	SC	Yes	0	1	268.5	3.00
338	Piketon	2000	SC	Yes	15	1	77.0	2.30
339	Piketon	1000	SC	No	0	2	275.0	2.90
340	Piketon	1000	SC	No	15	2	112.4	2.90
341	Piketon	1000	SC	No	0	3	310.9	3.30
342	Piketon	1000	SC	No	15	3	151.8	2.50
343	Piketon	2000	SC	Yes	0	4	277.2	2.30
344	Piketon	2000	SC	Yes	15	4	115.1	3.10
345	Piketon	0	SC	Yes	0	4	487.3	4.90
346	Piketon	0	SC	Yes	15	4	126.6	3.10
347	Piketon	2000	SC	No	0	3	526.1	5.00
348	Piketon	2000	SC	No	15	3	153.7	3.50
349	Piketon	0	SC	No	0	2	507.5	6.20
350	Piketon	0	SC	No	15	2	165.8	3.00
351	Piketon	0	SC	Yes	0	1	114.0	1.30
352	Piketon	0	SC	Yes	15	1	57.6	1.10
353	Piketon	1000	SC	Yes	0	1	176.8	2.10
354	Piketon	1000	SC	Yes	15	1	185.8	3.80
355	Piketon	2000	SC	No	0	2	299.2	3.10
356	Piketon	2000	SC	No	15	2	161.4	3.70
357	Piketon	0	SC	No	0	3	536.9	5.90
358	Piketon	0	SC	No	15	3	116.9	3.20
359	Piketon	1000	SC	Yes	0	4	494.7	4.90
360	Piketon	1000	SC	Yes	15	4	188.0	12.40
361	Piketon	1000	SC	No	0	4	469.4	4.10
362	Piketon	1000	SC	No	15	4	131.5	3.20
363	Piketon	2000	SC	Yes	0	3	401.0	4.00
364	Piketon	2000	SC	Yes	15	3	82.0	1.60
365	Piketon	1000	SC	Yes	0	2	339.3	3.40
366	Piketon	1000	SC	Yes	15	2	135.2	2.90
367	Piketon	2000	SC	No	0	1	443.7	6.80
368	Piketon	2000	SC	No	15	1	129.0	2.70
369	Piketon	0	SC	No	0	1	137.3	1.90
370	Piketon	0	SC	No	15	1	63.1	1.30
371	Piketon	0	SC	Yes	0	2	190.9	2.20

372	Piketon	0	SC	Yes	15	2	147.6	3.30
373	Piketon	1000	SC	Yes	0	3	294.0	2.90
374	Piketon	1000	SC	Yes	15	3	130.9	3.60
375	Piketon	2000	SC	No	0	4	242.8	2.70
376	Piketon	2000	SC	No	15	4	117.7	3.80
377	Piketon	0	SC	No	0	4	252.6	3.70
378	Piketon	0	SC	No	15	4	228.8	3.60
379	Piketon	0	SC	Yes	0	3	384.9	5.60
380	Piketon	0	SC	Yes	15	3	136.5	3.70
381	Piketon	2000	SC	Yes	0	2	146.2	1.60
382	Piketon	2000	SC	Yes	15	2	101.1	2.60
383	Piketon	1000	SC	No	0	1	423.4	4.70
384	Piketon	1000	SC	No	15	1	136.5	2.70
385	Piketon	2000	CS	Yes	0	1	258.6	1.90
386	Piketon	2000	CS	Yes	15	1	120.8	3.30
387	Piketon	1000	CS	No	0	2	299.9	2.30
388	Piketon	1000	CS	No	15	2	117.8	2.50
389	Piketon	1000	CS	No	0	3	248.1	2.10
390	Piketon	1000	CS	No	15	3	118.4	2.70
391	Piketon	2000	CS	Yes	0	4	299.2	2.60
392	Piketon	2000	CS	Yes	15	4	133.3	2.90
393	Piketon	0	CS	Yes	0	4	276.3	2.10
394	Piketon	0	CS	Yes	15	4	112.8	2.20
395	Piketon	2000	CS	No	0	3	277.8	2.30
396	Piketon	2000	CS	No	15	3	99.2	2.70
397	Piketon	0	CS	No	0	2	366.8	3.30
398	Piketon	0	CS	No	15	2	130.1	2.10
399	Piketon	0	CS	Yes	0	1	305.1	3.40
400	Piketon	0	CS	Yes	15	1	156.3	3.00
401	Piketon	1000	CS	Yes	0	1	238.7	2.00
402	Piketon	1000	CS	Yes	15	1	144.0	2.60
403	Piketon	2000	CS	No	0	2	271.1	3.80
404	Piketon	2000	CS	No	15	2	130.1	2.90
405	Piketon	0	CS	No	0	3	320.5	3.70
406	Piketon	0	CS	No	15	3	107.3	3.40
407	Piketon	1000	CS	Yes	0	4	180.5	1.60
408	Piketon	1000	CS	Yes	15	4	107.8	3.30
409	Piketon	1000	CS	No	0	4	153.2	1.20
410	Piketon	1000	CS	No	15	4	92.4	2.80
411	Piketon	2000	CS	Yes	0	3	252.3	3.50
412	Piketon	2000	CS	Yes	15	3	100.6	2.60
413	Piketon	1000	CS	Yes	0	2	343.6	3.10
414	Piketon	1000	CS	Yes	15	2	94.1	2.00
415	Piketon	2000	CS	No	0	1	232.2	2.50
416	Piketon	2000	CS	No	15	1	71.1	1.50
417	Piketon	0	CS	No	0	1	224.1	2.00
418	Piketon	0	CS	No	15	1	115.3	3.60

419	Piketon	0	CS	Yes	0	2	238.7	2.30
420	Piketon	0	CS	Yes	15	2	133.7	4.00
421	Piketon	1000	CS	Yes	0	3	379.3	3.20
422	Piketon	1000	CS	Yes	15	3	132.5	3.40
423	Piketon	2000	CS	No	0	4	384.6	3.90
424	Piketon	2000	CS	No	15	4	119.6	1.80
425	Piketon	0	CS	No	0	4	409.5	3.70
426	Piketon	0	CS	No	15	4	131.1	2.20
427	Piketon	0	CS	Yes	0	3	228.1	2.00
428	Piketon	0	CS	Yes	15	3	89.7	1.90
429	Piketon	2000	CS	Yes	0	2	392.9	3.50
430	Piketon	2000	CS	Yes	15	2	115.7	2.90
431	Piketon	1000	CS	No	0	1	295.6	2.70
432	Piketon	1000	CS	No	15	1	132.4	2.80
433	Hoytvill	1000	SS	No	0	1	187.6	1.00
434	Hoytvill	1000	SS	No	15	1	116.5	0.60
435	Hoytvill	2000	SS	No	0	1	124.7	0.70
436	Hoytvill	2000	SS	No	15	1	115.0	0.60
437	Hoytvill	0	SS	No	0	1	96.2	0.60
438	Hoytvill	0	SS	No	15	1	100.0	0.60
439	Hoytvill	1000	SS	No	0	2	112.0	0.70
440	Hoytvill	1000	SS	No	15	2	125.4	0.70
441	Hoytvill	2000	SS	Yes	0	1	197.5	1.00
442	Hoytvill	2000	SS	Yes	15	1	157.7	0.80
443	Hoytvill	2000	SS	Yes	0	2	169.6	0.90
444	Hoytvill	2000	SS	Yes	15	2	148.7	0.80
445	Hoytvill	1000	SS	Yes	0	1	133.7	0.80
446	Hoytvill	1000	SS	Yes	15	1	160.2	1.00
447	Hoytvill	2000	SS	Yes	0	3	138.8	0.80
448	Hoytvill	2000	SS	Yes	15	3	91.7	0.50
449	Hoytvill	0	SS	No	0	2	230.9	1.40
450	Hoytvill	0	SS	No	15	2	162.1	1.00
451	Hoytvill	0	SS	No	0	3	339.1	2.00
452	Hoytvill	0	SS	No	15	3	210.1	1.30
453	Hoytvill	2000	SS	No	0	2	281.2	1.60
454	Hoytvill	2000	SS	No	15	2	254.5	1.50
455	Hoytvill	2000	SS	No	0	3	309.3	1.90
456	Hoytvill	2000	SS	No	15	3	160.8	0.90
457	Hoytvill	0	SS	Yes	0	1	256.5	1.50
458	Hoytvill	0	SS	Yes	15	1	244.4	1.30
459	Hoytvill	1000	SS	Yes	0	2	180.5	1.10
460	Hoytvill	1000	SS	Yes	15	2	205.3	1.40
461	Hoytvill	0	SS	Yes	0	2	110.1	0.70
462	Hoytvill	0	SS	Yes	15	2	112.7	0.70
463	Hoytvill	1000	SS	Yes	0	3	175.1	1.00
464	Hoytvill	1000	SS	Yes	15	3	184.1	1.00
465	Hoytvill	2000	SS	No	0	4	190.2	1.10

466	Hoytvill	2000	SS	No	15	4	213.8	1.10
467	Hoytvill	1000	SS	No	0	3	274.4	1.30
468	Hoytvill	1000	SS	No	15	3	175.3	1.00
469	Hoytvill	1000	SS	No	0	4	133.4	0.80
470	Hoytvill	1000	SS	No	15	4	145.8	0.80
471	Hoytvill	0	SS	No	0	4	139.6	0.80
472	Hoytvill	0	SS	No	15	4	214.5	1.20
473	Hoytvill	1000	SS	Yes	0	4	96.4	0.50
474	Hoytvill	1000	SS	Yes	15	4	158.3	0.70
475	Hoytvill	0	SS	Yes	0	3	162.8	1.00
476	Hoytvill	0	SS	Yes	15	3	203.9	1.20
477	Hoytvill	2000	SS	Yes	0	4	168.9	1.10
478	Hoytvill	2000	SS	Yes	15	4	136.5	0.90
479	Hoytvill	0	SS	Yes	0	4	127.8	0.80
480	Hoytvill	0	SS	Yes	15	4	91.7	0.60
481	Hoytvill	1000	SC	No	0	1	180.6	1.10
482	Hoytvill	1000	SC	No	15	1	167.6	1.10
483	Hoytvill	1000	SC	No	0	1	170.9	1.00
484	Hoytvill	1000	SC	No	15	1	139.4	0.90
485	Hoytvill	0	SC	No	0	1	139.0	0.90
486	Hoytvill	0	SC	No	15	1	52.8	0.30
487	Hoytvill	0	SC	No	0	2	95.5	0.60
488	Hoytvill	0	SC	No	15	2	94.1	0.60
489	Hoytvill	1000	SC	Yes	0	1	151.2	0.90
490	Hoytvill	1000	SC	Yes	15	1	105.0	0.60
491	Hoytvill	0	SC	Yes	0	2	173.6	1.00
492	Hoytvill	0	SC	Yes	15	2	138.6	0.80
493	Hoytvill	0	SC	Yes	0	1	102.6	0.70
494	Hoytvill	0	SC	Yes	15	1	65.2	0.40
495	Hoytvill	2000	SC	Yes	0	3	237.4	1.20
496	Hoytvill	2000	SC	Yes	15	3	191.0	1.00
497	Hoytvill	2000	SC	No	0	2	294.9	1.70
498	Hoytvill	2000	SC	No	15	2	287.4	1.80
499	Hoytvill	2000	SC	No	0	3	235.8	1.40
500	Hoytvill	2000	SC	No	15	3	250.6	1.50
501	Hoytvill	1000	SC	No	0	2	116.7	0.70
502	Hoytvill	1000	SC	No	15	2	57.1	0.40
503	Hoytvill	1000	SC	No	0	3	185.9	1.10
504	Hoytvill	1000	SC	No	15	3	124.0	0.80
505	Hoytvill	2000	SC	Yes	0	1	153.7	1.00
506	Hoytvill	2000	SC	Yes	15	1	148.8	0.80
507	Hoytvill	1000	SC	Yes	0	2	169.2	1.00
508	Hoytvill	1000	SC	Yes	15	2	175.3	1.10
509	Hoytvill	1000	SC	Yes	0	2	124.1	0.80
510	Hoytvill	1000	SC	Yes	15	2	54.8	0.40
511	Hoytvill	0	SC	Yes	0	3	116.1	0.60
512	Hoytvill	0	SC	Yes	15	3	183.3	1.10

513	Hoytvill	0	SC	No	0	4	201.7	1.00
514	Hoytvill	0	SC	No	15	4	239.8	1.40
515	Hoytvill	0	SC	No	0	3	173.4	1.00
516	Hoytvill	0	SC	No	15	3	155.9	0.90
517	Hoytvill	2000	SC	No	0	4	250.4	1.30
518	Hoytvill	2000	SC	No	15	4	214.3	1.00
519	Hoytvill	2000	SC	No	0	4	111.9	0.60
520	Hoytvill	2000	SC	No	15	4	141.5	0.80
521	Hoytvill	0	SC	Yes	0	4	93.3	0.50
522	Hoytvill	0	SC	Yes	15	4	117.9	0.70
523	Hoytvill	2000	SC	Yes	0	3	131.7	0.70
524	Hoytvill	2000	SC	Yes	15	3	180.1	0.80
525	Hoytvill	2000	SC	Yes	0	4	91.3	0.60
526	Hoytvill	2000	SC	Yes	15	4	131.6	0.90
527	Hoytvill	1000	SC	Yes	0	4	331.6	1.90
528	Hoytvill	1000	SC	Yes	15	4	92.3	0.50
529	Hoytvill	0	CS	No	0	1	125.3	0.80
530	Hoytvill	0	CS	No	15	1	85.5	0.50
531	Hoytvill	2000	CS	No	0	1	187.2	1.10
532	Hoytvill	2000	CS	No	15	1	108.8	0.70
533	Hoytvill	0	CS	No	0	2	72.1	0.40
534	Hoytvill	0	CS	No	15	2	54.9	0.40
535	Hoytvill	0	CS	No	0	3	182.0	1.10
536	Hoytvill	0	CS	No	15	3	130.0	0.70
537	Hoytvill	0	CS	Yes	0	1	83.1	0.50
538	Hoytvill	0	CS	Yes	15	1	87.7	0.50
539	Hoytvill	1000	CS	Yes	0	1	147.9	0.90
540	Hoytvill	1000	CS	Yes	15	1	72.3	0.40
541	Hoytvill	2000	CS	Yes	0	1	114.9	0.70
542	Hoytvill	2000	CS	Yes	15	1	64.2	0.40
543	Hoytvill	1000	CS	Yes	0	2	124.0	0.70
544	Hoytvill	1000	CS	Yes	15	2	139.7	0.90
545	Hoytvill	2000	CS	No	0	2	283.9	1.70
546	Hoytvill	2000	CS	No	15	2	286.8	1.70
547	Hoytvill	0	CS	No	0	4	200.7	1.20
548	Hoytvill	0	CS	No	15	4	204.9	1.30
549	Hoytvill	2000	CS	No	0	3	298.7	1.80
550	Hoytvill	2000	CS	No	15	3	248.0	1.50
551	Hoytvill	2000	CS	No	0	4	266.5	1.60
552	Hoytvill	2000	CS	No	15	4	157.4	0.80
553	Hoytvill	2000	CS	Yes	0	2	305.3	1.80
554	Hoytvill	2000	CS	Yes	15	2	211.4	1.30
555	Hoytvill	2000	CS	Yes	0	3	204.5	1.20
556	Hoytvill	2000	CS	Yes	15	3	155.5	0.90
557	Hoytvill	1000	CS	Yes	0	3	91.1	0.60
558	Hoytvill	1000	CS	Yes	15	3	96.3	0.50
559	Hoytvill	2000	CS	Yes	0	4	123.5	0.80

560	Hoytvill	2000	CS	Yes	15	4	113.5	0.70
561	Hoytvill	1000	CS	No	0	1	299.8	1.50
562	Hoytvill	1000	CS	No	15	1	268.5	1.50
563	Hoytvill	1000	CS	No	0	2	254.2	1.50
564	Hoytvill	1000	CS	No	15	2	198.9	1.00
565	Hoytvill	1000	CS	No	0	3	260.9	1.40
566	Hoytvill	1000	CS	No	15	3	195.7	1.10
567	Hoytvill	1000	CS	No	0	4	231.7	1.20
568	Hoytvill	1000	CS	No	15	4	190.0	1.00
569	Hoytvill	1000	CS	Yes	0	4	174.1	1.00
570	Hoytvill	1000	CS	Yes	15	4	107.6	0.50
571	Hoytvill	0	CS	Yes	0	2	215.4	1.30
572	Hoytvill	0	CS	Yes	15	2	208.2	1.20
573	Hoytvill	0	CS	Yes	0	3	128.8	0.80
574	Hoytvill	0	CS	Yes	15	3	168.3	1.00
575	Hoytvill	0	CS	Yes	0	4	134.1	0.80
576	Hoytvill	0	CS	Yes	15	4	138.5	0.80

The ANOVA Procedure of SAS

Class Level Information

Class	Levels	Values
Site	4	Alabama Hoytvill Indiana Piketon
Gypsum	3	0 1000 2000
CR	3	CS SC SS
CC	2	No Yes
Depth	2	0 15
Rep	4	1 2 3 4

Number of 576

Number of 576

The ANOVA Procedure

Dependent Variable: SMB

Source	DF	Sum of Squ	Mean Squa	F Value	Pr > F
Model	143	3685121	25770.08	6.38	<.0001
Error	432	1745482	4040.468		
Corrected ⁻	575	5430603			

R-Square	Coeff Var	Root MSE	SMB Mean
0.678584	35.96834	63.56468	176.724

Source	DF	Anova SS	Mean Squa	F Value	Pr > F
Site*Depth	3	365695.4	121898.5	30.17	<.0001
Site	3	813173.3	271057.8	67.09	<.0001
Gypsum	2	307961.5	153980.7	38.11	<.0001
CR	2	14085.98	7042.99	1.74	0.1762
CC	1	10354.76	10354.76	2.56	0.1101
Depth	1	1027199	1027199	254.23	<.0001
Gypsum*Cl	4	60069.7	15017.43	3.72	0.0055
Gypsum*C(	2	5691.014	2845.507	0.7	0.495
Gypsum*D	2	18423.89	9211.944	2.28	0.1035
CR*CC	2	1296.296	648.148	0.16	0.8518
CR*Depth	2	1684.369	842.185	0.21	0.8119
CC*Depth	1	2760.627	2760.627	0.68	0.4089
Gypsum*Cl	4	29112.79	7278.198	1.8	0.1276
Gypsum*Cl	4	7004.256	1751.064	0.43	0.7845
Gypsum*C(	2	7682.527	3841.264	0.95	0.3873
CR*CC*De	2	1500.007	750.003	0.19	0.8307
Site*Gyps*	106	1011425	9541.749	2.36	<.0001

The ANOVA Procedure

Dependent Variable: qR

Source	DF	Sum of Squ	Mean Squa	F Value	Pr > F
Model	143	790.9049	5.530804	4.51	<.0001
Error	432	529.48	1.225648		
Corrected ⁻	575	1320.385			

R-Square	Coeff Var	Root MSE	qR Mean
0.598996	53.04307	1.10709	2.087153

Source	DF	Anova SS	Mean Squa	F Value	Pr > F
Site*Depth	3	20.48792	6.829306	5.57	0.0009
Site	3	375.422	125.1407	102.1	<.0001
Gypsum	2	19.00545	9.502726	7.75	0.0005
CR	2	66.02691	33.01345	26.94	<.0001
CC	1	0.5625	0.5625	0.46	0.4985
Depth	1	8.027778	8.027778	6.55	0.0108
Gypsum*Cl	4	6.817882	1.704471	1.39	0.2362
Gypsum*C(	2	3.955104	1.977552	1.61	0.2004

Gypsum*D	2	8.851076	4.425538	3.61	0.0278
CR*CC	2	0.121979	0.06099	0.05	0.9515
CR*Depth	2	19.74941	9.874705	8.06	0.0004
CC*Depth	1	2.918403	2.918403	2.38	0.1235
Gypsum*Cl	4	13.00448	3.25112	2.65	0.0327
Gypsum*Cl	4	3.192674	0.798168	0.65	0.6263
Gypsum*C	2	0.965035	0.482517	0.39	0.6748
CR*CC*De	2	0.904618	0.452309	0.37	0.6916
Site*Gyps*	106	240.8917	2.272563	1.85	<.0001

The ANOVA Procedure

Dependent Variable: SOC

Source	DF	Sum of Squ	Mean Squa	F Value	Pr > F
Model	143	146.948	1.027608	23.08	<.0001
Error	432	19.23743	0.044531		
Corrected	575	166.1854			

R-Square	Coeff Var	Root MSE	SOC Mean
0.884241	19.54647	0.211024	1.079601

Source	DF	Anova SS	Mean Squa	F Value	Pr > F
Site*Depth	3	6.545321	2.181774	48.99	<.0001
Site	3	115.1533	38.38445	861.97	<.0001
Gypsum	2	0.046039	0.023019	0.52	0.5967
CR	2	2.886871	1.443436	32.41	<.0001
CC	1	0.011827	0.011827	0.27	0.6066
Depth	1	16.3654	16.3654	367.51	<.0001
Gypsum*Cl	4	0.14539	0.036348	0.82	0.5153
Gypsum*C	2	0.09785	0.048925	1.1	0.3342
Gypsum*D	2	0.085976	0.042988	0.97	0.3817
CR*CC	2	0.000907	0.000454	0.01	0.9899
CR*Depth	2	0.226292	0.113146	2.54	0.08
CC*Depth	1	0.034379	0.034379	0.77	0.3801
Gypsum*Cl	4	0.492533	0.123133	2.77	0.0272
Gypsum*Cl	4	0.015465	0.003866	0.09	0.9865
Gypsum*C	2	0.040935	0.020467	0.46	0.6318
CR*CC*De	2	0.014413	0.007206	0.16	0.8506
Site*Gyps*	106	4.785051	0.045142	1.01	0.4523

The ANOVA Procedure

Dependent Variable: TN

Source	DF	Sum of Squ	Mean Squa	F Value	Pr > F
Model	143	2.086201	0.014589	37.5	<.0001
Error	432	0.16806	0.000389		
Corrected ⁻	575	2.254261			

R-Square	Coeff Var	Root MSE	TN Mean
0.925448	16.31095	0.019724	0.120924

Source	DF	Anova SS	Mean Squa	F Value	Pr > F
Site*Depth	3	0.055873	0.018624	47.87	<.0001
Site	3	1.831595	0.610532	1569.38	<.0001
Gypsum	2	0.002129	0.001065	2.74	0.0659
CR	2	0.010418	0.005209	13.39	<.0001
CC	1	0.000374	0.000374	0.96	0.3275
Depth	1	0.125729	0.125729	323.19	<.0001
Gypsum*C	4	0.003184	0.000796	2.05	0.0871
Gypsum*C	2	0.001964	0.000982	2.52	0.0813
Gypsum*D	2	0.000224	0.000112	0.29	0.7502
CR*CC	2	0.000267	0.000133	0.34	0.7102
CR*Depth	2	0.00072	0.00036	0.93	0.397
CC*Depth	1	0.001567	0.001567	4.03	0.0454
Gypsum*C	4	0.00191	0.000477	1.23	0.2986
Gypsum*C	4	0.000239	5.97E-05	0.15	0.9614
Gypsum*C	2	0.000733	0.000366	0.94	0.3907
CR*CC*De	2	1.24E-05	6.17E-06	0.02	0.9843
Site*Gyps*	106	0.049264	0.000465	1.19	0.113

The ANOVA Procedure

Dependent Variable: AC

Source	DF	Sum of Squ	Mean Squa	F Value	Pr > F
Model	143	13442543	94003.8	24.78	<.0001
Error	432	1638703	3793.29		
Corrected ⁻	575	15081247			

R-Square	Coeff Var	Root MSE	AC Mean

0.891342	16.14508	61.58973	381.4767

Source	DF	Anova SS	Mean Squa	F Value	Pr > F
Site*Depth	3	413440.7	137813.6	36.33	<.0001
Site	3	8965307	2988436	787.82	<.0001
Gypsum	2	129131.3	64565.63	17.02	<.0001
CR	2	486511.6	243255.8	64.13	<.0001
CC	1	40337.38	40337.38	10.63	0.0012
Depth	1	2126444	2126444	560.58	<.0001
Gypsum*Cl	4	19018.37	4754.594	1.25	0.2877
Gypsum*C	2	677.274	338.637	0.09	0.9146
Gypsum*D	2	19333.39	9666.696	2.55	0.0794
CR*CC	2	7179.326	3589.663	0.95	0.389
CR*Depth	2	27465.45	13732.73	3.62	0.0276
CC*Depth	1	101.002	101.002	0.03	0.8705
Gypsum*Cl	4	8358.843	2089.711	0.55	0.6985
Gypsum*Cl	4	9155.406	2288.852	0.6	0.6604
Gypsum*C	2	5513.466	2756.733	0.73	0.4841
CR*CC*Dep	2	63.771	31.885	0.01	0.9916
Site*Gyps*	106	1184504	11174.57	2.95	<.0001

The ANOVA Procedure

Dependent Variable: CWC

Source	DF	Sum of Squ	Mean Squa	F Value	Pr > F
Model	143	36261.67	253.5781	7.18	<.0001
Error	432	15265.73	35.33734		
Corrected T	575	51527.4			

R-Square	Coeff Var	Root MSE	CWC Mean
0.703736	32.10784	5.944522	18.51424

Source	DF	Anova SS	Mean Squa	F Value	Pr > F
Site*Depth	3	738.6134	246.2045	6.97	0.0001
Site	3	24292.65	8097.551	229.15	<.0001
Gypsum	2	243.9442	121.9721	3.45	0.0326
CR	2	76.22878	38.11439	1.08	0.341
CC	1	53.77778	53.77778	1.52	0.218
Depth	1	6583.97	6583.97	186.32	<.0001
Gypsum*Cl	4	164.6315	41.15788	1.16	0.3257
Gypsum*C	2	1.97712	0.98856	0.03	0.9724
Gypsum*D	2	51.22462	25.61231	0.72	0.485

CR*CC	2	112.0973	56.04866	1.59	0.2059
CR*Depth	2	9.08712	4.54356	0.13	0.8794
CC*Depth	1	39.0625	39.0625	1.11	0.2937
Gypsum*CI	4	317.4578	79.36444	2.25	0.0633
Gypsum*CI	4	18.29882	4.5747	0.13	0.9717
Gypsum*C	2	39.25198	19.62599	0.56	0.5743
CR*CC*De	2	8.8251	4.41255	0.12	0.8826
Site*Gyps*	106	3510.573	33.11862	0.94	0.6513

The ANOVA Procedure

Dependent Variable: HWC

Source	DF	Sum of Squ	Mean Squa	F Value	Pr > F
Model	143	167913.5	1174.22	7.02	<.0001
Error	432	72254.82	167.2565		
Corrected	575	240168.3			

R-Square	Coeff Var	Root MSE	HWC Mean
0.699149	26.46063	12.93277	48.87552

Source	DF	Anova SS	Mean Squa	F Value	Pr > F
Site*Depth	3	14902.27	4967.424	29.7	<.0001
Site	3	48008.15	16002.72	95.68	<.0001
Gypsum	2	7825.435	3912.718	23.39	<.0001
CR	2	87.85542	43.92771	0.26	0.7691
CC	1	726.5271	726.5271	4.34	0.0377
Depth	1	66660.79	66660.79	398.55	<.0001
Gypsum*CI	4	1762.587	440.6469	2.63	0.0337
Gypsum*C	2	89.68066	44.84033	0.27	0.765
Gypsum*D	2	634.7564	317.3782	1.9	0.1512
CR*CC	2	216.376	108.188	0.65	0.5242
CR*Depth	2	348.7629	174.3815	1.04	0.3534
CC*Depth	1	51.54043	51.54043	0.31	0.5791
Gypsum*CI	4	834.1358	208.534	1.25	0.2904
Gypsum*CI	4	378.9145	94.72862	0.57	0.6872
Gypsum*C	2	96.18462	48.09231	0.29	0.7503
CR*CC*De	2	44.58931	22.29465	0.13	0.8752
Site*Gyps*	106	25244.93	238.1597	1.42	0.0079

The ANOVA Procedure

Dependent Variable: CPI

Source	DF	Sum of Squ	Mean Squa	F Value	Pr > F
Model	143	14.17148	0.099101	1.6	0.0002
Error	432	26.70525	0.061818		
Corrected ⁻	575	40.87673			

R-Square	Coeff Var	Root MSE	CPI Mean
0.346688	24.60854	0.248632	1.010347

Source	DF	Anova SS	Mean Squa	F Value	Pr > F
Site*Depth	3	0.305053	0.101684	1.64	0.1783
Site	3	0.284676	0.094892	1.54	0.2047
Gypsum	2	0.041156	0.020578	0.33	0.717
CR	2	4.804152	2.402076	38.86	<.0001
CC	1	0.003701	0.003701	0.06	0.8068
Depth	1	0.244201	0.244201	3.95	0.0475
Gypsum*CI	4	0.083088	0.020772	0.34	0.8537
Gypsum*C	2	0.147318	0.073659	1.19	0.3047
Gypsum*D	2	0.150718	0.075359	1.22	0.2965
CR*CC	2	0.00538	0.00269	0.04	0.9574
CR*Depth	2	0.285121	0.142561	2.31	0.1009
CC*Depth	1	0.077469	0.077469	1.25	0.2636
Gypsum*CI	4	0.606111	0.151528	2.45	0.0455
Gypsum*CI	4	0.047907	0.011977	0.19	0.9416
Gypsum*C	2	0.014672	0.007336	0.12	0.8881
CR*CC*Dep	2	0.043427	0.021714	0.35	0.704
Site*Gyps*	106	7.027329	0.066296	1.07	0.3121

The ANOVA Procedure

Dependent Variable: NPI

Source	DF	Sum of Squ	Mean Squa	F Value	Pr > F
Model	143	12.74384	0.089118	2	<.0001
Error	432	19.2386	0.044534		
Corrected ⁻	575	31.98244			

R-Square	Coeff Var	Root MSE	NPI Mean
0.398464	21.11403	0.21103	0.999479

Source	DF	Anova SS	Mean Squa	F Value	Pr > F
Site*Depth	3	0.527626	0.175875	3.95	0.0085
Site	3	1.881059	0.62702	14.08	<.0001
Gypsum	2	0.481079	0.24054	5.4	0.0048
CR	2	0.447726	0.223863	5.03	0.0069
CC	1	0.156684	0.156684	3.52	0.0614
Depth	1	1.092025	1.092025	24.52	<.0001
Gypsum*C	4	0.196651	0.049163	1.1	0.3541
Gypsum*C	2	0.617918	0.308959	6.94	0.0011
Gypsum*D	2	0.017379	0.00869	0.2	0.8228
CR*CC	2	0.050984	0.025492	0.57	0.5646
CR*Depth	2	0.034789	0.017394	0.39	0.6769
CC*Depth	1	0.2704	0.2704	6.07	0.0141
Gypsum*C	4	0.275664	0.068916	1.55	0.1875
Gypsum*C	4	0.014895	0.003724	0.08	0.9874
Gypsum*C	2	0.056629	0.028315	0.64	0.53
CR*CC*De	2	0.007353	0.003677	0.08	0.9208
Site*Gyps*	106	6.614982	0.062405	1.4	0.0106

The ANOVA Procedure

Dependent Variable: MBCCLI

Source	DF	Sum of Squ	Mean Squa	F Value	Pr > F
Model	143	158.8162	1.110603	3.25	<.0001
Error	432	147.7727	0.342067		
Corrected	575	306.5889			

R-Square	Coeff Var	Root MSE	MBCCLI Mean
0.51801	50.74517	0.584865	1.152552

Source	DF	Anova SS	Mean Squa	F Value	Pr > F
Site*Depth	3	7.072789	2.357596	6.89	0.0002
Site	3	27.99661	9.332203	27.28	<.0001
Gypsum	2	12.08591	6.042954	17.67	<.0001
CR	2	21.47727	10.73863	31.39	<.0001
CC	1	0.153077	0.153077	0.45	0.5039
Depth	1	0.861029	0.861029	2.52	0.1133
Gypsum*C	4	3.036085	0.759021	2.22	0.0661
Gypsum*C	2	0.297282	0.148641	0.43	0.6478
Gypsum*D	2	1.252615	0.626307	1.83	0.1615
CR*CC	2	0.390388	0.195194	0.57	0.5656

CR*Depth	2	2.638751	1.319376	3.86	0.0219
CC*Depth	1	0.789877	0.789877	2.31	0.1293
Gypsum*Cl	4	4.969233	1.242308	3.63	0.0063
Gypsum*Cl	4	0.60884	0.15221	0.44	0.7761
Gypsum*C	2	0.272572	0.136286	0.4	0.6716
CR*CC*De	2	0.01715	0.008575	0.03	0.9752
Site*Gyps*	106	74.89675	0.706573	2.07	<.0001

The ANOVA Procedure

Dependent Variable: ACCLI

Source	DF	Sum of Squ	Mean Squa	F Value	Pr > F
Model	143	26.63421	0.186253	2.44	<.0001
Error	432	32.94288	0.076257		
Corrected	575	59.57709			

R-Square	Coeff Var	Root MSE	ACCLI Mean
0.447055	25.63625	0.276146	1.07717

Source	DF	Anova SS	Mean Squa	F Value	Pr > F
Site*Depth	3	6.510762	2.170254	28.46	<.0001
Site	3	1.65406	0.551353	7.23	<.0001
Gypsum	2	1.293969	0.646985	8.48	0.0002
CR	2	0.091701	0.045851	0.6	0.5486
CC	1	0.388025	0.388025	5.09	0.0246
Depth	1	2.550675	2.550675	33.45	<.0001
Gypsum*Cl	4	0.479328	0.119832	1.57	0.1809
Gypsum*C	2	0.055565	0.027782	0.36	0.6949
Gypsum*D	2	0.041355	0.020677	0.27	0.7626
CR*CC	2	0.077643	0.038822	0.51	0.6014
CR*Depth	2	0.032326	0.016163	0.21	0.8091
CC*Depth	1	0.400584	0.400584	5.25	0.0224
Gypsum*Cl	4	0.799734	0.199934	2.62	0.0344
Gypsum*Cl	4	0.188242	0.047061	0.62	0.6505
Gypsum*C	2	0.013955	0.006977	0.09	0.9126
CR*CC*De	2	0.009585	0.004792	0.06	0.9391
Site*Gyps*	106	12.0467	0.113648	1.49	0.0031

The ANOVA Procedure

Dependent Variable: CWCCLI

Source	DF	Sum of Squ	Mean Squa	F Value	Pr > F
Model	143	76.68552	0.536262	2.59	<.0001
Error	432	89.39323	0.206929		
Corrected ⁻	575	166.0787			

R-Square	Coeff Var	Root MSE	CWCCLI Mean
0.461742	41.83136	0.454894	1.087448

Source	DF	Anova SS	Mean Squa	F Value	Pr > F
Site*Depth	3	22.5492	7.516401	36.32	<.0001
Site	3	2.279649	0.759883	3.67	0.0123
Gypsum	2	1.67497	0.837485	4.05	0.0181
CR	2	9.762153	4.881077	23.59	<.0001
CC	1	0.362504	0.362504	1.75	0.1863
Depth	1	3.140279	3.140279	15.18	0.0001
Gypsum*CI	4	0.39413	0.098533	0.48	0.7532
Gypsum*C	2	0.14448	0.07224	0.35	0.7055
Gypsum*D	2	1.326832	0.663416	3.21	0.0415
CR*CC	2	0.864157	0.432078	2.09	0.1252
CR*Depth	2	1.872392	0.936196	4.52	0.0114
CC*Depth	1	0.029613	0.029613	0.14	0.7054
Gypsum*CI	4	3.14877	0.787193	3.8	0.0047
Gypsum*CI	4	0.353508	0.088377	0.43	0.7891
Gypsum*C	2	0.416023	0.208012	1.01	0.3668
CR*CC*De	2	0.235086	0.117543	0.57	0.5671
Site*Gyps*	106	28.13178	0.265394	1.28	0.0453

The ANOVA Procedure

Dependent Variable: HWCCLI

Source	DF	Sum of Squ	Mean Squa	F Value	Pr > F
Model	143	74.52376	0.521145	2.79	<.0001
Error	432	80.55328	0.186466		
Corrected ⁻	575	155.077			

R-Square	Coeff Var	Root MSE	HWCCLI Mean
0.48056	40.34429	0.431817	1.07033

Source	DF	Anova SS	Mean Squa	F Value	Pr > F
Site*Depth	3	6.53523	2.17841	11.68	<.0001
Site	3	6.875877	2.291959	12.29	<.0001
Gypsum	2	3.673318	1.836659	9.85	<.0001
CR	2	13.3946	6.697299	35.92	<.0001
CC	1	0.058604	0.058604	0.31	0.5754
Depth	1	1.084202	1.084202	5.81	0.0163
Gypsum*Cl	4	0.947869	0.236967	1.27	0.2807
Gypsum*C	2	0.461726	0.230863	1.24	0.291
Gypsum*D	2	1.183663	0.591831	3.17	0.0428
CR*CC	2	0.327007	0.163503	0.88	0.4168
CR*Depth	2	2.585189	1.292594	6.93	0.0011
CC*Depth	1	0.383884	0.383884	2.06	0.1521
Gypsum*Cl	4	2.512705	0.628176	3.37	0.0099
Gypsum*Cl	4	0.383415	0.095854	0.51	0.7254
Gypsum*C	2	0.115089	0.057544	0.31	0.7346
CR*CC*Dep	2	0.012094	0.006047	0.03	0.9681
Site*Gyps*	106	33.98929	0.320654	1.72	<.0001

The ANOVA Procedure

Dependent Variable: MBCCMI

Source	DF	Sum of Squ	Mean Squa	F Value	Pr > F
Model	143	78.66598	0.550112	4.02	<.0001
Error	432	59.18243	0.136996		
Corrected T	575	137.8484			

R-Square	Coeff Var	Root MSE	MBCCMI Mean
0.57067	34.95627	0.37013	1.058838

Source	DF	Anova SS	Mean Squa	F Value	Pr > F
Site*Depth	3	2.606544	0.868848	6.34	0.0003
Site	3	20.71385	6.904615	50.4	<.0001
Gypsum	2	13.10536	6.55268	47.83	<.0001
CR	2	1.593297	0.796648	5.82	0.0032
CC	1	0.293769	0.293769	2.14	0.1438
Depth	1	0.910124	0.910124	6.64	0.0103
Gypsum*Cl	4	3.135235	0.783809	5.72	0.0002
Gypsum*C	2	0.106028	0.053014	0.39	0.6793
Gypsum*D	2	0.133522	0.066761	0.49	0.6146
CR*CC	2	0.067913	0.033956	0.25	0.7806
CR*Depth	2	0.032299	0.01615	0.12	0.8888

CC*Depth	1	0.067516	0.067516	0.49	0.483
Gypsum*Cl	4	1.353954	0.338488	2.47	0.044
Gypsum*Cl	4	0.111931	0.027983	0.2	0.936
Gypsum*C	2	0.15788	0.07894	0.58	0.5625
CR*CC*De	2	0.048521	0.02426	0.18	0.8378
Site*Gyps*	106	34.22825	0.322908	2.36	<.0001

The ANOVA Procedure

Dependent Variable: ACCMI

Source	DF	Sum of Squ	Mean Squa	F Value	Pr > F
Model	143	33.90085	0.237069	4.92	<.0001
Error	432	20.80253	0.048154		
Corrected	575	54.70337			

R-Square	Coeff Var	Root MSE	ACCMI Mean
0.619721	20.83739	0.21944	1.053108

Source	DF	Anova SS	Mean Squa	F Value	Pr > F
Site*Depth	3	4.18387	1.394623	28.96	<.0001
Site	3	2.660268	0.886756	18.42	<.0001
Gypsum	2	1.658753	0.829377	17.22	<.0001
CR	2	7.509167	3.754583	77.97	<.0001
CC	1	0.267565	0.267565	5.56	0.0189
Depth	1	0.511511	0.511511	10.62	0.0012
Gypsum*Cl	4	0.400493	0.100123	2.08	0.0826
Gypsum*C	2	0.003165	0.001583	0.03	0.9677
Gypsum*D	2	0.154164	0.077082	1.6	0.2029
CR*CC	2	0.116626	0.058313	1.21	0.2989
CR*Depth	2	0.902031	0.451015	9.37	0.0001
CC*Depth	1	0.004548	0.004548	0.09	0.7587
Gypsum*Cl	4	0.068144	0.017036	0.35	0.8414
Gypsum*Cl	4	0.103115	0.025779	0.54	0.7098
Gypsum*C	2	0.043874	0.021937	0.46	0.6344
CR*CC*De	2	0.036645	0.018322	0.38	0.6838
Site*Gyps*	106	15.27691	0.144122	2.99	<.0001

The ANOVA Procedure

Dependent Variable: CWCCMI

Source	DF	Sum of Squ	Mean Squa	F Value	Pr > F
Model	143	32.27371	0.22569	2.4	<.0001
Error	432	40.60245	0.093987		
Corrected ⁻	575	72.87616			

R-Square	Coeff Var	Root MSE	CWCCMI Mean
0.442857	30.366	0.306573	1.009594

Source	DF	Anova SS	Mean Squa	F Value	Pr > F
Site*Depth	3	13.88524	4.628414	49.25	<.0001
Site	3	4.901335	1.633778	17.38	<.0001
Gypsum	2	0.957883	0.478942	5.1	0.0065
CR	2	0.210267	0.105134	1.12	0.3277
CC	1	0.078013	0.078013	0.83	0.3628
Depth	1	0.019108	0.019108	0.2	0.6523
Gypsum*C	4	0.291817	0.072954	0.78	0.5411
Gypsum*C	2	0.01485	0.007425	0.08	0.9241
Gypsum*D	2	0.11926	0.05963	0.63	0.5307
CR*CC	2	0.400039	0.20002	2.13	0.1203
CR*Depth	2	0.012161	0.00608	0.06	0.9374
CC*Depth	1	0.223264	0.223264	2.38	0.124
Gypsum*C	4	1.197324	0.299331	3.18	0.0135
Gypsum*C	4	0.050484	0.012621	0.13	0.9697
Gypsum*C	2	0.141086	0.070543	0.75	0.4727
CR*CC*De	2	0.103529	0.051764	0.55	0.5769
Site*Gyps*	106	9.668046	0.091208	0.97	0.5651

The ANOVA Procedure

Dependent Variable: HWCCMI

Source	DF	Sum of Squ	Mean Squa	F Value	Pr > F
Model	143	24.59707	0.172007	2.88	<.0001
Error	432	25.82971	0.059791		
Corrected ⁻	575	50.42677			

R-Square	Coeff Var	Root MSE	HWCCMI Mean
0.487778	24.70955	0.244522	0.989585

Source	DF	Anova SS	Mean Squa	F Value	Pr > F

Site*Depth	3	2.270912	0.756971	12.66	<.0001
Site	3	6.984104	2.328035	38.94	<.0001
Gypsum	2	3.919835	1.959917	32.78	<.0001
CR	2	0.443095	0.221548	3.71	0.0254
CC	1	0.19982	0.19982	3.34	0.0682
Depth	1	0.380663	0.380663	6.37	0.012
Gypsum*CI	4	0.973818	0.243454	4.07	0.003
Gypsum*C(	2	0.01466	0.00733	0.12	0.8847
Gypsum*D	2	0.103996	0.051998	0.87	0.4198
CR*CC	2	0.073904	0.036952	0.62	0.5395
CR*Depth	2	0.070868	0.035434	0.59	0.5533
CC*Depth	1	0.000521	0.000521	0.01	0.9257
Gypsum*CI	4	0.368706	0.092177	1.54	0.1891
Gypsum*CI	4	0.042375	0.010594	0.18	0.9501
Gypsum*C(	2	0.010747	0.005373	0.09	0.9141
CR*CC*Dep	2	0.034645	0.017322	0.29	0.7486
Site*Gyps*	106	8.704399	0.082117	1.37	0.0152

The ANOVA Procedure

Level of Site	Level of Depth	N	SMB Mean	Std Dev	qR Mean	Std Dev	SOC Mean	Std Dev
Alabama	0	72	147.4472	42.26933	1.954167	0.938824	0.8	0.22
Alabama	15	72	92.6375	36.86833	2.715278	2.06129	0.4	0.20
Hoytvill	0	72	178.8681	68.53737	1.038889	0.383665	1.7	0.13
Hoytvill	15	72	154.0278	58.32482	0.890278	0.342386	1.7	0.17
Indiana	0	72	266.5944	118.2186	1.9	0.93236	1.5	0.30
Indiana	15	72	167	83.15917	1.805556	1.209922	1.0	0.34
Piketon	0	72	282.9042	109.1401	2.983333	1.272903	1.0	0.18
Piketon	15	72	124.3125	36.98939	3.409722	1.886659	0.4	0.13

The ANOVA Procedure

t Tests (LSD) for SMB

Note: This test controls the Type I comparisonwise error rate, not the experimentwise error rate.

Alpha	0.05
Error Degre	432
Error Mear	4040.468

Critical Valu	1.96547
Least Signif	14.724

Means with the same letter are not significantly different.

t Grouping	Mean	N	Site
A	216.797	144	Indiana
A			
A	203.608	144	Piketon
B	166.448	144	Hoytvill
C	120.042	144	Alabama

The ANOVA Procedure

t Tests (LSD) for qR

Note: This test controls the Type I comparisonwise error rate, not the experimentwise error rate.

Alpha	0.05
Error Degre	432
Error Mear	1.225648
Critical Valu	1.96547
Least Signif	0.2564

Means with the same letter are not significantly different.

t Grouping	Mean	N	Site
A	3.1965	144	Piketon
B	2.3347	144	Alabama
C	1.8528	144	Indiana
D	0.9646	144	Hoytvill

The ANOVA Procedure

t Tests (LSD) for SOC

Note:	This test controls the Type I comparisonwise error rate, not the experimentwise error rate.

Alpha	0.05
Error Degre	432
Error Mear	0.044531
Critical Valu	1.96547
Least Signif	0.0489

Means with the same letter are
not significantly different.

t Grouping	Mean	N	Site
A	1.73083	144	Hoytvill
B	1.25639	144	Indiana
C	0.69306	144	Piketon
D	0.63813	144	Alabama

The ANOVA Procedure

t Tests (LSD) for TN

Note:	This test controls the Type I comparisonwise error rate, not the experimentwise error rate.

Alpha	0.05
Error Degre	432
Error Mear	0.000389
Critical Valu	1.96547
Least Signif	0.0046

Means with the same letter are
not significantly different.

t Grouping	Mean	N	Site
A	0.203215	144	Hoytvill
B	0.139729	144	Indiana
C	0.085924	144	Piketon
D	0.054826	144	Alabama

The ANOVA Procedure

t Tests (LSD) for AC

Note: This test controls the Type I comparisonwise error rate, not the experimentwise error rate.

Alpha	0.05
Error Degre	432
Error Mear	3793.294
Critical Valu	1.96547
Least Signif	14.266

Means with the same letter are not significantly different.

t Grouping	Mean	N	Site
A	549.079	144	Hoytvill
B	404.019	144	Indiana
C	374.673	144	Piketon
D	198.135	144	Alabama

The ANOVA Procedure

t Tests (LSD) for CWC

Note: This test controls the Type I comparisonwise error rate, not the experimentwise error rate.

Alpha	0.05
Error Degre	432
Error Mear	35.33734
Critical Valu	1.96547
Least Signif	1.3769

Means with the same letter are not significantly different.

t Grouping	Mean	N	Site
A	28.5639	144	Indiana
B	19.275	144	Hoytvill
C	15.1937	144	Piketon
D	11.0243	144	Alabama

The ANOVA Procedure

t Tests (LSD) for HWC

Note: This test controls the Type I comparisonwise error rate, not the experimentwise error rate.

Alpha	0.05
Error Degre	432
Error Mear	167.2565
Critical Valu	1.96547
Least Signif	2.9957

Means with the same letter are
not significantly different.

t Grouping	Mean	N	Site
A	58.558	144	Piketon
B	55.089	144	Hoytvill
C	47.038	144	Indiana
D	34.817	144	Alabama

The ANOVA Procedure

t Tests (LSD) for CPI

Note: This test controls the Type I comparisonwise error rate, not the experimentwise error rate.

Alpha	0.05
Error Degre	432
Error Mear	0.061818
Critical Valu	1.96547
Least Signif	0.0576

Means with the same letter are
not significantly different.

t Grouping		Mean	N	Site
	A	1.03958	144	Hoytvill
	A			
B	A	1.01618	144	Alabama

B	A			
B	A	1.00819	144	Piketon
B				
B		0.97743	144	Indiana

The ANOVA Procedure

t Tests (LSD) for NPI

Note: This test controls the Type I comparisonwise error rate, not the experimentwise error rate.

Alpha	0.05
Error Degre	432
Error Mear	0.044534
Critical Valı	1.96547
Least Signif	0.0489

Means with the same letter are
not significantly different.

t Grouping	Mean	N	Site
A	1.08507	144	Alabama
B	1.01597	144	Hoytvill
C	0.95972	144	Indiana
C			
C	0.93715	144	Piketon

The ANOVA Procedure

t Tests (LSD) for MBCCLI

Note: This test controls the Type I comparisonwise error rate, not the experimentwise error rate.

Alpha	0.05
Error Degre	432
Error Mear	0.342066
Critical Valı	1.96547
Least Signif	0.1355

Means with the same letter are

not significantly different.

t Grouping	Mean	N	Site
A	1.46424	144	Indiana
B	1.21132	144	Alabama
B			
B	1.07958	144	Hoytvill
C	0.85507	144	Piketon

First paper

The ANOVA Procedure

First paper

The ANOVA Procedure

t Tests (LSD) for ACCLI

Note: This test controls the Type I comparisonwise error rate, not the experimentwise error rate.

Alpha	0.05
Error Degre	432
Error Mear	0.076257
Critical Valu	1.96547
Least Signif	0.064

Means with the same letter are
not significantly different.

t Grouping		Mean	N	Site
	A	1.14569	144	Piketon
	A			
B	A	1.10229	144	Alabama
B				
B	C	1.06014	144	Hoytvill
	C			

	C	1.00056	144	Indiana

First paper

The ANOVA Procedure

First paper

The ANOVA Procedure

t Tests (LSD) for CWCCLI

Note: This test controls the Type I comparisonwise error rate, not the experimentwise error rate.

Alpha	0.05
Error Degre	432
Error Mear	0.206929
Critical Valu	1.96547
Least Signif	0.1054

Means with the same letter are not significantly different.

t Grouping		Mean	N	Site
	A	1.16153	144	Alabama
	A			
	A	1.12424	144	Hoytvill
	A			
B	A	1.06972	144	Indiana
B				
B		0.99431	144	Piketon

First paper

The ANOVA Procedure

First paper

The ANOVA Procedure

t Tests (LSD) for HWCCLI

Note: This test controls the Type I comparisonwise error rate, not the experimentwise error rate.

Alpha	0.05
Error Degre	432
Error Mear	0.186466
Critical Valı	1.96547
Least Signif	0.1

Means with the same letter are
not significantly different.

t Grouping		Mean	N	Site
	A	1.19236	144	Indiana
	A			
B	A	1.12361	144	Alabama
B				
B		1.06826	144	Hoytvill
	C	0.89708	144	Piketon

First paper

The ANOVA Procedure

First paper

The ANOVA Procedure

t Tests (LSD) for MBCCMI

Note: This test controls the Type I comparisonwise error rate, not the experimentwise error rate.

Alpha	0.05
Error Degr	432
Error Mear	0.136996
Critical Valı	1.96547
Least Signi	0.0857

Means with the same letter are not significantly different.

t Grouping	Mean	N	Site
A	1.31629	144	Indiana
B	1.12251	144	Hoytvill
C	1.00304	144	Alabama
D	0.79352	144	Piketon

First paper

The ANOVA Procedure

First paper

The ANOVA Procedure

t Tests (LSD) for ACCMI

Note: This test controls the Type I comparisonwise error rate, not the experimentwise error rate.

Alpha	0.05
Error Degre	432
Error Mear	0.048154
Critical Valı	1.96547
Least Signif	0.0508

Means with the same letter are
not significantly different.

t Grouping	Mean	N	Site
A	1.12916	144	Piketon
A			
A	1.09882	144	Hoytvill
B	1.03213	144	Alabama
C	0.95231	144	Indiana

First paper

The ANOVA Procedure

First paper

The ANOVA Procedure

t Tests (LSD) for CWCCMI

Note: This test controls the Type I comparisonwise error rate, not the experimentwise error rate.

Alpha	0.05
Error Degre	432
Error Mear	0.093987
Critical Valı	1.96547
Least Signif	0.071

Means with the same letter are
not significantly different.

t Grouping		Mean	N	Site
	A	1.15717	144	Hoytvill
	B	1.0059	144	Alabama
	B			
C	B	0.96842	144	Indiana
C				
C		0.90688	144	Piketon

First paper

The ANOVA Procedure

First paper

The ANOVA Procedure

t Tests (LSD) for HWCCMI

Note: This test controls the Type I comparisonwise error rate, not the experimentwise error rate.

Alpha	0.05
Error Degre	432
Error Mear	0.059791
Critical Valu	1.96547
Least Signif	0.0566

Means with the same letter are not significantly different.

t Grouping	Mean	N	Site
A	1.10573	144	Hoytvill
A			
A	1.07705	144	Indiana
B	0.94537	144	Alabama
C	0.83019	144	Piketon

First paper

The ANOVA Procedure

First paper

The ANOVA Procedure

t Tests (LSD) for SMB

Note: This test controls the Type I comparisonwise error rate, not the experimentwise error rate.

Alpha	0.05
Error Degre	432
Error Mear	4040.468
Critical Valu	1.96547
Least Signif	12.751

Means with the same letter
are not significantly different.

t Grouping	Mean	N	Gypsum
A	206.6	192	2000
B	173.299	192	1000
C	150.273	192	0

First paper

The ANOVA Procedure

First paper

The ANOVA Procedure

t Tests (LSD) for qR

Note: This test controls the Type I comparisonwise error rate, not the experimentwise error rate.

Alpha	0.05
Error Degre	432
Error Mear	1.225648
Critical Valu	1.96547
Least Signif	0.2221

Means with the same letter
are not significantly different.

t Grouping	Mean	N	Gypsum
A	2.2703	192	2000
A			
A	2.1516	192	1000
B	1.8396	192	0

First paper

The ANOVA Procedure

First paper

The ANOVA Procedure

t Tests (LSD) for SOC

Note: This test controls the Type I comparisonwise error rate, not the experimentwise error rate.

Alpha	0.05
Error Degre	432
Error Mear	0.044531
Critical Valu	1.96547
Least Signif	0.0423

Means with the same letter
are not significantly different.

t Grouping	Mean	N	Gypsum
A	1.0887	192	2000
A			
A	1.08266	192	1000
A			
A	1.06745	192	0

First paper

The ANOVA Procedure

First paper

The ANOVA Procedure

t Tests (LSD) for TN

Note: This test controls the Type I comparisonwise error rate, not the experimentwise error rate.

Alpha	0.05
Error Degre	432
Error Mear	0.000389
Critical Valu	1.96547
Least Signif	0.004

Means with the same letter

are not significantly different.

t Grouping		Mean	N	Gypsum
	A	0.123635	192	2000
	A			
B	A	0.11974	192	1000
B				
B		0.119396	192	0

First paper

The ANOVA Procedure

First paper

The ANOVA Procedure

t Tests (LSD) for AC

Note: This test controls the Type I comparisonwise error rate, not the experimentwise error rate.

Alpha	0.05
Error Degre	432
Error Mear	3793.294
Critical Valı	1.96547
Least Signif	12.355

Means with the same letter
are not significantly different.

t Grouping	Mean	N	Gypsum
A	401.417	192	2000
B	377.676	192	1000
B			
B	365.337	192	0

First paper

The ANOVA Procedure

First paper

The ANOVA Procedure

t Tests (LSD) for CWC

Note: This test controls the Type I comparisonwise error rate, not the experimentwise error rate.

Alpha	0.05
Error Degre	432
Error Mear	35.33734
Critical Valu	1.96547
Least Signif	1.1925

Means with the same letter
are not significantly different.

t Grouping		Mean	N	Gypsum
	A	19.2214	192	2000
	A			
B	A	18.6708	192	1000
B				
B		17.6505	192	0

First paper

The ANOVA Procedure

First paper

The ANOVA Procedure

t Tests (LSD) for HWC

Note: This test controls the Type I comparisonwise error rate, not the experimentwise error rate.

Alpha	0.05
Error Degre	432
Error Mear	167.2565
Critical Valı	1.96547
Least Signif	2.5943

Means with the same letter
are not significantly different.

t Grouping	Mean	N	Gypsum
A	53.53	192	2000
B	48.58	192	1000
C	44.516	192	0

First paper

The ANOVA Procedure

First paper

The ANOVA Procedure

t Tests (LSD) for CPI

Note: This test controls the Type I comparisonwise error rate, not the experimentwise error rate.

Alpha	0.05
Error Degre	432
Error Mear	0.061818
Critical Valu	1.96547
Least Signif	0.0499

Means with the same letter
are not significantly different.

t Grouping	Mean	N	Gypsum
A	1.02229	192	2000
A			
A	1.00479	192	0
A			
A	1.00396	192	1000

First paper

The ANOVA Procedure

First paper

The ANOVA Procedure

t Tests (LSD) for NPI

Note: This test controls the Type I comparisonwise error rate, not the experimentwise error rate.

Alpha	0.05
Error Degre	432
Error Mear	0.044534
Critical Valu	1.96547
Least Signif	0.0423

Means with the same letter
are not significantly different.

t Grouping	Mean	N	Gypsum
A	1.03958	192	2000

B	0.98625	192	0
B			
B	0.9726	192	1000

First paper

The ANOVA Procedure

First paper

The ANOVA Procedure

t Tests (LSD) for MBCCLI

Note: This test controls the Type I comparisonwise error rate, not the experimentwise error rate.

Alpha	0.05
Error Degr	432
Error Mear	0.342066
Critical Valu	1.96547
Least Signif	0.1173

Means with the same letter are not significantly different.

t Grouping	Mean	N	Gypsum
A	1.32266	192	2000
B	1.16635	192	1000
C	0.96865	192	0

First paper

The ANOVA Procedure

First paper

The ANOVA Procedure

t Tests (LSD) for ACCLI

Note: This test controls the Type I comparisonwise error rate, not the experimentwise error rate.

Alpha	0.05
Error Degre	432
Error Mear	0.076257
Critical Valu	1.96547
Least Signif	0.0554

Means with the same letter
are not significantly different.

t Grouping	Mean	N	Gypsum
A	1.13979	192	2000
B	1.06656	192	1000
B			
B	1.02516	192	0

First paper

The ANOVA Procedure

First paper

The ANOVA Procedure

t Tests (LSD) for CWCCLI

Note: This test controls the Type I comparisonwise error rate, not the experimentwise error rate.

Alpha	0.05
Error Degre	432
Error Mear	0.206929
Critical Valu	1.96547
Least Signif	0.0913

Means with the same letter
are not significantly different.

t Grouping		Mean	N	Gypsum
	A	1.14396	192	1000
	A			
B	A	1.10354	192	2000
B				
B		1.01484	192	0

First paper

The ANOVA Procedure

First paper

The ANOVA Procedure

t Tests (LSD) for HWCCLI

Note: This test controls the Type I comparisonwise error rate, not the experimentwise error rate.

Alpha	0.05
Error Degre	432
Error Mear	0.186466

Critical Valı	1.96547		
Least Signif	0.0866		

Means with the same letter
are not significantly different.

t Grouping	Mean	N	Gypsum
A	1.15391	192	2000
A			
A	1.09432	192	1000
B	0.96276	192	0

First paper

The ANOVA Procedure

First paper

The ANOVA Procedure

t Tests (LSD) for MBCCMI

Note: This test controls the Type I comparisonwise error rate, not the experimentwise error rate.

Alpha	0.05
Error Degre	432
Error Mear	0.136996
Critical Valı	1.96547
Least Signif	0.0742

Means with the same letter
are not significantly different.

t Grouping	Mean	N	Gypsum
A	1.25111	192	2000
B	1.04271	192	1000

C	0.88269	192	0

First paper

The ANOVA Procedure

First paper

The ANOVA Procedure

t Tests (LSD) for ACCMI

Note: This test controls the Type I comparisonwise error rate, not the experimentwise error rate.

Alpha	0.05
Error Degre	432
Error Mear	0.048154
Critical Valu	1.96547
Least Signif	0.044

Means with the same letter
are not significantly different.

t Grouping	Mean	N	Gypsum
A	1.12692	192	2000
B	1.03148	192	1000
B			
B	1.00092	192	0

First paper

The ANOVA Procedure

First paper

The ANOVA Procedure

t Tests (LSD) for CWCCMI

Note: This test controls the Type I comparisonwise error rate, not the experimentwise error rate.

Alpha	0.05
Error Degre	432
Error Mear	0.093987
Critical Valu	1.96547
Least Signif	0.0615

Means with the same letter
are not significantly different.

t Grouping	Mean	N	Gypsum
A	1.04457	192	1000
A			
A	1.03182	192	2000
B	0.95239	192	0

First paper

The ANOVA Procedure

First paper

The ANOVA Procedure

t Tests (LSD) for HWCCMI

Note:	This test controls the Type I comparisonwise error rate, not the experimentwise error rate.

Alpha	0.05
Error Degre	432
Error Mear	0.059791
Critical Valı	1.96547
Least Signif	0.0491

Means with the same letter
are not significantly different.

t Grouping	Mean	N	Gypsum
A	1.08999	192	2000
B	0.99083	192	1000
C	0.88793	192	0

First paper

The ANOVA Procedure

First paper

The ANOVA Procedure

t Tests (LSD) for SMB

Note:	This test controls the Type I comparisonwise error rate, not the experimentwise error rate.

Alpha	0.05
Error Degre	432
Error Mear	4040.468
Critical Valı	1.96547
Least Signif	12.751

Means with the same letter
are not significantly different.

t Grouping	Mean	N	CR
A	183.544	192	SS
A			
A	174.656	192	SC
A			
A	171.972	192	CS

First paper

The ANOVA Procedure

First paper

The ANOVA Procedure

t Tests (LSD) for qR

Note: This test controls the Type I comparisonwise error rate, not the experimentwise error rate.

Alpha	0.05
Error Degre	432
Error Mear	1.225648
Critical Valu	1.96547
Least Signif	0.2221

Means with the same letter
are not significantly different.

t Grouping	Mean	N	CR
A	2.5359	192	SS
B	2.0073	192	SC
C	1.7182	192	CS

First paper

The ANOVA Procedure

First paper

The ANOVA Procedure

t Tests (LSD) for SOC

Note: This test controls the Type I comparisonwise error rate, not the experimentwise error rate.

Alpha	0.05
Error Degre	432
Error Mear	0.044531
Critical Valı	1.96547
Least Signif	0.0423

Means with the same letter
are not significantly different.

t Grouping	Mean	N	CR
A	1.17531	192	CS
B	1.05719	192	SC
C	1.0063	192	SS

First paper

The ANOVA Procedure

First paper

The ANOVA Procedure

t Tests (LSD) for TN

Note: This test controls the Type I comparisonwise error rate, not the experimentwise error rate.

Alpha	0.05
Error Degre	432
Error Mear	0.000389
Critical Valu	1.96547
Least Signif	0.004

Means with the same letter
are not significantly different.

t Grouping	Mean	N	CR
A	0.126938	192	CS
B	0.11799	192	SS
B			
B	0.117844	192	SC

First paper

The ANOVA Procedure

First paper

The ANOVA Procedure

t Tests (LSD) for AC

Note: This test controls the Type I comparisonwise error rate, not the experimentwise error rate.

Alpha	0.05
Error Degre	432
Error Mear	3793.294
Critical Valu	1.96547
Least Signif	12.355

Means with the same letter
are not significantly different.

t Grouping	Mean	N	CR
A	418.687	192	CS
B	377.988	192	SC
C	347.755	192	SS

First paper

The ANOVA Procedure

First paper

The ANOVA Procedure

t Tests (LSD) for CWC

Note: This test controls the Type I comparisonwise error rate, not the experimentwise error rate.

Alpha	0.05
Error Degre	432
Error Mear	35.33734
Critical Valu	1.96547
Least Signif	1.1925

Means with the same letter
are not significantly different.

t Grouping	Mean	N	CR

A	18.7849	192	SS
A			
A	18.7578	192	CS
A			
A	18	192	SC

First paper

The ANOVA Procedure

First paper

The ANOVA Procedure

t Tests (LSD) for HWC

Note: This test controls the Type I comparisonwise error rate, not the experimentwise error rate.

Alpha	0.05
Error Degre	432
Error Mear	167.2565
Critical Valı	1.96547
Least Signif	2.5943

Means with the same letter
are not significantly different.

t Grouping	Mean	N	CR
A	49.368	192	SS
A			
A	48.845	192	CS
A			
A	48.413	192	SC

First paper

The ANOVA Procedure

First paper

The ANOVA Procedure

t Tests (LSD) for CPI

Note: This test controls the Type I comparisonwise error rate, not the experimentwise error rate.

Alpha	0.05
Error Degre	432
Error Mear	0.061818
Critical Valu	1.96547
Least Signif	0.0499

Means with the same letter are not significantly different.

t Grouping	Mean	N	CR
A	1.12417	192	CS
B	1.0063	192	SC
C	0.90057	192	SS

First paper

The ANOVA Procedure

First paper

The ANOVA Procedure

t Tests (LSD) for NPI

Note: This test controls the Type I comparisonwise error rate, not the experimentwise error rate.

Alpha	0.05
Error Degre	432
Error Mear	0.044534
Critical Valu	1.96547
Least Signif	0.0423

Means with the same letter
are not significantly different.

t Grouping	Mean	N	CR
A	1.03891	192	CS
B	0.98005	192	SC
B			
B	0.97948	192	SS

First paper

The ANOVA Procedure

First paper

The ANOVA Procedure

t Tests (LSD) for MBCCLI

Note: This test controls the Type I comparisonwise error rate, not the experimentwise error rate.

Alpha	0.05
Error Degre	432

Error Mear	0.342066
Critical Valı	1.96547
Least Signif	0.1173

Means with the same letter
are not significantly different.

t Grouping	Mean	N	CR
A	1.41714	192	SS
B	1.0788	192	SC
B			
B	0.96172	192	CS

First paper

The ANOVA Procedure

First paper

The ANOVA Procedure

t Tests (LSD) for ACCLI

Note: This test controls the Type I comparisonwise error rate, not the experimentwise error rate.

Alpha	0.05
Error Degre	432
Error Mear	0.076257
Critical Valı	1.96547
Least Signif	0.0554

Means with the same letter
are not significantly different.

t Grouping	Mean	N	CR
A	1.0888	192	SC
A			
A	1.08307	192	CS

A			
A	1.05964	192	SS

First paper

The ANOVA Procedure

First paper

The ANOVA Procedure

t Tests (LSD) for CWCCLI

Note:	This test controls the Type I comparisonwise error rate, not the experimentwise error rate.

Alpha	0.05
Error Degre	432
Error Mear	0.206929
Critical Valu	1.96547
Least Signif	0.0913

Means with the same letter
are not significantly different.

t Grouping	Mean	N	CR
A	1.26646	192	SS
B	1.03521	192	SC
B			
B	0.96068	192	CS

First paper

The ANOVA Procedure

First paper

The ANOVA Procedure

t Tests (LSD) for HWCCLI

Note: This test controls the Type I comparisonwise error rate, not the experimentwise error rate.

Alpha	0.05
Error Degre	432
Error Mear	0.186466
Critical Valu	1.96547
Least Signif	0.0866

Means with the same letter
are not significantly different.

t Grouping	Mean	N	CR
A	1.27979	192	SS
B	1.01005	192	SC
C	0.92115	192	CS

First paper

The ANOVA Procedure

First paper

The ANOVA Procedure

t Tests (LSD) for MBCCMI

Note:	This test controls the Type I comparisonwise error rate, not the experimentwise error rate.

Alpha	0.05
Error Degre	432
Error Mear	0.136996
Critical Valu	1.96547
Least Signif	0.0742

Means with the same letter
are not significantly different.

t Grouping	Mean	N	CR
A	1.13303	192	SS
B	1.02626	192	CS
B			
B	1.01722	192	SC

First paper

The ANOVA Procedure

First paper

The ANOVA Procedure

t Tests (LSD) for ACCMI

Note:	This test controls the Type I comparisonwise error rate, not the experimentwise error rate.

Alpha	0.05
Error Degre	432
Error Mear	0.048154
Critical Valu	1.96547
Least Signif	0.044

Means with the same letter
are not significantly different.

t Grouping	Mean	N	CR
A	1.18581	192	CS
B	1.06644	192	SC
C	0.90708	192	SS

First paper

The ANOVA Procedure

First paper

The ANOVA Procedure

t Tests (LSD) for CWCCMI

Note: This test controls the Type I comparisonwise error rate, not the experimentwise error rate.

Alpha	0.05
Error Degre	432
Error Mear	0.093987
Critical Valu	1.96547
Least Signif	0.0615

Means with the same letter
are not significantly different.

t Grouping	Mean	N	CR
A	1.02676	192	CS
A			
A	1.01908	192	SS
A			
A	0.98294	192	SC

First paper

The ANOVA Procedure

First paper

The ANOVA Procedure

t Tests (LSD) for HWCCMI

Note: This test controls the Type I comparisonwise error rate, not the experimentwise error rate.

Alpha	0.05
Error Degre	432
Error Mear	0.059791
Critical Valu	1.96547
Least Signif	0.0491

Means with the same letter
are not significantly different.

t Grouping		Mean	N	CR
	A	1.02454	192	SS
	A			
B	A	0.98752	192	CS
B				
B		0.9567	192	SC

First paper

The ANOVA Procedure

First paper

The ANOVA Procedure

t Tests (LSD) for SMB

Note: This test controls the Type I comparisonwise error rate, not the experimentwise error rate.

Alpha	0.05
Error Degre	432
Error Mear	4040.468
Critical Valu	1.96547
Least Signif	10.411

Means with the same letter
are not significantly different.

t Grouping	Mean	N	CC
A	180.964	288	No
A			
A	172.484	288	Yes

First paper

The ANOVA Procedure

First paper

The ANOVA Procedure

t Tests (LSD) for qR

Note: This test controls the Type I comparisonwise error rate, not the experimentwise error rate.

Alpha	0.05
Error Degre	432
Error Mear	1.225648
Critical Valu	1.96547
Least Signif	0.1813

Means with the same letter
are not significantly different.

t Grouping	Mean	N	CC
A	2.1184	288	No
A			
A	2.0559	288	Yes

First paper

The ANOVA Procedure

First paper

The ANOVA Procedure

t Tests (LSD) for SOC

Note: This test controls the Type I comparisonwise error rate, not the experimentwise error rate.

Alpha	0.05
Error Degre	432
Error Mear	0.044531
Critical Valu	1.96547
Least Signif	0.0346

Means with the same letter
are not significantly different.

t Grouping	Mean	N	CC
A	1.08413	288	Yes
A			
A	1.07507	288	No

First paper

The ANOVA Procedure

First paper

The ANOVA Procedure

t Tests (LSD) for TN

Note: This test controls the Type I comparisonwise error rate, not the experimentwise error rate.

Alpha	0.05
Error Degre	432
Error Mear	0.000389
Critical Valu	1.96547
Least Signif	0.0032

Means with the same letter
are not significantly different.

t Grouping	Mean	N	CC
A	0.121729	288	Yes
A			
A	0.120118	288	No

First paper

The ANOVA Procedure

First paper

The ANOVA Procedure

t Tests (LSD) for AC

Note: This test controls the Type I comparisonwise error rate, not the experimentwise error rate.

Alpha	0.05
Error Degre	432
Error Mear	3793.294
Critical Valu	1.96547
Least Signif	10.088

Means with the same letter
are not significantly different.

t Grouping	Mean	N	CC
A	389.845	288	Yes
B	373.108	288	No

First paper

The ANOVA Procedure

First paper

The ANOVA Procedure

t Tests (LSD) for CWC

Note: This test controls the Type I comparisonwise error rate, not the experimentwise error rate.

Alpha	0.05

Error Degr	432
Error Mear	35.33734
Critical Val	1.96547
Least Signi	0.9736

Means with the same letter
are not significantly different.

t Grouping	Mean	N	CC
A	18.8198	288	Yes
A			
A	18.2087	288	No

First paper

The ANOVA Procedure

First paper

The ANOVA Procedure

t Tests (LSD) for HWC

Note: This test controls the Type I comparisonwise error rate, not the experimentwise error rate.

Alpha	0.05
Error Degr	432
Error Mear	167.2565
Critical Val	1.96547
Least Signi	2.1182

Means with the same letter
are not significantly different.

t Grouping	Mean	N	CC
A	49.999	288	No
B	47.752	288	Yes

First paper

The ANOVA Procedure

First paper

The ANOVA Procedure

t Tests (LSD) for CPI

Note:	This test controls the Type I comparisonwise error rate, not the experimentwise error rate.

Alpha	0.05
Error Degre	432
Error Mear	0.061818
Critical Valu	1.96547
Least Signif	0.0407

Means with the same letter
are not significantly different.

t Grouping	Mean	N	CC
A	1.01288	288	Yes
A			
A	1.00781	288	No

First paper

The ANOVA Procedure

First paper

The ANOVA Procedure

t Tests (LSD) for NPI

Note: This test controls the Type I comparisonwise error rate, not the experimentwise error rate.

Alpha	0.05
Error Degre	432
Error Mear	0.044534
Critical Valu	1.96547
Least Signif	0.0346

Means with the same letter
are not significantly different.

t Grouping	Mean	N	CC
A	1.01597	288	Yes
A			
A	0.98299	288	No

First paper

The ANOVA Procedure

First paper

The ANOVA Procedure

t Tests (LSD) for MBCCLI

Note: This test controls the Type I comparisonwise error rate, not the experimentwise error rate.

Alpha	0.05
Error Degre	432

Error Mear	0.342066
Critical Valı	1.96547
Least Signif	0.0958

Means with the same letter
are not significantly different.

t Grouping	Mean	N	CC
A	1.16885	288	No
A			
A	1.13625	288	Yes

First paper

The ANOVA Procedure

First paper

The ANOVA Procedure

t Tests (LSD) for ACCLI

Note: This test controls the Type I comparisonwise error rate, not the experimentwise error rate.

Alpha	0.05
Error Degre	432
Error Mear	0.076257
Critical Valı	1.96547
Least Signif	0.0452

Means with the same letter
are not significantly different.

t Grouping	Mean	N	CC
A	1.10313	288	Yes
B	1.05122	288	No

First paper

The ANOVA Procedure

First paper

The ANOVA Procedure

t Tests (LSD) for CWCCLI

Note: This test controls the Type I comparisonwise error rate, not the experimentwise error rate.

Alpha	0.05
Error Degre	432
Error Mear	0.206929
Critical Valu	1.96547
Least Signif	0.0745

Means with the same letter
are not significantly different.

t Grouping	Mean	N	CC
A	1.11253	288	Yes
A			
A	1.06236	288	No

First paper

The ANOVA Procedure

First paper

The ANOVA Procedure

t Tests (LSD) for HWCCLI

Note:	This test controls the Type I comparisonwise error rate, not the experimentwise error rate.

Alpha	0.05
Error Degr€	432
Error Mear	0.186466
Critical Valı	1.96547
Least Signil	0.0707

Means with the same letter
are not significantly different.

t Grouping	Mean	N	CC
A	1.08042	288	No
A			
A	1.06024	288	Yes

First paper

The ANOVA Procedure

First paper

The ANOVA Procedure

t Tests (LSD) for MBCCMI

Note:	This test controls the Type I comparisonwise error rate, not the experimentwise error rate.

Alpha	0.05
Error Degr€	432
Error Mear	0.136996

Critical Valu	1.96547
Least Signif	0.0606

Means with the same letter
are not significantly different.

t Grouping	Mean	N	CC
A	1.08142	288	No
A			
A	1.03625	288	Yes

First paper

The ANOVA Procedure

First paper

The ANOVA Procedure

t Tests (LSD) for ACCMI

Note: This test controls the Type I comparisonwise error rate, not the experimentwise error rate.

Alpha	0.05
Error Degre	432
Error Mean	0.048154
Critical Valu	1.96547
Least Signif	0.0359

Means with the same letter
are not significantly different.

t Grouping	Mean	N	CC
A	1.07466	288	Yes
B	1.03156	288	No

First paper

The ANOVA Procedure

First paper

The ANOVA Procedure

t Tests (LSD) for CWCCMI

Note: This test controls the Type I comparisonwise error rate, not the experimentwise error rate.

Alpha	0.05
Error Degre	432
Error Mear	0.093987
Critical Valu	1.96547
Least Signif	0.0502

Means with the same letter
are not significantly different.

t Grouping	Mean	N	CC
A	1.02123	288	Yes
A			
A	0.99796	288	No

First paper

The ANOVA Procedure

First paper

The ANOVA Procedure

t Tests (LSD) for HWCCMI

Note: This test controls the Type I comparisonwise error rate, not the experimentwise error rate.

Alpha	0.05
Error Degre	432
Error Mear	0.059791
Critical Valı	1.96547
Least Signif	0.0401

Means with the same letter
are not significantly different.

t Grouping	Mean	N	CC
A	1.00821	288	No
A			
A	0.97096	288	Yes

First paper

The ANOVA Procedure

First paper

The ANOVA Procedure

t Tests (LSD) for SMB

Note: This test controls the Type I comparisonwise error rate, not the experimentwise error rate.

Alpha	0.05
Error Degre	432
Error Mear	4040.468
Critical Valı	1.96547

Least Signif	10.411		

Means with the same letter
are not significantly different.

t Grouping	Mean	N	Depth
A	218.953	288	0
B	134.494	288	15

First paper

The ANOVA Procedure

First paper

The ANOVA Procedure

t Tests (LSD) for qR

Note: This test controls the Type I comparisonwise error rate, not the experimentwise error rate.

Alpha	0.05
Error Degre	432
Error Mear	1.225648
Critical Valu	1.96547
Least Signif	0.1813

Means with the same letter
are not significantly different.

t Grouping	Mean	N	Depth
A	2.20521	288	15
B	1.9691	288	0

First paper

The ANOVA Procedure

First paper

The ANOVA Procedure

t Tests (LSD) for SOC

Note:	This test controls the Type I comparisonwise error rate, not the experimentwise error rate.

Alpha	0.05
Error Degre	432
Error Mear	0.044531
Critical Valu	1.96547
Least Signif	0.0346

Means with the same letter
are not significantly different.

t Grouping	Mean	N	Depth
A	1.24816	288	0
B	0.91104	288	15

First paper

The ANOVA Procedure

First paper

The ANOVA Procedure

t Tests (LSD) for TN

Note:	This test controls the Type I comparisonwise error rate, not the experimentwise error rate.

Alpha	0.05
Error Degre	432
Error Mear	0.000389
Critical Valı	1.96547
Least Signif	0.0032

Means with the same letter
are not significantly different.

t Grouping	Mean	N	Depth
A	0.135698	288	0
B	0.106149	288	15

First paper

The ANOVA Procedure

First paper

The ANOVA Procedure

t Tests (LSD) for AC

Note:	This test controls the Type I comparisonwise error rate, not the experimentwise error rate.

Alpha	0.05
Error Degre	432
Error Mear	3793.294
Critical Valı	1.96547
Least Signif	10.088

Means with the same letter
are not significantly different.

t Grouping	Mean	N	Depth
A	442.236	288	0
B	320.717	288	15

First paper

The ANOVA Procedure

First paper

The ANOVA Procedure

t Tests (LSD) for CWC

Note: This test controls the Type I comparisonwise error rate, not the experimentwise error rate.

Alpha	0.05
Error Degre	432
Error Mear	35.33734
Critical Valı	1.96547
Least Signif	0.9736

Means with the same letter
are not significantly different.

t Grouping	Mean	N	Depth
A	21.8951	288	0
B	15.1333	288	15

First paper

The ANOVA Procedure

First paper

The ANOVA Procedure

t Tests (LSD) for HWC

Note: This test controls the Type I comparisonwise error rate, not the experimentwise error rate.

Alpha	0.05
Error Degre	432
Error Mear	167.2565
Critical Valu	1.96547
Least Signif	2.1182

Means with the same letter
are not significantly different.

t Grouping	Mean	N	Depth
A	59.633	288	0
B	38.118	288	15

First paper

The ANOVA Procedure

First paper

The ANOVA Procedure

t Tests (LSD) for CPI

Note: This test controls the Type I comparisonwise error rate, not the experimentwise error rate.

Alpha	0.05
Error Degre	432
Error Mear	0.061818
Critical Valu	1.96547
Least Signif	0.0407

Means with the same letter
are not significantly different.

t Grouping	Mean	N	Depth
A	1.03094	288	0
B	0.98976	288	15

First paper

The ANOVA Procedure

First paper

The ANOVA Procedure

t Tests (LSD) for NPI

Note: This test controls the Type I comparisonwise error rate, not the experimentwise error rate.

Alpha	0.05
Error Degre	432
Error Mear	0.044534
Critical Valu	1.96547
Least Signif	0.0346

Means with the same letter
are not significantly different.

t Grouping	Mean	N	Depth
A	1.04302	288	0
B	0.95594	288	15

First paper

The ANOVA Procedure

First paper

The ANOVA Procedure

t Tests (LSD) for MBCCLI

Note: This test controls the Type I comparisonwise error rate, not the experimentwise error rate.

Alpha	0.05
Error Degre	432
Error Mear	0.342066
Critical Valı	1.96547
Least Signif	0.0958

Means with the same letter
are not significantly different.

t Grouping	Mean	N	Depth
A	1.19122	288	15
A			
A	1.11389	288	0

First paper

The ANOVA Procedure

First paper

The ANOVA Procedure

t Tests (LSD) for ACCLI

Note: This test controls the Type I comparisonwise error rate, not the experimentwise error rate.

Alpha	0.05
Error Degre	432
Error Mear	0.076257
Critical Valu	1.96547
Least Signif	0.0452

Means with the same letter
are not significantly different.

t Grouping	Mean	N	Depth
A	1.14372	288	15
B	1.01063	288	0

First paper

The ANOVA Procedure

First paper

The ANOVA Procedure

t Tests (LSD) for CWCCLI

Note: This test controls the Type I comparisonwise error rate, not the experimentwise error rate.

Alpha	0.05
Error Degre	432
Error Mear	0.206929
Critical Valu	1.96547
Least Signif	0.0745

Means with the same letter
are not significantly different.

t Grouping	Mean	N	Depth
A	1.16128	288	15
B	1.01361	288	0

First paper

The ANOVA Procedure

First paper

The ANOVA Procedure

t Tests (LSD) for HWCCLI

Note: This test controls the Type I comparisonwise error rate, not the experimentwise error rate.

Alpha	0.05
Error Degre	432
Error Mear	0.186466
Critical Valu	1.96547
Least Signif	0.0707

Means with the same letter

are not significantly different.

t Grouping	Mean	N	Depth
A	1.11372	288	15
B	1.02694	288	0

First paper

The ANOVA Procedure

First paper

The ANOVA Procedure

t Tests (LSD) for MBCCMI

Note: This test controls the Type I comparisonwise error rate, not the experimentwise error rate.

Alpha	0.05
Error Degre	432
Error Mear	0.136996
Critical Valı	1.96547
Least Signif	0.0606

Means with the same letter
are not significantly different.

t Grouping	Mean	N	Depth
A	1.09859	288	0
B	1.01909	288	15

First paper

The ANOVA Procedure

First paper

The ANOVA Procedure

t Tests (LSD) for ACCMI

Note: This test controls the Type I comparisonwise error rate, not the experimentwise error rate.

Alpha	0.05
Error Degre	432
Error Mear	0.048154
Critical Valu	1.96547
Least Signif	0.0359

Means with the same letter
are not significantly different.

t Grouping	Mean	N	Depth
A	1.08291	288	15
B	1.02331	288	0

First paper

The ANOVA Procedure

First paper

The ANOVA Procedure

t Tests (LSD) for CWCCMI

Note: This test controls the Type I comparisonwise error rate, not the experimentwise error rate.

Alpha	0.05
Error Degre	432
Error Mear	0.093987
Critical Valı	1.96547
Least Signif	0.0502

Means with the same letter
are not significantly different.

t Grouping	Mean	N	Depth
A	1.01535	288	15
A			
A	1.00383	288	0

First paper

The ANOVA Procedure

First paper

The ANOVA Procedure

t Tests (LSD) for HWCCMI

Note: This test controls the Type I comparisonwise error rate, not the experimentwise error rate.

Alpha	0.05
Error Degre	432
Error Mear	0.059791
Critical Valı	1.96547
Least Signif	0.0401

Means with the same letter
are not significantly different.

t Grouping	Mean	N	Depth
A	1.01529	288	0
B	0.96388	288	15

The ANOVA Procedure

Level of Gypsum	Level of CR	N	SMB Mean	Std Dev	qR Mean	Std Dev	SOC Mean	Std Dev
0	CS	64	131.5125	79.26178	1.3375	0.982788	1.2	0.50
0	SC	64	142.5188	100.0267	1.717188	1.394155	1.0	0.50
0	SS	64	176.7875	92.12106	2.464063	1.820201	1.0	0.55
1000	CS	64	173.4016	73.88058	1.748438	1.04608	1.2	0.55
1000	SC	64	175.2266	95.21127	2.132813	1.777554	1.0	0.48
1000	SS	64	171.2688	87.19487	2.573438	2.070522	1.0	0.62
2000	CS	64	211.0031	89.92727	2.06875	0.891739	1.2	0.51
2000	SC	64	206.2219	110.9689	2.171875	1.199003	1.1	0.54
2000	SS	64	202.575	113.2835	2.570313	1.582411	1.0	0.58

Level of Gypsum	Level of CC	N	SMB Mean	Std Dev	qR Mean	Std Dev	SOC Mean	Std Dev
0	No	96	158.551	101.6497	1.954167	1.687472	1.1	0.52
0	Yes	96	141.9948	81.90959	1.725	1.301982	1.1	0.52
1000	No	96	177.1292	85.02307	2.069792	1.336383	1.1	0.56
1000	Yes	96	169.4688	86.13977	2.233333	2.023424	1.1	0.55
2000	No	96	207.2115	97.56295	2.33125	1.345035	1.1	0.54
2000	Yes	96	205.9885	112.0013	2.209375	1.191291	1.1	0.55

Level of Gypsum	Level of Depth	N	SMB Mean	Std Dev	qR Mean	Std Dev	SOC Mean	Std Dev
0	0	96	187.0896	106.6842	1.758333	1.260799	1.2	0.41
0	15	96	113.4563	55.31154	1.920833	1.722294	0.9	0.56
1000	0	96	213.1354	94.32201	1.866667	0.991826	1.3	0.43
1000	15	96	133.4625	50.74144	2.436458	2.178513	0.9	0.60
2000	0	96	256.6354	106.4556	2.282292	1.157856	1.3	0.43
2000	15	96	156.5646	75.30587	2.258333	1.376545	0.9	0.59

Level of CR	Level of CC	N	SMB Mean	Std Dev	qR Mean	Std Dev	SOC Mean	Std Dev

CS	No	96	177.7208	84.47954	1.7625	0.956556	1.2	0.52
CS	Yes	96	166.224	89.86544	1.673958	1.075003	1.2	0.51
SC	No	96	179.4333	108.5765	2.045833	1.396606	1.1	0.50
SC	Yes	96	169.8781	101.7116	1.96875	1.571544	1.1	0.51
SS	No	96	185.7375	96.62878	2.546875	1.825835	1.0	0.58
SS	Yes	96	181.35	101.0422	2.525	1.836301	1.0	0.58

Level of CR	Level of Depth	N	SMB Mean	Std Dev	qR Mean	Std Dev	SOC Mean	Std Dev
CS	0	96	214.0625	89.46563	1.6875	0.863926	1.4	0.38
CS	15	96	129.8823	60.78506	1.748958	1.151657	1.0	0.56
SC	0	96	219.0458	117.3496	2.059375	1.387734	1.2	0.40
SC	15	96	130.2656	66.53458	1.955208	1.578598	0.9	0.55
SS	0	96	223.7521	110.8312	2.160417	1.127408	1.2	0.46
SS	15	96	143.3354	63.28797	2.911458	2.269332	0.8	0.64

Level of CC	Level of Depth	N	SMB Mean	Std Dev	qR Mean	Std Dev	SOC Mean	Std Dev
No	0	144	225.3826	103.7079	2.071528	1.240216	1.2	0.44
No	15	144	136.5451	63.80378	2.165278	1.66961	0.9	0.58
Yes	0	144	212.5243	108.7165	1.866667	1.069409	1.3	0.41
Yes	15	144	132.4438	63.68511	2.245139	1.919274	0.9	0.59

Level of Gypsum	Level of CR	Level of CC	N	SMB Mean	Std Dev	qR Mean	Std Dev	SOC Mean
0	CS	No	32	136.1219	89.67954	1.4	1.1	1.15
0	CS	Yes	32	126.9031	68.41956	1.275	0.9	1.19
0	SC	No	32	147.8313	112.8966	1.659375	1.5	1.09
0	SC	Yes	32	137.2063	86.7729	1.775	1.3	0.99
0	SS	No	32	191.7	95.4167	2.803125	2.1	0.95
0	SS	Yes	32	161.875	87.652	2.125	1.5	1.03
1000	CS	No	32	190.2594	70.3972	1.80625	0.7	1.22
1000	CS	Yes	32	156.5438	74.50023	1.690625	1.3	1.18
1000	SC	No	32	180.1969	95.64765	2.190625	1.3	1.01
1000	SC	Yes	32	170.2563	96.03813	2.075	2.1	1.07
1000	SS	No	32	160.9313	87.12288	2.2125	1.7	1.06
1000	SS	Yes	32	181.6063	87.40783	2.934375	2.3	0.96
2000	CS	No	32	206.7813	77.79651	2.08125	1.0	1.14
2000	CS	Yes	32	215.225	101.7133	2.05625	0.8	1.17
2000	SC	No	32	210.2719	110.5433	2.2875	1.3	1.06
2000	SC	Yes	32	202.1719	113.0121	2.05625	1.0	1.12
2000	SS	No	32	204.5813	104.31	2.625	1.6	1.00

2000	SS	Yes	32	200.5688	123.2534	2.515625	1.6	1.03

Level of Gypsum	Level of CR	Level of Depth	N	SMB Mean	Std Dev	qR Mean	Std Dev	SOC Mean
0	CS	0	32	166.4219	89.28409	1.365625	1.0	1.36
0	CS	15	32	96.60313	47.74849	1.309375	1.0	0.99
0	SC	0	32	179.8188	122.0406	1.84375	1.6	1.17
0	SC	15	32	105.2188	50.66746	1.590625	1.2	0.91
0	SS	0	32	215.0281	103.4988	2.065625	1.1	1.15
0	SS	15	32	138.5469	59.28985	2.8625	2.3	0.83
1000	CS	0	32	212.8719	72.5289	1.609375	0.7	1.40
1000	CS	15	32	133.9313	51.14679	1.8875	1.3	0.99
1000	SC	0	32	223.0625	105.1446	2.046875	1.2	1.21
1000	SC	15	32	127.3906	51.41226	2.21875	2.2	0.87
1000	SS	0	32	203.4719	103.6816	1.94375	1.0	1.19
1000	SS	15	32	139.0656	50.59927	3.203125	2.6	0.84
2000	CS	0	32	262.8938	80.56603	2.0875	0.8	1.35
2000	CS	15	32	159.1125	66.21795	2.05	1.0	0.96
2000	SC	0	32	254.2563	115.6162	2.2875	1.4	1.24
2000	SC	15	32	158.1875	83.0352	2.05625	1.0	0.95
2000	SS	0	32	252.7563	121.6953	2.471875	1.3	1.17
2000	SS	15	32	152.3938	77.92144	2.66875	1.9	0.86

Level of Gypsum	Level of CC	Level of Depth	N	SMB Mean	Std Dev	qR Mean	Std Dev	SOC Mean
0	No	0	48	202.0833	116.7538	1.927083	1.4	1.22
0	No	15	48	115.0188	58.16329	1.98125	2.0	0.91
0	Yes	0	48	172.0958	94.41682	1.589583	1.1	1.23
0	Yes	15	48	111.8938	52.876	1.860417	1.5	0.91
1000	No	0	48	219.0458	91.64135	1.9125	1.0	1.28
1000	No	15	48	135.2125	51.23216	2.227083	1.6	0.91
1000	Yes	0	48	207.225	97.53503	1.820833	1.0	1.26
1000	Yes	15	48	131.7125	50.72652	2.645833	2.6	0.88
2000	No	0	48	255.0188	95.92531	2.375	1.3	1.21
2000	No	15	48	159.4042	73.27744	2.2875	1.4	0.93
2000	Yes	0	48	258.2521	117.0458	2.189583	1.0	1.29
2000	Yes	15	48	153.725	77.95225	2.229167	1.3	0.93

Level of CR	Level of CC	Level of Depth	N	SMB Mean	Std Dev	qR Mean	Std Dev	SOC Mean
CS	No	0	48	222.9771	80.37051	1.789583	0.9	1.35

CS	No	15	48	132.4646	61.50285	1.735417	1.1	0.99
CS	Yes	0	48	205.1479	97.75868	1.585417	0.9	1.39
CS	Yes	15	48	127.3	60.59736	1.7625	1.3	0.97
SC	No	0	48	227.3104	122.4171	2.222917	1.6	1.19
SC	No	15	48	131.5563	64.50277	1.86875	1.2	0.92
SC	Yes	0	48	210.7813	112.7359	1.895833	1.2	1.22
SC	Yes	15	48	128.975	69.16559	2.041667	1.9	0.90
SS	No	0	48	225.8604	106.1524	2.202083	1.2	1.16
SS	No	15	48	145.6146	65.69902	2.891667	2.3	0.84
SS	Yes	0	48	221.6438	116.4089	2.11875	1.1	1.17
SS	Yes	15	48	141.0563	61.39205	2.93125	2.3	0.85

Level of Site	Level of Gypsum	Level of CR	Level of CC	Level of Depth	N	SMB Mean	Std Dev	qR Mean
Alabama	0	CS	No	0	4	107.1	21.0	1.20
Alabama	0	CS	No	15	4	41.825	23.2	0.75
Alabama	0	CS	Yes	0	4	88.875	26.8	0.90
Alabama	0	CS	Yes	15	4	71.55	27.8	1.20
Alabama	0	SC	No	0	4	128.8	35.1	1.55
Alabama	0	SC	No	15	4	67.475	29.0	1.35
Alabama	0	SC	Yes	0	4	128.4	18.9	1.70
Alabama	0	SC	Yes	15	4	83.725	28.5	2.50
Alabama	0	SS	No	0	4	153.325	40.7	2.43
Alabama	0	SS	No	15	4	122.875	36.7	4.65
Alabama	0	SS	Yes	0	4	144.3	35.2	2.08
Alabama	0	SS	Yes	15	4	102.625	30.6	4.30
Alabama	1000	CS	No	0	4	167.725	43.0	1.80
Alabama	1000	CS	No	15	4	112.2	51.1	2.45
Alabama	1000	CS	Yes	0	4	135.35	9.6	1.33
Alabama	1000	CS	Yes	15	4	88.4	62.8	2.48
Alabama	1000	SC	No	0	4	141.125	43.3	2.20
Alabama	1000	SC	No	15	4	95.375	37.3	1.93
Alabama	1000	SC	Yes	0	4	134.875	36.6	1.48
Alabama	1000	SC	Yes	15	4	66.175	14.1	1.33
Alabama	1000	SS	No	0	4	148.125	38.1	2.20
Alabama	1000	SS	No	15	4	91.9	14.4	3.15
Alabama	1000	SS	Yes	0	4	145.45	44.3	2.38
Alabama	1000	SS	Yes	15	4	103.075	43.2	6.80
Alabama	2000	CS	No	0	4	196.35	30.3	1.98
Alabama	2000	CS	No	15	4	123.65	29.6	2.70
Alabama	2000	CS	Yes	0	4	207.125	22.2	2.00
Alabama	2000	CS	Yes	15	4	125.3	23.4	2.25
Alabama	2000	SC	No	0	4	163.75	72.8	2.28
Alabama	2000	SC	No	15	4	75.425	29.7	1.73

Alabama	2000	SC	Yes	0	4	155.15	32.4	1.78
Alabama	2000	SC	Yes	15	4	86.325	23.8	1.93
Alabama	2000	SS	No	0	4	151.225	40.3	3.10
Alabama	2000	SS	No	15	4	104.275	35.2	3.40
Alabama	2000	SS	Yes	0	4	157	37.6	2.83
Alabama	2000	SS	Yes	15	4	105.3	32.9	4.00
Hoytvill	0	CS	No	0	4	145.025	58.2	0.88
Hoytvill	0	CS	No	15	4	118.825	65.1	0.73
Hoytvill	0	CS	Yes	0	4	140.35	55.0	0.85
Hoytvill	0	CS	Yes	15	4	150.675	50.8	0.88
Hoytvill	0	SC	No	0	4	152.4	45.8	0.88
Hoytvill	0	SC	No	15	4	135.65	81.3	0.80
Hoytvill	0	SC	Yes	0	4	121.4	36.0	0.70
Hoytvill	0	SC	Yes	15	4	126.25	49.0	0.75
Hoytvill	0	SS	No	0	4	201.45	107.6	1.20
Hoytvill	0	SS	No	15	4	171.675	53.4	1.03
Hoytvill	0	SS	Yes	0	4	164.3	65.3	1.00
Hoytvill	0	SS	Yes	15	4	163.175	72.8	0.95
Hoytvill	1000	CS	No	0	4	261.65	28.3	1.40
Hoytvill	1000	CS	No	15	4	213.275	37.0	1.15
Hoytvill	1000	CS	Yes	0	4	134.275	35.3	0.80
Hoytvill	1000	CS	Yes	15	4	103.975	28.0	0.58
Hoytvill	1000	SC	No	0	4	163.525	31.8	0.98
Hoytvill	1000	SC	No	15	4	122.025	46.9	0.80
Hoytvill	1000	SC	Yes	0	4	194.025	93.6	1.15
Hoytvill	1000	SC	Yes	15	4	106.85	50.4	0.65
Hoytvill	1000	SS	No	0	4	176.85	72.4	0.95
Hoytvill	1000	SS	No	15	4	140.75	26.1	0.78
Hoytvill	1000	SS	Yes	0	4	146.425	39.4	0.85
Hoytvill	1000	SS	Yes	15	4	176.975	22.2	1.03
Hoytvill	2000	CS	No	0	4	259.075	49.7	1.55
Hoytvill	2000	CS	No	15	4	200.25	81.6	1.18
Hoytvill	2000	CS	Yes	0	4	187.05	88.6	1.13
Hoytvill	2000	CS	Yes	15	4	136.15	62.5	0.83
Hoytvill	2000	SC	No	0	4	223.25	78.4	1.25
Hoytvill	2000	SC	No	15	4	223.45	62.3	1.28
Hoytvill	2000	SC	Yes	0	4	153.525	61.6	0.88
Hoytvill	2000	SC	Yes	15	4	162.875	27.5	0.88
Hoytvill	2000	SS	No	0	4	226.35	84.7	1.33
Hoytvill	2000	SS	No	15	4	186.025	60.9	1.03
Hoytvill	2000	SS	Yes	0	4	168.7	24.0	0.95
Hoytvill	2000	SS	Yes	15	4	133.65	29.3	0.75
Indiana	0	CS	No	0	4	138.475	17.9	0.83
Indiana	0	CS	No	15	4	86.55	20.0	0.83
Indiana	0	CS	Yes	0	4	119.275	26.4	0.65
Indiana	0	CS	Yes	15	4	59.325	31.2	0.50
Indiana	0	SC	No	0	4	120.25	23.9	0.88

Indiana	0	SC	No	15	4	75.85	28.8	0.63
Indiana	0	SC	Yes	0	4	134.45	25.3	1.13
Indiana	0	SC	Yes	15	4	92.075	22.9	1.13
Indiana	0	SS	No	0	4	310.1	69.1	2.50
Indiana	0	SS	No	15	4	144.7	51.9	1.78
Indiana	0	SS	Yes	0	4	280.9	150.9	2.05
Indiana	0	SS	Yes	15	4	158.25	100.2	1.90
Indiana	1000	CS	No	0	4	259.55	42.0	1.68
Indiana	1000	CS	No	15	4	143.225	9.9	1.20
Indiana	1000	CS	Yes	0	4	209.7	40.4	1.33
Indiana	1000	CS	Yes	15	4	175.525	35.9	1.73
Indiana	1000	SC	No	0	4	233.975	62.4	1.93
Indiana	1000	SC	No	15	4	182.825	65.3	3.13
Indiana	1000	SC	Yes	0	4	221.1	58.8	1.58
Indiana	1000	SC	Yes	15	4	152.85	45.8	1.43
Indiana	1000	SS	No	0	4	280.875	170.3	2.03
Indiana	1000	SS	No	15	4	130.3	83.4	1.33
Indiana	1000	SS	Yes	0	4	346.3	49.6	2.55
Indiana	1000	SS	Yes	15	4	195.35	33.0	2.15
Indiana	2000	CS	No	0	4	269.925	50.1	1.80
Indiana	2000	CS	No	15	4	208.575	49.2	2.10
Indiana	2000	CS	Yes	0	4	391.45	39.2	2.25
Indiana	2000	CS	Yes	15	4	256.375	42.4	2.20
Indiana	2000	SC	No	0	4	294.45	80.7	2.18
Indiana	2000	SC	No	15	4	183.45	65.2	1.78
Indiana	2000	SC	Yes	0	4	392.75	12.5	2.83
Indiana	2000	SC	Yes	15	4	299.725	70.3	3.05
Indiana	2000	SS	No	0	4	344.875	115.8	2.70
Indiana	2000	SS	No	15	4	238.175	120.6	3.08
Indiana	2000	SS	Yes	0	4	450.3	125.0	3.35
Indiana	2000	SS	Yes	15	4	222.875	94.7	2.60
Piketon	0	CS	No	0	4	330.225	79.5	3.18
Piketon	0	CS	No	15	4	120.95	11.6	2.83
Piketon	0	CS	Yes	0	4	262.05	35.4	2.45
Piketon	0	CS	Yes	15	4	123.125	28.5	2.78
Piketon	0	SC	No	0	4	358.575	195.1	4.43
Piketon	0	SC	No	15	4	143.65	70.6	2.78
Piketon	0	SC	Yes	0	4	294.275	171.9	3.50
Piketon	0	SC	Yes	15	4	117.075	40.6	2.80
Piketon	0	SS	No	0	4	279.275	144.0	3.20
Piketon	0	SS	No	15	4	150.2	73.0	5.65
Piketon	0	SS	Yes	0	4	186.575	61.7	2.08
Piketon	0	SS	Yes	15	4	94.875	14.3	2.65
Piketon	1000	CS	No	0	4	249.2	68.2	2.08
Piketon	1000	CS	No	15	4	115.25	16.7	2.70
Piketon	1000	CS	Yes	0	4	285.525	92.0	2.48
Piketon	1000	CS	Yes	15	4	119.6	22.7	2.83

Piketon	1000	SC	No	0	4	369.675	91.7	3.75
Piketon	1000	SC	No	15	4	133.05	16.3	2.83
Piketon	1000	SC	Yes	0	4	326.2	131.6	3.33
Piketon	1000	SC	Yes	15	4	159.975	31.2	5.68
Piketon	1000	SS	No	0	4	176.275	53.8	1.98
Piketon	1000	SS	No	15	4	142.375	57.7	5.30
Piketon	1000	SS	Yes	0	4	207.475	128.2	2.63
Piketon	1000	SS	Yes	15	4	131.8	28.4	5.10
Piketon	2000	CS	No	0	4	291.425	65.3	3.13
Piketon	2000	CS	No	15	4	105	26.0	2.23
Piketon	2000	CS	Yes	0	4	300.75	64.9	2.88
Piketon	2000	CS	Yes	15	4	117.6	13.5	2.93
Piketon	2000	SC	No	0	4	377.95	130.0	4.40
Piketon	2000	SC	No	15	4	140.45	20.5	3.43
Piketon	2000	SC	Yes	0	4	273.225	104.1	2.73
Piketon	2000	SC	Yes	15	4	93.8	17.6	2.40
Piketon	2000	SS	No	0	4	261.6	96.4	2.83
Piketon	2000	SS	No	15	4	124.125	49.0	3.55
Piketon	2000	SS	Yes	0	4	262	63.5	2.70
Piketon	2000	SS	Yes	15	4	104.725	37.1	2.95

SOC (%)	TN (%)	AC (mg/kg)	CWC (mg/kg)	HWC (mg/kg)	CPI	NPI	MBCCLI	ACCLI
1.30	0.139	503.4	26.0	66.8	0.89	0.87	2.95	1.19
0.67	0.089	229.9	22.0	35.1	0.61	0.68	1.81	1.05
1.38	0.152	522.2	33.8	63.9	0.94	0.95	2.02	1.16
0.71	0.083	196.6	28.0	42.7	0.65	0.64	1.91	0.83
1.06	0.125	451.3	45.5	58.8	0.73	0.78	1.15	1.30
1.27	0.079	232.8	25.6	32.9	1.15	0.61	0.53	0.55
1.04	0.140	381.8	36.8	64.3	0.71	0.88	2.47	1.13
0.47	0.074	144.5	18.1	23.8	0.43	0.57	1.10	0.93
1.31	0.142	502.0	29.1	62.5	0.89	0.89	2.38	1.18
0.66	0.088	270.4	19.6	33.9	0.60	0.68	2.00	1.25
1.47	0.155	545.4	43.0	84.5	1.00	0.97	2.64	1.14
0.75	0.095	280.5	36.9	56.5	0.69	0.73	2.41	1.14
1.38	0.148	516.4	34.7	70.0	0.95	0.93	2.37	1.14
0.64	0.089	280.5	29.6	43.4	0.58	0.68	2.01	1.35
1.21	0.140	420.9	19.8	64.0	0.83	0.88	3.44	1.06
0.62	0.090	247.3	19.6	54.9	0.56	0.69	5.50	1.22
1.23	0.137	494.7	53.1	76.9	0.84	0.86	1.79	1.24
0.61	0.090	276.2	31.0	42.8	0.55	0.69	1.79	1.39
1.29	0.150	475.9	53.2	93.0	0.88	0.94	2.90	1.13
0.65	0.084	276.2	36.5	49.4	0.59	0.65	1.83	1.30
1.20	0.133	483.1	57.2	91.0	0.82	0.83	2.64	1.24
0.59	0.085	242.9	26.9	39.6	0.54	0.65	1.99	1.26
1.12	0.127	400.7	19.4	33.4	0.77	0.79	1.15	1.09
0.73	0.095	260.3	16.1	21.6	0.66	0.73	0.69	1.09
1.33	0.143	484.6	27.3	53.1	0.91	0.89	1.79	1.11
1.33	0.144	490.4	15.3	35.0	1.21	1.11	1.36	1.13
1.08	0.126	341.3	27.7	52.2	0.74	0.79	2.10	0.96
1.00	0.112	331.2	19.8	36.3	0.91	0.86	1.52	1.01
1.25	0.139	468.7	39.8	58.9	0.86	0.87	1.41	1.15
1.03	0.121	370.3	17.9	32.6	0.94	0.93	1.30	1.09
1.38	0.154	425.3	24.5	51.4	0.95	0.96	1.80	0.93
1.10	0.128	329.7	14.3	32.7	1.00	0.98	1.54	0.91
1.25	0.138	458.5	29.0	54.6	0.86	0.86	1.89	1.12
0.71	0.092	261.7	23.5	45.5	0.64	0.71	2.91	1.13
1.44	0.152	504.9	39.0	64.6	0.99	0.95	1.64	1.07
1.12	0.124	355.8	30.7	45.4	1.02	0.95	1.20	0.97
1.95	0.191	585.9	38.1	63.1	1.34	1.19	1.17	0.91
1.43	0.148	455.6	25.3	42.9	1.30	1.14	1.13	0.97
1.85	0.187	535.2	34.2	49.5	1.26	1.17	0.76	0.88
1.72	0.168	491.8	19.9	25.0	1.56	1.29	0.27	0.87
1.38	0.150	504.9	24.8	77.3	0.95	0.94	3.58	1.11
0.86	0.090	248.7	20.0	49.8	0.78	0.69	3.24	0.87

1.33	0.146	455.6	42.8	52.7	0.91	0.91	0.67	1.04
1.15	0.129	370.3	28.9	34.3	1.05	0.99	0.43	0.98
1.83	0.183	506.3	40.4	71.5	1.25	1.14	1.57	0.84
1.58	0.163	438.3	23.6	43.9	1.44	1.25	1.18	0.84
1.62	0.168	407.9	40.8	68.3	1.11	1.05	1.56	0.76
1.49	0.156	383.3	27.6	48.3	1.36	1.20	1.27	0.78
1.06	0.124	407.9	39.9	54.8	0.73	0.78	1.28	1.17
0.53	0.077	148.8	30.0	38.9	0.48	0.59	1.56	0.85
1.12	0.130	350.0	32.9	45.8	0.77	0.81	1.06	0.95
0.67	0.089	190.8	21.9	29.2	0.61	0.68	1.01	0.86
1.34	0.149	399.2	49.0	62.3	0.92	0.93	0.91	0.90
0.86	0.112	254.5	41.3	50.4	0.78	0.86	0.96	0.89
1.08	0.130	364.5	38.6	48.5	0.74	0.81	0.84	1.03
0.85	0.100	231.3	26.8	32.9	0.77	0.77	0.65	0.82
1.17	0.133	413.7	35.8	44.0	0.80	0.83	0.64	1.08
1.02	0.089	198.0	31.6	34.7	0.92	0.68	0.28	0.59
1.23	0.136	431.0	44.7	53.4	0.84	0.85	0.64	1.07
0.73	0.092	219.8	33.0	41.1	0.66	0.71	1.01	0.91
1.29	0.143	415.1	44.7	63.0	0.88	0.89	1.31	0.98
1.03	0.094	219.8	26.5	42.7	0.94	0.72	1.44	0.64
1.30	0.157	383.3	36.2	69.5	0.89	0.98	2.38	0.89
0.87	0.104	295.0	31.5	51.7	0.79	0.80	2.16	1.04
1.17	0.132	378.9	46.2	62.7	0.80	0.83	1.30	0.99
0.70	0.093	238.6	34.2	42.1	0.64	0.72	1.02	1.04
1.35	0.149	451.3	44.3	77.3	0.93	0.93	2.27	1.01
0.96	0.113	299.4	22.7	54.1	0.87	0.87	3.08	0.95
1.81	0.184	519.3	42.5	74.7	1.24	1.15	1.64	0.87
1.38	0.145	420.9	24.5	44.6	1.25	1.12	1.34	0.93
1.71	0.190	590.2	34.3	50.2	1.17	1.19	0.85	1.05
1.37	0.170	400.7	30.8	44.0	1.25	1.31	0.88	0.89
1.46	0.156	439.7	24.9	43.9	1.00	0.98	1.19	0.92
1.18	0.133	326.8	14.9	28.6	1.07	1.02	1.07	0.84
1.61	0.172	517.9	28.3	53.6	1.10	1.08	1.45	0.98
1.38	0.154	358.7	17.2	36.3	1.26	1.18	1.27	0.78
1.69	0.179	542.5	28.8	38.6	1.16	1.12	0.52	0.98
1.59	0.169	429.6	15.4	23.3	1.45	1.30	0.45	0.82
1.77	0.184	538.1	26.3	51.4	1.21	1.15	1.30	0.92
1.45	0.162	383.3	21.9	36.8	1.31	1.25	0.94	0.80
1.46	0.184	506.3	22.4	35.4	1.00	1.15	0.81	1.05
1.19	0.125	328.3	13.5	22.8	1.08	0.96	0.71	0.84
1.68	0.167	536.7	33.9	47.0	1.15	1.04	0.71	0.97
1.52	0.152	392.0	17.1	25.8	1.38	1.17	0.52	0.78
1.35	0.209	436.8	28.1	53.4	0.93	1.31	1.73	0.98
1.22	0.134	339.9	16.7	32.1	1.11	1.03	1.16	0.84
0.85	0.100	224.1	21.5	32.4	0.58	0.63	1.18	0.80
0.56	0.070	328.3	16.4	21.4	0.51	0.54	0.82	1.83
1.28	0.141	373.2	21.3	55.2	0.88	0.88	2.47	0.88

0.95	0.104	300.8	15.5	36.1	0.87	0.80	2.00	0.96
1.11	0.124	464.3	18.0	43.1	0.76	0.78	2.09	1.28
0.43	0.064	172.0	9.4	30.5	0.39	0.49	4.66	1.21
1.16	0.126	420.9	14.0	37.9	0.79	0.79	1.91	1.11
0.61	0.078	224.1	10.8	29.9	0.56	0.60	2.93	1.12
1.27	0.132	425.3	15.4	50.0	0.87	0.83	2.55	1.02
0.85	0.095	287.8	9.7	39.8	0.77	0.73	3.35	1.03
1.15	0.129	457.1	23.9	50.4	0.78	0.81	2.15	1.22
0.97	0.133	227.0	22.9	35.2	0.88	1.02	1.16	0.70
1.28	0.143	464.3	28.0	49.4	0.88	0.89	1.54	1.11
0.77	0.141	300.8	26.5	44.3	0.70	1.08	2.17	1.20
1.72	0.180	519.3	36.1	55.1	1.18	1.13	1.01	0.91
0.94	0.112	332.6	32.5	47.3	0.86	0.86	1.44	1.08
1.48	0.159	519.3	39.1	63.6	1.01	0.99	1.53	1.07
1.34	0.147	407.9	19.0	40.2	1.22	1.13	1.45	0.92
1.78	0.185	570.0	40.1	53.0	1.22	1.16	0.66	0.97
1.25	0.138	387.6	35.7	44.7	1.14	1.06	0.66	0.94
1.89	0.195	538.1	38.0	48.7	1.30	1.22	0.51	0.86
1.34	0.197	378.9	33.0	41.7	1.21	1.52	0.59	0.86
1.94	0.201	574.3	39.7	53.6	1.33	1.26	0.65	0.90
1.51	0.159	461.4	31.9	39.1	1.37	1.22	0.43	0.93
1.98	0.204	648.1	47.9	65.8	1.35	1.28	0.82	1.00
1.41	0.153	431.0	21.5	37.9	1.28	1.18	1.06	0.93
1.27	0.142	509.2	15.6	26.3	0.87	0.89	0.76	1.23
0.67	0.089	282.0	13.5	22.0	0.60	0.68	1.18	1.30
1.43	0.154	509.2	19.0	26.8	0.98	0.96	0.49	1.08
0.69	0.093	279.1	14.7	18.3	0.63	0.72	0.48	1.23
1.68	0.177	522.2	17.7	26.8	1.15	1.11	0.49	0.94
1.40	0.153	418.0	13.8	17.9	1.27	1.18	0.27	0.90
1.81	0.187	551.2	13.3	25.2	1.24	1.17	0.60	0.93
1.43	0.154	410.8	10.4	15.4	1.30	1.18	0.32	0.87
1.78	0.183	584.4	33.5	46.7	1.22	1.14	0.67	1.00
1.21	0.133	350.0	26.6	37.4	1.10	1.02	0.82	0.88
1.69	0.178	513.5	46.2	70.4	1.16	1.11	1.32	0.92
1.26	0.138	367.4	25.2	41.7	1.15	1.06	1.20	0.88
1.79	0.185	536.7	52.9	69.6	1.22	1.16	0.86	0.91
1.55	0.165	435.4	33.3	45.7	1.41	1.27	0.73	0.85
2.05	0.211	571.4	19.7	30.7	1.40	1.32	0.49	0.84
1.44	0.151	399.2	20.8	24.2	1.30	1.16	0.22	0.84
1.25	0.140	519.3	37.5	63.9	0.85	0.88	1.97	1.28
0.60	0.087	255.9	22.4	43.5	0.54	0.67	3.33	1.32
1.28	0.144	436.8	34.3	65.2	0.88	0.90	2.23	1.04
0.84	0.102	296.5	24.5	44.1	0.76	0.78	2.17	1.08
1.68	0.225	541.0	40.9	70.9	1.15	1.41	1.65	0.98
1.28	0.137	426.7	27.1	50.1	1.16	1.05	1.66	1.02
1.78	0.184	545.4	22.6	44.3	1.22	1.15	1.11	0.93
1.53	0.154	444.1	19.9	32.7	1.39	1.18	0.77	0.88

1.94	0.195	548.3	38.0	74.2	1.33	1.22	1.72	0.85
1.26	0.133	412.2	29.6	55.9	1.14	1.02	1.94	1.00
1.72	0.174	477.4	44.2	66.8	1.18	1.09	1.20	0.84
1.06	0.123	396.3	37.5	50.2	0.96	0.95	1.09	1.14
1.94	0.197	606.2	20.2	38.0	1.33	1.23	0.84	0.95
1.39	0.147	445.5	15.8	26.8	1.26	1.13	0.72	0.98
2.27	0.230	610.5	35.9	72.1	1.55	1.44	1.47	0.81
1.41	0.150	397.8	28.8	47.2	1.28	1.15	1.19	0.85
0.64	0.093	226.5	17.1	45.7	0.80	1.55	1.07	1.13
0.25	0.068	133.0	8.4	24.8	0.56	1.69	1.56	1.70
0.83	0.098	258.9	12.5	51.5	1.04	1.63	1.13	0.99
0.31	0.064	123.4	9.9	47.3	0.69	1.60	3.00	1.28
0.71	0.081	207.4	21.1	51.8	0.89	1.35	1.04	0.93
0.31	0.051	119.6	9.4	22.3	0.69	1.28	1.00	1.24
0.61	0.062	146.3	15.5	38.7	0.77	1.03	0.91	0.75
0.39	0.044	131.1	15.3	29.4	0.86	1.10	0.86	1.08
0.85	0.075	182.6	14.0	33.8	1.06	1.24	0.56	0.68
0.53	0.045	112.0	6.0	15.1	1.18	1.14	0.65	0.67
0.97	0.075	163.5	31.0	55.3	1.22	1.25	0.59	0.53
0.65	0.046	136.8	10.4	23.5	1.45	1.14	0.48	0.66
0.50	0.136	184.5	16.2	45.4	0.63	2.27	1.40	1.18
0.29	0.047	131.1	9.4	32.1	0.64	1.18	1.92	1.47
0.83	0.079	276.1	14.9	50.5	1.03	1.32	1.03	1.07
0.16	0.040	136.8	6.0	28.5	0.36	0.99	3.49	2.86
0.69	0.067	310.4	16.6	57.0	0.86	1.12	1.41	1.46
0.16	0.033	104.4	5.9	40.9	0.35	0.81	5.75	2.17
0.61	0.062	264.6	9.8	34.8	0.76	1.04	0.98	1.40
0.32	0.040	169.2	7.0	33.5	0.71	1.00	2.03	1.73
0.55	0.052	190.2	8.5	36.8	0.68	0.86	1.24	1.11
0.40	0.036	125.4	6.2	26.8	0.88	0.90	1.25	1.01
1.10	0.077	237.9	16.6	55.7	1.37	1.28	0.85	0.68
0.58	0.038	92.9	6.3	24.9	1.29	0.96	0.77	0.50
0.73	0.121	165.4	14.2	49.9	0.92	2.02	1.17	0.71
0.28	0.038	98.6	7.0	30.9	0.61	0.96	2.70	1.14
0.68	0.063	176.9	11.5	49.3	0.85	1.05	1.34	0.82
0.11	0.031	113.9	9.2	26.4	0.25	0.77	3.88	3.49
0.41	0.087	115.8	20.4	60.1	0.51	1.46	2.40	0.90
0.18	0.086	96.7	13.5	43.5	0.40	2.15	4.25	1.76
0.63	0.056	169.2	14.2	54.9	0.79	0.93	1.56	0.85
0.20	0.035	92.9	7.0	32.8	0.45	0.88	3.16	1.47
0.57	0.043	106.3	10.4	39.1	0.72	0.72	1.20	0.58
0.28	0.033	87.2	8.6	28.3	0.63	0.82	1.69	0.98
0.75	0.058	159.7	10.7	32.2	0.94	0.96	0.68	0.67
0.20	0.032	68.1	11.0	30.5	0.46	0.80	2.34	1.06
0.50	0.051	161.6	11.1	33.7	0.63	0.84	1.07	1.02
0.21	0.035	83.4	7.8	28.7	0.47	0.88	2.41	1.25
0.34	0.037	140.6	16.6	61.1	0.43	0.61	3.25	1.32

0.32	0.029	96.7	8.9	38.7	0.71	0.73	2.29	0.96
0.47	0.042	155.9	16.0	53.0	0.59	0.71	1.92	1.06
0.31	0.035	115.8	11.0	24.9	0.68	0.87	1.88	1.21
0.43	0.046	96.7	8.0	30.5	0.54	0.76	1.25	0.71
0.13	0.028	49.0	8.0	22.4	0.28	0.70	4.34	1.24
0.51	0.039	133.0	8.3	35.7	0.64	0.65	1.51	0.82
0.23	0.030	49.0	4.2	22.8	0.50	0.74	2.22	0.69
0.68	0.104	136.8	7.1	47.7	0.85	1.73	1.45	0.64
0.31	0.031	96.7	10.0	25.0	0.70	0.77	1.86	0.98
0.45	0.041	203.6	12.2	51.3	0.56	0.68	2.14	1.47
0.39	0.040	194.0	13.6	43.0	0.87	1.00	1.82	1.60
0.68	0.067	243.6	10.1	35.7	0.85	1.11	0.90	1.15
0.51	0.033	192.1	8.7	23.6	1.13	0.84	0.70	1.21
0.72	0.063	222.7	12.4	30.7	0.90	1.05	0.60	0.98
0.58	0.050	197.9	9.9	27.2	1.30	1.26	0.70	1.08
0.96	0.073	300.9	9.7	43.9	1.20	1.22	0.85	1.00
0.77	0.044	176.9	9.1	25.3	1.71	1.10	0.50	0.73
0.80	0.066	243.6	8.0	34.4	1.00	1.11	0.79	0.97
0.28	0.035	152.1	8.0	23.0	0.63	0.87	1.91	1.75
1.13	0.097	237.9	7.1	31.7	1.41	1.62	0.52	0.66
0.33	0.058	175.0	4.1	22.4	0.74	1.45	1.52	1.71
0.61	0.184	247.5	15.3	46.0	0.76	3.07	1.22	1.31
0.23	0.073	173.1	10.0	23.0	0.51	1.83	1.36	2.50
0.57	0.064	234.1	9.2	30.1	0.71	1.06	1.15	1.33
0.28	0.034	163.5	8.0	24.7	0.62	0.84	2.11	1.94
0.70	0.056	199.8	26.4	44.7	0.88	0.93	0.62	0.90
0.42	0.037	155.9	6.4	23.4	0.93	0.93	0.97	1.19
0.78	0.034	230.3	13.1	43.1	0.98	0.57	0.92	0.93
0.31	0.028	182.6	10.9	26.8	0.69	0.70	1.24	1.94
1.03	0.072	251.3	15.1	51.7	1.29	1.20	0.85	0.77
0.59	0.066	245.6	12.1	24.3	1.32	1.65	0.49	1.33
0.93	0.051	169.2	20.1	38.9	1.16	0.84	0.48	0.57
0.85	0.044	165.4	17.0	32.8	1.89	1.10	0.44	0.61
0.89	0.070	255.1	13.2	36.2	1.11	1.16	0.62	0.91
0.48	0.041	175.0	9.1	13.4	1.06	1.02	0.57	1.17
0.96	0.063	218.8	11.1	46.4	1.21	1.05	0.87	0.72
0.45	0.031	152.1	12.9	23.6	0.99	0.78	0.57	1.09
0.84	0.107	243.6	8.4	42.5	1.05	1.78	1.05	0.92
0.79	0.049	176.9	5.7	23.8	1.76	1.23	0.46	0.71
0.88	0.074	251.3	12.3	41.4	1.10	1.23	0.79	0.91
0.46	0.037	171.1	1.2	23.7	1.02	0.93	1.18	1.20
0.92	0.058	215.0	8.1	43.9	1.15	0.97	0.93	0.74
0.49	0.039	157.8	8.0	24.0	1.09	0.98	1.14	1.03
1.19	0.071	251.3	12.0	28.5	1.49	1.18	0.53	0.67
0.62	0.041	142.5	10.0	24.3	1.38	1.04	0.93	0.73
0.77	0.056	211.2	7.1	20.2	0.96	0.94	0.58	0.87
0.51	0.032	152.1	4.1	9.3	1.13	0.81	0.37	0.95

0.60	0.042	199.8	8.3	63.7	0.76	0.71	2.25	1.05
0.39	0.029	199.8	8.1	9.6	0.88	0.72	0.47	1.65
0.63	0.038	222.7	11.0	44.0	0.79	0.63	1.37	1.13
0.38	0.032	157.8	8.1	12.9	0.84	0.80	0.74	1.34
0.95	0.070	224.6	12.6	39.7	1.18	1.17	0.68	0.75
0.44	0.041	175.0	7.7	20.2	0.98	1.03	0.67	1.28
0.89	0.063	220.8	14.0	34.1	1.12	1.05	0.88	0.78
0.54	0.039	182.6	8.0	13.8	1.21	0.97	0.60	1.07
1.17	0.068	234.1	7.4	47.6	1.46	1.13	0.82	0.63
0.92	0.046	207.4	4.1	25.5	2.05	1.16	0.62	0.71
0.85	0.060	354.3	18.0	64.7	1.07	1.00	1.32	1.34
0.46	0.037	152.1	17.5	39.0	1.02	0.93	1.13	1.06
0.70	0.048	381.0	17.9	41.2	0.88	0.79	0.80	1.77
0.53	0.029	129.2	17.3	23.3	1.18	0.72	0.27	0.77
0.71	0.041	381.0	13.3	50.1	0.89	0.68	1.24	1.75
0.53	0.038	241.7	9.0	36.2	1.18	0.95	1.62	1.48
0.81	0.052	316.2	15.0	28.5	1.01	0.87	0.80	1.26
0.25	0.033	102.5	10.1	47.6	0.56	0.83	3.76	1.31
0.86	0.056	119.6	10.0	26.3	1.07	0.93	0.55	0.44
0.50	0.038	110.1	6.5	11.3	1.10	0.95	0.49	0.70
1.12	0.121	445.9	8.1	44.6	1.40	2.02	0.91	1.28
0.48	0.039	241.7	6.5	29.2	1.07	0.98	1.46	1.63
0.87	0.054	428.7	11.0	49.4	1.09	0.90	1.36	1.60
0.66	0.038	321.9	4.0	32.4	1.46	0.96	1.15	1.59
1.05	0.063	281.8	12.7	27.6	1.31	1.05	0.47	0.86
0.77	0.039	197.9	6.9	32.2	1.70	0.98	0.60	0.82
1.08	0.067	442.1	18.9	47.2	1.35	1.12	0.62	1.32
0.74	0.039	272.3	8.6	27.6	1.63	0.98	0.61	1.19
1.09	0.067	459.3	8.3	41.9	1.36	1.11	0.80	1.36
0.27	0.035	66.2	5.0	26.6	0.60	0.87	2.35	0.77
0.97	0.071	281.8	10.0	26.7	1.21	1.18	0.41	0.92
0.58	0.040	169.2	9.2	18.5	1.29	0.99	0.64	0.92
0.91	0.057	321.9	18.1	43.2	1.13	0.95	0.66	1.14
0.32	0.036	159.7	12.1	38.6	0.71	0.90	2.02	1.61
1.07	0.072	257.0	20.6	31.6	1.34	1.20	0.24	0.76
0.67	0.038	194.0	13.5	23.5	1.50	0.95	0.35	0.92
1.05	0.057	203.6	17.1	48.9	1.31	0.95	0.72	0.61
0.72	0.047	163.5	12.2	23.6	1.60	1.17	0.37	0.72
1.17	0.085	449.7	9.3	47.5	1.46	1.42	0.78	1.24
0.65	0.066	266.5	6.0	27.2	1.44	1.65	0.98	1.32
1.08	0.070	60.5	7.0	29.5	1.34	1.16	0.52	0.17
0.54	0.040	14.7	5.7	5.3	1.21	1.00	0.20	0.08
1.29	0.087	283.7	9.0	50.9	1.61	1.45	0.93	0.69
0.49	0.037	215.0	8.5	30.0	1.08	0.93	1.06	1.43
0.95	0.062	264.6	8.2	40.5	1.19	1.04	0.81	0.88
0.27	0.036	157.8	5.0	11.1	0.60	0.90	0.95	1.92
0.93	0.062	224.6	20.7	48.1	1.16	1.04	0.70	0.76

0.56	0.040	205.5	13.9	23.3	1.24	1.00	0.40	1.18
1.00	0.058	432.5	17.4	61.3	1.25	0.96	1.05	1.40
0.92	0.054	220.8	16.0	38.7	2.05	1.36	0.58	0.76
1.05	0.066	251.3	10.5	49.2	1.31	1.10	0.88	0.76
0.99	0.047	169.2	5.0	37.3	2.19	1.19	0.89	0.54
1.17	0.076	272.3	25.0	53.3	1.46	1.26	0.57	0.73
0.35	0.034	176.9	8.0	7.7	0.78	0.86	0.50	1.63
1.09	0.072	237.9	8.2	32.6	1.36	1.20	0.53	0.69
0.52	0.038	161.6	7.2	28.3	1.15	0.96	0.97	0.99
1.04	0.068	430.6	12.4	58.9	1.30	1.14	1.07	1.33
0.45	0.039	152.1	9.4	29.2	1.00	0.98	1.06	1.08
1.02	0.113	549.7	21.3	72.0	1.11	1.03	0.61	1.04
0.72	0.096	271.0	16.0	39.4	1.68	1.38	0.40	0.71
0.93	0.103	328.3	16.8	47.1	1.01	0.94	0.40	0.67
0.39	0.062	120.6	14.1	34.3	0.90	0.88	0.64	0.58
1.18	0.125	341.3	13.6	58.0	1.28	1.13	0.46	0.54
0.34	0.058	120.6	12.5	41.3	0.80	0.83	1.06	0.66
1.06	0.120	525.1	16.2	72.2	1.16	1.10	0.65	0.94
0.35	0.056	315.3	16.1	39.5	0.81	0.80	0.84	1.81
1.26	0.128	292.1	23.0	68.3	1.37	1.16	0.44	0.43
0.61	0.077	182.1	13.3	30.0	1.42	1.10	0.33	0.56
1.01	0.109	418.0	18.9	68.3	1.10	0.99	0.60	0.78
0.69	0.081	322.5	14.1	31.9	1.60	1.15	0.31	0.89
1.20	0.122	293.6	12.9	32.3	1.30	1.11	0.20	0.46
0.52	0.066	166.2	9.8	31.7	1.20	0.94	0.52	0.60
1.14	0.122	293.6	15.2	40.2	1.23	1.10	0.27	0.48
0.52	0.067	203.8	13.3	37.2	1.21	0.95	0.56	0.74
0.98	0.111	416.6	17.2	40.1	1.06	1.01	0.29	0.81
0.26	0.047	164.8	13.0	38.8	0.60	0.68	1.26	1.24
0.89	0.102	502.0	20.8	62.8	0.96	0.93	0.58	1.09
0.43	0.065	284.9	12.5	31.6	1.01	0.93	0.54	1.28
0.90	0.104	271.9	21.1	111.1	0.98	0.95	1.27	0.57
0.23	0.044	105.4	20.0	67.3	0.53	0.62	2.76	0.88
0.80	0.095	431.0	18.4	86.7	0.87	0.86	1.07	1.04
0.30	0.052	169.1	11.8	46.8	0.70	0.75	1.48	1.08
0.84	0.098	429.6	14.2	64.1	0.91	0.89	0.74	0.98
0.29	0.051	185.0	10.8	58.8	0.68	0.72	2.12	1.22
1.03	0.119	574.3	21.8	96.0	1.12	1.08	0.89	1.07
0.40	0.061	306.6	20.0	50.7	0.92	0.88	0.97	1.52
0.74	0.096	483.1	19.2	86.4	0.80	0.87	1.15	1.27
0.24	0.047	160.4	15.8	46.3	0.57	0.67	1.60	1.28
1.15	0.210	543.9	18.6	104.8	1.25	1.91	0.94	0.90
0.29	0.051	258.8	9.6	49.9	0.67	0.73	1.81	1.80
0.79	0.095	240.0	15.0	90.3	0.86	0.87	1.19	0.57
0.27	0.106	157.5	9.5	52.6	0.63	1.51	2.07	1.12
0.75	0.095	247.3	16.2	71.2	0.82	0.86	0.91	0.62
0.37	0.034	251.6	11.0	32.2	0.86	0.49	0.71	1.33

0.75	0.092	363.0	17.4	35.6	0.81	0.83	0.30	0.93
0.22	0.046	127.1	10.4	31.3	0.52	0.66	1.19	1.10
0.65	0.083	522.2	13.6	58.9	0.71	0.75	0.87	1.59
0.31	0.053	247.3	13.1	41.7	0.73	0.75	1.15	1.56
0.85	0.100	271.9	15.9	69.2	0.93	0.91	0.78	0.60
0.22	0.043	70.7	12.9	28.6	0.50	0.62	0.91	0.62
0.70	0.088	232.8	15.4	49.0	0.76	0.80	0.59	0.62
0.20	0.043	79.4	11.9	31.0	0.47	0.62	1.19	0.74
0.75	0.094	564.2	11.5	53.8	0.82	0.85	0.70	1.48
0.15	0.039	150.3	11.5	23.1	0.36	0.55	0.94	1.96
0.68	0.086	458.5	11.0	36.6	0.73	0.78	0.46	1.33
0.16	0.042	56.2	11.3	35.6	0.37	0.60	1.97	0.66
0.88	0.105	474.5	17.4	74.6	0.96	0.96	0.81	1.04
0.34	0.055	209.6	15.5	31.9	0.78	0.78	0.60	1.21
0.95	0.105	487.5	21.6	80.2	1.03	0.96	0.77	0.99
0.39	0.036	368.8	11.5	35.4	0.91	0.52	0.76	1.90
0.93	0.110	481.7	18.7	84.9	1.01	1.00	0.89	0.99
0.61	0.081	342.8	17.6	49.9	1.42	1.16	0.65	1.08
1.22	0.129	462.9	20.6	79.6	1.33	1.17	0.60	0.72
0.37	0.056	360.1	19.9	44.4	0.85	0.79	0.83	1.98
0.99	0.117	486.0	21.5	125.3	1.08	1.06	1.32	0.93
0.41	0.057	363.0	15.2	42.2	0.94	0.82	0.83	1.79
1.04	0.118	460.0	22.4	134.5	1.14	1.08	1.36	0.84
0.44	0.062	367.4	13.3	46.1	1.03	0.89	0.92	1.65
0.81	0.097	451.3	14.8	122.8	0.88	0.88	1.71	1.07
0.56	0.075	348.6	9.4	44.7	1.30	1.07	0.78	1.21
0.87	0.103	457.1	12.8	37.0	0.95	0.94	0.34	1.01
0.51	0.064	348.6	9.4	21.6	1.19	0.91	0.29	1.33
0.86	0.101	465.8	16.0	53.7	0.93	0.91	0.54	1.05
0.49	0.064	355.8	10.1	49.7	1.14	0.92	1.01	1.43
0.98	0.108	449.9	16.3	80.0	1.06	0.98	0.81	0.88
0.44	0.060	374.6	14.2	48.6	1.02	0.85	0.98	1.69
0.91	0.106	455.6	17.6	132.0	0.99	0.97	1.60	0.96
0.36	0.054	216.9	11.6	36.5	0.84	0.78	0.85	1.16
1.00	0.114	477.4	12.8	118.2	1.09	1.03	1.33	0.91
0.15	0.038	40.3	11.2	51.3	0.35	0.55	3.62	0.50
1.13	0.124	445.5	19.1	119.1	1.23	1.13	1.11	0.75
0.41	0.063	352.9	14.2	42.2	0.96	0.90	0.84	1.69
1.00	0.113	444.1	22.0	107.4	1.09	1.02	1.07	0.84
0.50	0.069	360.1	9.5	27.0	1.17	0.99	0.42	1.40
1.00	0.112	447.0	14.8	87.0	1.09	1.02	0.90	0.85
0.47	0.064	345.7	12.2	41.0	1.09	0.91	0.76	1.45
0.65	0.083	432.5	23.4	117.9	0.71	0.76	1.88	1.30
0.48	0.066	338.4	12.6	40.1	1.12	0.94	0.71	1.38
0.72	0.090	460.0	17.1	46.3	0.79	0.81	0.50	1.24
0.48	0.067	367.4	10.0	23.5	1.13	0.95	0.34	1.49
0.88	0.106	432.5	21.3	61.9	0.96	0.96	0.57	0.94

0.45	0.062	339.9	13.7	45.1	1.04	0.88	0.87	1.49
1.02	0.113	458.5	15.6	78.2	1.11	1.02	0.76	0.86
0.36	0.056	335.5	13.1	41.0	0.83	0.81	0.97	1.87
0.90	0.106	464.3	19.1	70.8	0.98	0.96	0.71	0.99
0.31	0.052	221.2	12.2	37.2	0.72	0.74	1.01	1.39
0.68	0.082	471.6	22.6	76.4	0.73	0.74	1.00	1.36
0.64	0.080	315.3	16.6	65.4	1.48	1.14	0.96	0.95
0.68	0.085	455.6	22.3	104.3	0.74	0.77	1.53	1.30
0.37	0.054	293.6	14.1	43.2	0.86	0.78	0.98	1.56
0.90	0.106	538.1	12.8	43.9	0.97	0.97	0.42	1.16
0.39	0.057	352.9	12.5	34.0	0.91	0.82	0.68	1.80
0.90	0.098	444.1	17.0	107.2	0.97	0.89	1.27	0.95
0.51	0.067	354.8	14.0	43.1	1.18	0.96	0.71	1.36
1.35	0.136	594.6	16.0	71.1	1.47	1.24	0.50	0.84
0.37	0.054	357.2	15.3	41.0	0.86	0.78	0.86	1.94
1.31	0.142	568.5	20.0	83.8	1.43	1.29	0.60	0.82
0.48	0.060	357.2	12.1	37.2	1.11	0.86	0.65	1.47
1.17	0.126	588.8	16.5	69.3	1.27	1.15	0.56	0.97
0.43	0.058	383.3	11.3	36.5	1.01	0.84	0.72	1.77
1.17	0.125	601.8	15.1	78.9	1.27	1.14	0.67	0.98
0.45	0.062	355.8	9.4	37.8	1.05	0.88	0.78	1.55
1.29	0.139	445.5	21.7	80.5	1.41	1.26	0.56	0.65
0.51	0.067	363.0	10.9	34.9	1.18	0.96	0.58	1.40
1.20	0.133	577.2	14.2	73.4	1.31	1.21	0.61	0.92
0.37	0.056	335.5	12.5	33.7	0.87	0.80	0.70	1.80
1.12	0.122	410.8	14.3	92.4	1.21	1.11	0.87	0.70
0.61	0.075	347.1	8.2	35.9	1.41	1.08	0.56	1.10
0.89	0.105	454.2	12.1	77.1	0.97	0.95	0.91	0.98
0.52	0.068	364.5	9.9	43.2	1.20	0.97	0.80	1.38
1.17	0.127	525.1	11.3	62.2	1.27	1.15	0.54	0.86
0.54	0.068	467.2	10.0	40.7	1.26	0.98	0.70	1.71
0.70	0.084	554.1	19.3	77.0	0.77	0.77	1.03	1.55
0.46	0.062	339.9	8.0	35.7	1.06	0.88	0.75	1.47
0.87	0.105	454.2	16.6	84.9	0.95	0.96	0.98	1.00
0.32	0.051	250.1	11.2	34.1	0.74	0.73	0.90	1.56
1.14	0.131	562.7	12.7	51.2	1.24	1.19	0.41	0.94
0.33	0.053	283.4	12.0	34.9	0.76	0.76	0.87	1.72
1.27	0.134	538.1	17.9	50.6	1.38	1.22	0.31	0.81
0.33	0.051	185.0	15.8	35.5	0.77	0.73	0.74	1.08
0.73	0.085	557.0	19.2	72.9	0.79	0.77	0.92	1.51
0.39	0.058	337.0	13.0	34.4	0.92	0.83	0.67	1.70
1.13	0.127	555.5	15.9	89.1	1.22	1.15	0.81	0.94
0.48	0.063	370.3	15.7	35.8	1.12	0.90	0.51	1.52
0.91	0.107	552.6	18.1	67.5	0.99	0.98	0.67	1.17
0.47	0.069	350.0	12.9	28.1	1.10	0.98	0.39	1.45
1.14	0.124	543.9	19.7	67.4	1.23	1.13	0.52	0.91
0.32	0.053	190.8	16.4	40.9	0.75	0.76	0.95	1.14

1.04	0.118	542.5	17.6	68.5	1.13	1.07	0.60	1.00
0.34	0.055	324.0	10.7	39.1	0.78	0.78	1.06	1.94
1.17	0.128	565.6	21.2	102.0	1.27	1.16	0.86	0.92
0.39	0.055	326.8	11.0	39.2	0.91	0.79	0.89	1.65
0.98	0.113	545.4	18.6	100.5	1.06	1.03	1.05	1.08
0.65	0.078	480.3	11.3	36.7	1.52	1.12	0.48	1.44
1.10	0.126	554.1	21.7	108.9	1.20	1.14	0.99	0.96
0.60	0.075	488.9	9.0	36.9	1.40	1.07	0.57	1.61
1.14	0.128	626.4	22.5	71.1	1.23	1.16	0.53	1.06
0.48	0.063	237.1	14.1	33.2	1.11	0.89	0.49	0.95
1.14	0.125	552.6	20.1	103.8	1.24	1.14	0.92	0.93
0.39	0.058	299.4	15.1	39.7	0.92	0.83	0.78	1.50
1.09	0.120	532.4	21.7	84.7	1.18	1.09	0.72	0.94
0.48	0.064	306.6	15.4	43.6	1.11	0.92	0.73	1.25
1.91	0.220	472.5	28.8	68.8	1.14	1.10	1.10	0.82
1.86	0.214	408.8	18.5	43.3	1.13	1.07	0.70	0.72
1.72	0.197	522.6	21.8	48.3	1.03	0.98	0.81	1.01
1.81	0.211	491.8	16.3	40.8	1.10	1.06	0.71	0.90
1.62	0.188	530.3	23.1	43.6	0.96	0.94	0.67	1.10
1.81	0.209	476.3	18.0	39.3	1.10	1.04	0.62	0.87
1.70	0.199	509.1	20.7	44.5	1.01	1.00	0.74	1.00
1.93	0.223	545.8	14.3	41.1	1.17	1.11	0.73	0.94
2.01	0.222	675.1	17.2	59.2	1.20	1.11	1.10	1.12
1.96	0.228	594.0	16.3	49.9	1.19	1.14	0.90	1.01
1.85	0.217	597.9	17.7	53.9	1.10	1.09	1.03	1.08
1.88	0.217	592.1	15.7	49.2	1.14	1.08	0.88	1.05
1.69	0.198	580.5	23.0	48.2	1.01	0.99	0.88	1.14
1.53	0.188	470.5	19.7	57.1	0.93	0.94	1.17	1.02
1.79	0.207	601.7	26.6	56.2	1.07	1.04	0.87	1.12
1.74	0.204	588.2	25.4	44.9	1.05	1.02	0.59	1.13
1.66	0.198	565.1	20.6	69.8	0.99	0.99	1.56	1.13
1.65	0.198	540.0	16.1	50.7	1.00	0.99	1.10	1.09
1.69	0.198	555.4	15.2	87.5	1.00	0.99	2.28	1.10
1.60	0.195	491.8	13.0	57.7	0.97	0.98	1.48	1.02
1.75	0.202	584.4	15.7	75.6	1.04	1.01	1.81	1.11
1.72	0.201	540.0	11.9	66.1	1.04	1.01	1.67	1.05
1.59	0.187	572.8	24.0	89.9	0.95	0.94	2.20	1.21
1.73	0.204	561.2	12.7	56.9	1.05	1.02	1.04	1.08
1.74	0.199	611.4	29.0	83.7	1.03	1.00	1.67	1.18
1.87	0.216	607.5	18.4	70.4	1.14	1.08	1.47	1.08
1.72	0.196	570.9	31.7	70.1	1.02	0.98	1.18	1.11
1.44	0.161	412.6	20.4	64.1	0.87	0.81	1.61	0.95
1.47	0.175	628.8	17.8	41.3	0.88	0.88	0.84	1.44
1.69	0.197	561.2	8.7	41.8	1.02	0.98	0.75	1.11
1.81	0.202	644.2	23.2	60.5	1.07	1.01	1.09	1.19
1.89	0.214	615.3	18.9	58.1	1.14	1.07	1.09	1.09
1.77	0.204	572.8	16.3	56.8	1.05	1.02	1.21	1.08

1.93	0.219	418.4	4.2	47.9	1.17	1.09	1.24	0.71
2.17	0.242	669.3	22.6	81.0	1.29	1.21	1.42	1.03
1.83	0.210	511.1	21.0	58.3	1.11	1.05	1.08	0.93
1.78	0.204	588.2	21.8	50.2	1.06	1.02	0.84	1.10
1.93	0.221	557.4	18.8	49.8	1.17	1.10	0.85	0.96
1.68	0.197	580.5	20.4	50.1	1.00	0.99	0.93	1.15
1.74	0.206	534.2	6.0	46.3	1.05	1.03	1.39	1.02
1.88	0.213	648.1	30.8	51.3	1.12	1.07	0.57	1.15
2.14	0.244	642.3	14.6	48.3	1.30	1.22	0.83	1.00
1.67	0.194	578.6	19.1	53.7	0.99	0.97	1.10	1.16
1.73	0.200	563.2	12.0	45.6	1.05	1.00	1.33	1.09
1.60	0.190	613.3	24.4	60.4	0.95	0.95	1.18	1.28
1.56	0.183	534.2	18.6	47.7	0.95	0.92	0.98	1.14
1.54	0.178	601.7	21.8	49.0	0.91	0.89	0.93	1.31
1.63	0.187	592.1	9.0	20.4	0.99	0.93	0.63	1.22
1.65	0.196	482.1	21.4	59.9	0.98	0.98	1.23	0.97
1.56	0.188	451.2	17.3	53.0	0.94	0.94	1.21	0.96
1.78	0.204	590.2	19.3	55.7	1.06	1.02	1.08	1.11
1.59	0.190	430.0	20.4	50.1	0.96	0.95	0.99	0.90
1.62	0.196	516.8	18.2	47.8	0.97	0.98	0.96	1.06
1.56	0.191	430.0	17.5	28.7	0.95	0.95	0.38	0.91
1.67	0.198	540.0	15.5	35.8	1.00	0.99	0.64	1.08
1.55	0.191	491.8	14.3	34.3	0.94	0.96	0.68	1.06
1.75	0.201	592.1	21.3	53.5	1.04	1.00	0.97	1.13
1.68	0.201	528.4	16.7	39.0	1.02	1.00	0.70	1.05
1.67	0.198	530.3	19.6	56.6	0.99	0.99	1.17	1.06
1.64	0.199	487.9	15.8	45.3	0.99	1.00	0.95	0.99
1.54	0.187	476.3	24.0	45.9	0.92	0.93	0.75	1.03
1.67	0.204	457.0	17.5	31.4	1.01	1.02	0.44	0.91
2.02	0.228	702.1	30.4	81.0	1.20	1.14	1.32	1.16
1.83	0.212	617.2	12.3	52.9	1.11	1.06	1.17	1.13
1.69	0.199	609.5	14.7	77.5	1.00	1.00	1.98	1.21
1.60	0.193	493.7	11.1	72.4	0.97	0.97	2.03	1.03
1.65	0.199	499.5	23.2	73.4	0.98	0.99	1.61	1.01
1.68	0.202	489.8	17.9	71.3	1.02	1.01	1.68	0.97
1.66	0.195	509.1	30.9	55.7	0.99	0.98	0.79	1.02
1.56	0.188	482.1	17.7	39.8	0.94	0.94	0.41	1.03
1.70	0.201	574.7	25.5	65.1	1.01	1.00	1.23	1.13
1.52	0.180	507.2	12.6	49.0	0.92	0.90	0.91	1.11
1.53	0.182	634.5	28.4	61.1	0.91	0.91	1.13	1.40
1.90	0.219	526.5	16.7	58.4	1.15	1.10	0.88	0.92
1.74	0.199	584.4	24.4	60.4	1.04	1.00	1.09	1.12
1.64	0.197	543.9	12.3	59.6	1.00	0.98	1.20	1.10
1.56	0.183	530.3	21.4	47.8	0.93	0.92	0.89	1.14
1.45	0.175	507.2	14.2	33.0	0.88	0.88	0.42	1.17
1.81	0.202	607.5	33.3	58.0	1.08	1.01	0.72	1.12
1.74	0.202	530.3	11.0	50.0	1.05	1.01	1.18	1.01

1.97	0.222	580.5	14.8	57.7	1.17	1.11	1.15	0.98
1.68	0.200	464.7	4.1	52.5	1.02	1.00	1.60	0.92
1.69	0.197	543.9	21.0	57.9	1.01	0.99	1.15	1.07
1.82	0.210	524.6	17.5	50.7	1.10	1.05	0.96	0.96
1.90	0.214	690.5	16.5	59.8	1.13	1.07	1.48	1.21
2.20	0.244	651.9	9.0	46.6	1.33	1.22	1.09	0.98
1.80	0.205	650.0	28.2	52.1	1.07	1.02	0.70	1.21
1.81	0.208	561.2	16.2	51.8	1.10	1.04	0.88	1.03
1.86	0.210	665.4	24.1	43.9	1.11	1.05	0.56	1.20
1.73	0.201	555.4	14.2	39.3	1.05	1.00	0.76	1.07
1.87	0.208	686.6	22.7	50.7	1.11	1.04	0.79	1.23
2.23	0.249	638.4	9.7	48.0	1.35	1.25	0.90	0.95
1.60	0.189	605.6	30.3	49.8	0.95	0.94	0.64	1.27
1.53	0.182	516.8	20.5	48.5	0.93	0.91	0.96	1.12
1.74	0.201	659.6	30.9	101.5	1.04	1.00	2.16	1.27
1.72	0.200	570.9	12.0	20.9	1.04	1.00	0.60	1.11
1.61	0.189	397.2	19.9	46.6	0.96	0.94	0.87	0.82
1.59	0.188	354.8	13.9	37.5	0.96	0.94	0.60	0.74
1.78	0.209	540.0	24.4	64.2	1.06	1.05	1.18	1.01
1.58	0.188	422.3	13.2	44.4	0.96	0.94	0.77	0.89
1.62	0.210	509.1	18.6	34.0	0.96	1.05	0.50	1.05
1.42	0.176	431.9	13.7	29.0	0.86	0.88	0.43	1.02
1.64	0.201	509.1	20.9	59.7	0.97	1.00	1.25	1.04
1.75	0.206	528.4	17.2	44.8	1.06	1.03	0.83	1.01
1.70	0.215	549.6	39.1	56.8	1.01	1.07	0.55	1.08
1.80	0.214	538.1	17.4	36.0	1.09	1.07	0.54	0.99
1.70	0.214	555.4	21.9	53.4	1.01	1.07	0.98	1.09
1.82	0.213	545.8	17.4	32.8	1.10	1.06	0.44	1.00
1.65	0.202	524.6	21.7	46.2	0.98	1.01	0.78	1.06
1.62	0.197	505.3	11.2	34.8	0.98	0.99	0.44	1.04
1.76	0.216	599.8	32.8	59.2	1.05	1.08	0.79	1.14
1.59	0.200	460.9	26.7	56.5	0.96	1.00	0.99	0.96
1.70	0.202	513.0	33.7	94.2	1.01	1.01	1.89	1.01
1.68	0.202	435.8	21.0	82.1	1.02	1.01	1.93	0.86
1.72	0.203	553.5	21.3	64.1	1.02	1.01	1.31	1.07
1.63	0.195	482.1	18.6	62.2	0.99	0.97	1.41	0.98
1.69	0.202	514.9	15.6	79.3	1.01	1.01	2.00	1.01
1.68	0.202	472.5	13.6	66.4	1.02	1.01	1.66	0.93
1.64	0.196	549.6	35.9	92.7	0.98	0.98	1.83	1.12
1.86	0.216	567.0	28.9	62.4	1.13	1.08	0.95	1.01
1.71	0.200	582.4	24.2	89.3	1.02	1.00	2.02	1.14
1.60	0.193	486.0	21.8	66.8	0.97	0.96	1.48	1.01
1.68	0.203	592.1	28.8	72.4	1.00	1.01	1.37	1.18
1.75	0.203	534.2	16.8	61.8	1.06	1.02	1.00	1.02
1.56	0.200	541.9	21.8	41.2	0.93	1.00	0.65	1.16
1.81	0.212	538.1	13.7	34.3	1.10	1.06	0.59	0.99
1.55	0.188	567.0	19.6	45.9	0.92	0.94	0.89	1.23

1.56	0.184	553.5	15.8	40.0	0.94	0.92	0.82	1.19
1.98	0.226	568.9	12.4	76.2	1.18	1.13	1.71	0.95
1.84	0.218	540.0	7.1	58.9	1.12	1.09	1.65	0.97
1.73	0.204	567.0	26.8	81.0	1.03	1.02	1.65	1.09
1.93	0.219	538.1	26.2	68.6	1.17	1.10	1.16	0.93
1.87	0.218	549.6	17.7	73.3	1.11	1.09	1.58	0.98
1.75	0.207	520.7	10.6	58.3	1.06	1.03	1.25	0.99
1.94	0.223	570.9	31.2	80.5	1.16	1.12	1.34	0.98
1.94	0.221	551.6	24.5	65.0	1.18	1.10	1.10	0.94
1.73	0.202	636.5	18.9	56.0	1.03	1.01	1.13	1.23
2.12	0.243	621.0	16.7	39.6	1.29	1.21	0.57	0.97
1.65	0.208	605.6	18.2	64.1	0.98	1.04	1.47	1.23
1.73	0.202	586.3	8.0	45.1	1.05	1.01	1.35	1.13
1.65	0.221	586.3	24.7	52.1	0.98	1.10	0.87	1.19
1.73	0.196	565.1	7.0	36.5	1.05	0.98	1.09	1.09
1.63	0.195	580.5	24.1	52.6	0.97	0.97	0.92	1.19
1.80	0.204	578.6	10.1	49.6	1.09	1.02	0.86	1.07

TN		AC		CWC		HWC		CPI
Mean	Std Dev	Mean	Std Dev	Mean	Std Dev	Mean	Std Dev	Mean
0.07	0.024	242.8	89.9	13.2	4.94	43.31	10.13	1.03
0.04	0.011	153.5	54.8	8.8	3.35	26.32	8.87	1.00
0.20	0.012	575.0	56.5	23.2	5.63	61.07	14.94	1.03
0.20	0.016	523.1	61.4	15.4	5.07	49.11	12.44	1.05
0.16	0.028	483.1	74.4	33.6	10.59	56.28	15.02	1.00
0.12	0.031	325.0	86.9	23.6	7.56	37.80	9.88	0.95
0.11	0.019	468.1	93.8	17.6	3.31	77.88	24.56	1.06
0.06	0.013	281.3	102.1	12.8	2.67	39.24	8.48	0.96

TN		AC		CWC		HWC		CPI
Mean	Std Dev	Mean	Std Dev	Mean	Std Dev	Mean	Std Dev	Mean
0.13	0.064	396.0	159.0	17.1	8.54	41.62	20.24	1.13
0.12	0.060	372.0	136.3	18.0	10.00	44.03	24.38	0.99
0.12	0.059	328.0	182.1	17.9	9.81	47.91	19.03	0.90
0.13	0.067	422.4	151.9	19.3	9.01	49.49	18.24	1.13
0.11	0.060	368.0	151.7	18.1	8.08	49.06	22.06	1.00
0.12	0.066	342.7	184.3	18.6	8.04	47.19	15.43	0.88
0.13	0.062	437.7	123.9	19.9	10.93	55.43	19.64	1.11
0.12	0.064	394.0	156.9	17.9	9.64	52.16	22.76	1.03
0.12	0.062	372.6	179.6	19.9	10.84	53.00	18.19	0.92

TN		AC		CWC		HWC		CPI
Mean	Std Dev	Mean	Std Dev	Mean	Std Dev	Mean	Std Dev	Mean
0.12	0.061	356.6	156.5	17.3	8.80	46.16	23.14	1.00
0.12	0.062	374.0	167.4	18.0	10.06	42.87	19.46	1.01
0.12	0.065	370.8	160.9	18.4	8.18	49.27	18.82	1.02
0.12	0.064	384.6	171.3	18.9	8.57	47.89	18.66	0.99
0.12	0.065	391.9	150.4	19.0	10.42	54.56	21.49	1.00
0.13	0.060	410.9	163.0	19.5	10.57	52.50	18.94	1.04

TN		AC		CWC		HWC		CPI
Mean	Std Dev	Mean	Std Dev	Mean	Std Dev	Mean	Std Dev	Mean
0.13	0.053	418.2	149.9	21.1	9.71	54.64	23.29	1.01
0.11	0.065	312.4	156.7	14.2	7.80	34.39	13.05	1.00
0.13	0.057	444.4	142.5	21.7	8.74	58.49	19.02	1.05
0.11	0.069	311.0	161.3	15.7	6.79	38.67	11.96	0.96
0.14	0.055	464.1	143.4	22.9	11.68	65.77	18.94	1.03
0.11	0.066	338.8	144.5	15.5	7.49	41.29	12.70	1.01

TN		AC		CWC		HWC		CPI
Mean	Std Dev	Mean	Std Dev	Mean	Std Dev	Mean	Std Dev	Mean

0.13	0.064	407.1	145.9	18.9	9.80	50.53	21.28	1.12
0.13	0.064	430.3	145.9	18.7	9.39	47.16	18.82	1.13
0.12	0.063	374.5	142.6	17.9	8.75	49.83	24.68	1.01
0.12	0.060	381.4	154.3	18.1	9.73	47.00	21.68	1.00
0.12	0.064	337.7	172.0	17.9	9.01	49.64	18.05	0.90
0.12	0.061	357.8	191.9	19.7	10.16	49.10	17.47	0.90

TN		AC		CWC		HWC		CPI
Mean	Std Dev	Mean	Std Dev	Mean	Std Dev	Mean	Std Dev	Mean
0.14	0.057	486.7	120.2	22.0	10.10	59.64	19.88	1.15
0.11	0.067	350.7	138.0	15.5	7.83	38.06	13.51	1.10
0.13	0.054	429.5	139.3	21.4	9.82	60.11	25.19	1.00
0.10	0.065	326.5	139.4	14.6	7.17	36.72	13.12	1.02
0.13	0.053	410.6	165.5	22.3	10.49	59.16	17.24	0.95
0.10	0.068	285.0	176.8	15.3	7.14	39.58	11.84	0.86

TN		AC		CWC		HWC		CPI
Mean	Std Dev	Mean	Std Dev	Mean	Std Dev	Mean	Std Dev	Mean
0.13	0.057	433.4	139.2	21.3	9.84	61.06	22.15	1.02
0.11	0.067	312.8	149.0	15.1	7.26	38.94	13.62	1.00
0.14	0.052	451.0	152.5	22.5	10.38	58.21	19.68	1.05
0.11	0.066	328.7	159.6	15.2	7.51	37.29	12.03	0.98

	TN		AC		CWC		HWC	
Std Dev	Mean	Std Dev	Mean	Std Dev	Mean	Std Dev	Mean	Std Dev
0.50	0.126	0.1	380.6	156.8	17.45	8.31	42.73	22.69
0.50	0.127	0.1	411.4	162.2	16.73	8.88	40.51	17.76
0.50	0.119	0.1	375.2	133.3	16.92	9.17	44.93	26.01
0.51	0.113	0.1	368.9	141.3	19.03	10.81	43.12	23.02
0.55	0.115	0.1	314.2	173.0	17.43	9.15	50.83	20.34
0.55	0.116	0.1	341.8	192.5	18.35	10.56	44.98	17.45
0.57	0.129	0.1	415.5	152.5	18.39	8.72	52.22	18.66
0.53	0.128	0.1	429.2	153.4	20.23	9.34	46.76	17.68
0.48	0.111	0.1	363.8	144.4	18.44	7.73	49.85	22.12
0.49	0.116	0.1	372.2	160.9	17.78	8.52	48.26	22.33
0.63	0.121	0.1	333.0	177.8	18.35	8.32	45.74	15.03
0.61	0.113	0.1	352.3	193.0	18.84	7.88	48.64	15.93
0.50	0.123	0.1	425.2	127.4	20.73	11.98	56.64	20.50
0.52	0.128	0.1	450.2	120.9	19.03	9.88	54.22	18.98
0.54	0.119	0.1	384.7	153.2	18.36	9.45	54.70	25.51
0.54	0.128	0.1	403.2	162.4	17.48	9.97	49.61	19.71
0.58	0.118	0.1	365.9	166.5	17.82	9.75	52.34	18.28

0.58	0.125	0.1	379.3	194.3	21.92	11.62	53.67	18.38

	TN		AC		CWC		HWC	
Std Dev	Mean	Std Dev	Mean	Std Dev	Mean	Std Dev	Mean	Std Dev
0.38	0.143	0.1	453.5	148.2	20.10	8.59	51.11	22.30
0.53	0.110	0.1	338.4	150.3	14.07	7.45	32.12	12.22
0.43	0.128	0.1	420.6	130.9	21.38	10.36	54.84	28.12
0.54	0.104	0.1	323.5	125.4	14.57	8.49	33.21	13.25
0.41	0.129	0.0	380.6	164.4	21.74	10.32	57.96	18.69
0.62	0.102	0.1	275.4	186.2	14.04	7.65	37.85	13.34
0.38	0.145	0.1	492.4	123.0	22.12	9.19	59.55	17.13
0.61	0.113	0.1	352.4	147.2	16.50	8.02	39.43	13.18
0.38	0.128	0.1	426.5	136.3	21.28	8.68	61.11	23.74
0.51	0.099	0.1	309.5	145.5	14.94	6.05	37.00	11.20
0.49	0.131	0.1	414.3	157.9	21.60	8.59	54.82	15.15
0.68	0.103	0.1	271.0	183.0	15.59	6.25	39.57	11.60
0.39	0.142	0.1	514.2	72.4	23.69	12.16	68.24	16.39
0.54	0.109	0.1	361.2	117.7	16.07	8.04	42.62	13.27
0.41	0.138	0.1	441.3	153.3	21.59	10.61	64.37	23.27
0.62	0.109	0.1	346.6	148.0	14.25	6.97	39.95	14.26
0.48	0.137	0.1	436.7	174.2	23.55	12.44	64.69	16.72
0.63	0.106	0.1	308.5	163.5	16.18	7.49	41.32	10.55

	TN		AC		CWC		HWC	
Std Dev	Mean	Std Dev	Mean	Std Dev	Mean	Std Dev	Mean	Std Dev
0.42	0.132	0.1	413.2	142.0	20.29	9.18	57.13	25.50
0.56	0.107	0.1	300.1	151.0	14.24	7.32	35.19	13.67
0.40	0.135	0.1	423.3	158.8	21.86	10.26	52.15	20.82
0.58	0.103	0.1	324.8	162.8	14.21	8.32	33.59	12.49
0.45	0.135	0.1	436.5	137.8	20.90	8.53	59.05	19.20
0.61	0.106	0.1	305.1	156.5	15.88	7.04	39.50	12.33
0.41	0.134	0.1	452.3	148.1	22.43	8.96	57.94	19.02
0.61	0.104	0.1	316.9	167.4	15.47	6.59	37.84	11.63
0.44	0.133	0.1	450.7	138.1	22.80	11.58	66.99	20.44
0.60	0.108	0.1	333.1	140.0	15.14	7.46	42.14	14.15
0.43	0.146	0.0	477.5	148.7	23.09	11.91	64.55	17.44
0.59	0.108	0.1	344.4	150.2	15.86	7.58	40.45	11.16

	TN		AC		CWC		HWC	
Std Dev	Mean	Std Dev	Mean	Std Dev	Mean	Std Dev	Mean	Std Dev
0.39	0.140	0.1	475.1	117.1	21.85	10.62	61.96	20.48

0.56	0.112	0.1	339.0	140.8	15.87	7.95	39.09	15.13
0.37	0.146	0.1	498.3	123.3	22.10	9.67	57.31	19.19
0.56	0.110	0.1	362.3	135.6	15.22	7.78	37.02	11.73
0.42	0.128	0.1	425.4	135.8	20.89	9.11	61.81	27.22
0.55	0.104	0.1	323.6	131.8	14.92	7.32	37.85	14.03
0.39	0.135	0.1	433.5	144.0	21.94	10.55	58.40	23.15
0.57	0.104	0.1	329.4	147.9	14.25	7.07	35.59	12.20
0.47	0.131	0.1	399.8	154.3	21.25	9.90	59.39	18.11
0.64	0.105	0.1	275.6	167.6	14.48	6.52	39.89	11.68
0.45	0.134	0.0	421.3	177.0	23.35	11.05	58.92	16.52
0.64	0.102	0.1	294.3	186.7	16.06	7.70	39.27	12.12

	SOC		TN		AC		CWC	
Std Dev	Mean	Std Dev	Mean	Std Dev	Mean	Std Dev	Mean	Std Dev
0.37	0.920	0.2	0.1	0.0	236.98	134.22	13.90	6.46
0.40	0.553	0.0	0.0	0.0	129.65	82.73	11.53	5.11
0.28	1.018	0.1	0.1	0.0	224.08	71.93	12.88	5.47
0.51	0.615	0.1	0.0	0.0	165.90	40.60	8.53	3.33
0.31	0.840	0.2	0.1	0.0	239.35	19.11	11.15	3.39
0.70	0.518	0.1	0.0	0.0	190.23	40.37	6.53	4.83
0.45	0.788	0.2	0.1	0.0	238.40	14.54	11.10	2.95
1.56	0.395	0.1	0.0	0.0	168.30	13.35	8.28	0.55
0.40	0.628	0.1	0.1	0.0	194.98	44.30	11.58	2.41
1.61	0.280	0.1	0.0	0.0	106.28	21.30	7.73	1.59
0.75	0.730	0.2	0.1	0.0	189.28	61.43	12.85	3.06
2.20	0.300	0.2	0.0	0.0	99.10	36.96	5.80	1.17
0.56	0.940	0.1	0.1	0.0	298.03	48.43	13.70	5.11
0.98	0.510	0.3	0.0	0.0	159.70	7.11	9.90	6.07
0.22	1.028	0.2	0.1	0.0	308.55	100.37	19.00	4.31
3.02	0.515	0.3	0.0	0.0	178.80	70.24	9.73	1.87
1.50	0.800	0.3	0.1	0.0	211.70	34.45	13.83	4.22
1.11	0.560	0.2	0.0	0.0	164.93	21.60	12.18	3.94
0.26	0.893	0.1	0.1	0.0	236.03	44.53	14.95	7.72
0.40	0.520	0.2	0.0	0.0	164.98	12.79	9.03	2.81
1.05	0.740	0.3	0.1	0.0	195.00	43.23	15.10	2.97
1.26	0.335	0.2	0.0	0.0	102.45	28.18	9.18	2.28
0.40	0.603	0.1	0.1	0.0	182.58	91.40	12.90	3.93
3.65	0.198	0.1	0.0	0.0	99.60	35.50	9.60	4.04
0.35	1.023	0.2	0.1	0.0	389.13	77.43	12.00	4.22
1.45	0.553	0.3	0.0	0.0	185.93	80.64	9.63	4.61
0.47	1.050	0.1	0.1	0.0	438.73	10.62	10.20	1.89
0.39	0.560	0.1	0.0	0.0	245.55	70.75	6.48	2.23
1.38	0.755	0.1	0.0	0.0	216.95	13.03	10.48	2.64
0.70	0.443	0.1	0.0	0.0	184.53	19.41	9.23	1.42

0.61	0.938	0.3	0.1	0.0	240.78	5.94	9.55	3.87
1.03	0.568	0.3	0.1	0.0	183.10	16.27	5.98	2.79
2.01	0.558	0.2	0.1	0.0	179.73	70.45	14.48	5.38
1.04	0.308	0.0	0.0	0.0	118.18	36.63	8.48	1.04
1.39	0.640	0.2	0.1	0.0	150.15	30.09	18.68	9.92
2.85	0.358	0.2	0.1	0.0	115.33	21.63	10.83	1.83
0.36	1.648	0.0	0.2	0.0	492.23	66.72	20.18	1.20
0.40	1.598	0.1	0.2	0.0	449.30	74.31	15.85	2.44
0.33	1.658	0.0	0.2	0.0	580.50	23.23	26.53	8.88
0.30	1.765	0.0	0.2	0.0	567.03	21.18	10.63	4.70
0.19	1.738	0.2	0.2	0.0	545.30	26.34	17.38	2.83
0.47	1.653	0.1	0.2	0.0	477.78	40.18	13.35	6.35
0.22	1.720	0.1	0.2	0.0	569.88	83.39	25.25	5.76
0.29	1.695	0.0	0.2	0.0	507.65	43.77	14.63	2.77
0.63	1.663	0.0	0.2	0.0	557.83	21.06	19.83	3.32
0.31	1.700	0.1	0.2	0.0	510.58	31.36	13.28	5.27
0.36	1.605	0.1	0.2	0.0	605.13	20.94	21.93	5.00
0.35	1.730	0.1	0.2	0.0	581.00	22.62	12.03	4.50
0.14	1.880	0.1	0.2	0.0	564.10	9.80	22.03	8.53
0.24	1.865	0.1	0.2	0.0	537.60	12.75	17.10	9.66
0.18	1.688	0.1	0.2	0.0	583.40	43.18	23.85	6.13
0.22	1.835	0.2	0.2	0.0	541.45	65.44	18.63	5.62
0.19	1.698	0.1	0.2	0.0	539.03	51.73	24.28	5.11
0.29	1.558	0.0	0.2	0.0	467.63	33.97	17.00	3.24
0.51	1.698	0.1	0.2	0.0	591.60	53.02	24.50	4.50
0.31	1.623	0.1	0.2	0.0	537.60	26.82	13.80	2.16
0.26	1.890	0.2	0.2	0.0	559.78	87.54	23.48	3.63
0.17	1.888	0.1	0.2	0.0	505.78	67.58	18.15	2.80
0.26	1.775	0.1	0.2	0.0	610.93	40.89	27.18	4.72
0.29	1.750	0.3	0.2	0.0	535.18	111.21	18.40	2.61
0.31	1.703	0.1	0.2	0.0	529.38	18.25	27.40	9.31
0.50	1.700	0.1	0.2	0.0	474.40	65.28	19.18	7.41
0.50	1.648	0.1	0.2	0.0	566.53	29.80	23.58	3.96
0.38	1.633	0.1	0.2	0.0	519.75	29.98	16.40	4.35
0.47	1.760	0.1	0.2	0.0	612.38	82.20	20.65	6.22
0.46	1.823	0.3	0.2	0.0	549.15	75.94	13.55	4.19
0.28	1.755	0.2	0.2	0.0	657.20	44.94	27.95	3.62
0.10	1.873	0.3	0.2	0.0	574.73	62.02	14.80	4.77
0.53	1.708	0.1	0.2	0.0	563.15	27.58	19.45	4.09
0.38	1.798	0.1	0.2	0.0	502.85	63.35	11.28	5.09
0.13	1.813	0.2	0.2	0.0	622.00	36.00	21.48	4.74
0.17	1.785	0.2	0.2	0.0	577.13	28.72	19.00	4.45
0.13	1.728	0.3	0.2	0.0	543.20	27.16	26.65	14.14
0.48	1.238	0.4	0.1	0.0	383.28	75.58	22.20	11.91
0.10	1.735	0.3	0.2	0.0	543.20	32.20	24.13	10.68
0.24	1.195	0.3	0.1	0.0	370.98	62.52	21.25	10.12
0.17	1.405	0.3	0.2	0.0	464.35	89.21	34.28	4.13

0.17	1.245	0.4	0.1	0.0	312.73	115.09	22.73	7.76
0.30	1.220	0.3	0.1	0.0	390.15	119.46	34.40	14.49
0.17	0.835	0.3	0.1	0.0	282.73	54.50	26.05	13.31
0.65	1.260	0.2	0.1	0.0	464.35	57.43	36.18	4.88
0.50	0.820	0.3	0.1	0.0	285.25	103.67	21.58	6.13
1.01	1.408	0.3	0.2	0.0	497.60	49.57	37.93	7.67
1.64	1.113	0.5	0.1	0.0	316.70	118.37	26.48	7.34
0.70	1.648	0.3	0.2	0.0	521.53	67.92	27.73	11.09
0.29	1.238	0.3	0.1	0.0	378.23	103.38	24.03	9.44
0.48	1.690	0.3	0.2	0.0	554.03	79.64	36.38	8.39
0.73	1.083	0.3	0.1	0.0	353.60	55.48	26.78	4.50
0.70	1.260	0.3	0.1	0.0	470.83	83.16	26.55	12.50
2.03	0.735	0.4	0.1	0.0	236.40	113.97	20.25	11.74
0.15	1.410	0.3	0.2	0.0	435.73	78.04	32.20	9.03
0.26	1.083	0.3	0.1	0.0	280.18	90.26	21.30	4.79
1.20	1.395	0.3	0.1	0.0	466.50	49.65	32.15	11.28
0.75	1.033	0.4	0.1	0.0	324.70	96.81	22.65	5.27
0.33	1.368	0.0	0.1	0.0	487.13	44.41	30.08	4.97
0.37	0.945	0.3	0.1	0.0	324.30	123.62	21.80	8.12
0.58	1.553	0.2	0.2	0.0	522.20	10.05	43.93	7.08
1.40	1.188	0.4	0.1	0.0	366.65	78.94	24.98	6.10
0.39	1.793	0.4	0.2	0.0	534.15	72.00	37.28	2.85
0.53	1.198	0.2	0.1	0.0	383.30	59.06	27.50	2.26
0.59	1.358	0.2	0.2	0.0	429.23	64.71	34.70	8.54
0.63	1.043	0.3	0.1	0.0	308.05	53.46	24.90	9.25
0.50	1.428	0.3	0.2	0.0	442.28	60.76	30.88	14.68
1.16	1.035	0.2	0.1	0.0	327.23	62.72	18.10	6.82
1.18	1.368	0.4	0.1	0.0	460.70	104.36	34.68	14.39
2.44	0.915	0.4	0.1	0.0	327.58	92.17	23.93	5.41
1.03	1.373	0.2	0.2	0.0	467.95	41.89	44.00	14.58
1.01	0.898	0.4	0.1	0.0	287.78	65.32	27.75	6.77
0.81	1.058	0.1	0.1	0.0	490.75	69.68	18.08	3.28
0.78	0.463	0.2	0.1	0.0	319.23	130.18	11.20	3.69
0.65	1.090	0.2	0.1	0.0	517.15	85.00	18.48	4.76
0.94	0.463	0.1	0.1	0.0	322.15	59.72	11.40	1.85
2.02	0.780	0.1	0.1	0.0	459.63	8.74	18.03	3.28
1.01	0.510	0.1	0.1	0.0	312.05	66.99	11.90	3.27
2.07	0.855	0.1	0.1	0.0	457.80	21.91	19.48	4.47
1.16	0.435	0.1	0.1	0.0	336.28	30.00	13.10	2.55
1.78	0.935	0.2	0.1	0.0	269.35	22.08	16.23	3.48
3.57	0.310	0.1	0.1	0.0	124.95	45.04	13.05	4.88
1.01	0.963	0.3	0.1	0.0	266.45	31.06	17.45	3.73
1.30	0.425	0.2	0.1	0.0	179.23	72.61	12.38	1.13
0.63	1.210	0.1	0.1	0.0	556.95	26.49	19.03	2.29
0.14	0.430	0.1	0.1	0.0	308.03	87.98	13.65	2.28
0.80	1.153	0.0	0.1	0.0	552.23	18.58	15.28	4.39
0.66	0.435	0.1	0.1	0.0	361.93	78.64	12.18	2.49

0.81	0.978	0.1	0.1	0.0	464.70	23.11	19.10	1.90
0.30	0.480	0.1	0.1	0.0	354.83	10.70	14.33	2.51
1.18	0.970	0.1	0.1	0.0	462.18	12.76	14.80	1.42
4.50	0.368	0.2	0.1	0.0	269.33	152.91	11.65	1.29
0.57	0.908	0.2	0.1	0.0	389.43	64.39	13.90	2.38
2.52	0.295	0.1	0.1	0.0	120.60	52.58	12.18	1.47
1.77	0.818	0.1	0.1	0.0	423.43	49.38	18.05	0.93
0.68	0.255	0.0	0.0	0.0	155.35	19.17	12.75	2.29
0.84	0.948	0.2	0.1	0.0	557.33	13.78	17.55	2.29
0.68	0.488	0.1	0.1	0.0	376.43	69.52	11.18	2.22
0.78	1.098	0.3	0.1	0.0	576.50	25.29	17.60	2.42
0.29	0.400	0.0	0.1	0.0	337.35	26.92	13.20	2.74
1.89	0.893	0.2	0.1	0.0	451.68	14.14	20.30	3.24
0.50	0.418	0.1	0.1	0.0	325.40	71.21	13.08	0.88
1.02	1.000	0.2	0.1	0.0	479.90	40.77	18.20	4.08
0.63	0.400	0.1	0.1	0.0	320.68	74.13	14.35	4.44
0.67	0.925	0.2	0.1	0.1	496.53	55.07	17.98	3.08
2.41	0.430	0.2	0.1	0.0	278.38	33.36	12.33	1.93
0.48	0.965	0.1	0.1	0.0	553.33	21.35	17.70	4.85
0.98	0.405	0.2	0.1	0.0	260.80	76.12	15.90	3.47

CWCCLI	HWCCLI	MBCCMI1	ACCMI1	CWCCMI1	HWCCMI1	MBCCMI	ACCMI	CWCCMI
0.88	1.53	77.1	77.5	45.4	71.7	2.63	1.06	0.78
1.44	1.55	32.4	46.8	51.0	50.0	1.10	0.64	0.88
1.07	1.37	56.3	80.3	58.9	68.5	1.90	1.09	1.01
1.72	1.77	36.5	39.7	65.0	61.0	1.24	0.54	1.12
1.87	1.64	24.7	69.8	79.5	63.2	0.84	0.95	1.37
0.88	0.77	17.9	46.6	59.2	46.8	0.61	0.63	1.01
1.55	1.84	51.6	58.7	64.2	69.1	1.75	0.80	1.10
1.67	1.49	14.0	29.3	41.9	33.9	0.47	0.40	0.72
0.97	1.42	62.8	77.3	50.7	67.1	2.12	1.05	0.86
1.29	1.52	35.5	55.4	45.4	48.3	1.20	0.75	0.77
1.28	1.71	78.1	83.8	75.1	90.8	2.64	1.14	1.28
2.14	2.22	48.7	57.2	85.7	80.6	1.66	0.79	1.48
1.10	1.50	66.1	79.4	60.6	75.1	2.25	1.08	1.05
2.03	2.02	34.3	57.6	68.7	62.0	1.17	0.78	1.18
0.71	1.56	84.0	64.5	34.6	68.7	2.86	0.88	0.59
1.38	2.63	91.3	50.6	45.4	78.5	3.08	0.68	0.77
1.89	1.86	44.3	76.3	92.8	82.7	1.50	1.04	1.59
2.22	2.08	29.2	56.8	71.9	61.1	0.98	0.76	1.22
1.81	2.14	75.1	73.2	93.0	100.0	2.55	0.99	1.59
2.45	2.25	31.9	56.7	84.7	70.5	1.08	0.77	1.45
2.09	2.25	63.6	74.5	100.0	97.9	2.16	1.02	1.71
1.99	1.99	31.4	49.8	62.5	56.5	1.07	0.68	1.07
0.76	0.88	25.9	61.5	33.9	35.8	0.89	0.84	0.59
0.96	0.88	13.5	53.0	37.2	30.7	0.46	0.72	0.63
0.89	1.18	48.1	74.4	47.7	57.0	1.63	1.01	0.81
0.50	0.78	48.4	100.0	35.4	49.7	1.65	1.37	0.61
1.11	1.42	45.9	52.2	48.2	56.0	1.55	0.71	0.82
0.86	1.07	40.7	67.3	45.7	51.6	1.38	0.92	0.78
1.39	1.39	35.5	72.1	69.4	63.2	1.21	0.99	1.20
0.76	0.93	36.0	75.4	41.5	46.3	1.22	1.02	0.71
0.77	1.10	50.1	64.9	42.8	55.1	1.71	0.88	0.73
0.57	0.88	45.3	66.8	33.1	46.5	1.54	0.91	0.57
1.01	1.29	47.6	70.5	50.6	58.5	1.63	0.96	0.87
1.44	1.90	55.3	53.4	54.4	64.9	1.86	0.72	0.92
1.18	1.32	47.6	77.4	68.0	69.3	1.62	1.06	1.17
1.20	1.20	36.0	72.2	71.2	64.6	1.22	0.99	1.22
0.85	0.95	46.3	89.4	66.4	67.6	1.57	1.22	1.14
0.77	0.88	43.3	92.5	58.5	61.0	1.47	1.26	1.00
0.81	0.79	28.3	81.6	59.6	53.1	0.96	1.11	1.02
0.51	0.43	12.2	99.5	46.1	35.5	0.42	1.36	0.80
0.78	1.65	100.0	77.6	43.3	83.0	3.40	1.05	0.74
1.01	1.71	74.9	50.3	46.4	71.0	2.53	0.68	0.79

1.41	1.17	18.1	69.8	74.8	56.5	0.61	0.95	1.28
1.09	0.88	13.3	75.2	66.8	48.8	0.45	1.03	1.14
0.96	1.16	57.8	77.1	70.5	76.7	1.96	1.05	1.20
0.65	0.82	49.8	88.6	54.6	62.4	1.70	1.21	0.94
1.10	1.24	51.1	61.9	71.1	73.2	1.73	0.84	1.22
0.81	0.96	50.7	77.3	63.9	68.7	1.73	1.06	1.10
1.64	1.52	27.5	62.8	69.7	58.8	0.93	0.85	1.20
2.49	2.19	22.0	30.1	69.7	55.6	0.75	0.41	1.20
1.29	1.21	23.8	53.5	57.5	49.1	0.82	0.73	0.99
1.43	1.29	18.1	38.6	50.7	41.6	0.62	0.52	0.87
1.59	1.37	24.5	60.9	85.6	66.9	0.84	0.83	1.46
2.09	1.73	22.2	51.5	95.9	71.8	0.75	0.69	1.63
1.56	1.33	18.3	55.8	67.4	52.1	0.62	0.76	1.15
1.38	1.14	14.9	46.7	62.1	46.8	0.50	0.63	1.06
1.33	1.11	15.1	63.5	62.4	47.2	0.51	0.86	1.06
1.36	1.01	7.7	39.7	73.1	49.4	0.26	0.54	1.25
1.59	1.28	15.8	66.1	78.1	57.2	0.54	0.90	1.34
1.98	1.67	19.8	44.5	76.6	58.6	0.67	0.60	1.31
1.52	1.45	34.0	63.5	78.0	67.6	1.15	0.86	1.34
1.12	1.22	39.9	44.1	61.4	60.8	1.35	0.60	1.05
1.21	1.58	62.5	58.5	63.2	74.6	2.12	0.79	1.08
1.59	1.77	50.0	60.0	73.1	73.7	1.71	0.82	1.26
1.73	1.59	30.5	58.0	80.7	67.3	1.04	0.79	1.38
2.13	1.78	19.2	48.5	79.4	60.0	0.65	0.67	1.36
1.43	1.69	62.0	69.1	77.3	83.1	2.11	0.94	1.33
1.04	1.67	78.8	60.7	52.6	77.1	2.68	0.83	0.90
1.03	1.22	59.9	79.1	74.1	80.1	2.03	1.08	1.28
0.77	0.95	49.6	85.3	56.6	63.5	1.68	1.16	0.96
0.87	0.87	29.3	90.5	59.8	53.8	0.99	1.23	1.02
0.98	0.95	32.2	81.1	71.3	62.6	1.10	1.11	1.23
0.75	0.89	35.1	67.1	43.5	47.0	1.19	0.92	0.75
0.55	0.72	33.6	66.0	34.4	40.6	1.14	0.90	0.59
0.77	0.98	47.0	79.2	49.3	57.4	1.60	1.08	0.85
0.54	0.77	46.9	72.3	39.7	51.6	1.60	0.98	0.68
0.74	0.67	17.8	83.0	50.3	41.3	0.60	1.14	0.86
0.42	0.43	19.2	86.7	35.6	33.0	0.65	1.19	0.61
0.65	0.86	46.5	82.2	45.8	55.1	1.57	1.11	0.79
0.66	0.75	36.3	77.4	50.7	52.2	1.23	1.05	0.86
0.67	0.71	23.9	77.6	39.0	37.9	0.81	1.05	0.67
0.50	0.56	22.5	66.3	31.3	32.3	0.77	0.91	0.54
0.88	0.83	24.0	82.1	59.1	50.3	0.82	1.12	1.01
0.49	0.50	21.2	79.0	39.6	36.7	0.72	1.08	0.68
0.91	1.17	47.1	66.8	49.0	57.3	1.61	0.91	0.85
0.59	0.77	37.9	68.7	38.5	45.7	1.29	0.93	0.65
1.10	1.13	20.2	34.1	37.5	34.8	0.68	0.46	0.64
1.27	1.13	12.3	68.5	37.9	30.4	0.42	0.93	0.65
0.72	1.27	63.8	56.9	37.0	59.2	2.17	0.77	0.63

0.71	1.12	51.0	61.0	35.8	51.3	1.74	0.84	0.62
0.71	1.15	46.9	71.7	31.4	46.2	1.59	0.97	0.54
0.94	2.08	54.1	35.2	21.8	43.6	1.82	0.47	0.37
0.53	0.97	44.6	64.7	24.4	40.6	1.51	0.88	0.42
0.77	1.44	47.9	45.7	24.9	42.5	1.64	0.63	0.43
0.53	1.17	65.2	65.1	26.8	53.7	2.22	0.89	0.46
0.50	1.39	75.9	58.5	22.3	56.6	2.58	0.79	0.39
0.91	1.30	49.6	70.5	41.6	54.1	1.68	0.95	0.71
1.03	1.07	30.2	45.7	53.0	50.1	1.02	0.62	0.91
0.95	1.14	39.7	71.3	48.8	53.0	1.36	0.98	0.84
1.51	1.71	44.5	61.5	61.3	63.2	1.52	0.84	1.06
0.91	0.94	35.1	79.3	62.9	59.0	1.19	1.07	1.07
1.51	1.48	36.2	67.7	75.4	67.3	1.24	0.93	1.30
1.15	1.27	45.5	79.7	68.2	68.2	1.55	1.08	1.16
0.62	0.88	52.1	82.6	44.0	57.2	1.77	1.12	0.76
0.98	0.88	23.6	87.2	70.0	56.8	0.81	1.18	1.20
1.25	1.06	22.0	78.6	82.7	63.6	0.75	1.07	1.43
0.87	0.76	19.6	82.0	66.2	52.1	0.66	1.12	1.13
1.08	0.92	21.2	76.6	76.4	59.3	0.71	1.04	1.31
0.89	0.82	25.6	87.6	69.1	57.4	0.86	1.20	1.18
0.92	0.77	17.4	93.5	73.8	55.5	0.59	1.27	1.26
1.06	0.98	32.9	99.2	83.5	70.5	1.11	1.35	1.43
0.66	0.79	40.2	87.3	49.8	53.9	1.36	1.19	0.84
0.53	0.61	19.6	78.5	27.2	28.1	0.66	1.07	0.46
0.88	0.98	21.0	57.9	31.2	31.3	0.71	0.78	0.53
0.58	0.55	14.2	78.1	33.2	28.7	0.48	1.06	0.57
0.92	0.78	8.9	57.1	34.0	26.1	0.30	0.77	0.58
0.46	0.47	16.5	79.8	30.9	28.6	0.56	1.08	0.53
0.43	0.38	10.0	84.6	31.9	25.4	0.34	1.14	0.55
0.32	0.41	21.8	84.1	23.2	27.0	0.74	1.15	0.40
0.32	0.32	12.2	83.1	24.1	21.9	0.42	1.13	0.42
0.82	0.77	24.2	89.4	58.4	50.0	0.82	1.22	1.00
0.96	0.91	26.4	70.8	61.6	53.2	0.90	0.97	1.06
1.19	1.23	45.0	78.4	80.5	75.5	1.53	1.07	1.38
0.87	0.97	40.5	74.3	58.3	59.3	1.38	1.01	1.00
1.29	1.15	30.9	81.9	92.3	74.7	1.05	1.11	1.57
0.94	0.87	30.2	88.0	77.1	65.0	1.03	1.20	1.33
0.42	0.44	20.2	87.0	34.4	32.9	0.69	1.18	0.59
0.63	0.50	8.4	80.7	48.0	34.4	0.29	1.09	0.82
1.31	1.52	49.4	80.2	65.4	68.6	1.67	1.09	1.11
1.64	2.16	53.1	52.5	52.0	62.1	1.80	0.71	0.89
1.16	1.50	57.9	66.9	59.9	70.0	1.96	0.92	1.02
1.28	1.56	48.7	60.4	56.8	62.9	1.65	0.82	0.97
1.06	1.25	56.0	82.7	71.3	76.1	1.90	1.13	1.22
0.92	1.16	56.9	86.7	62.7	71.4	1.93	1.18	1.07
0.55	0.73	39.9	83.3	39.4	47.4	1.35	1.13	0.67
0.57	0.63	31.4	89.9	45.9	46.5	1.07	1.22	0.79

0.85	1.13	67.4	83.5	66.3	79.6	2.29	1.13	1.13
1.02	1.31	65.4	83.7	68.4	79.7	2.21	1.14	1.16
1.12	1.14	41.7	72.7	77.2	71.6	1.42	0.99	1.32
1.54	1.40	31.0	80.9	87.0	71.5	1.05	1.09	1.48
0.45	0.58	32.8	92.6	35.1	40.6	1.12	1.26	0.60
0.50	0.57	26.8	90.4	36.6	38.1	0.91	1.23	0.63
0.69	0.94	67.3	92.9	62.5	77.3	2.28	1.26	1.07
0.89	0.99	45.1	80.4	66.7	67.1	1.52	1.09	1.14
1.49	1.20	40.6	39.0	55.0	53.4	0.86	0.90	1.19
1.85	1.64	41.8	41.5	48.2	51.7	0.87	0.95	1.04
0.84	1.04	55.4	44.4	40.2	60.0	1.18	1.03	0.87
1.78	2.58	97.8	38.0	56.8	98.9	2.07	0.88	1.23
1.66	1.22	43.6	35.5	67.9	60.5	0.93	0.83	1.48
1.70	1.21	32.5	36.8	53.9	46.3	0.69	0.86	1.17
1.41	1.06	32.9	24.9	49.7	45.2	0.70	0.58	1.09
2.20	1.27	35.4	40.1	87.8	61.1	0.74	0.93	1.89
0.92	0.67	27.9	31.0	45.0	39.4	0.59	0.72	0.98
0.63	0.48	36.1	33.8	34.2	31.1	0.77	0.79	0.74
1.77	0.95	34.2	27.7	99.6	64.5	0.72	0.65	2.16
0.89	0.60	32.7	41.3	59.3	48.6	0.70	0.96	1.29
1.81	1.53	41.6	31.9	52.2	53.1	0.88	0.74	1.14
1.83	1.88	58.1	40.6	53.9	66.8	1.23	0.94	1.17
1.01	1.03	50.5	47.5	47.9	58.9	1.06	1.10	1.04
2.07	2.99	59.4	44.2	34.3	59.7	1.26	1.03	0.75
1.34	1.39	57.7	54.1	53.4	66.6	1.21	1.26	1.15
2.06	4.39	96.4	33.0	33.6	86.4	2.01	0.76	0.72
0.90	0.96	35.3	46.0	31.6	40.5	0.74	1.06	0.68
1.22	1.77	68.0	52.8	40.0	69.7	1.44	1.23	0.87
0.86	1.13	40.3	32.8	27.2	42.9	0.84	0.75	0.58
0.87	1.14	52.2	38.3	35.5	55.6	1.10	0.89	0.77
0.84	0.85	55.4	40.4	53.2	64.9	1.16	0.93	1.15
0.60	0.72	46.8	27.9	35.8	51.6	0.99	0.65	0.77
1.08	1.14	50.8	28.1	45.6	58.2	1.08	0.65	0.99
1.41	1.89	78.7	30.2	40.0	64.4	1.65	0.70	0.86
0.94	1.21	54.0	30.2	36.8	57.6	1.14	0.70	0.80
4.60	4.02	45.8	37.5	52.9	55.7	0.97	0.87	1.15
2.79	2.49	57.9	19.8	65.7	70.7	1.22	0.46	1.42
4.19	4.13	80.5	30.2	77.2	91.8	1.70	0.70	1.68
1.24	1.46	58.5	28.9	45.5	64.2	1.23	0.67	0.98
1.92	2.73	67.8	28.8	40.2	68.8	1.42	0.66	0.86
1.01	1.15	40.8	18.0	33.4	45.6	0.86	0.42	0.73
1.69	1.68	50.4	26.6	49.0	58.9	1.06	0.62	1.06
0.79	0.71	30.3	27.1	34.5	37.5	0.64	0.63	0.74
3.00	2.52	50.5	20.8	63.0	63.8	1.08	0.49	1.38
1.22	1.12	32.1	27.8	35.6	39.3	0.67	0.64	0.77
2.03	2.27	54.2	25.7	44.4	59.9	1.13	0.59	0.95
2.70	3.03	66.0	24.4	53.4	72.2	1.40	0.57	1.16

1.55	2.04	77.0	29.5	51.0	80.7	1.63	0.68	1.10
1.90	1.90	53.4	26.8	51.5	62.1	1.13	0.63	1.12
1.99	1.36	60.9	35.6	62.9	51.6	1.28	0.82	1.35
1.04	1.19	32.0	16.5	25.8	35.6	0.68	0.38	0.56
3.52	2.99	58.0	15.1	45.9	47.0	1.22	0.35	0.99
0.90	1.17	45.7	22.7	26.7	41.6	0.97	0.52	0.58
1.04	1.71	52.6	14.8	24.0	47.5	1.11	0.35	0.52
0.58	1.18	58.1	23.2	22.7	55.7	1.23	0.54	0.49
1.78	1.34	61.4	29.5	57.2	51.8	1.30	0.69	1.25
1.51	1.93	56.8	35.5	39.1	60.2	1.20	0.82	0.85
1.93	1.84	75.3	60.3	77.9	89.6	1.58	1.39	1.68
0.83	0.88	36.2	42.0	32.6	41.6	0.77	0.98	0.71
0.94	0.77	37.4	59.0	49.4	48.8	0.79	1.37	1.06
0.95	0.71	25.8	38.2	39.8	35.7	0.54	0.88	0.86
0.95	0.78	43.2	60.5	56.6	56.2	0.91	1.40	1.24
0.56	0.77	48.4	51.7	31.2	51.2	1.02	1.20	0.67
0.66	0.55	40.5	53.5	52.0	52.3	0.86	1.25	1.13
0.56	0.72	37.3	41.8	25.6	40.1	0.79	0.97	0.56
1.58	1.37	56.9	47.5	45.7	47.8	1.20	1.10	1.00
0.35	0.47	34.5	40.4	22.8	36.8	0.73	0.93	0.49
0.68	1.12	53.6	54.6	23.4	46.5	1.12	1.27	0.50
1.41	1.27	43.7	42.9	49.3	53.8	0.93	1.00	1.07
2.42	1.68	32.9	55.3	57.4	47.9	0.69	1.28	1.23
0.90	0.89	38.8	40.6	29.5	35.1	0.82	0.94	0.64
1.61	1.50	61.4	51.4	45.7	51.3	1.31	1.20	1.00
2.09	1.07	25.8	34.2	84.8	52.2	0.55	0.79	1.84
0.85	0.93	42.8	47.9	36.5	48.5	0.90	1.11	0.79
0.93	0.92	42.5	39.5	42.1	50.3	0.90	0.91	0.91
1.96	1.46	40.4	57.4	62.3	55.8	0.86	1.34	1.35
0.82	0.84	51.6	42.8	48.6	60.2	1.10	0.99	1.06
1.14	0.69	30.3	75.7	69.3	50.2	0.65	1.76	1.50
1.20	0.70	26.5	28.7	64.5	45.3	0.56	0.66	1.39
1.11	0.64	39.5	49.9	96.9	67.9	0.83	1.15	2.10
0.83	0.68	32.4	43.7	42.5	42.2	0.69	1.01	0.92
1.06	0.47	28.7	53.7	52.0	27.8	0.60	1.24	1.12
0.64	0.81	49.9	37.2	35.7	54.0	1.05	0.87	0.77
1.61	0.89	26.7	46.5	73.7	48.9	0.56	1.08	1.59
0.56	0.85	52.2	41.7	27.0	49.5	1.10	0.97	0.59
0.40	0.50	38.6	53.5	32.5	49.3	0.81	1.25	0.70
0.78	0.79	41.1	43.0	39.6	48.3	0.87	1.00	0.86
0.14	0.87	57.1	52.6	6.8	49.2	1.20	1.22	0.14
0.49	0.80	50.7	36.6	26.1	51.2	1.07	0.85	0.56
0.91	0.82	58.7	48.2	45.7	49.8	1.24	1.12	0.99
0.56	0.40	37.1	42.7	38.5	33.1	0.79	1.00	0.83
0.90	0.66	61.0	43.1	57.1	50.3	1.28	1.01	1.24
0.52	0.44	26.1	36.1	22.8	23.5	0.56	0.84	0.50
0.45	0.30	19.6	46.3	23.4	19.1	0.42	1.07	0.51

0.77	1.78	80.5	34.4	26.8	74.7	1.71	0.80	0.59
1.14	0.41	19.5	62.2	46.3	19.8	0.41	1.45	1.00
0.97	1.17	51.3	38.4	35.3	51.4	1.08	0.89	0.77
1.19	0.57	29.7	48.7	46.3	26.8	0.62	1.13	1.00
0.74	0.70	38.2	38.3	40.4	46.2	0.80	0.89	0.87
0.98	0.77	31.3	53.9	44.0	41.8	0.66	1.25	0.96
0.87	0.64	46.3	37.6	45.0	39.7	0.99	0.87	0.97
0.82	0.42	34.6	55.9	45.7	28.6	0.73	1.29	0.99
0.35	0.68	56.8	39.7	23.6	55.4	1.20	0.92	0.51
0.25	0.46	60.3	62.7	23.4	52.6	1.27	1.46	0.51
1.18	1.27	66.6	61.5	57.9	75.6	1.41	1.43	1.26
2.12	1.43	54.5	46.5	100.0	81.1	1.15	1.08	2.16
1.42	0.99	33.0	67.0	57.4	48.1	0.70	1.56	1.25
1.82	0.73	14.8	39.1	99.0	48.2	0.32	0.91	2.15
1.05	1.18	52.3	67.0	42.9	58.4	1.10	1.56	0.93
0.95	1.15	90.2	74.9	51.4	75.2	1.91	1.75	1.12
1.03	0.59	38.2	54.7	48.2	33.1	0.81	1.27	1.04
2.23	3.21	99.8	31.6	57.6	100.0	2.11	0.73	1.25
0.65	0.51	27.7	20.2	32.1	30.5	0.59	0.47	0.70
0.73	0.38	25.6	33.3	37.1	23.3	0.54	0.77	0.80
0.40	0.67	60.5	77.2	26.0	51.9	1.27	1.79	0.56
0.75	1.01	74.2	75.2	37.1	60.5	1.56	1.74	0.80
0.70	0.95	70.0	75.0	35.3	57.6	1.48	1.74	0.76
0.34	0.83	79.1	100.1	22.8	67.0	1.68	2.32	0.50
0.68	0.44	29.1	48.2	40.8	32.1	0.62	1.13	0.89
0.50	0.70	48.8	60.0	39.4	66.6	1.02	1.39	0.85
0.97	0.73	39.8	76.7	60.6	54.9	0.84	1.78	1.31
0.65	0.63	47.5	83.6	49.0	57.0	0.99	1.94	1.06
0.42	0.64	51.8	79.7	26.6	48.8	1.09	1.85	0.57
1.02	1.64	67.3	20.1	28.6	55.3	1.41	0.46	0.61
0.57	0.46	23.4	48.3	32.1	31.0	0.50	1.11	0.69
0.88	0.53	39.2	51.5	52.5	38.3	0.83	1.19	1.14
1.11	0.80	35.5	55.5	58.0	50.4	0.75	1.29	1.25
2.09	2.03	68.3	49.7	68.9	80.5	1.43	1.14	1.48
1.07	0.49	15.2	43.8	66.3	36.7	0.32	1.02	1.43
1.11	0.58	25.1	59.1	77.0	48.7	0.53	1.38	1.67
0.91	0.78	44.8	34.5	55.0	56.9	0.94	0.80	1.19
0.94	0.55	28.4	49.5	69.4	48.7	0.59	1.15	1.50
0.44	0.68	53.9	77.8	29.8	55.3	1.14	1.81	0.64
0.51	0.70	66.8	82.2	34.2	56.2	1.41	1.90	0.73
0.36	0.46	33.4	10.1	22.5	34.3	0.70	0.23	0.48
0.58	0.16	11.3	4.4	32.5	10.9	0.24	0.10	0.70
0.39	0.66	70.9	48.2	28.9	59.3	1.50	1.11	0.63
0.98	1.04	54.1	66.5	48.8	62.1	1.14	1.54	1.06
0.48	0.71	45.7	45.3	26.4	47.2	0.96	1.05	0.57
1.03	0.69	26.8	49.6	28.6	23.0	0.57	1.15	0.62
1.24	0.87	38.7	38.3	66.4	56.1	0.81	0.88	1.44

1.39	0.70	23.5	63.1	79.2	48.2	0.50	1.46	1.72
0.97	1.03	62.3	75.2	55.8	71.5	1.31	1.75	1.21
0.97	0.70	56.5	66.9	91.6	80.0	1.19	1.56	1.99
0.56	0.78	54.8	42.8	33.7	57.4	1.15	1.00	0.73
0.28	0.63	92.7	50.9	28.5	77.2	1.95	1.18	0.61
1.19	0.76	39.7	46.4	80.5	62.0	0.83	1.07	1.74
1.26	0.37	18.4	55.1	45.7	16.0	0.39	1.27	0.98
0.42	0.50	34.3	40.5	26.5	37.9	0.72	0.94	0.57
0.77	0.91	53.3	49.3	40.9	58.7	1.12	1.14	0.89
0.66	0.95	66.1	74.7	39.7	68.7	1.39	1.73	0.86
1.16	1.09	50.0	46.5	53.4	60.5	1.06	1.08	1.16
0.78	0.67	42.7	51.0	49.4	48.9	0.68	1.15	0.87
0.82	0.51	41.9	52.9	79.3	57.2	0.67	1.19	1.38
0.67	0.48	25.3	29.9	38.8	31.9	0.40	0.68	0.68
1.36	0.84	36.4	23.4	70.3	50.0	0.58	0.52	1.22
0.43	0.46	37.2	30.9	31.6	39.3	0.59	0.69	0.55
1.35	1.14	53.0	23.5	61.9	60.4	0.85	0.53	1.08
0.56	0.64	47.2	48.5	37.5	49.0	0.75	1.09	0.65
1.71	1.07	42.7	65.1	79.9	57.7	0.68	1.47	1.39
0.68	0.51	37.9	26.3	53.4	46.3	0.60	0.59	0.93
0.80	0.46	29.8	35.3	65.8	43.4	0.47	0.80	1.14
0.69	0.63	41.6	38.3	43.8	46.4	0.66	0.86	0.76
0.76	0.44	31.7	63.6	70.2	46.3	0.50	1.42	1.22
0.40	0.25	16.1	26.4	30.0	21.9	0.26	0.60	0.52
0.70	0.58	39.3	32.3	48.4	45.9	0.62	0.72	0.84
0.50	0.33	20.8	26.5	35.2	27.2	0.33	0.59	0.62
0.95	0.67	42.9	39.9	66.1	54.0	0.68	0.90	1.15
0.65	0.38	19.1	38.2	39.8	27.2	0.31	0.86	0.69
1.88	1.43	47.6	33.1	64.9	56.8	0.76	0.74	1.13
0.87	0.67	35.4	46.7	48.2	42.7	0.56	1.05	0.84
1.07	0.68	34.2	57.3	62.2	45.8	0.55	1.29	1.08
0.87	1.17	77.7	24.6	48.9	75.8	1.24	0.56	0.85
3.26	2.84	92.3	20.8	99.7	100.0	1.46	0.47	1.73
0.85	1.02	58.6	40.0	42.6	59.1	0.93	0.90	0.74
1.45	1.47	65.2	33.7	58.7	68.6	1.04	0.76	1.02
0.63	0.72	42.3	39.8	32.9	43.5	0.67	0.89	0.57
1.37	1.91	91.4	37.1	53.8	86.5	1.44	0.83	0.93
0.78	0.88	63.2	53.4	50.6	65.3	1.00	1.20	0.87
1.88	1.21	56.1	62.4	99.7	74.1	0.89	1.40	1.73
0.96	1.11	57.8	45.4	44.4	58.9	0.92	1.02	0.77
2.42	1.81	57.1	32.3	78.8	68.1	0.91	0.73	1.38
0.60	0.86	73.5	50.2	43.1	71.3	1.18	1.13	0.75
1.25	1.65	75.9	53.5	47.9	73.3	1.21	1.21	0.84
0.70	1.07	64.8	21.7	34.8	61.5	1.02	0.49	0.60
1.31	1.85	82.0	31.4	47.3	77.4	1.30	0.71	0.83
0.80	0.89	46.9	22.5	37.5	48.5	0.75	0.51	0.66
1.10	0.82	38.3	50.7	54.6	46.8	0.61	1.14	0.95

0.86	0.45	15.2	33.5	40.4	24.1	0.24	0.75	0.70
1.73	1.33	38.7	25.3	51.5	45.8	0.62	0.57	0.90
0.78	0.86	38.5	49.9	31.6	40.1	0.62	1.13	0.55
1.55	1.26	52.7	50.4	64.9	61.0	0.84	1.14	1.13
0.69	0.76	45.2	24.7	36.8	47.0	0.73	0.56	0.64
2.23	1.25	28.6	13.7	64.2	41.7	0.46	0.31	1.12
0.81	0.66	28.3	21.1	35.7	33.3	0.45	0.47	0.62
2.19	1.46	35.2	15.5	59.3	45.4	0.56	0.35	1.03
0.57	0.67	35.8	53.6	26.6	36.5	0.57	1.21	0.47
2.79	1.42	21.2	31.3	57.4	33.9	0.34	0.71	1.00
0.61	0.51	21.4	43.2	25.6	24.8	0.34	0.97	0.45
2.64	2.13	46.0	11.0	56.5	52.5	0.73	0.24	0.98
0.74	0.80	48.5	44.0	40.5	50.7	0.78	1.00	0.71
1.72	0.90	29.6	42.0	76.9	46.4	0.47	0.94	1.34
0.85	0.80	49.6	45.1	50.1	54.5	0.79	1.02	0.88
1.09	0.86	43.4	76.5	57.0	51.5	0.69	1.73	0.99
0.75	0.86	56.4	44.6	43.3	57.7	0.90	1.00	0.76
1.07	0.77	58.4	68.2	87.1	72.5	0.92	1.53	1.52
0.63	0.61	49.7	42.3	47.7	54.0	0.80	0.96	0.84
2.02	1.15	44.6	75.1	98.8	64.8	0.71	1.68	1.72
0.80	1.19	89.8	44.9	49.8	85.5	1.43	1.00	0.86
1.39	0.98	49.0	74.9	75.6	61.5	0.78	1.68	1.31
0.80	1.22	97.1	42.3	52.0	91.8	1.55	0.96	0.91
1.12	0.98	59.7	75.3	66.1	67.1	0.95	1.70	1.15
0.67	1.43	94.9	42.0	34.2	84.1	1.50	0.94	0.59
0.62	0.75	64.1	69.9	46.7	65.0	1.01	1.57	0.81
0.54	0.40	20.2	42.4	29.6	25.1	0.32	0.96	0.51
0.68	0.40	21.8	70.3	46.5	31.3	0.35	1.58	0.81
0.69	0.59	31.7	43.3	37.1	36.4	0.50	0.98	0.64
0.77	0.96	72.4	72.1	50.1	72.4	1.15	1.63	0.88
0.62	0.77	54.1	41.4	37.7	54.4	0.86	0.93	0.66
1.20	1.04	62.8	77.0	70.7	70.9	1.00	1.72	1.22
0.72	1.37	100.0	42.1	40.9	90.3	1.58	0.95	0.71
1.19	0.95	45.3	43.3	57.5	53.2	0.71	0.97	1.00
0.48	1.12	91.2	44.0	29.7	80.6	1.45	0.99	0.52
2.76	3.27	80.5	7.8	56.1	76.6	1.27	0.18	0.97
0.63	0.99	85.8	40.7	44.4	81.2	1.37	0.92	0.77
1.27	0.96	50.9	72.5	70.3	61.4	0.81	1.62	1.22
0.82	1.01	73.2	40.8	50.9	73.2	1.17	0.92	0.89
0.70	0.50	31.2	72.9	47.2	39.1	0.49	1.64	0.82
0.55	0.82	61.5	41.1	34.2	59.2	0.98	0.93	0.60
0.97	0.83	52.2	70.2	60.5	59.6	0.83	1.58	1.06
1.34	1.73	83.5	40.7	54.2	80.9	1.33	0.92	0.95
0.97	0.79	49.7	68.4	62.4	58.3	0.80	1.55	1.09
0.88	0.60	24.5	43.1	39.7	31.4	0.40	0.98	0.70
0.77	0.45	24.0	74.7	49.8	34.0	0.38	1.68	0.87
0.89	0.66	34.2	39.9	49.3	42.0	0.55	0.90	0.85

1.13	0.95	57.2	69.1	67.9	65.7	0.90	1.55	1.18
0.57	0.72	53.1	42.2	36.1	53.1	0.84	0.95	0.63
1.36	1.08	50.9	69.6	65.2	59.8	0.81	1.55	1.13
0.78	0.74	43.7	43.0	44.2	48.1	0.70	0.97	0.76
1.46	1.13	45.9	44.8	60.4	54.4	0.73	1.00	1.05
1.24	1.07	46.0	44.5	52.5	52.1	0.73	0.99	0.91
0.97	0.97	89.0	62.3	82.5	95.2	1.42	1.41	1.44
1.21	1.45	71.5	42.9	51.9	71.4	1.13	0.96	0.90
1.42	1.10	53.2	59.9	70.2	63.0	0.84	1.34	1.22
0.53	0.46	26.0	50.3	29.7	29.8	0.41	1.13	0.51
1.19	0.82	38.9	72.9	62.0	49.5	0.62	1.64	1.08
0.70	1.13	77.9	41.0	39.4	73.1	1.23	0.92	0.68
1.02	0.80	52.6	71.7	69.6	62.7	0.84	1.60	1.20
0.44	0.49	46.2	54.6	37.1	48.2	0.74	1.23	0.65
1.54	1.05	46.8	74.3	76.1	59.8	0.74	1.67	1.32
0.56	0.60	53.8	52.2	46.3	56.9	0.86	1.17	0.80
0.95	0.74	45.3	72.6	60.2	54.1	0.72	1.63	1.05
0.52	0.56	44.4	54.5	38.1	47.0	0.71	1.23	0.66
0.97	0.80	45.7	79.0	56.1	53.1	0.73	1.79	0.98
0.48	0.63	53.8	55.7	35.1	53.5	0.85	1.24	0.61
0.77	0.79	51.5	72.6	46.6	54.9	0.82	1.63	0.81
0.62	0.59	49.5	40.5	50.3	54.6	0.79	0.92	0.87
0.80	0.65	43.3	73.5	53.9	50.7	0.68	1.65	0.94
0.44	0.57	49.9	53.3	32.9	49.8	0.80	1.21	0.58
1.25	0.85	38.2	69.3	62.3	49.0	0.61	1.57	1.09
0.48	0.78	66.5	37.5	33.1	62.8	1.05	0.85	0.58
0.50	0.56	49.9	69.2	40.8	52.1	0.79	1.55	0.71
0.51	0.82	55.4	42.0	28.1	52.4	0.88	0.95	0.49
0.71	0.79	60.5	73.7	48.9	62.8	0.96	1.66	0.85
0.36	0.50	42.7	48.3	26.2	42.1	0.69	1.09	0.46
0.68	0.70	55.5	96.1	49.6	59.1	0.88	2.15	0.86
1.02	1.03	49.4	52.8	44.7	52.5	0.79	1.19	0.79
0.65	0.74	50.2	69.0	39.7	51.9	0.80	1.56	0.69
0.71	0.92	58.3	42.1	38.5	57.8	0.93	0.95	0.67
1.32	1.02	41.6	51.0	55.7	49.7	0.67	1.15	0.98
0.41	0.42	32.1	52.0	29.5	34.7	0.51	1.17	0.51
1.36	1.01	41.8	58.3	59.5	50.9	0.66	1.31	1.03
0.52	0.37	27.2	49.4	41.5	34.2	0.43	1.12	0.72
1.78	1.02	35.7	36.8	78.5	51.7	0.57	0.83	1.37
0.98	0.95	45.8	53.0	44.4	49.6	0.73	1.19	0.77
1.23	0.82	38.7	69.2	64.7	50.1	0.62	1.56	1.13
0.52	0.75	62.1	51.3	36.9	60.5	0.99	1.15	0.63
1.22	0.70	36.0	75.4	78.1	52.0	0.57	1.70	1.37
0.73	0.70	41.8	51.7	41.9	45.9	0.66	1.16	0.72
1.02	0.56	27.1	71.0	64.2	40.7	0.43	1.60	1.12
0.64	0.56	40.1	50.2	45.7	45.7	0.64	1.12	0.79
1.88	1.20	44.8	38.1	81.5	59.8	0.71	0.86	1.41

0.63	0.62	42.8	50.3	40.9	46.5	0.68	1.13	0.71
1.18	1.10	52.2	67.4	53.0	57.1	0.83	1.51	0.92
0.67	0.82	68.7	52.2	49.2	69.4	1.09	1.17	0.85
1.04	0.94	51.4	67.0	54.6	57.1	0.81	1.50	0.95
0.71	0.97	70.2	50.7	43.1	68.4	1.11	1.14	0.75
0.64	0.53	45.7	97.4	55.9	53.3	0.73	2.19	0.97
0.73	0.93	74.6	51.2	50.3	74.2	1.19	1.15	0.88
0.56	0.58	50.3	100.0	44.6	53.6	0.80	2.25	0.78
0.73	0.59	40.8	58.2	52.1	48.2	0.65	1.30	0.90
1.09	0.65	34.3	46.9	69.9	48.2	0.54	1.05	1.21
0.66	0.86	71.3	51.0	46.5	70.6	1.14	1.15	0.82
1.43	0.95	44.7	60.9	75.0	57.9	0.72	1.38	1.32
0.74	0.73	53.3	49.2	50.3	57.5	0.85	1.11	0.87
1.20	0.86	51.1	61.6	76.5	63.5	0.81	1.39	1.33
1.51	1.20	54.8	66.4	73.7	67.6	1.25	0.93	1.72
1.00	0.78	34.5	58.4	48.2	43.3	0.79	0.81	1.13
1.26	0.94	36.3	73.9	55.7	47.4	0.83	1.04	1.30
0.90	0.75	34.1	70.6	42.4	40.7	0.78	0.99	0.99
1.43	0.90	28.0	75.2	59.2	42.8	0.64	1.06	1.37
1.00	0.73	29.6	68.3	46.9	39.2	0.68	0.96	1.10
1.22	0.88	32.6	72.0	52.9	43.7	0.75	1.01	1.23
0.75	0.71	37.2	78.4	37.3	41.0	0.85	1.10	0.88
0.86	0.99	57.7	95.8	43.9	58.2	1.32	1.34	1.03
0.83	0.85	46.8	85.5	42.4	49.8	1.07	1.20	0.99
0.96	0.97	49.5	84.7	45.4	52.9	1.13	1.19	1.06
0.83	0.87	44.1	85.4	40.9	49.2	1.00	1.20	0.95
1.36	0.95	38.9	82.4	58.8	47.3	0.89	1.15	1.37
1.29	1.25	47.6	67.8	51.3	57.2	1.09	0.95	1.20
1.48	1.05	40.4	85.4	68.0	55.1	0.93	1.20	1.58
1.46	0.86	27.1	85.0	66.1	44.9	0.62	1.19	1.53
1.24	1.40	67.7	80.2	52.8	68.6	1.54	1.12	1.23
0.98	1.03	48.2	78.0	42.0	50.6	1.10	1.09	0.98
0.90	1.74	100.0	78.8	39.0	86.1	2.28	1.10	0.90
0.81	1.21	62.7	70.8	33.8	57.7	1.44	0.99	0.79
0.90	1.44	82.6	82.9	40.2	74.3	1.88	1.15	0.94
0.69	1.29	76.0	77.9	30.9	66.1	1.74	1.09	0.72
1.51	1.90	91.2	81.5	61.5	88.5	2.09	1.15	1.43
0.74	1.10	47.8	81.0	33.1	56.9	1.09	1.13	0.78
1.67	1.61	75.2	86.9	74.3	82.3	1.72	1.22	1.72
0.98	1.26	72.9	87.7	47.8	70.4	1.68	1.23	1.12
1.85	1.37	52.7	81.0	81.1	68.9	1.20	1.13	1.89
1.42	1.49	61.3	59.3	53.1	64.2	1.40	0.83	1.24
1.21	0.94	32.1	90.1	45.6	40.5	0.74	1.27	1.06
0.52	0.83	33.4	81.1	22.6	41.8	0.77	1.13	0.53
1.29	1.12	51.1	91.6	59.4	59.4	1.17	1.27	1.38
1.00	1.03	54.7	88.8	49.2	58.1	1.24	1.24	1.14
0.92	1.07	55.6	81.2	41.7	55.8	1.27	1.13	0.97

0.22	0.83	63.6	59.7	10.9	47.9	1.45	0.83	0.26
1.04	1.25	80.3	94.7	57.7	79.6	1.83	1.33	1.34
1.15	1.07	52.1	73.4	54.6	58.3	1.20	1.03	1.28
1.23	0.94	38.9	83.4	55.7	49.3	0.89	1.17	1.30
0.98	0.86	43.2	80.2	48.9	49.8	0.99	1.12	1.15
1.22	1.00	40.7	82.5	52.2	49.2	0.93	1.15	1.22
0.35	0.89	63.9	77.0	15.6	46.3	1.46	1.07	0.37
1.64	0.91	28.0	92.1	78.7	50.3	0.64	1.29	1.84
0.68	0.75	46.9	92.5	37.9	48.2	1.08	1.30	0.88
1.15	1.08	47.5	82.2	48.8	52.8	1.09	1.15	1.14
0.69	0.88	60.7	81.3	31.2	45.5	1.40	1.14	0.72
1.53	1.26	49.4	87.5	62.5	59.4	1.12	1.22	1.45
1.19	1.02	40.5	77.2	48.4	47.6	0.93	1.08	1.13
1.42	1.07	37.3	85.9	55.8	48.2	0.85	1.19	1.29
0.55	0.42	27.1	85.8	23.4	20.3	0.62	1.21	0.54
1.30	1.21	52.8	68.1	54.8	58.8	1.21	0.95	1.27
1.11	1.14	49.9	64.9	45.1	53.0	1.14	0.90	1.04
1.09	1.05	49.9	83.7	49.3	54.6	1.14	1.18	1.16
1.29	1.06	41.4	61.7	53.1	50.1	0.95	0.86	1.24
1.12	0.98	40.5	73.2	46.4	46.9	0.93	1.03	1.09
1.12	0.61	15.6	61.8	45.5	28.7	0.36	0.86	1.06
0.93	0.71	27.8	76.5	39.6	35.1	0.64	1.08	0.93
0.92	0.74	27.9	70.9	37.2	34.3	0.64	1.00	0.86
1.21	1.02	44.1	84.0	54.5	52.5	1.01	1.18	1.26
0.99	0.77	31.1	76.2	43.4	39.0	0.71	1.07	1.01
1.18	1.13	50.7	75.1	50.2	55.6	1.16	1.05	1.17
0.96	0.92	41.2	70.2	41.1	45.3	0.94	0.98	0.95
1.57	1.00	29.9	67.4	61.5	45.1	0.69	0.95	1.44
1.05	0.63	19.3	65.6	45.5	31.3	0.44	0.92	1.06
1.51	1.34	69.4	99.8	77.8	79.6	1.58	1.39	1.81
0.67	0.97	56.8	89.2	31.9	52.9	1.30	1.25	0.74
0.87	1.54	86.8	86.7	37.5	76.2	1.98	1.21	0.87
0.70	1.51	86.1	71.1	29.0	72.5	1.97	1.00	0.68
1.41	1.49	69.1	70.6	59.3	72.2	1.58	0.99	1.38
1.07	1.42	74.9	70.5	46.7	71.4	1.71	0.99	1.09
1.86	1.12	34.0	72.0	79.0	54.7	0.78	1.01	1.84
1.14	0.86	16.9	69.5	46.1	39.8	0.39	0.97	1.07
1.50	1.28	54.3	81.6	65.2	64.0	1.24	1.14	1.52
0.83	1.08	36.8	73.3	32.8	49.0	0.84	1.02	0.76
1.86	1.34	44.9	90.8	72.6	60.0	1.03	1.27	1.69
0.88	1.03	44.1	75.6	43.5	58.4	1.01	1.06	1.01
1.40	1.16	49.4	82.9	62.4	59.4	1.13	1.16	1.46
0.75	1.21	52.2	78.5	32.0	59.6	1.20	1.10	0.75
1.37	1.03	36.2	75.3	54.7	46.9	0.83	1.06	1.27
0.98	0.76	16.2	73.4	37.0	33.0	0.37	1.03	0.86
1.85	1.07	33.8	86.2	85.2	57.0	0.78	1.21	2.00
0.63	0.96	54.5	76.4	28.6	50.0	1.24	1.06	0.66

0.75	0.98	58.9	82.0	37.8	56.7	1.35	1.15	0.88
0.24	1.04	71.6	66.7	10.7	52.5	1.63	0.94	0.24
1.24	1.15	50.6	77.1	53.6	56.9	1.16	1.08	1.25
0.97	0.93	46.3	75.4	45.6	50.7	1.06	1.06	1.07
0.87	1.05	73.3	98.3	42.2	58.8	1.67	1.37	0.98
0.41	0.71	63.7	93.8	23.4	46.5	1.45	1.30	0.55
1.57	0.97	32.5	92.5	72.2	51.1	0.75	1.29	1.68
0.90	0.96	42.0	80.9	42.2	51.7	0.97	1.13	0.99
1.30	0.79	27.1	94.6	61.6	43.1	0.62	1.33	1.44
0.82	0.76	34.9	80.1	37.0	39.3	0.80	1.12	0.86
1.21	0.91	38.3	97.8	58.0	49.8	0.88	1.37	1.34
0.43	0.72	53.4	91.8	25.1	48.0	1.22	1.28	0.58
1.90	1.04	26.5	86.3	77.6	48.9	0.61	1.21	1.81
1.34	1.06	39.1	74.7	53.3	48.5	0.89	1.04	1.25
1.78	1.95	97.7	94.0	79.1	100.0	2.25	1.32	1.85
0.70	0.41	27.3	82.5	31.2	20.8	0.62	1.15	0.73
1.24	0.97	36.5	55.9	51.0	45.8	0.84	0.79	1.19
0.88	0.79	25.3	50.7	36.2	37.5	0.58	0.71	0.84
1.37	1.21	54.7	76.4	62.3	63.1	1.25	1.07	1.45
0.84	0.94	32.3	60.6	34.4	44.4	0.74	0.85	0.81
1.15	0.70	20.9	72.1	47.6	33.3	0.48	1.01	1.10
0.97	0.68	16.2	62.2	35.7	28.9	0.37	0.88	0.83
1.28	1.22	53.2	72.1	53.5	58.6	1.21	1.01	1.24
0.98	0.86	38.5	76.1	44.7	44.8	0.88	1.07	1.04
2.30	1.12	24.1	77.9	100.1	55.8	0.56	1.09	2.32
0.96	0.67	25.9	77.4	45.2	36.0	0.59	1.08	1.05
1.30	1.05	43.1	78.8	56.1	52.5	0.99	1.10	1.31
0.95	0.60	21.4	78.6	45.2	32.7	0.48	1.10	1.05
1.32	0.94	33.4	74.3	55.5	45.3	0.76	1.04	1.29
0.69	0.72	19.0	72.8	29.2	34.8	0.43	1.02	0.68
1.87	1.13	36.1	85.2	84.0	58.2	0.83	1.20	1.96
1.69	1.19	41.5	66.3	69.7	56.5	0.95	0.92	1.62
1.99	1.86	83.5	72.5	86.3	92.7	1.91	1.02	2.01
1.25	1.64	85.9	62.5	54.7	82.2	1.97	0.88	1.28
1.24	1.25	58.7	78.4	54.6	63.0	1.34	1.09	1.26
1.14	1.28	61.1	69.4	48.4	62.2	1.40	0.97	1.13
0.93	1.57	87.9	72.8	39.9	77.9	2.02	1.02	0.94
0.81	1.32	74.1	67.9	35.4	66.4	1.69	0.95	0.83
2.19	1.89	78.3	78.0	92.0	91.2	1.79	1.10	2.15
1.56	1.12	46.7	81.7	75.3	62.4	1.07	1.14	1.76
1.42	1.75	89.9	82.7	62.0	87.8	2.06	1.16	1.45
1.36	1.39	63.0	70.0	56.7	66.8	1.44	0.98	1.32
1.72	1.45	59.8	84.2	73.7	71.1	1.37	1.18	1.72
0.96	1.18	46.2	77.0	43.7	61.8	1.06	1.08	1.02
1.40	0.88	26.5	77.0	55.7	40.4	0.60	1.08	1.30
0.76	0.63	28.5	77.4	35.8	34.2	0.65	1.09	0.84
1.27	0.99	36.0	80.7	50.2	45.1	0.82	1.13	1.17

1.02	0.86	33.6	80.1	41.2	40.0	0.77	1.12	0.96
0.62	1.29	88.0	80.3	31.6	74.9	2.02	1.12	0.73
0.39	1.07	80.2	77.7	18.5	58.9	1.85	1.09	0.44
1.55	1.56	74.6	80.4	68.6	79.6	1.70	1.12	1.60
1.36	1.19	59.1	77.3	68.2	68.6	1.36	1.09	1.59
0.95	1.31	76.5	77.7	45.3	72.0	1.75	1.09	1.05
0.60	1.11	58.3	74.9	27.6	58.3	1.33	1.05	0.64
1.61	1.39	67.8	80.7	79.8	79.2	1.55	1.14	1.87
1.26	1.12	56.5	79.3	63.9	65.0	1.30	1.11	1.49
1.09	1.08	50.8	90.6	48.3	55.0	1.16	1.27	1.12
0.79	0.62	31.8	89.3	43.5	39.6	0.74	1.25	1.02
1.10	1.30	63.1	86.2	46.5	62.9	1.44	1.21	1.08
0.46	0.87	62.0	84.7	20.8	45.1	1.42	1.19	0.48
1.50	1.05	37.5	83.4	63.2	51.2	0.85	1.17	1.47
0.41	0.71	50.0	81.6	18.2	36.5	1.14	1.14	0.43
1.47	1.08	39.1	82.6	61.6	51.7	0.89	1.15	1.43
0.56	0.92	41.1	83.5	26.3	49.6	0.94	1.17	0.61

	NPI		MBCCLI		ACCLI		CWCCLI	
Std Dev	Mean	Std Dev	Mean	Std Dev	Mean	Std Dev	Mean	Std Dev
0.28	1.14	0.4	1.0	0.5	1.0	0.31	0.97	0.49
0.45	1.03	0.3	1.4	1.1	1.2	0.54	1.35	0.84
0.08	1.01	0.1	1.2	0.4	1.1	0.11	1.35	0.34
0.10	1.02	0.1	1.0	0.4	1.0	0.10	0.90	0.31
0.21	1.00	0.2	1.5	0.7	1.0	0.13	1.03	0.39
0.31	0.92	0.2	1.4	1.0	1.0	0.20	1.10	0.54
0.20	1.02	0.2	0.8	0.4	0.9	0.25	0.69	0.19
0.30	0.86	0.2	0.9	0.5	1.3	0.39	1.29	0.57

	NPI		MBCCLI		ACCLI		CWCCLI	
Std Dev	Mean	Std Dev	Mean	Std Dev	Mean	Std Dev	Mean	Std Dev
0.21	1.03	0.2	0.7	0.3	1.0	0.30	0.88	0.40
0.20	0.96	0.2	0.9	0.4	1.1	0.27	1.01	0.41
0.26	0.96	0.3	1.4	0.8	1.0	0.35	1.15	0.53
0.26	1.04	0.1	1.0	0.5	1.1	0.29	1.01	0.46
0.26	0.93	0.2	1.1	0.7	1.1	0.28	1.08	0.47
0.27	0.95	0.2	1.4	0.9	1.1	0.41	1.34	0.74
0.25	1.05	0.2	1.2	0.6	1.2	0.29	0.99	0.39
0.25	1.04	0.3	1.3	0.7	1.1	0.35	1.02	0.49
0.28	1.03	0.3	1.5	1.0	1.1	0.31	1.31	0.69

	NPI		MBCCLI		ACCLI		CWCCLI	
Std Dev	Mean	Std Dev	Mean	Std Dev	Mean	Std Dev	Mean	Std Dev
0.24	0.99	0.2	1.0	0.6	1.0	0.26	1.01	0.47
0.25	0.98	0.2	0.9	0.6	1.1	0.35	1.02	0.45
0.30	0.98	0.2	1.2	0.7	1.0	0.29	1.10	0.51
0.27	0.97	0.2	1.2	0.9	1.1	0.37	1.19	0.65
0.26	0.98	0.2	1.4	0.8	1.1	0.28	1.08	0.45
0.29	1.10	0.4	1.3	0.8	1.2	0.34	1.13	0.64

	NPI		MBCCLI		ACCLI		CWCCLI	
Std Dev	Mean	Std Dev	Mean	Std Dev	Mean	Std Dev	Mean	Std Dev
0.19	1.02	0.2	0.9	0.5	1.0	0.24	0.98	0.38
0.29	0.95	0.2	1.0	0.7	1.1	0.36	1.05	0.53
0.20	1.02	0.2	1.1	0.5	1.0	0.19	1.00	0.40
0.34	0.93	0.2	1.3	1.0	1.1	0.42	1.29	0.69
0.22	1.09	0.3	1.3	0.7	1.1	0.23	1.06	0.51
0.32	0.99	0.2	1.3	0.8	1.2	0.37	1.15	0.60

	NPI		MBCCLI		ACCLI		CWCCLI	
Std Dev	Mean	Std Dev	Mean	Std Dev	Mean	Std Dev	Mean	Std Dev

0.26	1.03	0.2	1.0	0.5	1.1	0.31	0.98	0.44
0.22	1.05	0.2	0.9	0.5	1.1	0.29	0.94	0.40
0.23	0.95	0.2	1.1	0.6	1.1	0.26	1.02	0.40
0.25	1.01	0.3	1.1	0.6	1.1	0.34	1.05	0.50
0.26	0.97	0.2	1.4	0.8	1.0	0.27	1.19	0.56
0.29	0.99	0.3	1.4	1.0	1.1	0.43	1.34	0.74

	NPI		MBCCLI		ACCLI		CWCCLI	
Std Dev	Mean	Std Dev	Mean	Std Dev	Mean	Std Dev	Mean	Std Dev
0.18	1.09	0.2	1.0	0.5	1.0	0.25	0.92	0.42
0.28	0.99	0.2	1.0	0.6	1.1	0.34	1.00	0.41
0.18	1.01	0.3	1.1	0.5	1.0	0.18	1.01	0.41
0.29	0.95	0.2	1.1	0.7	1.2	0.37	1.06	0.50
0.19	1.03	0.3	1.3	0.7	1.0	0.25	1.11	0.45
0.33	0.93	0.3	1.6	1.0	1.1	0.44	1.42	0.79

	NPI		MBCCLI		ACCLI		CWCCLI	
Std Dev	Mean	Std Dev	Mean	Std Dev	Mean	Std Dev	Mean	Std Dev
0.20	1.00	0.2	1.2	0.6	1.0	0.23	1.00	0.40
0.31	0.96	0.2	1.2	0.8	1.1	0.32	1.13	0.53
0.20	1.08	0.3	1.1	0.6	1.0	0.22	1.03	0.46
0.32	0.95	0.2	1.2	0.9	1.2	0.43	1.19	0.69

CPI		NPI		MBCCLI		ACCLI		CWCCLI
Mean	Std Dev	Mean	Std Dev	Mean	Std Dev	Mean	Std Dev	Mean
1.11	0.21	1.0	0.2	0.7	0.3	0.97	0.33	0.93
1.15	0.21	1.0	0.1	0.7	0.3	1.01	0.27	0.83
1.05	0.20	1.0	0.2	0.8	0.4	1.03	0.19	0.88
0.93	0.19	0.9	0.1	0.9	0.4	1.15	0.32	1.14
0.85	0.23	1.0	0.2	1.5	0.7	0.99	0.24	1.21
0.95	0.28	1.0	0.3	1.2	0.8	1.00	0.44	1.09
1.14	0.27	1.0	0.1	1.1	0.4	1.06	0.30	0.97
1.12	0.25	1.0	0.1	0.9	0.6	1.12	0.29	1.05
0.99	0.30	0.9	0.2	1.2	0.8	1.09	0.30	1.12
1.02	0.22	1.0	0.1	1.0	0.6	1.02	0.27	1.04
0.94	0.28	1.0	0.2	1.2	0.6	0.95	0.26	1.20
0.82	0.26	0.9	0.2	1.6	1.1	1.18	0.50	1.47
1.11	0.29	1.0	0.2	1.2	0.6	1.14	0.30	1.04
1.12	0.22	1.1	0.3	1.2	0.5	1.20	0.29	0.94
0.99	0.18	1.0	0.2	1.3	0.5	1.12	0.28	1.05
1.07	0.31	1.1	0.4	1.2	0.8	1.13	0.40	0.98
0.91	0.26	1.0	0.2	1.5	1.0	1.12	0.28	1.15

0.94	0.30	1.1	0.4	1.5	1.0	1.13	0.34	1.46

CPI		NPI		MBCCLI		ACCLI		CWCCLI
Mean	Std Dev	Mean	Std Dev	Mean	Std Dev	Mean	Std Dev	Mean
1.14	0.17	1.1	0.1	0.7	0.3	0.94	0.27	0.87
1.12	0.25	1.0	0.2	0.7	0.3	1.04	0.32	0.89
0.96	0.16	1.0	0.1	0.9	0.3	1.03	0.15	1.01
1.02	0.24	0.9	0.2	0.8	0.4	1.15	0.34	1.00
0.94	0.18	1.0	0.3	1.2	0.6	0.94	0.28	1.06
0.85	0.32	0.9	0.3	1.5	0.9	1.04	0.41	1.25
1.18	0.16	1.1	0.1	0.9	0.4	0.99	0.17	0.90
1.08	0.33	1.0	0.1	1.0	0.7	1.18	0.35	1.12
1.01	0.18	1.0	0.1	1.1	0.4	0.99	0.19	1.03
0.99	0.33	0.9	0.2	1.2	0.9	1.11	0.35	1.13
0.95	0.19	1.0	0.2	1.1	0.6	1.00	0.22	1.07
0.81	0.32	0.9	0.2	1.6	1.1	1.13	0.53	1.60
1.14	0.22	1.1	0.3	1.2	0.5	1.13	0.25	0.98
1.09	0.28	1.0	0.2	1.2	0.6	1.21	0.33	1.00
1.02	0.18	1.1	0.4	1.3	0.6	1.01	0.19	0.99
1.04	0.31	1.0	0.2	1.2	0.7	1.24	0.42	1.04
0.94	0.21	1.1	0.3	1.5	0.9	1.06	0.24	1.21
0.91	0.34	1.0	0.3	1.5	1.1	1.19	0.35	1.41

CPI		NPI		MBCCLI		ACCLI		CWCCLI
Mean	Std Dev	Mean	Std Dev	Mean	Std Dev	Mean	Std Dev	Mean
1.00	0.18	1.0	0.2	1.0	0.5	0.98	0.25	0.95
1.00	0.29	1.0	0.2	1.0	0.7	1.02	0.27	1.07
1.02	0.20	1.0	0.2	0.9	0.5	0.97	0.24	1.01
1.00	0.29	0.9	0.2	1.0	0.7	1.14	0.42	1.03
1.05	0.22	1.0	0.2	1.1	0.5	0.98	0.20	0.97
0.99	0.36	0.9	0.2	1.2	0.7	1.08	0.35	1.23
1.04	0.18	1.0	0.1	1.0	0.5	1.01	0.18	1.04
0.93	0.33	0.9	0.2	1.3	1.1	1.20	0.47	1.34
1.00	0.21	1.0	0.2	1.4	0.7	1.08	0.23	1.07
1.01	0.30	1.0	0.2	1.3	0.9	1.18	0.33	1.09
1.07	0.23	1.2	0.4	1.3	0.7	1.06	0.24	1.04
1.01	0.34	1.0	0.3	1.3	0.8	1.25	0.41	1.21

CPI		NPI		MBCCLI		ACCLI		CWCCLI
Mean	Std Dev	Mean	Std Dev	Mean	Std Dev	Mean	Std Dev	Mean
1.13	0.18	1.1	0.1	1.0	0.5	1.02	0.27	0.92

1.10	0.32	1.0	0.2	1.0	0.6	1.10	0.35	1.04
1.17	0.19	1.1	0.2	0.9	0.4	1.02	0.22	0.91
1.09	0.25	1.0	0.2	0.9	0.6	1.19	0.32	0.97
0.98	0.18	1.0	0.2	1.2	0.5	1.02	0.17	0.99
1.04	0.28	0.9	0.2	1.0	0.7	1.13	0.32	1.04
1.02	0.17	1.1	0.3	1.0	0.5	1.00	0.18	1.03
0.99	0.31	1.0	0.2	1.1	0.7	1.20	0.42	1.08
0.94	0.21	1.0	0.2	1.3	0.7	0.99	0.24	1.07
0.85	0.29	0.9	0.2	1.5	0.9	1.05	0.29	1.31
0.95	0.18	1.1	0.3	1.3	0.7	1.01	0.26	1.15
0.86	0.36	0.9	0.3	1.6	1.2	1.19	0.54	1.53

HWC		CPI		NPI		MBCCLI		ACCLI
Mean	Std Dev	Mean	Std Dev	Mean	Std Dev	Mean	Std Dev	Mean
36.38	10.03	1.1	0.2	1.0	0.2	0.61	0.18	0.91
17.60	8.51	1.2	0.0	0.9	0.1	0.38	0.19	0.74
29.53	3.05	1.3	0.1	1.1	0.1	0.45	0.14	0.69
23.83	9.08	1.4	0.3	1.0	0.0	0.60	0.27	0.86
37.25	13.15	1.1	0.2	1.1	0.1	0.78	0.14	0.93
20.23	7.29	1.2	0.1	1.1	0.4	0.69	0.36	1.17
33.70	2.57	1.0	0.2	1.1	0.1	0.86	0.22	1.00
18.73	5.96	0.9	0.3	0.9	0.1	1.30	0.83	1.48
44.23	10.53	0.8	0.2	1.1	0.4	1.25	0.22	0.99
33.90	9.28	0.6	0.2	1.1	0.4	2.46	0.87	1.25
42.48	8.96	0.9	0.2	1.3	0.6	1.07	0.39	0.82
24.33	7.03	0.7	0.4	1.0	0.2	2.27	1.20	1.34
49.40	10.83	1.2	0.1	1.0	0.1	0.92	0.28	1.03
31.50	13.62	1.1	0.7	1.0	0.1	1.25	0.53	1.28
44.48	10.96	1.3	0.2	1.1	0.2	0.68	0.10	0.98
26.63	16.41	1.1	0.6	1.0	0.2	1.31	1.64	1.21
40.68	9.58	1.0	0.4	0.8	0.2	1.13	0.79	0.96
28.25	12.77	1.2	0.5	1.0	0.1	0.98	0.59	1.07
43.68	2.85	1.1	0.2	1.1	0.1	0.76	0.12	0.84
23.13	2.13	1.2	0.4	1.0	0.1	0.68	0.21	1.07
46.65	10.52	0.9	0.3	1.1	0.4	1.13	0.55	0.89
26.28	2.82	0.7	0.4	1.1	0.4	1.64	0.66	1.12
43.88	11.65	0.8	0.1	1.0	0.2	1.23	0.22	0.94
29.78	7.95	0.4	0.3	0.8	0.2	3.71	2.06	2.00
51.05	7.95	1.3	0.3	1.1	0.3	1.01	0.19	1.30
32.88	5.56	1.2	0.6	1.0	0.2	1.40	0.76	1.11
50.10	6.19	1.3	0.2	1.4	0.5	1.03	0.25	1.36
29.50	2.15	1.2	0.2	1.1	0.3	1.16	0.21	1.41
45.35	13.64	0.9	0.2	0.8	0.2	1.18	0.73	0.93
21.90	8.32	1.0	0.3	0.9	0.3	0.89	0.36	1.43

41.95	7.16	1.2	0.3	1.9	0.8	0.90	0.30	0.88
23.68	1.35	1.3	0.8	1.4	0.3	0.99	0.53	1.41
46.70	12.01	0.7	0.2	0.9	0.3	1.62	1.09	1.06
30.70	7.03	0.7	0.0	1.0	0.2	1.75	0.56	1.23
52.13	6.79	0.8	0.3	1.7	0.4	1.46	0.74	0.81
31.03	9.12	0.8	0.5	1.3	0.6	2.13	1.56	1.22
51.10	13.61	1.0	0.0	1.0	0.0	0.98	0.38	1.00
43.38	14.11	1.0	0.1	1.0	0.1	0.82	0.43	0.94
56.40	5.55	1.0	0.0	1.0	0.1	0.95	0.38	1.17
41.80	6.67	1.1	0.0	1.0	0.0	0.96	0.34	1.07
49.80	10.46	1.0	0.1	1.0	0.1	0.98	0.24	1.05
41.55	11.85	1.0	0.1	1.0	0.0	0.91	0.52	0.96
51.10	7.23	1.0	0.1	1.0	0.1	0.80	0.26	1.10
41.50	8.03	1.0	0.0	1.0	0.0	0.83	0.31	1.00
62.75	19.91	1.0	0.0	1.0	0.0	1.36	0.72	1.12
48.50	7.72	1.0	0.1	1.0	0.0	1.15	0.39	1.00
56.93	18.57	1.0	0.1	0.9	0.1	1.14	0.37	1.27
44.55	20.49	1.1	0.1	1.0	0.1	1.05	0.42	1.13
77.75	3.67	1.1	0.1	1.1	0.0	1.57	0.16	1.00
62.70	4.96	1.1	0.1	1.1	0.0	1.29	0.25	0.96
52.45	7.87	1.0	0.1	1.0	0.0	0.89	0.21	1.16
40.80	10.87	1.1	0.1	1.1	0.1	0.65	0.24	0.98
59.10	4.46	1.0	0.0	1.0	0.0	1.08	0.21	1.06
47.98	5.71	0.9	0.0	0.9	0.0	0.88	0.34	1.00
65.80	24.35	1.0	0.1	1.0	0.0	1.28	0.59	1.17
38.13	16.18	1.0	0.1	1.0	0.1	0.73	0.33	1.11
61.13	16.83	1.1	0.1	1.1	0.1	1.03	0.30	0.99
48.13	7.72	1.1	0.0	1.1	0.0	0.84	0.17	0.89
57.53	9.88	1.1	0.1	1.0	0.0	0.93	0.27	1.15
56.90	6.51	1.1	0.2	1.0	0.2	1.18	0.32	1.02
82.60	13.98	1.0	0.0	1.0	0.0	1.73	0.37	1.04
63.83	15.49	1.0	0.1	1.0	0.1	1.33	0.56	0.92
63.45	21.24	1.0	0.0	1.0	0.0	1.27	0.56	1.15
50.85	15.81	1.0	0.1	1.0	0.0	0.94	0.43	1.07
65.70	11.81	1.0	0.1	1.0	0.0	1.44	0.54	1.16
60.53	13.26	1.1	0.2	1.1	0.1	1.42	0.53	1.00
60.65	14.50	1.0	0.1	1.0	0.1	0.97	0.31	1.27
51.95	4.83	1.1	0.2	1.1	0.1	0.98	0.13	1.03
67.65	18.71	1.0	0.0	1.0	0.0	1.51	0.62	1.10
52.93	10.98	1.1	0.1	1.0	0.0	1.17	0.40	0.94
57.43	2.94	1.1	0.1	1.0	0.1	1.05	0.13	1.15
47.93	2.22	1.1	0.1	1.0	0.1	0.84	0.17	1.08
38.45	14.81	1.2	0.2	1.1	0.2	0.63	0.10	0.98
29.55	12.86	1.1	0.4	1.2	0.3	0.63	0.38	0.99
34.33	12.59	1.2	0.2	1.1	0.1	0.53	0.09	0.96
26.28	12.62	1.1	0.3	1.0	0.2	0.41	0.20	0.98
44.53	4.37	1.0	0.2	1.0	0.2	0.68	0.13	1.02

29.18	5.49	1.1	0.3	1.0	0.3	0.48	0.15	0.75
45.88	14.35	0.8	0.2	0.9	0.2	0.89	0.23	0.96
33.93	14.18	0.8	0.2	0.8	0.2	0.88	0.14	1.12
62.58	2.62	0.9	0.1	0.9	0.0	1.98	0.53	1.13
33.93	8.87	0.7	0.3	0.8	0.2	1.40	0.41	1.06
61.85	15.57	1.0	0.2	0.9	0.2	1.61	0.83	1.11
39.98	13.88	1.0	0.4	0.8	0.3	1.53	1.32	0.92
49.88	12.37	1.1	0.2	1.1	0.2	1.33	0.57	0.99
36.23	9.96	1.1	0.2	1.1	0.1	0.94	0.22	0.93
54.25	8.46	1.2	0.2	1.1	0.2	1.01	0.38	1.01
41.73	4.87	1.0	0.3	1.0	0.1	1.37	0.59	1.02
46.50	7.49	0.9	0.2	0.9	0.2	1.53	0.57	1.15
35.83	6.83	0.7	0.4	0.7	0.4	2.51	1.67	1.02
51.03	8.59	1.0	0.2	1.0	0.1	1.22	0.12	0.94
34.33	6.72	1.0	0.3	0.9	0.3	1.12	0.22	0.79
56.10	17.11	1.0	0.2	0.9	0.2	1.59	0.98	1.04
33.73	9.18	0.9	0.4	0.9	0.3	1.03	0.61	0.99
59.60	8.87	0.9	0.0	0.9	0.0	2.00	0.27	1.09
38.45	5.40	0.9	0.3	0.9	0.2	1.71	0.31	1.06
66.88	3.63	1.1	0.2	1.0	0.1	1.42	0.46	1.05
42.78	2.37	1.1	0.4	1.0	0.3	1.68	1.14	0.99
70.60	3.85	1.2	0.3	1.2	0.2	1.77	0.33	0.92
49.33	5.02	1.1	0.2	1.0	0.2	1.74	0.42	0.99
59.80	7.79	0.9	0.1	1.1	0.2	1.72	0.48	0.96
40.55	8.49	1.0	0.3	0.9	0.2	1.40	0.52	0.93
64.30	13.72	1.0	0.2	0.9	0.1	2.23	0.41	0.95
43.65	7.79	0.9	0.2	0.9	0.2	2.44	0.94	0.97
64.05	10.11	0.9	0.3	0.9	0.2	2.13	0.96	1.04
44.23	7.76	0.8	0.4	0.8	0.2	2.49	2.03	1.15
82.40	11.71	0.9	0.1	0.9	0.1	2.67	0.84	1.06
46.78	4.83	0.8	0.4	0.8	0.3	2.08	0.83	1.05
88.40	17.22	1.1	0.1	1.1	0.1	0.84	0.22	0.89
36.95	2.88	1.1	0.4	0.9	0.2	0.75	0.21	1.35
74.30	5.48	1.2	0.2	1.1	0.1	0.65	0.18	0.92
37.60	4.48	1.1	0.2	0.9	0.1	0.73	0.25	1.42
94.38	40.24	0.8	0.1	0.9	0.1	1.20	0.56	1.16
42.53	17.57	1.2	0.3	1.0	0.2	0.73	0.27	1.20
82.13	40.00	0.9	0.1	0.9	0.1	0.94	0.57	1.05
38.03	11.02	1.0	0.1	0.8	0.1	0.74	0.31	1.54
75.73	33.63	1.0	0.2	1.0	0.1	0.86	0.49	0.55
45.05	18.27	0.7	0.3	0.9	0.4	1.57	1.03	0.81
57.18	15.00	1.0	0.3	1.0	0.2	0.55	0.27	0.54
32.60	3.20	1.0	0.4	0.8	0.3	0.70	0.36	0.84
72.10	15.98	1.3	0.1	1.2	0.1	0.55	0.17	0.89
38.20	3.67	1.0	0.2	0.8	0.1	0.71	0.04	1.39
76.13	23.47	1.3	0.0	1.2	0.0	0.66	0.22	0.92
37.65	2.75	1.0	0.2	0.9	0.1	0.74	0.18	1.65

97.85	18.42	1.1	0.1	1.0	0.1	1.01	0.22	0.92
42.65	5.93	1.1	0.2	0.9	0.3	0.74	0.08	1.51
84.28	26.65	1.1	0.1	1.0	0.1	0.88	0.33	0.92
45.75	5.52	0.9	0.4	0.8	0.2	1.59	1.36	1.31
51.45	12.14	1.0	0.2	0.9	0.1	0.52	0.15	0.88
42.50	11.28	0.7	0.2	0.8	0.1	1.45	0.71	0.78
62.20	28.18	0.9	0.1	0.9	0.1	0.70	0.47	1.01
40.80	7.31	0.6	0.1	0.7	0.0	1.38	0.19	1.18
79.60	14.47	1.0	0.2	1.0	0.2	0.84	0.23	1.18
33.55	3.84	1.1	0.3	0.9	0.1	0.58	0.17	1.54
81.68	15.12	1.2	0.3	1.1	0.2	0.75	0.21	1.07
38.23	2.87	0.9	0.1	0.8	0.0	0.77	0.08	1.67
100.80	30.34	1.0	0.2	0.9	0.1	1.19	0.54	1.00
43.00	5.26	1.0	0.2	0.9	0.1	0.91	0.14	1.53
76.38	26.02	1.1	0.2	1.0	0.1	0.73	0.28	0.94
34.33	7.33	0.9	0.2	0.8	0.1	0.63	0.17	1.60
73.70	21.09	1.0	0.2	1.1	0.5	0.75	0.18	1.09
38.78	8.78	1.0	0.4	0.9	0.2	0.95	0.67	1.38
73.50	17.30	1.1	0.2	1.0	0.1	0.71	0.12	1.13
38.18	11.36	0.9	0.5	0.9	0.3	0.79	0.26	1.50

HWCCMI

1.36
0.95
1.29
1.15
1.20
0.89
1.31
0.64
1.26
0.91
1.71
1.53
1.43
1.17
1.29
1.47
1.56
1.14
1.88
1.33
1.85
1.07
0.68
0.58
1.07
0.94
1.05
0.97
1.20
0.87
1.05
0.88
1.11
1.22
1.31
1.22
1.27
1.14
1.00
0.67
1.57
1.33

1.06
0.92
1.45
1.18
1.38
1.31
1.11
1.05
0.93
0.79
1.26
1.35
0.98
0.88
0.89
0.93
1.08
1.10
1.28
1.15
1.41
1.40
1.27
1.14
1.57
1.45
1.51
1.19
1.02
1.19
0.89
0.77
1.08
0.97
0.78
0.62
1.04
0.98
0.71
0.60
0.95
0.69
1.09
0.85
0.66
0.58
1.12

0.97
0.87
0.81
0.77
0.81
1.02
1.07
1.01
0.94
1.00
1.20
1.11
1.27
1.28
1.07
1.07
1.21
0.99
1.11
1.09
1.05
1.32
1.01
0.53
0.59
0.54
0.49
0.54
0.48
0.51
0.42
0.94
1.00
1.43
1.12
1.40
1.23
0.62
0.65
1.29
1.17
1.32
1.19
1.44
1.35
0.89
0.88

1.50
1.49
1.35
1.34
0.77
0.72
1.46
1.27
0.96
0.92
1.08
1.78
1.09
0.83
0.82
1.09
0.71
0.57
1.16
0.87
0.96
1.20
1.06
1.08
1.20
1.54
0.73
1.26
0.77
1.00
1.16
0.93
1.05
1.15
1.03
1.01
1.27
1.65
1.15
1.23
0.83
1.06
0.67
1.16
0.71
1.07
1.30

1.45
1.12
0.92
0.64
0.84
0.75
0.86
1.00
0.94
1.08
1.60
0.75
0.87
0.64
1.01
0.92
0.94
0.72
0.86
0.66
0.83
0.97
0.86
0.63
0.93
0.94
0.86
0.90
1.01
1.08
0.91
0.81
1.21
0.75
0.50
0.98
0.88
0.89
0.88
0.87
0.89
0.92
0.89
0.60
0.91
0.42
0.34

1.35
0.36
0.92
0.48
0.83
0.75
0.72
0.51
0.99
0.94
1.36
1.46
0.87
0.86
1.05
1.36
0.60
1.80
0.55
0.42
0.94
1.08
1.04
1.21
0.58
1.19
0.99
1.03
0.87
0.98
0.56
0.68
0.90
1.44
0.66
0.87
1.02
0.88
0.99
1.01
0.62
0.19
1.06
1.12
0.84
0.41
1.01

0.87
1.29
1.44
1.02
1.38
1.11
0.29
0.68
1.05
1.24
1.09
0.74
0.86
0.48
0.76
0.59
0.91
0.74
0.87
0.70
0.65
0.69
0.70
0.33
0.70
0.41
0.81
0.40
0.86
0.64
0.69
1.15
1.51
0.89
1.03
0.66
1.30
0.99
1.11
0.89
1.03
1.08
1.11
0.92
1.17
0.73
0.71

0.36
0.69
0.61
0.92
0.71
0.63
0.50
0.69
0.55
0.51
0.37
0.79
0.77
0.70
0.82
0.78
0.87
1.09
0.81
0.98
1.29
0.92
1.39
1.01
1.26
0.98
0.38
0.48
0.55
1.09
0.82
1.06
1.36
0.80
1.22
1.14
1.22
0.92
1.10
0.59
0.89
0.90
1.23
0.88
0.47
0.51
0.63

0.99
0.80
0.90
0.73
0.81
0.78
1.44
1.07
0.95
0.45
0.75
1.10
0.94
0.72
0.90
0.86
0.82
0.71
0.81
0.80
0.83
0.83
0.77
0.75
0.74
0.94
0.79
0.80
0.95
0.64
0.88
0.79
0.78
0.87
0.75
0.52
0.77
0.51
0.79
0.75
0.75
0.92
0.78
0.69
0.62
0.69
0.90

0.70
0.86
1.04
0.86
1.03
0.81
1.12
0.81
0.73
0.72
1.07
0.87
0.86
0.95
1.37
0.88
0.97
0.83
0.86
0.80
0.89
0.83
1.19
1.01
1.07
0.99
0.96
1.16
1.12
0.90
1.39
1.03
1.74
1.17
1.50
1.34
1.81
1.16
1.66
1.44
1.40
1.30
0.83
0.85
1.20
1.17
1.12

0.97
1.61
1.19
1.00
1.01
1.00
0.93
1.02
0.98
1.07
0.92
1.20
0.97
0.97
0.42
1.19
1.07
1.11
1.02
0.95
0.58
0.71
0.70
1.06
0.79
1.12
0.91
0.92
0.64
1.61
1.08
1.54
1.46
1.46
1.45
1.11
0.81
1.29
0.99
1.22
1.18
1.21
1.21
0.96
0.67
1.16
1.01

1.15
1.06
1.16
1.02
1.19
0.94
1.04
1.06
0.88
0.80
1.01
0.97
0.99
0.99
2.03
0.43
0.93
0.76
1.28
0.90
0.67
0.58
1.18
0.91
1.13
0.73
1.06
0.66
0.92
0.71
1.19
1.14
1.88
1.67
1.28
1.27
1.59
1.35
1.85
1.27
1.79
1.35
1.45
1.25
0.82
0.69
0.91

0.81
1.52
1.20
1.61
1.39
1.45
1.18
1.61
1.32
1.11
0.80
1.27
0.91
1.03
0.75
1.05
1.00

HWCCLI		MBCCMI		ACCMI		CWCCMI		HWCCMI
Mean	Std Dev	Mean	Std Dev	Mean	Std Dev	Mean	Std Dev	Mean
0.97	0.451299	0.942601	0.276287	0.968315	0.366705	0.921881	0.344785	0.908331
1.28	0.932983	1.063474	0.437675	1.095946	0.393851	1.089918	0.415094	0.982407
1.19	0.283116	1.197126	0.463077	1.142714	0.113715	1.380446	0.336826	1.216038
0.95	0.253979	1.047885	0.400175	1.054936	0.125771	0.933894	0.308058	0.995426
1.18	0.381196	1.437253	0.650713	1.006793	0.154099	1.002333	0.316945	1.138932
1.21	0.533326	1.195332	0.60847	0.897833	0.240234	0.934513	0.300478	1.015161
0.78	0.286865	0.817371	0.324767	0.97541	0.200557	0.710676	0.133218	0.797869
1.02	0.499198	0.76966	0.242159	1.282917	0.476284	1.103089	0.231382	0.862515

HWCCLI		MBCCMI		ACCMI		CWCCMI		HWCCMI
Mean	Std Dev	Mean	Std Dev	Mean	Std Dev	Mean	Std Dev	Mean
0.75	0.25878	0.739448	0.27894	1.090513	0.31149	0.961114	0.405133	0.815925
0.91	0.32665	0.814116	0.325286	1.060292	0.261624	0.954513	0.331367	0.857081
1.23	0.605579	1.094502	0.508823	0.851956	0.269198	0.941555	0.282516	0.990791
0.95	0.441703	1.048659	0.405506	1.183009	0.265947	1.071044	0.383764	1.011809
1.06	0.465737	1.014728	0.353672	1.031138	0.296011	1.026044	0.372946	0.974386
1.28	0.717681	1.064756	0.480235	0.880305	0.253613	1.036611	0.326051	0.986305
1.06	0.360946	1.290683	0.490931	1.283898	0.354863	1.048114	0.364754	1.134823
1.07	0.373674	1.222805	0.547163	1.107877	0.260552	0.968261	0.356657	1.03862
1.33	0.661958	1.239842	0.633978	0.988984	0.249003	1.079089	0.356252	1.096523

HWCCLI		MBCCMI		ACCMI		CWCCMI		HWCCMI
Mean	Std Dev	Mean	Std Dev	Mean	Std Dev	Mean	Std Dev	Mean
0.99	0.480243	0.916727	0.426848	0.978823	0.316561	0.937246	0.329825	0.913691
0.93	0.456633	0.84865	0.395138	1.023018	0.281649	0.967542	0.35423	0.862174
1.06	0.43153	1.072901	0.42734	1.013035	0.27979	1.040108	0.378703	1.005756
1.12	0.681011	1.012528	0.401691	1.049932	0.315532	1.049024	0.343066	0.97591
1.18	0.477279	1.254635	0.508982	1.102807	0.309614	1.016514	0.339416	1.105184
1.12	0.520873	1.247584	0.606222	1.151032	0.319803	1.047129	0.38142	1.074794

HWCCLI		MBCCMI		ACCMI		CWCCMI		HWCCMI
Mean	Std Dev	Mean	Std Dev	Mean	Std Dev	Mean	Std Dev	Mean
0.94	0.349385	0.910074	0.420262	0.961633	0.229231	0.960039	0.345564	0.911109
0.99	0.56363	0.855303	0.403162	1.040207	0.353352	0.944749	0.339403	0.864755
0.99	0.333964	1.073381	0.446356	1.024702	0.205456	1.018845	0.390098	1.001496
1.20	0.719517	1.012048	0.380371	1.038266	0.369087	1.070288	0.328081	0.980171
1.15	0.457273	1.312308	0.571574	1.083589	0.26597	1.032619	0.40633	1.133272
1.15	0.540154	1.189911	0.540672	1.170251	0.353235	1.031024	0.309923	1.046706

HWCCLI		MBCCMI		ACCMI		CWCCMI		HWCCMI
Mean	Std Dev	Mean	Std Dev	Mean	Std Dev	Mean	Std Dev	Mean

0.96	0.380834	1.064008	0.451201	1.147092	0.328103	1.041043	0.417392	1.019592
0.89	0.383592	0.988519	0.465695	1.224522	0.310936	1.012472	0.353053	0.955447
1.03	0.386619	1.03433	0.424639	1.062564	0.278183	0.981529	0.336996	0.976138
0.99	0.410072	1.000102	0.476465	1.070307	0.270416	0.984349	0.371191	0.937254
1.26	0.56433	1.145925	0.539473	0.88501	0.247874	0.971296	0.287441	1.028902
1.30	0.747701	1.120142	0.558243	0.929153	0.2765	1.066874	0.356425	1.020177

HWCCLI		MBCCMI		ACCMI		CWCCMI		HWCCMI
Mean	Std Dev	Mean	Std Dev	Mean	Std Dev	Mean	Std Dev	Mean
0.91	0.342936	1.069058	0.448034	1.151892	0.244271	1.014582	0.409	1.011895
0.93	0.420507	0.983469	0.467866	1.219722	0.381273	1.038932	0.362897	0.963144
1.03	0.335036	1.064228	0.445596	0.990357	0.158204	0.979492	0.374655	0.996605
0.99	0.45298	0.970204	0.452666	1.142514	0.337365	0.986386	0.33311	0.916786
1.14	0.459391	1.162477	0.616257	0.927675	0.247535	1.017428	0.362145	1.037377
1.42	0.793636	1.10359	0.470613	0.886489	0.277033	1.020742	0.28831	1.011702

HWCCLI		MBCCMI		ACCMI		CWCCMI		HWCCMI
Mean	Std Dev	Mean	Std Dev	Mean	Std Dev	Mean	Std Dev	Mean
1.06	0.406401	1.131998	0.485084	1.004565	0.222844	0.972508	0.344926	1.034869
1.10	0.524266	1.030844	0.460878	1.058545	0.369651	1.023403	0.35735	0.981552
0.99	0.379058	1.065178	0.533032	1.042051	0.254115	1.03516	0.413711	0.995716
1.13	0.700055	1.007331	0.472489	1.107271	0.355226	1.007303	0.299254	0.946203

	HWCCLI		MBCCMI		ACCMI		CWCCMI	
Std Dev	Mean	Std Dev	Mean	Std Dev	Mean	Std Dev	Mean	Std Dev
0.40	0.774688	0.271638	0.744175	0.274919	1.050191	0.372147	0.990425	0.396937
0.41	0.721875	0.246713	0.734722	0.287225	1.130834	0.235228	0.931803	0.417406
0.34	0.854063	0.279064	0.829422	0.375158	1.069931	0.263616	0.888363	0.3035
0.43	0.958125	0.365208	0.798809	0.271675	1.050653	0.263471	1.020663	0.34929
0.58	1.351563	0.594372	1.176584	0.483449	0.816347	0.240905	0.93295	0.279812
0.47	1.11625	0.602847	1.012419	0.527751	0.887566	0.294312	0.950159	0.289409
0.50	0.98375	0.369208	1.172469	0.427305	1.153263	0.208663	1.031156	0.433833
0.41	0.9175	0.507854	0.92485	0.346321	1.212756	0.31364	1.110931	0.328337
0.43	1.095313	0.427196	1.047294	0.339299	1.031022	0.303479	1.067878	0.402383
0.51	1.018125	0.505221	0.982163	0.369987	1.031253	0.293211	0.984209	0.342255
0.58	1.114063	0.49154	0.998941	0.495348	0.854822	0.240389	1.021291	0.296872
0.85	1.437188	0.866745	1.130572	0.463009	0.905788	0.267558	1.051931	0.356974
0.41	1.112188	0.417672	1.275381	0.449284	1.237822	0.360051	1.101547	0.426137
0.37	1.016875	0.292624	1.305984	0.536153	1.329975	0.349138	0.994681	0.287917
0.42	1.129375	0.388827	1.226275	0.463869	1.086738	0.271792	0.988347	0.278023
0.56	1.005313	0.353042	1.219334	0.627083	1.129016	0.251338	0.948175	0.424714
0.52	1.30875	0.589897	1.26225	0.612811	0.983863	0.237849	0.959647	0.287283

0.81	1.350938	0.735945	1.217434	0.663513	0.994106	0.263398	1.198531	0.382022

	HWCCLI		MBCCMI		ACCMI		CWCCMI	
Std Dev	Mean	Std Dev	Mean	Std Dev	Mean	Std Dev	Mean	Std Dev
0.44	0.755	0.267979	0.7763	0.251999	1.03915	0.232685	0.953122	0.416883
0.38	0.741563	0.253353	0.702597	0.303015	1.141875	0.370936	0.969106	0.399546
0.35	0.946563	0.288384	0.842425	0.316106	0.977847	0.148359	0.957497	0.331108
0.46	0.865625	0.360966	0.785806	0.336834	1.142738	0.321028	0.951528	0.336897
0.33	1.110625	0.39205	1.111497	0.559243	0.867903	0.263317	0.969497	0.287012
0.66	1.357188	0.748466	1.077506	0.461308	0.836009	0.27823	0.913613	0.279673
0.38	0.88875	0.301745	1.077047	0.383465	1.155069	0.168403	1.037419	0.399842
0.50	1.0125	0.545474	1.020272	0.430656	1.21095	0.337292	1.104669	0.370262
0.46	1.04125	0.336056	1.076966	0.352987	0.981106	0.150007	1.004606	0.400592
0.49	1.072188	0.572182	0.952491	0.348721	1.081169	0.387817	1.047481	0.348196
0.37	1.035938	0.349773	1.066131	0.58209	0.937931	0.22632	1.014509	0.381332
0.91	1.515313	0.897664	1.063381	0.360357	0.822678	0.269513	1.058713	0.263866
0.46	1.081563	0.376693	1.353828	0.482033	1.261456	0.27355	1.053206	0.415923
0.31	1.0475	0.349673	1.227538	0.499212	1.306341	0.42432	1.043022	0.31199
0.43	1.099063	0.368312	1.273294	0.536549	1.012119	0.177338	0.976372	0.398607
0.55	1.035625	0.38215	1.172316	0.561502	1.203634	0.295948	0.96015	0.315417
0.60	1.28375	0.580799	1.309803	0.691064	0.977191	0.24676	1.068278	0.41118
0.77	1.375938	0.74081	1.169881	0.573714	1.000778	0.254612	1.0899	0.297684

	HWCCLI		MBCCMI		ACCMI		CWCCMI	
Std Dev	Mean	Std Dev	Mean	Std Dev	Mean	Std Dev	Mean	Std Dev
0.32	0.977083	0.3459	0.973613	0.431713	0.954092	0.226475	0.91419	0.2716
0.58	1.009792	0.588201	0.859842	0.418676	1.003554	0.387316	0.960302	0.380829
0.43	0.897708	0.351956	0.846535	0.402959	0.969175	0.234104	1.005888	0.404116
0.48	0.966458	0.543302	0.850765	0.391414	1.07686	0.315616	0.929196	0.29549
0.40	1.002292	0.364699	1.11729	0.469585	1.006475	0.179744	0.983579	0.371183
0.57	1.126458	0.485305	1.028513	0.380249	1.019596	0.354731	1.096638	0.381549
0.41	0.975	0.303399	1.029473	0.422208	1.042929	0.228771	1.05411	0.40899
0.80	1.273542	0.894347	0.995583	0.383796	1.056935	0.38575	1.043938	0.265643
0.47	1.209167	0.465553	1.305092	0.502601	1.053129	0.249957	1.019756	0.380214
0.43	1.157708	0.492286	1.204179	0.515587	1.152485	0.355306	1.013271	0.297108
0.55	1.100417	0.447066	1.319525	0.63846	1.114048	0.280378	1.045481	0.434535
0.72	1.148333	0.589364	1.175644	0.569761	1.188017	0.354003	1.048777	0.324393

	HWCCLI		MBCCMI		ACCMI		CWCCMI	
Std Dev	Mean	Std Dev	Mean	Std Dev	Mean	Std Dev	Mean	Std Dev
0.43	0.964792	0.372861	1.128571	0.429471	1.12525	0.239119	1.00285	0.410278

0.44	0.948958	0.39243	0.999446	0.46748	1.168933	0.399301	1.079235	0.425242
0.43	0.852083	0.303644	1.009546	0.462665	1.178533	0.248954	1.026315	0.411717
0.38	0.91875	0.450497	0.967492	0.472648	1.27051	0.359335	0.998629	0.28643
0.35	1.070208	0.34438	1.092746	0.415193	0.97909	0.140883	0.946081	0.30116
0.46	0.982292	0.423774	0.975915	0.430254	1.146038	0.349769	1.016977	0.369168
0.47	0.987708	0.32375	1.03571	0.476749	1.001625	0.174591	1.012902	0.43674
0.54	1	0.484772	0.964494	0.478527	1.13899	0.328158	0.955796	0.29341
0.43	1.153542	0.476041	1.174677	0.59637	0.909356	0.22197	0.968594	0.318261
0.64	1.362708	0.628296	1.117173	0.480529	0.860665	0.271493	0.973998	0.256316
0.47	1.133333	0.44693	1.150277	0.641612	0.945994	0.271836	1.066263	0.398657
0.91	1.469583	0.934092	1.090006	0.465166	0.912313	0.282943	1.067485	0.312815

	CWCCLI		HWCCLI		MBCCMI		ACCMI	
Std Dev	Mean	Std Dev	Mean	Std Dev	Mean	Std Dev	Mean	Std Dev
0.66	0.8975	0.512014	0.695	0.275741	0.677225	0.131746	0.94505	0.554366
0.47	1.1675	0.548657	0.53	0.261916	0.47055	0.259507	0.91385	0.589883
0.18	0.705	0.269629	0.485	0.031091	0.56165	0.169897	0.88855	0.28898
0.13	0.7775	0.251578	0.6425	0.221867	0.799875	0.311815	1.170625	0.2917
0.16	0.7375	0.1466	0.7375	0.201722	0.821825	0.224454	0.95175	0.078313
0.16	0.6675	0.455732	0.6575	0.249449	0.764875	0.330332	1.3551	0.292709
0.24	0.79	0.155991	0.7325	0.109962	0.820075	0.123433	0.9495	0.057389
0.43	1.2675	0.390843	0.94	0.574398	0.960425	0.347269	1.21005	0.081023
0.11	1.04	0.219697	1.1875	0.186078	0.981225	0.26714	0.774625	0.176513
0.19	1.65	0.529969	2.18	0.719305	1.431175	0.449795	0.75525	0.153987
0.18	0.9775	0.083417	1.0025	0.229692	0.924325	0.225779	0.750225	0.248269
1.04	1.2875	0.611249	1.7675	1.02802	1.1951	0.364455	0.71515	0.284003
0.26	0.8325	0.363444	0.89	0.256255	1.068725	0.283108	1.1912	0.20596
0.61	1.38	0.891478	1.195	0.665006	1.276475	0.574965	1.139725	0.042526
0.36	1.025	0.120416	0.715	0.085829	0.8551	0.060089	1.229875	0.415995
0.38	1.27	0.68678	1.19	1.351049	1.020475	0.765783	1.274175	0.500026
0.42	1.06	0.400083	1.05	0.666783	0.9068	0.289852	0.84385	0.141455
0.48	1.2825	0.448655	0.9275	0.609556	1.08	0.434594	1.169475	0.16123
0.13	1.0075	0.725414	0.8375	0.161529	0.855175	0.234382	0.93705	0.180026
0.24	1.025	0.411542	0.785	0.170783	0.7445	0.160464	1.172125	0.092206
0.24	1.255	0.535319	1.165	0.53157	0.948125	0.248223	0.7727	0.167935
0.49	1.86	0.983904	1.56	0.746905	1.055425	0.17037	0.72685	0.203174
0.35	1.1825	0.227797	1.2125	0.135739	0.931825	0.28351	0.728375	0.374487
1.11	3.095	1.199708	3.1675	1.396815	1.234325	0.553938	0.727	0.262804
0.44	0.7075	0.351034	0.8775	0.269861	1.25035	0.193856	1.567	0.327348
0.40	0.98	0.029439	1.1325	0.388791	1.41385	0.351528	1.3277	0.584449
0.16	0.55	0.151877	0.8125	0.158824	1.32155	0.148722	1.76885	0.038591
0.26	0.69	0.355622	0.9075	0.17595	1.4281	0.268773	1.761575	0.515635
0.13	0.785	0.212525	1.0525	0.49257	1.055275	0.489214	0.8606	0.048475
0.44	1.24	0.490374	0.8675	0.435995	0.85545	0.340633	1.329325	0.145379

0.31	0.6675	0.504802	0.8175	0.33935	0.990025	0.204514	0.953	0.034582
0.87	0.9375	1.00427	0.94	0.578504	0.97475	0.2687	1.311375	0.096652
0.38	1.5675	0.826131	1.59	0.966264	0.982975	0.286359	0.719225	0.285543
0.36	1.54	0.224054	1.675	0.345688	1.205475	0.415493	0.845725	0.274184
0.29	1.7375	0.90441	1.5375	0.678301	1.014575	0.255486	0.59825	0.123477
0.49	2.1725	1.412619	1.9875	1.521652	1.2317	0.412807	0.82195	0.146922
0.12	1.2275	0.055	1.035	0.25619	0.965975	0.387778	0.97385	0.13044
0.13	0.9925	0.108128	0.9025	0.262345	0.805375	0.445944	0.9071	0.153078
0.06	1.5925	0.505528	1.1375	0.112064	0.935275	0.368897	1.154175	0.047557
0.06	0.5975	0.249583	0.7925	0.121209	1.022	0.349485	1.1441	0.046604
0.05	1.01	0.215252	0.955	0.181934	1.01955	0.304553	1.083875	0.048509
0.07	0.8125	0.391014	0.83	0.192007	0.92205	0.552797	0.963825	0.081791
0.08	1.475	0.298496	0.9975	0.148183	0.811875	0.239613	1.13465	0.170117
0.07	0.865	0.18303	0.8175	0.151959	0.855475	0.329953	1.0208	0.089671
0.02	1.1975	0.219754	1.26	0.386092	1.3494	0.725409	1.106175	0.039299
0.09	0.785	0.302269	0.965	0.204206	1.169275	0.363958	1.02685	0.063819
0.13	1.3625	0.235425	1.175	0.296929	1.09865	0.439385	1.205775	0.049479
0.06	0.685	0.210159	0.8475	0.343645	1.11525	0.502532	1.178925	0.048079
0.06	1.1825	0.478983	1.3875	0.122848	1.756375	0.193544	1.117075	0.020762
0.03	0.9025	0.480026	1.1225	0.049917	1.45705	0.261754	1.083275	0.024851
0.06	1.415	0.329697	1.035	0.108474	0.896925	0.23802	1.1609	0.08733
0.02	1.0475	0.436377	0.76	0.286938	0.704675	0.194178	1.090475	0.134755
0.08	1.4375	0.327656	1.165	0.10083	1.09365	0.211553	1.069575	0.106969
0.09	1.0925	0.191898	1.035	0.121518	0.8276	0.319734	0.93895	0.069699
0.07	1.44	0.241523	1.29	0.444597	1.304125	0.640614	1.18025	0.10713
0.05	0.855	0.151548	0.7875	0.327656	0.7269	0.347487	1.08875	0.052441
0.12	1.25	0.194079	1.0675	0.184639	1.1809	0.483504	1.109875	0.174796
0.11	0.97	0.165126	0.855	0.155885	0.9596	0.180731	1.017225	0.141114
0.03	1.535	0.258779	1.0875	0.209185	0.974275	0.264311	1.211225	0.080771
0.06	1.0975	0.329077	1.13	0.315383	1.2026	0.151911	1.079425	0.228302
0.06	1.62	0.577466	1.6325	0.316478	1.743275	0.341102	1.0521	0.038559
0.07	1.115	0.35819	1.255	0.299944	1.368625	0.562315	0.955375	0.130283
0.07	1.4325	0.201556	1.2825	0.387072	1.2534	0.603557	1.1283	0.06294
0.08	1.0075	0.275363	1.0375	0.303795	0.9244	0.42683	1.049675	0.062101
0.10	1.18	0.363868	1.2625	0.294095	1.4948	0.526013	1.21545	0.163592
0.03	0.77	0.283666	1.15	0.379561	1.5251	0.427673	1.106225	0.146877
0.10	1.62	0.324654	1.1575	0.217313	1.0243	0.411628	1.30945	0.085223
0.11	0.83	0.386523	0.945	0.154596	1.104625	0.185559	1.1591	0.12691
0.08	1.1475	0.292731	1.3375	0.430687	1.5193	0.574069	1.11955	0.053544
0.17	0.6375	0.29239	0.9925	0.248512	1.26515	0.416854	1.011675	0.134953
0.09	1.2075	0.346542	1.0675	0.132759	1.126225	0.158927	1.2366	0.072527
0.06	1.0775	0.306309	0.9	0.080416	0.906175	0.199483	1.1671	0.056434
0.17	0.6525	0.276451	0.65	0.182757	0.733175	0.095688	1.134575	0.053776
0.21	0.8	0.331461	0.7475	0.298371	0.60675	0.139559	1.056425	0.20785
0.10	0.61	0.255865	0.585	0.202073	0.633725	0.142171	1.1247	0.064246
0.17	0.8075	0.35706	0.68	0.303754	0.420925	0.222273	1.020375	0.166382
0.05	1.1275	0.382742	0.985	0.293201	0.638325	0.128086	0.969625	0.18566

0.11	0.9125	0.529111	0.77	0.357305	0.53205	0.204332	0.8599	0.320387
0.13	1.2375	0.443274	1.1225	0.292276	0.7173	0.137045	0.8102	0.24868
0.48	1.46	0.73598	1.2725	0.546283	0.6501	0.160635	0.783825	0.162448
0.05	1.2725	0.25224	1.4925	0.235425	1.677025	0.373746	0.9752	0.119419
0.14	1.23	0.37372	1.285	0.277188	1.02975	0.371326	0.790975	0.287849
0.17	1.2425	0.460534	1.3575	0.420585	1.515625	0.825592	1.04025	0.098171
0.28	1.2425	0.710041	1.33	0.864214	1.139	0.728786	0.874875	0.32771
0.16	0.7575	0.31213	0.9375	0.338268	1.3911	0.23003	1.085225	0.142497
0.18	0.91	0.481318	0.9175	0.391354	1.011175	0.072203	1.0421	0.291105
0.08	0.935	0.099499	0.9575	0.151959	1.11785	0.225234	1.15515	0.163909
0.15	1.16	0.422295	1.2225	0.442973	1.25405	0.261368	0.9818	0.14902
0.10	0.9375	0.488493	1.1275	0.286167	1.25655	0.339733	0.983075	0.17151
0.18	1.295	0.801852	1.665	0.580144	1.32675	0.49153	0.6549	0.31865
0.03	1.0525	0.419712	1.1025	0.280639	1.183	0.309625	0.906775	0.158616
0.10	0.94	0.409471	0.995	0.301828	1.0865	0.325077	0.76825	0.246604
0.15	1.0025	0.283828	1.185	0.266396	1.5208	0.939711	0.9737	0.103156
0.11	1.035	0.327058	1.0325	0.346157	0.92755	0.598933	0.899625	0.266114
0.11	0.9575	0.155644	1.2875	0.181361	1.8723	0.276935	1.01675	0.09594
0.23	1.205	0.784453	1.3625	0.624627	1.398225	0.230853	0.89995	0.347428
0.17	1.235	0.077244	1.2925	0.159661	1.45005	0.274904	1.086525	0.017982
0.22	1.0175	0.43714	1.22	0.628278	1.494125	0.363824	1.011425	0.213299
0.11	0.94	0.210871	1.205	0.23445	2.1065	0.20561	1.10705	0.141176
0.10	1.0275	0.177271	1.255	0.24173	1.8274	0.306347	1.058	0.162853
0.05	1.155	0.42501	1.33	0.304521	1.590525	0.440408	0.893375	0.135333
0.14	1.2125	0.77976	1.2725	0.580251	1.31175	0.473902	0.8506	0.140516
0.08	0.9275	0.393309	1.3375	0.23852	2.1342	0.080235	0.919975	0.126296
0.04	0.755	0.222486	1.2825	0.315529	2.168525	0.534571	0.904325	0.173071
0.15	1.14	0.526751	1.4475	0.379067	1.87015	0.657279	0.9628	0.218058
0.20	1.3075	0.6651	1.665	0.831404	1.729175	0.924961	0.90695	0.255509
0.21	1.445	0.608413	1.82	0.466405	2.46235	0.709843	0.977325	0.092535
0.27	1.565	0.78356	1.7275	0.557158	1.602175	0.688626	0.7967	0.180835
0.13	0.64	0.113431	0.7975	0.172506	0.952825	0.233715	1.017075	0.143851
0.27	1.065	0.659166	0.84	0.320416	0.741525	0.06337	1.4536	0.604971
0.18	0.6225	0.089954	0.655	0.110905	0.75055	0.106433	1.075225	0.178859
0.41	0.945	0.225462	0.7975	0.212191	0.753775	0.179557	1.468925	0.284149
0.18	0.8775	0.25773	1.1175	0.379243	1.05345	0.584059	0.9661	0.024096
0.22	0.8875	0.247437	0.78	0.241385	0.88325	0.441117	1.409275	0.311578
0.17	0.86	0.276526	0.925	0.480174	0.857	0.509959	0.957075	0.041869
0.19	1.155	0.342394	0.8575	0.311809	0.718225	0.253898	1.539125	0.143306
0.06	0.665	0.195021	0.8125	0.413632	0.81335	0.425844	0.5512	0.044767
0.25	1.875	1.117154	1.63	0.959062	0.961475	0.496362	0.5505	0.198073
0.10	0.6975	0.144309	0.5975	0.237118	0.532375	0.180594	0.539775	0.059673
0.34	1.26	0.631981	0.8525	0.431074	0.579025	0.088123	0.79555	0.33253
0.08	0.585	0.10504	0.565	0.148885	0.71165	0.200848	1.157875	0.05678
0.30	1.225	0.386997	0.855	0.120416	0.7072	0.100188	1.409625	0.419071
0.04	0.49	0.137356	0.6225	0.192592	0.81865	0.269246	1.14325	0.035359
0.09	1.075	0.294109	0.8375	0.161323	0.731075	0.140753	1.666425	0.36325

0.11	0.7325	0.092511	0.945	0.146629	1.0723	0.270396	0.9659	0.051333
0.36	1.1125	0.109049	0.8475	0.083815	0.8147	0.095717	1.62245	0.080744
0.09	0.5725	0.087321	0.8125	0.225592	0.944125	0.392585	0.962375	0.028394
0.58	1.465	0.897422	1.535	1.161163	1.012975	0.231639	1.23445	0.707037
0.35	0.585	0.106301	0.5425	0.120104	0.5005	0.157194	0.80765	0.146561
0.30	1.68	0.640052	1.505	0.613759	0.898625	0.378716	0.53095	0.2391
0.20	0.83	0.129872	0.74	0.378153	0.600325	0.376368	0.883175	0.108906
0.10	1.87	0.407676	1.51	0.208487	0.8307	0.181879	0.7004	0.086278
0.27	0.725	0.236995	0.8175	0.218689	0.842125	0.191142	1.17545	0.028545
0.17	0.89	0.298105	0.67	0.151658	0.64065	0.160762	1.727	0.308274
0.30	0.64	0.246035	0.7325	0.210456	0.863375	0.193437	1.206375	0.041946
0.20	1.2425	0.340135	0.9025	0.120381	0.72315	0.083435	1.559975	0.12744
0.21	0.885	0.313847	1.115	0.465081	1.1099	0.399688	0.9459	0.021782
0.17	1.1875	0.205163	0.985	0.143875	0.8674	0.127512	1.492425	0.337114
0.20	0.68	0.126754	0.72	0.238188	0.787325	0.309932	0.9992	0.090547
0.35	1.4075	0.583288	0.8425	0.268126	0.570925	0.111535	1.4757	0.355234
0.36	0.735	0.11619	0.755	0.122338	0.752375	0.284916	1.039575	0.126876
0.39	1.1575	0.330795	1.0075	0.549507	0.7734	0.329792	1.2654	0.1231
0.24	0.6725	0.124197	0.715	0.110905	0.750475	0.180015	1.1642	0.055234
0.56	1.8	0.807259	1.0525	0.389219	0.6458	0.228903	1.190725	0.343662

Std Dev
0.214586
0.333867
0.298586
0.252955
0.304939
0.266053
0.253939
0.188709

Std Dev
0.241863
0.253729
0.327016
0.306124
0.24372
0.273263
0.301895
0.270574
0.306209

Std Dev
0.291377
0.278252
0.281588
0.267969
0.298119
0.291531

Std Dev
0.291329
0.278761
0.295422
0.253072
0.328617
0.250113

Std Dev

0.332829
0.288752
0.261193
0.270307
0.301669
0.311156

Std Dev
0.323401
0.300736
0.280072
0.245748
0.348533
0.257006

Std Dev
0.318698
0.278275
0.317053
0.262762

HWCCMI

Mean	Std Dev
0.825391	0.252681
0.806459	0.2342
0.867788	0.257627
0.846375	0.253427
1.047894	0.318033
0.933688	0.330835
1.072466	0.327085
0.951153	0.275439
0.986775	0.215574
0.961997	0.271894
0.958028	0.286735
1.014581	0.26055
1.160919	0.325892
1.108728	0.278586
1.07385	0.272272
1.003391	0.268492
1.080784	0.295273

1.112263	0.32071

HWCCMI

Mean	Std Dev
0.831828	0.240273
0.800022	0.246229
0.891653	0.257674
0.822509	0.248938
1.009847	0.344997
0.971734	0.31234
1.020784	0.293397
1.002834	0.322799
1.012916	0.259115
0.935856	0.224744
0.970788	0.335766
1.001822	0.19626
1.183072	0.336008
1.086575	0.259835
1.085247	0.295111
0.991994	0.239173
1.131497	0.354962
1.06155	0.249057

HWCCMI

Mean	Std Dev
0.943556	0.288714
0.883825	0.29399
0.878663	0.293333
0.845685	0.264378
1.012685	0.310524
0.998827	0.252483
0.990306	0.282356
0.961515	0.254941
1.148365	0.32722
1.062004	0.262219
1.118179	0.332771
1.031408	0.23918

HWCCMI

Mean	Std Dev
1.055794	0.348294

0.98339	0.316103
0.967996	0.293525
0.942898	0.286445
1.010331	0.254905
0.941944	0.265576
0.982879	0.305268
0.891629	0.224156
1.038481	0.348807
1.019323	0.249181
1.036273	0.351947
1.004081	0.26702

CWCCMI		HWCCMI	
Mean	Std Dev	Mean	Std Dev
0.965025	0.452326	0.76335	0.21324
1.42705	0.637259	0.651675	0.317091
0.897825	0.380761	0.614675	0.063895
1.050875	0.410809	0.881125	0.335341
0.780125	0.236401	0.78075	0.276281
0.804575	0.600636	0.751825	0.275722
0.773675	0.20484	0.705875	0.052233
1.02735	0.064213	0.699875	0.228774
0.80165	0.168285	0.92725	0.223107
0.952975	0.198977	1.2697	0.353298
0.896275	0.215268	0.892175	0.188565
0.717175	0.142336	0.912675	0.263152
0.955425	0.356145	1.0324	0.229752
1.219375	0.750193	1.1734	0.507398
1.319825	0.299401	0.9282	0.227577
1.198775	0.232169	0.998275	0.621119
0.959575	0.290405	0.853275	0.203884
1.50465	0.485262	1.05	0.474166
1.0397	0.539293	0.917925	0.065608
1.11835	0.345778	0.860275	0.077585
1.0516	0.208047	0.978225	0.225113
1.1358	0.287376	0.9828	0.117678
0.899675	0.272584	0.920675	0.241758
1.18715	0.502033	1.117725	0.298566
0.836525	0.297141	1.067675	0.170852
1.194975	0.575519	1.2248	0.208141
0.70585	0.131293	1.050325	0.129423
0.798325	0.274513	1.097625	0.084485
0.728775	0.180013	0.95335	0.295616
1.145625	0.177633	0.819	0.310409

0.666025	0.273482	0.8783	0.14983
0.738475	0.343196	0.87715	0.048635
1.0124	0.377488	0.986575	0.258901
1.0511	0.131241	1.1496	0.263379
1.3039	0.690499	1.09895	0.141745
1.345925	0.225489	1.1658	0.354579
1.2002	0.071269	1.0154	0.271188
0.9616	0.145742	0.8805	0.290316
1.574225	0.529077	1.12045	0.1116
0.642575	0.279613	0.848025	0.13243
1.036575	0.169042	0.992175	0.211242
0.81015	0.388595	0.839725	0.238714
1.5134	0.348194	1.0178	0.139747
0.88335	0.168913	0.838275	0.159669
1.1801	0.199531	1.2475	0.39584
0.8083	0.321136	0.9853	0.156319
1.3039	0.293192	1.1321	0.364645
0.72915	0.273375	0.9057	0.418518
1.31255	0.514069	1.548875	0.075464
1.0377	0.586088	1.272225	0.102388
1.4253	0.369252	1.04445	0.159323
1.130625	0.340791	0.8238	0.220621
1.44645	0.302839	1.1751	0.086062
1.02925	0.196899	0.9728	0.114351
1.459925	0.275835	1.313275	0.487268
0.83755	0.12904	0.77265	0.327674
1.39975	0.21918	1.216425	0.334465
1.10765	0.166829	0.9765	0.158944
1.619425	0.280748	1.143625	0.197303
1.114775	0.158804	1.152	0.132608
1.6369	0.553557	1.649775	0.278244
1.1676	0.451965	1.2968	0.316418
1.4076	0.237677	1.26675	0.4275
0.99295	0.263656	1.028275	0.318537
1.2287	0.372382	1.30615	0.234301
0.826425	0.256732	1.22835	0.267502
1.663175	0.220285	1.206375	0.287335
0.8956	0.293729	1.05475	0.098181
1.158575	0.246403	1.348575	0.376774
0.6855	0.308433	1.073175	0.224171
1.281275	0.279254	1.143875	0.060812
1.14935	0.267671	0.968825	0.047195
0.79315	0.421909	0.779425	0.303124
0.8778	0.471184	0.793025	0.344129
0.72025	0.317848	0.692275	0.256746
0.842425	0.406951	0.7081	0.342289
1.0222	0.124041	0.900975	0.091797

0.89975	0.307976	0.780125	0.146535
1.0266	0.433561	0.92525	0.290954
1.031175	0.523802	0.908175	0.380571
1.08185	0.151065	1.2681	0.052499
0.857625	0.245767	0.912725	0.239704
1.133575	0.229506	1.253	0.315669
1.05145	0.297001	1.076025	0.377638
0.825225	0.334116	1.0053	0.247301
0.951775	0.368946	0.969875	0.266445
1.0853	0.250998	1.0937	0.168144
1.0641	0.185471	1.1205	0.135738
0.79335	0.373394	0.94195	0.152036
0.8045	0.469246	0.964075	0.187742
0.96685	0.269385	1.034575	0.17302
0.84455	0.191574	0.92165	0.178353
0.962875	0.333514	1.1385	0.348676
0.898125	0.210157	0.907775	0.247077
0.89805	0.151343	1.2079	0.180772
0.8676	0.324654	1.036475	0.146439
1.3073	0.212275	1.351125	0.074376
0.991975	0.243623	1.14555	0.06608
1.10995	0.085436	1.42935	0.077884
1.0855	0.085431	1.32295	0.131072
1.03855	0.254596	1.211075	0.15771
0.98865	0.373374	1.0906	0.236069
0.92545	0.442758	1.305	0.278002
0.7175	0.268094	1.171275	0.206988
1.034325	0.432225	1.29525	0.20946
0.94435	0.212397	1.183625	0.208831
1.31715	0.43761	1.668025	0.23998
1.102375	0.269236	1.260375	0.124438
0.729625	0.129007	0.90565	0.176756
0.96895	0.315417	0.8141	0.061914
0.744675	0.186025	0.7634	0.060777
0.981575	0.157137	0.823625	0.100477
0.7257	0.131454	0.96745	0.413955
1.027825	0.283526	0.929275	0.388476
0.7817	0.179987	0.84295	0.410958
1.12805	0.219428	0.8328	0.239459
0.654075	0.141719	0.77465	0.349438
1.127025	0.422097	0.997925	0.414585
0.70455	0.152578	0.584	0.155909
1.0652	0.095945	0.713825	0.068063
0.763	0.093382	0.7353	0.165356
1.1842	0.195987	0.84235	0.076294
0.612725	0.175375	0.77805	0.24135
1.0508	0.222437	0.82225	0.055132

0.771725	0.080787	1.0016	0.186958
1.233525	0.216955	0.9354	0.127248
0.599275	0.053866	0.865625	0.277967
1.007475	0.109145	1.01	0.128071
0.561425	0.094931	0.525275	0.123768
1.0531	0.129762	0.938725	0.249295
0.723275	0.037193	0.635675	0.291433
1.1055	0.205031	0.902575	0.162434
0.709275	0.09221	0.81525	0.147741
0.967825	0.19652	0.736375	0.08485
0.71225	0.099877	0.834325	0.158176
1.145025	0.241316	0.840225	0.064711
0.82125	0.135793	1.040125	0.320262
1.1288	0.076371	0.94215	0.113158
0.73905	0.168466	0.7816	0.268025
1.240125	0.382872	0.752675	0.164591
0.7245	0.120046	0.75545	0.215711
1.066425	0.162499	0.854025	0.198358
0.7141	0.194486	0.755275	0.178637
1.374175	0.296193	0.836975	0.247458
