# Supplementary material for: Gypsum, crop rotation, and cover crop impacts on soil organic carbon and biological dynamics in rainfed transitional no-till corn-soybean systems
Source: PLoS One. 2022 Sep 27;17(9):e0275198. doi: 10.1371/journal.pone.0275198 (PMC9514652; doi:10.1371/journal.pone.0275198)
Supplement: S1 Table — (DOCX) [file pone.0275198.s002.docx]

**S1 Table.** Interactive effects of gypsum, crop rotation, and cover crop on total soil organic carbon (SOC), total nitrogen (TN), microbial biomass (SBM), metabolic quotient (qR), active carbon (AC), cold (CWC) and hot (HWC) salt water extractable carbon, carbon pool index (CPI), nitrogen pool index (NPI), carbon lability index (CLI) and carbon management index (CMI) at different soil depths under a rainfed transitioning no-till soybean-corn rotation at Alabama site (2012 to 2016).

| Gypsum | Crop | Cover | Depth | SOC | TN | SMBC | SMBC: | AC | CWC | HWC | CPI | NPI | CLI | | | | CMI | | | |
| --- | --- | --- | --- | --- | --- | --- | --- | --- | --- | --- | --- | --- | --- | --- | --- | --- | --- | --- | --- | --- |
| (Mg/ha) | rotation | crop | (cm) | (g/kg) | | (mg/kg) | SOC(%) | (mg/kg) | | |  |  | SMBC | AC | CWC | HWC | SMBC | AC | CWC | HWC |
| 0 | CS | No | 0 | 9.2 | 0.63 | 107 | 1.2 | 237 | 13.9 | 36.4 | 1.15 | 1.04 | 0.61 | 0.91 | 0.9 | 0.7 | 0.68 | 0.95 | 0.97 | 0.76 |
|  |  |  | 15 | 5.5 | 0.37 | 42 | 0.75 | 130 | 11.5 | 17.6 | 1.23 | 0.93 | 0.38 | 0.74 | 1.17 | 0.53 | 0.47 | 0.91 | 1.43 | 0.65 |
|  |  | Rye | 0 | 10.2 | 0.66 | 89 | 0.9 | 224 | 12.9 | 29.5 | 1.27 | 1.1 | 0.45 | 0.69 | 0.71 | 0.49 | 0.56 | 0.89 | 0.9 | 0.61 |
|  |  |  | 15 | 6.2 | 0.38 | 72 | 1.2 | 166 | 8.5 | 23.8 | 1.36 | 0.96 | 0.6 | 0.86 | 0.78 | 0.64 | 0.8 | 1.17 | 1.05 | 0.88 |
|  | SC | No | 0 | 8.4 | 0.67 | 129 | 1.55 | 239 | 11.2 | 37.3 | 1.05 | 1.12 | 0.78 | 0.93 | 0.74 | 0.74 | 0.82 | 0.95 | 0.78 | 0.78 |
|  |  |  | 15 | 5.2 | 0.42 | 68 | 1.35 | 190 | 6.5 | 20.2 | 1.15 | 1.06 | 0.69 | 1.17 | 0.67 | 0.66 | 0.76 | 1.36 | 0.8 | 0.75 |
|  |  | Rye | 0 | 7.9 | 0.66 | 128 | 1.7 | 238 | 11.1 | 33.7 | 0.99 | 1.1 | 0.86 | 1 | 0.79 | 0.73 | 0.82 | 0.95 | 0.77 | 0.71 |
|  |  |  | 15 | 4 | 0.37 | 84 | 2.5 | 168 | 8.3 | 18.7 | 0.88 | 0.93 | 1.3 | 1.48 | 1.27 | 0.94 | 0.96 | 1.21 | 1.03 | 0.7 |
|  | SS | No | 0 | 6.3 | 0.64 | 153 | 2.43 | 195 | 11.6 | 44.2 | 0.79 | 1.07 | 1.25 | 0.99 | 1.04 | 1.19 | 0.98 | 0.77 | 0.8 | 0.93 |
|  |  |  | 15 | 2.8 | 0.43 | 123 | 4.65 | 106 | 7.7 | 33.9 | 0.62 | 1.07 | 2.46 | 1.25 | 1.65 | 2.18 | 1.43 | 0.76 | 0.95 | 1.27 |
|  |  | Rye | 0 | 7.3 | 0.79 | 144 | 2.08 | 189 | 12.9 | 42.5 | 0.91 | 1.31 | 1.07 | 0.82 | 0.98 | 1 | 0.92 | 0.75 | 0.9 | 0.89 |
|  |  |  | 15 | 3 | 0.38 | 103 | 4.3 | 99 | 5.8 | 24.3 | 0.66 | 0.96 | 2.27 | 1.34 | 1.29 | 1.77 | 1.2 | 0.72 | 0.72 | 0.91 |
| 1.1 | CS | No | 0 | 9.4 | 0.61 | 168 | 1.8 | 298 | 13.7 | 49.4 | 1.18 | 1.02 | 0.92 | 1.03 | 0.83 | 0.89 | 1.07 | 1.19 | 0.96 | 1.03 |
|  |  |  | 15 | 5.1 | 0.39 | 112 | 2.45 | 160 | 9.9 | 31.5 | 1.13 | 0.98 | 1.25 | 1.28 | 1.38 | 1.2 | 1.28 | 1.14 | 1.22 | 1.17 |
|  |  | Rye | 0 | 10.3 | 0.63 | 135 | 1.33 | 309 | 19 | 44.5 | 1.28 | 1.05 | 0.68 | 0.98 | 1.03 | 0.72 | 0.86 | 1.23 | 1.32 | 0.93 |
|  |  |  | 15 | 5.2 | 0.38 | 88 | 2.48 | 179 | 9.7 | 26.6 | 1.14 | 0.96 | 1.31 | 1.21 | 1.27 | 1.19 | 1.02 | 1.27 | 1.2 | 1 |
|  | SC | No | 0 | 8 | 0.5 | 141 | 2.2 | 212 | 13.8 | 40.7 | 1 | 0.83 | 1.13 | 0.96 | 1.06 | 1.05 | 0.91 | 0.84 | 0.96 | 0.85 |
|  |  |  | 15 | 5.6 | 0.39 | 95 | 1.93 | 165 | 12.2 | 28.3 | 1.25 | 0.99 | 0.98 | 1.07 | 1.28 | 0.93 | 1.08 | 1.17 | 1.5 | 1.05 |
|  |  | Rye | 0 | 8.9 | 0.66 | 135 | 1.48 | 236 | 15 | 43.7 | 1.12 | 1.09 | 0.76 | 0.84 | 1.01 | 0.84 | 0.86 | 0.94 | 1.04 | 0.92 |
|  |  |  | 15 | 5.2 | 0.38 | 66 | 1.33 | 165 | 9 | 23.1 | 1.15 | 0.96 | 0.68 | 1.07 | 1.03 | 0.79 | 0.74 | 1.17 | 1.12 | 0.86 |
|  | SS | No | 0 | 7.4 | 0.68 | 148 | 2.2 | 195 | 15.1 | 46.7 | 0.93 | 1.13 | 1.13 | 0.89 | 1.26 | 1.17 | 0.95 | 0.77 | 1.05 | 0.98 |
|  |  |  | 15 | 3.4 | 0.43 | 92 | 3.15 | 103 | 9.2 | 26.3 | 0.75 | 1.08 | 1.64 | 1.12 | 1.86 | 1.56 | 1.06 | 0.73 | 1.14 | 0.98 |
|  |  | Rye | 0 | 6 | 0.6 | 146 | 2.38 | 183 | 12.9 | 43.9 | 0.76 | 0.99 | 1.23 | 0.94 | 1.18 | 1.21 | 0.93 | 0.73 | 0.9 | 0.92 |
|  |  |  | 15 | 2 | 0.34 | 103 | 6.8 | 100 | 9.6 | 29.8 | 0.44 | 0.85 | 3.71 | 2 | 3.1 | 3.17 | 1.23 | 0.73 | 1.19 | 1.12 |
| 2.2 | CS | No | 0 | 10.2 | 0.63 | 196 | 1.98 | 389 | 12 | 51.1 | 1.28 | 1.05 | 1.01 | 1.3 | 0.71 | 0.88 | 1.25 | 1.57 | 0.84 | 1.07 |
|  |  |  | 15 | 5.5 | 0.41 | 124 | 2.7 | 186 | 9.6 | 32.9 | 1.23 | 1.03 | 1.4 | 1.11 | 0.98 | 1.13 | 1.41 | 1.33 | 1.19 | 1.22 |
|  |  | Rye | 0 | 10.5 | 0.82 | 207 | 2 | 439 | 10.2 | 50.1 | 1.31 | 1.37 | 1.03 | 1.36 | 0.55 | 0.81 | 1.32 | 1.77 | 0.71 | 1.05 |
|  |  |  | 15 | 5.6 | 0.46 | 125 | 2.25 | 246 | 6.5 | 29.5 | 1.24 | 1.14 | 1.16 | 1.41 | 0.69 | 0.91 | 1.43 | 1.76 | 0.8 | 1.1 |
|  | SC | No | 0 | 7.6 | 0.49 | 164 | 2.28 | 217 | 10.5 | 45.4 | 0.95 | 0.83 | 1.18 | 0.93 | 0.79 | 1.05 | 1.06 | 0.86 | 0.73 | 0.95 |
|  |  |  | 15 | 4.4 | 0.37 | 75 | 1.73 | 185 | 9.2 | 21.9 | 0.99 | 0.92 | 0.89 | 1.43 | 1.24 | 0.87 | 0.86 | 1.33 | 1.15 | 0.82 |
|  |  | Rye | 0 | 9.4 | 1.14 | 155 | 1.78 | 241 | 9.6 | 42 | 1.17 | 1.9 | 0.9 | 0.88 | 0.67 | 0.82 | 0.99 | 0.95 | 0.67 | 0.88 |
|  |  |  | 15 | 5.7 | 0.57 | 86 | 1.93 | 183 | 6 | 23.7 | 1.27 | 1.42 | 0.99 | 1.41 | 0.94 | 0.94 | 0.97 | 1.31 | 0.74 | 0.88 |
|  | SS | No | 0 | 5.6 | 0.56 | 151 | 3.1 | 180 | 14.5 | 46.7 | 0.7 | 0.93 | 1.62 | 1.06 | 1.57 | 1.59 | 0.98 | 0.72 | 1.01 | 0.99 |
|  |  |  | 15 | 3.1 | 0.38 | 104 | 3.4 | 118 | 8.5 | 30.7 | 0.69 | 0.96 | 1.75 | 1.23 | 1.54 | 1.68 | 1.21 | 0.85 | 1.05 | 1.15 |
|  |  | Rye | 0 | 6.4 | 1.01 | 157 | 2.83 | 150 | 18.7 | 52.1 | 0.8 | 1.68 | 1.46 | 0.81 | 1.74 | 1.54 | 1.01 | 0.6 | 1.3 | 1.1 |
|  |  |  | 15 | 3.6 | 0.53 | 105 | 4 | 115 | 10.8 | 31 | 0.8 | 1.31 | 2.13 | 1.22 | 2.17 | 1.99 | 1.23 | 0.82 | 1.35 | 1.17 |
| **Probability > F** | | |  |  |  |  |  |  |  |  |  |  |  |  |  |  |  |  |  |  |
| Gypsum | | |  | 0.93 | 0.001 | 0.001 | 0.2 | 0.003 | 0.02 | 0.001 | 0.89 | 0.002 | 0.2 | 0.2 | 0.01 | 0.08 | 0.001 | 0.001 | 0.02 | 0.001 |
| Crop rotation (CR) | | |  | 0.001 | 0.65 | 0.06 | 0.001 | 0.001 | 0.23 | 0.001 | 0.001 | 0.66 | 0.001 | 0.77 | 0.1 | 0.12 | 0.02 | 0.001 | 0.33 | 0.001 |
| Cover crop (CC) | | |  | 0.42 | 0.001 | 0.55 | 0.55 | 0.43 | 0.73 | 0.29 | 0.61 | 0.001 | 0.48 | 0.49 | 0.77 | 0.78 | 0.7 | 0.36 | 0.45 | 0.32 |
| Soil depth | | |  | 0.001 | 0.001 | 0.001 | 0.001 | 0.001 | 0.001 | 0.001 | 0.52 | 0.03 | 0.001 | 0.001 | 0.001 | 0.001 | 0.03 | 0.01 | 0.01 | 0.1 |
| Gypsum x CR | | |  | 0.001 | 0.3 | 0.86 | 0.88 | 0.001 | 0.03 | 0.09 | 0.85 | 0.88 | 0.32 | 0.05 | 0.22 | 0.91 | 0.001 | 0.001 | 0.03 | 0.11 |
| Gypsum x CC | | |  | 0.46 | 0.75 | 0.41 | 0.001 | 0.69 | 0.9 | 0.74 | 0.4 | 0.001 | 0.7 | 0.82 | 0.64 | 0.54 | 0.5 | 0.68 | 0.92 | 0.81 |
| Gypsum x depth | | |  | 0.23 | 0.35 | 0.92 | 0.35 | 0.62 | 0.85 | 0.33 | 0.89 | 0.65 | 0.34 | 0.8 | 0.3 | 0.36 | 0.88 | 0.82 | 0.95 | 0.92 |
| CR x CC | | |  | 0.98 | 0.37 | 0.79 | 0.28 | 0.23 | 0.64 | 0.9 | 0.81 | 0.32 | 0.34 | 0.91 | 0.22 | 0.4 | 0.99 | 0.16 | 0.5 | 0.97 |
| CR x depth | | |  | 0.46 | 0.001 | 0.3 | 0.89 | 0.001 | 0.38 | 0.93 | 0.26 | 0.93 | 0.001 | 0.09 | 0.16 | 0.01 | 0.14 | 0.01 | 0.54 | 0.43 |
| CC x depth | | |  | 0.59 | 0.09 | 0.36 | 0.01 | 0.84 | 0.2 | 0.91 | 0.49 | 0.03 | 0.07 | 0.09 | 0.57 | 0.14 | 0.64 | 0.62 | 0.14 | 0.88 |
| Gypsum x CR x CC | | |  | 0.61 | 0.1 | 0.3 | 0.17 | 0.83 | 0.15 | 0.75 | 0.39 | 0.15 | 0.1 | 0.27 | 0.22 | 0.18 | 0.45 | 0.83 | 0.12 | 0.54 |
| Gypsum x CR x depth | | |  | 0.95 | 0.41 | 0.74 | 0.98 | 0.04 | 0.67 | 0.98 | 0.75 | 0.99 | 0.39 | 0.52 | 0.27 | 0.47 | 0.86 | 0.58 | 0.78 | 0.96 |
| Gypsum x CC x depth | | |  | 0.8 | 0.63 | 0.97 | 0.16 | 0.97 | 0.93 | 0.89 | 0.83 | 0.56 | 0.59 | 0.92 | 0.85 | 0.58 | 0.76 | 0.96 | 0.96 | 0.95 |
| CR x CC x depth | | |  | 0.84 | 0.53 | 0.94 | 0.52 | 0.54 | 0.82 | 0.73 | 0.95 | 0.63 | 0.48 | 0.66 | 0.34 | 0.63 | 0.91 | 0.36 | 0.65 | 0.81 |
| Gypsum x CR x CC x depth | | | | 0.73 | 0.49 | 1 | 0.83 | 0.98 | 0.62 | 0.4 | 1 | 0.95 | 0.46 | 0.84 | 0.34 | 0.44 | 0.56 | 0.99 | 0.56 | 0.29 |
