# Supplementary material for: Gypsum, crop rotation, and cover crop impacts on soil organic carbon and biological dynamics in rainfed transitional no-till corn-soybean systems
Source: PLoS One. 2022 Sep 27;17(9):e0275198. doi: 10.1371/journal.pone.0275198 (PMC9514652; doi:10.1371/journal.pone.0275198)
Supplement: S2 Table — (DOCX) [file pone.0275198.s003.docx]

**S2 Table.** Interactive effects of gypsum, crop rotation, and cover crop on total soil organic C (SOC), total nitrogen (TN), microbial biomass (SBM), metabolic quotient (qR), active carbon (AC), cold (CWC) and hot (HWC) salt water extractable carbon, carbon pool index (CPI), nitrogen pool index (NPI), carbon lability index (CLI) and carbon management index (CMI) under a rainfed transitioning no-till soybean-corn rotation at Hoytville site (2012 to 2016).

| Gypsum | Crop | Cover | Depth | SOC | TN | SMBC | SMBC: | AC | CWC | HWC | CPI | NPI | CLI | | | | CMI | | | |
| --- | --- | --- | --- | --- | --- | --- | --- | --- | --- | --- | --- | --- | --- | --- | --- | --- | --- | --- | --- | --- |
| (Mg/ha) | rotation | crop | (cm) | (g/kg) | | (mg/kg) | SOC(%) | (mg/kg) | | |  |  | SMBC | AC | CWC | HWC | SMBC | AC | CWC | HWC |
| 0 | CS | No | 0 | 16.5 | 2.01 | 145 | 0.88 | 492 | 20.2 | 51.1 | 0.98 | 1 | 0.98 | 1 | 1.23 | 1.04 | 0.97 | 0.97 | 1.2 | 1.02 |
|  |  |  | 15 | 16 | 1.91 | 119 | 0.73 | 449 | 15.9 | 43.4 | 0.97 | 0.96 | 0.82 | 0.94 | 0.99 | 0.9 | 0.81 | 0.91 | 0.96 | 0.88 |
|  |  | Rye | 0 | 16.6 | 2.1 | 140 | 0.85 | 581 | 26.5 | 56.4 | 0.99 | 1.05 | 0.95 | 1.17 | 1.59 | 1.14 | 0.94 | 1.15 | 1.57 | 1.12 |
|  |  |  | 15 | 17.7 | 2.04 | 151 | 0.88 | 567 | 10.6 | 41.8 | 1.07 | 1.02 | 0.96 | 1.07 | 0.6 | 0.79 | 1.02 | 1.14 | 0.64 | 0.85 |
|  | SC | No | 0 | 17.4 | 2.03 | 152 | 0.88 | 545 | 17.4 | 49.8 | 1.04 | 1.02 | 0.98 | 1.05 | 1.01 | 0.96 | 1.02 | 1.08 | 1.04 | 0.99 |
|  |  |  | 15 | 16.5 | 1.98 | 136 | 0.8 | 478 | 13.4 | 41.6 | 1 | 0.99 | 0.91 | 0.96 | 0.81 | 0.83 | 0.92 | 0.96 | 0.81 | 0.84 |
|  |  | Rye | 0 | 17.2 | 1.99 | 121 | 0.7 | 570 | 25.3 | 51.1 | 1.03 | 1 | 0.8 | 1.1 | 1.48 | 1 | 0.81 | 1.13 | 1.51 | 1.02 |
|  |  |  | 15 | 17 | 2.02 | 126 | 0.75 | 508 | 14.6 | 41.5 | 1.03 | 1.01 | 0.83 | 1 | 0.87 | 0.82 | 0.86 | 1.02 | 0.88 | 0.84 |
|  | SS | No | 0 | 16.6 | 1.95 | 202 | 1.2 | 558 | 19.8 | 62.8 | 0.99 | 0.98 | 1.36 | 1.12 | 1.2 | 1.26 | 1.35 | 1.11 | 1.18 | 1.25 |
|  |  |  | 15 | 17 | 2.02 | 172 | 1.03 | 511 | 13.3 | 48.5 | 1.03 | 1.01 | 1.15 | 1 | 0.79 | 0.97 | 1.17 | 1.03 | 0.81 | 0.99 |
|  |  | Rye | 0 | 16.1 | 1.87 | 164 | 1 | 605 | 21.9 | 56.9 | 0.95 | 0.94 | 1.14 | 1.27 | 1.36 | 1.18 | 1.1 | 1.21 | 1.3 | 1.13 |
|  |  |  | 15 | 17.3 | 2 | 163 | 0.95 | 581 | 12 | 44.6 | 1.05 | 1 | 1.05 | 1.13 | 0.69 | 0.85 | 1.12 | 1.18 | 0.73 | 0.91 |
| 1.1 | CS | No | 0 | 18.8 | 2.18 | 262 | 1.4 | 564 | 22 | 77.8 | 1.12 | 1.09 | 1.57 | 1 | 1.18 | 1.39 | 1.76 | 1.12 | 1.31 | 1.55 |
|  |  |  | 15 | 18.7 | 2.16 | 213 | 1.15 | 538 | 17.1 | 62.7 | 1.13 | 1.08 | 1.29 | 0.96 | 0.9 | 1.12 | 1.46 | 1.08 | 1.04 | 1.27 |
|  |  | Rye | 0 | 16.9 | 2.08 | 134 | 0.8 | 583 | 23.9 | 52.5 | 1.01 | 1.04 | 0.89 | 1.16 | 1.42 | 1.04 | 0.9 | 1.16 | 1.43 | 1.04 |
|  |  |  | 15 | 18.4 | 2.17 | 104 | 0.58 | 542 | 18.6 | 40.8 | 1.11 | 1.08 | 0.65 | 0.98 | 1.05 | 0.76 | 0.7 | 1.09 | 1.13 | 0.82 |
|  | SC | No | 0 | 17 | 1.99 | 164 | 0.98 | 539 | 24.3 | 59.1 | 1.01 | 1 | 1.08 | 1.06 | 1.44 | 1.17 | 1.09 | 1.07 | 1.45 | 1.18 |
|  |  |  | 15 | 15.6 | 1.87 | 122 | 0.8 | 468 | 17 | 48 | 0.94 | 0.93 | 0.88 | 1 | 1.09 | 1.04 | 0.83 | 0.94 | 1.03 | 0.97 |
|  |  | Rye | 0 | 17 | 1.96 | 194 | 1.15 | 592 | 24.5 | 65.8 | 1.01 | 0.98 | 1.28 | 1.17 | 1.44 | 1.29 | 1.3 | 1.18 | 1.46 | 1.31 |
|  |  |  | 15 | 16.2 | 1.93 | 107 | 0.65 | 538 | 13.8 | 38.1 | 0.99 | 0.97 | 0.73 | 1.11 | 0.86 | 0.79 | 0.73 | 1.09 | 0.84 | 0.77 |
|  | SS | No | 0 | 18.9 | 2.16 | 177 | 0.95 | 560 | 23.5 | 61.1 | 1.13 | 1.08 | 1.03 | 0.99 | 1.25 | 1.07 | 1.18 | 1.11 | 1.4 | 1.22 |
|  |  |  | 15 | 18.9 | 2.17 | 141 | 0.78 | 506 | 18.2 | 48.1 | 1.15 | 1.08 | 0.84 | 0.89 | 0.97 | 0.86 | 0.96 | 1.02 | 1.11 | 0.98 |
|  |  | Rye | 0 | 17.8 | 2.02 | 146 | 0.85 | 611 | 27.2 | 57.5 | 1.06 | 1.01 | 0.93 | 1.15 | 1.54 | 1.09 | 0.97 | 1.21 | 1.62 | 1.14 |
|  |  |  | 15 | 17.5 | 2.02 | 177 | 1.03 | 535 | 18.4 | 56.9 | 1.06 | 1.01 | 1.18 | 1.02 | 1.1 | 1.13 | 1.2 | 1.08 | 1.11 | 1.15 |
| 2.2 | CS | No | 0 | 17 | 2.02 | 259 | 1.55 | 529 | 27.4 | 82.6 | 1.02 | 1.01 | 1.73 | 1.04 | 1.62 | 1.63 | 1.74 | 1.05 | 1.64 | 1.65 |
|  |  |  | 15 | 17 | 2.02 | 200 | 1.18 | 474 | 19.2 | 63.8 | 1.03 | 1.01 | 1.33 | 0.92 | 1.12 | 1.26 | 1.37 | 0.96 | 1.17 | 1.3 |
|  |  | Rye | 0 | 16.5 | 1.98 | 187 | 1.13 | 567 | 23.6 | 63.5 | 0.98 | 0.99 | 1.27 | 1.15 | 1.43 | 1.28 | 1.25 | 1.13 | 1.41 | 1.27 |
|  |  |  | 15 | 16.3 | 1.94 | 136 | 0.83 | 520 | 16.4 | 50.9 | 0.99 | 0.97 | 0.94 | 1.07 | 1.01 | 1.04 | 0.92 | 1.05 | 0.99 | 1.03 |
|  | SC | No | 0 | 17.6 | 2.04 | 223 | 1.25 | 612 | 20.7 | 65.7 | 1.05 | 1.02 | 1.44 | 1.16 | 1.18 | 1.26 | 1.49 | 1.22 | 1.23 | 1.31 |
|  |  |  | 15 | 18.2 | 2.12 | 224 | 1.28 | 549 | 13.6 | 60.5 | 1.11 | 1.06 | 1.42 | 1 | 0.77 | 1.15 | 1.53 | 1.11 | 0.83 | 1.23 |
|  |  | Rye | 0 | 17.6 | 2.02 | 154 | 0.88 | 657 | 28 | 60.7 | 1.04 | 1.01 | 0.97 | 1.27 | 1.62 | 1.16 | 1.02 | 1.31 | 1.66 | 1.21 |
|  |  |  | 15 | 18.7 | 2.16 | 163 | 0.88 | 575 | 14.8 | 52 | 1.14 | 1.08 | 0.98 | 1.03 | 0.83 | 0.95 | 1.1 | 1.16 | 0.9 | 1.05 |
|  | SS | No | 0 | 17.1 | 1.98 | 226 | 1.33 | 563 | 19.5 | 67.7 | 1.02 | 0.99 | 1.51 | 1.1 | 1.15 | 1.34 | 1.52 | 1.12 | 1.16 | 1.35 |
|  |  |  | 15 | 18 | 2.09 | 186 | 1.03 | 503 | 11.3 | 52.9 | 1.09 | 1.05 | 1.17 | 0.94 | 0.64 | 0.99 | 1.27 | 1.01 | 0.69 | 1.07 |
|  |  | Rye | 0 | 18.1 | 2.09 | 169 | 0.95 | 622 | 21.5 | 57.4 | 1.08 | 1.05 | 1.05 | 1.15 | 1.21 | 1.07 | 1.13 | 1.24 | 1.28 | 1.14 |
|  |  |  | 15 | 17.8 | 2.08 | 134 | 0.75 | 577 | 19 | 47.9 | 1.08 | 1.04 | 0.84 | 1.08 | 1.08 | 0.9 | 0.91 | 1.17 | 1.15 | 0.97 |
| **Probability > F** | | |  |  |  |  |  |  |  |  |  |  |  |  |  |  |  |  |  |  |
| Gypsum | | |  | 0.1 | 0.04 | 0.01 | 0.01 | 0.06 | 0.01 | 0.001 | 0.1 | 0.03 | 0.01 | 0.08 | 0.11 | 0.14 | 0.01 | 0.06 | 0.01 | 0.004 |
| Crop rotation (CR) | | |  | 0.25 | 0.28 | 0.4 | 0.46 | 0.03 | 0.4 | 0.24 | 0.25 | 0.29 | 0.04 | 0.06 | 0.3 | 0.27 | 0.014 | 0.04 | 0.4 | 0.23 |
| Cover crop (CC) | | |  | 0.6 | 0.55 | 0.001 | 0.003 | 0.001 | 0.09 | 0.1 | 0.59 | 0.55 | 0.12 | 0.001 | 0.06 | 0.01 | 0.001 | 0.001 | 0.09 | 0.002 |
| Soil depth | | |  | 0.59 | 0.59 | 0.01 | 0.01 | 0.001 | 0.001 | 0.001 | 0.06 | 0.6 | 0.01 | 0.001 | 0.001 | 0.001 | 0.03 | 0.001 | 0.001 | 0.001 |
| Gypsum x CR | | |  | 0.21 | 0.2 | 0.003 | 0.002 | 0.002 | 0.36 | 0.28 | 0.003 | 0.001 | 0.19 | 0 | 0.25 | 0.1 | 0.21 | 0.002 | 0.37 | 0.28 |
| Gypsum x CC | | |  | 0.09 | 0.09 | 0.22 | 0.35 | 0.48 | 0.81 | 0.18 | 0.23 | 0.35 | 0.08 | 0.87 | 0.97 | 0.23 | 0.09 | 0.49 | 0.82 | 0.18 |
| Gypsum x depth | | |  | 0.54 | 0.57 | 0.61 | 0.54 | 0.74 | 0.78 | 0.92 | 0.6 | 0.49 | 0.66 | 0.39 | 0.56 | 0.98 | 0.55 | 0.75 | 0.76 | 0.92 |
| CR x CC | | |  | 0.3 | 0.42 | 0.58 | 0.52 | 0.79 | 0.32 | 0.1 | 0.55 | 0.55 | 0.42 | 0.2 | 0.45 | 0.21 | 0.31 | 0.77 | 0.32 | 0.1 |
| CR x depth | | |  | 0.79 | 0.73 | 0.54 | 0.41 | 0.39 | 0.66 | 0.88 | 0.5 | 0.46 | 0.76 | 0.73 | 0.8 | 0.8 | 0.8 | 0.4 | 0.66 | 0.88 |
| CC x depth | | |  | 0.4 | 0.54 | 0.3 | 0.49 | 0.78 | 0.08 | 0.98 | 0.31 | 0.48 | 0.52 | 0.4 | 0.05 | 0.78 | 0.42 | 0.78 | 0.08 | 1 |
| Gypsum x CR x CC | | |  | 0.04 | 0.09 | 0.35 | 0.18 | 0.17 | 0.17 | 0.14 | 0.3 | 0.17 | 0.08 | 0.56 | 0.14 | 0.22 | 0.04 | 0.17 | 0.17 | 0.14 |
| Gypsum x CR x depth | | |  | 0.41 | 0.43 | 0.32 | 0.26 | 0.97 | 0.64 | 0.58 | 0.26 | 0.28 | 0.43 | 0.36 | 0.64 | 0.58 | 0.41 | 0.97 | 0.63 | 0.58 |
| Gypsum x CC x depth | | |  | 0.9 | 0.9 | 0.52 | 0.61 | 0.82 | 0.22 | 0.9 | 0.51 | 0.6 | 0.96 | 0.56 | 0.14 | 0.71 | 0.89 | 0.82 | 0.22 | 0.9 |
| CR x CC x depth | | |  | 0.72 | 0.63 | 0.57 | 0.65 | 0.99 | 0.5 | 0.42 | 0.59 | 0.62 | 0.64 | 0.63 | 0.41 | 0.33 | 0.71 | 0.99 | 0.49 | 0.42 |
| Gypsum x CR x CC x depth | | | | 0.88 | 0.89 | 0.94 | 0.96 | 0.95 | 0.62 | 0.81 | 0.94 | 0.96 | 0.88 | 0.59 | 0.62 | 0.77 | 0.88 | 0.95 | 0.62 | 0.81 |
