# Supplementary material for: Gypsum, crop rotation, and cover crop impacts on soil organic carbon and biological dynamics in rainfed transitional no-till corn-soybean systems
Source: PLoS One. 2022 Sep 27;17(9):e0275198. doi: 10.1371/journal.pone.0275198 (PMC9514652; doi:10.1371/journal.pone.0275198)
Supplement: S3 Table — (DOCX) [file pone.0275198.s004.docx]

**S3 Table.** Interactive effects of gypsum, crop rotation, and cover crop on total soil organic C (SOC), total nitrogen (TN), microbial biomass (SBM), metabolic quotient (qR), active carbon (AC), cold (CWC) and hot (HWC) salt water extractable carbon, carbon pool index (CPI), nitrogen pool index (NPI), carbon lability index (CLI) and carbon management index (CMI) under a rainfed transitioning no-till soybean-corn rotation at Indiana site (2012 to 2016).

| Gypsum | Crop | Cover | Depth | SOC | TN | SMBC | SMBC: | AC | CWC | HWC | CPI | NPI | CLI | | | | CMI | | | |
| --- | --- | --- | --- | --- | --- | --- | --- | --- | --- | --- | --- | --- | --- | --- | --- | --- | --- | --- | --- | --- |
| (Mg/ha) | rotation | crop | (cm) | (g/kg) | | (mg/kg) | SOC(%) | (mg/kg) | | |  |  | SMBC | AC | CWC | HWC | SMBC | AC | CWC | HWC |
| 0 | CS | No | 0 | 17.3 | 1.81 | 139 | 0.83 | 543 | 26.7 | 38.5 | 1.19 | 1.14 | 0.63 | 0.98 | 0.65 | 0.65 | 0.73 | 1.13 | 0.79 | 0.78 |
|  |  |  | 15 | 12.4 | 1.5 | 87 | 0.83 | 383 | 22.2 | 29.6 | 1.12 | 1.15 | 0.63 | 0.99 | 0.8 | 0.75 | 0.61 | 1.06 | 0.88 | 0.79 |
|  |  | Rye | 0 | 17.4 | 1.82 | 119 | 0.65 | 543 | 24.1 | 34.3 | 1.19 | 1.14 | 0.53 | 0.96 | 0.61 | 0.59 | 0.63 | 1.12 | 0.72 | 0.69 |
|  |  |  | 15 | 12 | 1.34 | 59 | 0.5 | 371 | 21.3 | 26.3 | 1.09 | 1.03 | 0.41 | 0.98 | 0.81 | 0.68 | 0.42 | 1.02 | 0.84 | 0.71 |
|  | SC | No | 0 | 14.1 | 1.52 | 120 | 0.88 | 464 | 34.3 | 44.5 | 0.96 | 0.95 | 0.68 | 1.02 | 1.13 | 0.99 | 0.64 | 0.97 | 1.02 | 0.9 |
|  |  |  | 15 | 12.5 | 1.28 | 76 | 0.63 | 313 | 22.7 | 29.2 | 1.13 | 0.98 | 0.48 | 0.75 | 0.91 | 0.77 | 0.53 | 0.86 | 0.9 | 0.78 |
|  |  | Rye | 0 | 12.2 | 1.42 | 135 | 1.13 | 390 | 34.4 | 45.9 | 0.84 | 0.89 | 0.89 | 0.96 | 1.24 | 1.12 | 0.72 | 0.81 | 1.03 | 0.93 |
|  |  |  | 15 | 8.4 | 1 | 92 | 1.13 | 283 | 26.1 | 33.9 | 0.76 | 0.77 | 0.88 | 1.12 | 1.46 | 1.27 | 0.65 | 0.78 | 1.03 | 0.91 |
|  | SS | No | 0 | 12.6 | 1.43 | 310 | 2.5 | 464 | 36.2 | 62.6 | 0.86 | 0.9 | 1.98 | 1.13 | 1.27 | 1.49 | 1.68 | 0.98 | 1.08 | 1.27 |
|  |  |  | 15 | 8.2 | 1.02 | 145 | 1.78 | 285 | 21.6 | 33.9 | 0.75 | 0.78 | 1.4 | 1.06 | 1.23 | 1.29 | 1.03 | 0.79 | 0.86 | 0.91 |
|  |  | Rye | 0 | 14.1 | 1.51 | 281 | 2.05 | 498 | 37.9 | 61.9 | 0.96 | 0.95 | 1.61 | 1.11 | 1.24 | 1.36 | 1.52 | 1.04 | 1.13 | 1.25 |
|  |  |  | 15 | 11.1 | 1.09 | 158 | 1.9 | 317 | 26.5 | 40 | 1.01 | 0.84 | 1.53 | 0.92 | 1.24 | 1.33 | 1.14 | 0.87 | 1.05 | 1.08 |
| 1.1 | CS | No | 0 | 16.5 | 1.71 | 260 | 1.68 | 522 | 27.7 | 49.9 | 1.13 | 1.07 | 1.33 | 0.99 | 0.76 | 0.94 | 1.39 | 1.09 | 0.83 | 1.01 |
|  |  |  | 15 | 12.4 | 1.39 | 143 | 1.2 | 378 | 24 | 36.2 | 1.12 | 1.07 | 0.94 | 0.93 | 0.91 | 0.92 | 1.01 | 1.04 | 0.95 | 0.97 |
|  |  | Rye | 0 | 16.9 | 1.78 | 210 | 1.33 | 554 | 36.4 | 54.3 | 1.16 | 1.11 | 1.01 | 1.01 | 0.94 | 0.96 | 1.12 | 1.16 | 1.09 | 1.09 |
|  |  |  | 15 | 10.8 | 1.35 | 176 | 1.73 | 354 | 26.8 | 41.7 | 0.99 | 1.04 | 1.37 | 1.02 | 1.16 | 1.22 | 1.25 | 0.98 | 1.06 | 1.12 |
|  | SC | No | 0 | 12.6 | 1.41 | 234 | 1.93 | 471 | 26.6 | 46.5 | 0.86 | 0.89 | 1.53 | 1.15 | 0.94 | 1.13 | 1.26 | 0.98 | 0.79 | 0.94 |
|  |  |  | 15 | 7.4 | 0.97 | 183 | 3.13 | 236 | 20.3 | 35.8 | 0.67 | 0.75 | 2.51 | 1.02 | 1.3 | 1.67 | 1.33 | 0.65 | 0.8 | 0.96 |
|  |  | Rye | 0 | 14.1 | 1.53 | 221 | 1.58 | 436 | 32.2 | 51 | 0.97 | 0.96 | 1.22 | 0.94 | 1.05 | 1.1 | 1.18 | 0.91 | 0.97 | 1.03 |
|  |  |  | 15 | 10.8 | 1.2 | 153 | 1.43 | 280 | 21.3 | 34.3 | 0.98 | 0.92 | 1.12 | 0.79 | 0.94 | 1 | 1.09 | 0.77 | 0.84 | 0.92 |
|  | SS | No | 0 | 14 | 1.49 | 281 | 2.03 | 467 | 32.2 | 56.1 | 0.96 | 0.93 | 1.59 | 1.04 | 1 | 1.19 | 1.52 | 0.97 | 0.96 | 1.14 |
|  |  |  | 15 | 10.3 | 1.19 | 130 | 1.33 | 325 | 22.7 | 33.7 | 0.94 | 0.91 | 1.03 | 0.99 | 1.04 | 1.03 | 0.93 | 0.9 | 0.9 | 0.91 |
|  |  | Rye | 0 | 13.7 | 1.49 | 346 | 2.55 | 487 | 30.1 | 59.6 | 0.94 | 0.93 | 2 | 1.09 | 0.96 | 1.29 | 1.87 | 1.02 | 0.9 | 1.21 |
|  |  |  | 15 | 9.5 | 1.11 | 195 | 2.15 | 324 | 21.8 | 38.5 | 0.86 | 0.85 | 1.71 | 1.06 | 1.21 | 1.36 | 1.4 | 0.9 | 0.87 | 1.04 |
| 2.2 | CS | No | 0 | 15.5 | 1.66 | 270 | 1.8 | 522 | 43.9 | 66.9 | 1.06 | 1.04 | 1.42 | 1.05 | 1.24 | 1.29 | 1.45 | 1.09 | 1.31 | 1.35 |
|  |  |  | 15 | 11.9 | 1.34 | 209 | 2.1 | 367 | 25 | 42.8 | 1.08 | 1.03 | 1.68 | 0.99 | 1.02 | 1.22 | 1.49 | 1.01 | 0.99 | 1.15 |
|  |  | Rye | 0 | 17.9 | 1.99 | 392 | 2.25 | 534 | 37.3 | 70.6 | 1.23 | 1.24 | 1.77 | 0.92 | 0.94 | 1.21 | 2.11 | 1.11 | 1.11 | 1.43 |
|  |  |  | 15 | 12 | 1.31 | 256 | 2.2 | 383 | 27.5 | 49.3 | 1.09 | 1 | 1.74 | 0.99 | 1.03 | 1.26 | 1.83 | 1.06 | 1.09 | 1.32 |
|  | SC | No | 0 | 13.6 | 1.68 | 295 | 2.18 | 429 | 34.7 | 59.8 | 0.93 | 1.05 | 1.72 | 0.96 | 1.16 | 1.33 | 1.59 | 0.89 | 1.04 | 1.21 |
|  |  |  | 15 | 10.4 | 1.21 | 184 | 1.78 | 308 | 24.9 | 40.6 | 0.95 | 0.93 | 1.4 | 0.93 | 1.21 | 1.27 | 1.31 | 0.85 | 0.99 | 1.09 |
|  |  | Rye | 0 | 14.3 | 1.52 | 393 | 2.83 | 442 | 30.9 | 64.3 | 0.98 | 0.95 | 2.23 | 0.95 | 0.93 | 1.34 | 2.13 | 0.92 | 0.93 | 1.31 |
|  |  |  | 15 | 10.4 | 1.14 | 300 | 3.05 | 327 | 18.1 | 43.7 | 0.94 | 0.88 | 2.44 | 0.97 | 0.76 | 1.28 | 2.17 | 0.9 | 0.72 | 1.17 |
|  | SS | No | 0 | 13.7 | 1.49 | 345 | 2.7 | 461 | 34.7 | 64.1 | 0.94 | 0.93 | 2.13 | 1.04 | 1.14 | 1.45 | 1.87 | 0.96 | 1.03 | 1.3 |
|  |  |  | 15 | 9.2 | 1.1 | 238 | 3.08 | 328 | 23.9 | 44.2 | 0.83 | 0.85 | 2.49 | 1.15 | 1.31 | 1.67 | 1.73 | 0.91 | 0.94 | 1.18 |
|  |  | Rye | 0 | 13.7 | 1.5 | 450 | 3.35 | 468 | 44 | 82.4 | 0.94 | 0.94 | 2.67 | 1.06 | 1.45 | 1.82 | 2.46 | 0.98 | 1.32 | 1.67 |
|  |  |  | 15 | 9 | 1.04 | 223 | 2.6 | 288 | 27.8 | 46.8 | 0.82 | 0.8 | 2.08 | 1.05 | 1.57 | 1.73 | 1.6 | 0.8 | 1.1 | 1.26 |
| **Probability > F** | | |  |  |  |  |  |  |  |  |  |  |  |  |  |  |  |  |  |  |
| Gypsum | | |  | 0.91 | 0.89 | 0.001 | 0.001 | 0.99 | 0.07 | 0.001 | 0.9 | 0.91 | 0.001 | 0.98 | 0.39 | 0.001 | 0.001 | 0.97 | 0.1 | 0.001 |
| Crop rotation (CR) | | |  | 0.001 | 0.001 | 0.001 | 0.001 | 0.001 | 0.36 | 0.001 | 0.001 | 0.001 | 0.001 | 0.02 | 0.01 | 0.001 | 0.001 | 0.001 | 0.38 | 0.001 |
| Cover crop (CC) | | |  | 0.69 | 0.93 | 0.02 | 0.44 | 0.96 | 0.47 | 0.03 | 0.76 | 0.85 | 0.44 | 0.58 | 0.72 | 0.48 | 0.02 | 0.99 | 0.45 | 0.03 |
| Soil depth | | |  | 0.001 | 0.001 | 0.001 | 0.52 | 0.001 | 0.001 | 0.001 | 0.23 | 0.02 | 0.61 | 0.21 | 0.38 | 0.7 | 0.001 | 0.001 | 0.2 | 0.001 |
| Gypsum x CR | | |  | 0.03 | 0.14 | 0.91 | 0.79 | 0.91 | 0.08 | 0.01 | 0.91 | 0.32 | 0.05 | 0.22 | 0.91 | 0.79 | 0.04 | 0.89 | 0.1 | 0.01 |
| Gypsum x CC | | |  | 0.01 | 0.28 | 0.78 | 0.63 | 0.88 | 0.77 | 0.36 | 0.8 | 0.7 | 0.82 | 0.64 | 0.54 | 0.64 | 0.02 | 0.91 | 0.79 | 0.43 |
| Gypsum x depth | | |  | 0.35 | 0.85 | 0.9 | 0.78 | 0.64 | 0.38 | 0.11 | 0.89 | 0.34 | 0.8 | 0.3 | 0.36 | 0.81 | 0.99 | 0.64 | 0.55 | 0.56 |
| CR x CC | | |  | 0.81 | 0.9 | 0.91 | 0.83 | 0.82 | 0.73 | 0.63 | 0.89 | 0.34 | 0.91 | 0.22 | 0.4 | 0.88 | 0.83 | 0.9 | 0.71 | 0.66 |
| CR x depth | | |  | 0.01 | 0.35 | 0.51 | 0.94 | 0.85 | 0.65 | 0.02 | 0.84 | 0.001 | 0.09 | 0.16 | 0.01 | 0.96 | 0.04 | 0.87 | 0.68 | 0.09 |
| CC x depth | | |  | 0.72 | 0.89 | 0.59 | 0.35 | 0.98 | 0.98 | 0.81 | 0.64 | 0.07 | 0.09 | 0.57 | 0.14 | 0.37 | 0.97 | 0.99 | 0.94 | 0.95 |
| Gypsum x CR x CC | | |  | 0.27 | 0.03 | 0.11 | 0.39 | 0.67 | 0.33 | 0.82 | 0.1 | 0.1 | 0.27 | 0.22 | 0.18 | 0.43 | 0.2 | 0.71 | 0.35 | 0.81 |
| Gypsum x CR x depth | | |  | 0.99 | 0.82 | 0.98 | 0.99 | 0.79 | 0.81 | 0.82 | 0.97 | 0.39 | 0.52 | 0.27 | 0.47 | 0.98 | 1 | 0.75 | 0.93 | 0.96 |
| Gypsum x CC x depth | | |  | 0.32 | 0.78 | 0.94 | 0.91 | 0.94 | 0.75 | 0.57 | 0.95 | 0.59 | 0.92 | 0.85 | 0.58 | 0.88 | 0.52 | 0.96 | 0.78 | 0.68 |
| CR x CC x depth | | |  | 0.87 | 0.92 | 0.78 | 0.64 | 0.5 | 0.92 | 0.87 | 0.78 | 0.48 | 0.66 | 0.34 | 0.63 | 0.68 | 0.87 | 0.56 | 0.91 | 0.91 |
| Gypsum x CR x CC x depth | | | | 0.54 | 0.24 | 0.88 | 0.93 | 0.94 | 0.81 | 0.7 | 0.75 | 0.46 | 0.84 | 0.34 | 0.44 | 0.91 | 0.41 | 0.92 | 0.85 | 0.68 |
