# Supplementary material for: Gypsum, crop rotation, and cover crop impacts on soil organic carbon and biological dynamics in rainfed transitional no-till corn-soybean systems
Source: PLoS One. 2022 Sep 27;17(9):e0275198. doi: 10.1371/journal.pone.0275198 (PMC9514652; doi:10.1371/journal.pone.0275198)
Supplement: S4 Table — (DOCX) [file pone.0275198.s005.docx]

**S4 Table.** Interactive effects of gypsum, crop rotation, and cover crop on total soil organic C (SOC), total nitrogen (TN), microbial biomass (SBM), metabolic quotient (qR), active carbon (AC), cold (CWC) and hot (HWC) salt water extractable carbon, carbon pool index (CPI), nitrogen pool index (NPI), carbon lability index (CLI) and carbon management index (CMI) under a rainfed transitioning no-till soybean-corn rotation at Piketon site (2012 to 2016).

| Gypsum | Crop | Cover | Depth | SOC | TN | SMBC | SMBC: | AC | CWC | HWC | CPI | NPI | CLI | | | | CMI | | | |
| --- | --- | --- | --- | --- | --- | --- | --- | --- | --- | --- | --- | --- | --- | --- | --- | --- | --- | --- | --- | --- |
| (Mg/ha) | rotation | crop | (cm) | (g/kg) | | (mg/kg) | SOC(%) | (mg/kg) | | |  |  | SMBC | AC | CWC | HWC | SMBC | AC | CWC | HWC |
| 0 | CS | No | 0 | 10.6 | 1.19 | 330 | 3.18 | 491 | 18.1 | 88.4 | 1.15 | 1.09 | 0.84 | 0.89 | 0.64 | 0.8 | 0.95 | 1.02 | 0.73 | 0.91 |
|  |  |  | 15 | 4.6 | 0.64 | 121 | 2.83 | 319 | 11.2 | 37 | 1.08 | 0.91 | 0.75 | 1.35 | 1.07 | 0.84 | 0.74 | 1.45 | 0.97 | 0.81 |
|  |  | Rye | 0 | 10.9 | 1.23 | 262 | 2.45 | 517 | 18.5 | 74.3 | 1.19 | 1.11 | 0.65 | 0.92 | 0.62 | 0.66 | 0.75 | 1.08 | 0.74 | 0.76 |
|  |  |  | 15 | 4.6 | 0.63 | 123 | 2.78 | 322 | 11.4 | 37.6 | 1.07 | 0.9 | 0.73 | 1.42 | 0.95 | 0.8 | 0.75 | 1.47 | 0.98 | 0.82 |
|  | SC | No | 0 | 7.8 | 0.94 | 359 | 4.43 | 460 | 18 | 94.4 | 0.85 | 0.85 | 1.2 | 1.16 | 0.88 | 1.12 | 1.05 | 0.97 | 0.73 | 0.97 |
|  |  |  | 15 | 5.1 | 0.69 | 144 | 2.78 | 312 | 11.9 | 42.5 | 1.19 | 0.99 | 0.73 | 1.2 | 0.89 | 0.78 | 0.88 | 1.41 | 1.03 | 0.93 |
|  |  | Rye | 0 | 8.6 | 1.03 | 294 | 3.5 | 458 | 19.5 | 82.1 | 0.93 | 0.93 | 0.94 | 1.05 | 0.86 | 0.93 | 0.86 | 0.96 | 0.78 | 0.84 |
|  |  |  | 15 | 4.4 | 0.59 | 117 | 2.8 | 336 | 13.1 | 38 | 1.01 | 0.85 | 0.74 | 1.54 | 1.16 | 0.86 | 0.72 | 1.54 | 1.13 | 0.83 |
|  | SS | No | 0 | 9.4 | 1.05 | 279 | 3.2 | 269 | 16.2 | 75.7 | 1.02 | 0.96 | 0.86 | 0.55 | 0.67 | 0.81 | 0.81 | 0.55 | 0.65 | 0.77 |
|  |  |  | 15 | 3.1 | 0.65 | 150 | 5.65 | 125 | 13.1 | 45.1 | 0.72 | 0.92 | 1.57 | 0.81 | 1.88 | 1.63 | 0.96 | 0.55 | 1.13 | 1 |
|  |  | Rye | 0 | 9.6 | 1.08 | 187 | 2.08 | 267 | 17.5 | 57.2 | 1.05 | 0.98 | 0.55 | 0.54 | 0.7 | 0.6 | 0.53 | 0.54 | 0.7 | 0.58 |
|  |  |  | 15 | 4.3 | 0.55 | 95 | 2.65 | 179 | 12.4 | 32.6 | 0.99 | 0.79 | 0.7 | 0.84 | 1.26 | 0.85 | 0.58 | 0.8 | 1.07 | 0.71 |
| 1.1 | CS | No | 0 | 12.1 | 1.31 | 249 | 2.08 | 557 | 19 | 72.1 | 1.32 | 1.19 | 0.55 | 0.89 | 0.59 | 0.57 | 0.71 | 1.16 | 0.76 | 0.74 |
|  |  |  | 15 | 4.3 | 0.58 | 115 | 2.7 | 308 | 13.7 | 38.2 | 1 | 0.84 | 0.71 | 1.39 | 1.23 | 0.86 | 0.71 | 1.41 | 1.18 | 0.84 |
|  |  | Rye | 0 | 11.5 | 1.28 | 286 | 2.48 | 552 | 15.3 | 76.1 | 1.25 | 1.16 | 0.66 | 0.92 | 0.49 | 0.62 | 0.82 | 1.14 | 0.61 | 0.78 |
|  |  |  | 15 | 4.4 | 0.6 | 120 | 2.83 | 362 | 12.2 | 37.7 | 1.01 | 0.86 | 0.74 | 1.65 | 1.08 | 0.84 | 0.73 | 1.67 | 1.05 | 0.82 |
|  | SC | No | 0 | 9.8 | 1.09 | 370 | 3.75 | 465 | 19.1 | 97.9 | 1.06 | 1 | 1.01 | 0.92 | 0.73 | 0.95 | 1.07 | 0.97 | 0.77 | 1 |
|  |  |  | 15 | 4.8 | 0.62 | 133 | 2.83 | 355 | 14.3 | 42.7 | 1.12 | 0.89 | 0.74 | 1.51 | 1.11 | 0.85 | 0.81 | 1.62 | 1.23 | 0.94 |
|  |  | Rye | 0 | 9.7 | 1.1 | 326 | 3.33 | 462 | 14.8 | 84.3 | 1.06 | 1 | 0.88 | 0.92 | 0.57 | 0.81 | 0.94 | 0.96 | 0.6 | 0.87 |
|  |  |  | 15 | 3.7 | 0.56 | 160 | 5.68 | 269 | 11.7 | 45.8 | 0.85 | 0.8 | 1.59 | 1.31 | 1.47 | 1.54 | 1.01 | 1.23 | 1.01 | 1.01 |
|  | SS | No | 0 | 9.1 | 1.03 | 176 | 1.98 | 389 | 13.9 | 51.5 | 0.98 | 0.94 | 0.52 | 0.88 | 0.59 | 0.54 | 0.5 | 0.81 | 0.56 | 0.53 |
|  |  |  | 15 | 3 | 0.53 | 142 | 5.3 | 121 | 12.2 | 42.5 | 0.69 | 0.76 | 1.45 | 0.78 | 1.68 | 1.51 | 0.9 | 0.53 | 1.05 | 0.94 |
|  |  | Rye | 0 | 8.2 | 0.99 | 208 | 2.63 | 423 | 18.1 | 62.2 | 0.89 | 0.89 | 0.7 | 1.01 | 0.83 | 0.74 | 0.6 | 0.88 | 0.72 | 0.64 |
|  |  |  | 15 | 2.6 | 0.48 | 132 | 5.1 | 155 | 12.8 | 40.8 | 0.6 | 0.69 | 1.38 | 1.18 | 1.87 | 1.51 | 0.83 | 0.7 | 1.11 | 0.9 |
| 2.2 | CS | No | 0 | 9.5 | 1.09 | 291 | 3.13 | 557 | 17.6 | 79.6 | 1.03 | 1 | 0.84 | 1.18 | 0.73 | 0.82 | 0.84 | 1.18 | 0.71 | 0.82 |
|  |  |  | 15 | 4.9 | 0.66 | 105 | 2.23 | 376 | 11.2 | 33.6 | 1.14 | 0.95 | 0.58 | 1.54 | 0.89 | 0.67 | 0.64 | 1.73 | 0.97 | 0.74 |
|  |  | Rye | 0 | 11 | 1.18 | 301 | 2.88 | 577 | 17.6 | 81.7 | 1.19 | 1.07 | 0.75 | 1.07 | 0.64 | 0.73 | 0.86 | 1.21 | 0.71 | 0.83 |
|  |  |  | 15 | 4 | 0.58 | 118 | 2.93 | 337 | 13.2 | 38.2 | 0.94 | 0.83 | 0.77 | 1.67 | 1.24 | 0.9 | 0.72 | 1.56 | 1.15 | 0.84 |
|  | SC | No | 0 | 8.9 | 1.04 | 378 | 4.4 | 452 | 20.3 | 100.8 | 0.97 | 0.95 | 1.19 | 1 | 0.89 | 1.12 | 1.11 | 0.95 | 0.82 | 1.04 |
|  |  |  | 15 | 4.2 | 0.6 | 141 | 3.43 | 325 | 13.1 | 43 | 0.97 | 0.86 | 0.91 | 1.53 | 1.19 | 0.99 | 0.87 | 1.49 | 1.13 | 0.94 |
|  |  | Rye | 0 | 10 | 1.13 | 273 | 2.73 | 480 | 18.2 | 76.4 | 1.09 | 1.03 | 0.73 | 0.94 | 0.68 | 0.72 | 0.79 | 1 | 0.74 | 0.78 |
|  |  |  | 15 | 4 | 0.59 | 94 | 2.4 | 321 | 14.4 | 34.3 | 0.93 | 0.85 | 0.63 | 1.6 | 1.41 | 0.84 | 0.57 | 1.48 | 1.24 | 0.75 |
|  | SS | No | 0 | 9.3 | 1.26 | 262 | 2.83 | 497 | 18 | 73.7 | 1.01 | 1.15 | 0.75 | 1.09 | 0.74 | 0.76 | 0.75 | 1.04 | 0.72 | 0.76 |
|  |  |  | 15 | 4.3 | 0.63 | 124 | 3.55 | 278 | 12.3 | 38.8 | 1 | 0.89 | 0.95 | 1.38 | 1.16 | 1.01 | 0.77 | 1.27 | 1.07 | 0.85 |
|  |  | Rye | 0 | 9.7 | 1.12 | 262 | 2.7 | 553 | 17.7 | 73.5 | 1.05 | 1.02 | 0.71 | 1.13 | 0.67 | 0.72 | 0.75 | 1.16 | 0.71 | 0.76 |
|  |  |  | 15 | 4.1 | 0.63 | 105 | 2.95 | 261 | 15.9 | 38.2 | 0.94 | 0.9 | 0.79 | 1.5 | 1.8 | 1.05 | 0.65 | 1.19 | 1.37 | 0.84 |
| **Probability > F** | | |  |  |  |  |  |  |  |  |  |  |  |  |  |  |  |  |  |  |
| Gypsum | | |  | 0.97 | 0.68 | 0.97 | 0.47 | 0.001 | 0.23 | 0.9 | 0.71 | 0.56 | 0.45 | 0.001 | 0.78 | 0.55 | 0.89 | 0.001 | 0.24 | 1 |
| Crop rotation (CR) | | |  | 0.001 | 0.02 | 0.002 | 0.02 | 0.001 | 0.38 | 0.002 | 0.001 | 0.04 | 0.02 | 0.001 | 0.002 | 0.01 | 0.01 | 0.001 | 0.41 | 0.01 |
| Cover crop (CC) | | |  | 0.93 | 0.61 | 0.09 | 0.23 | 0.32 | 0.92 | 0.12 | 0.72 | 0.42 | 0.25 | 0.16 | 0.52 | 0.44 | 0.06 | 0.53 | 0.71 | 0.09 |
| Soil depth | | |  | 0.001 | 0.001 | 0.001 | 0.09 | 0.001 | 0.001 | 0.001 | 0.01 | 0.001 | 0.1 | 0.001 | 0.001 | 0.001 | 0.31 | 0.001 | 0.001 | 0.09 |
| Gypsum x CR | | |  | 0.76 | 0.64 | 0.09 | 0.11 | 0 | 0.76 | 0.8 | 0.16 | 0.17 | 0.64 | 0.001 | 0.85 | 0.71 | 0.77 | 0.001 | 0.73 | 0.82 |
| Gypsum x CC | | |  | 0.21 | 0.04 | 0.32 | 0.95 | 0.86 | 0.2 | 0.37 | 0.43 | 0.96 | 0.04 | 0.8 | 0.4 | 0.08 | 0.12 | 0.72 | 0.12 | 0.28 |
| Gypsum x depth | | |  | 0.39 | 0.03 | 0.14 | 0.26 | 0.01 | 0.32 | 0.35 | 0.13 | 0.22 | 0.03 | 0.5 | 0.17 | 0.03 | 0.21 | 0.23 | 0.35 | 0.23 |
| CR x CC | | |  | 0.43 | 0.44 | 0.98 | 0.64 | 0.38 | 0.15 | 0.43 | 0.8 | 0.75 | 0.44 | 0.59 | 0.81 | 0.65 | 0.33 | 0.47 | 0.28 | 0.39 |
| CR x depth | | |  | 0.01 | 0 | 0.02 | 0.08 | 0.01 | 0.32 | 0.01 | 0.11 | 0.2 | 0.001 | 0.01 | 0.01 | 0.001 | 0.04 | 0.001 | 0.34 | 0.04 |
| CC x depth | | |  | 0.45 | 0.5 | 0.24 | 0.3 | 0.47 | 0.43 | 0.4 | 0.23 | 0.26 | 0.49 | 0.11 | 0.22 | 0.37 | 0.83 | 0.86 | 0.42 | 0.71 |
| Gypsum x CR x CC | | |  | 0.8 | 0.3 | 0.94 | 0.98 | 0.57 | 0.2 | 0.71 | 0.83 | 0.98 | 0.3 | 0.52 | 0.39 | 0.27 | 0.62 | 0.48 | 0.34 | 0.55 |
| Gypsum x CR x depth | | |  | 0.87 | 0.73 | 0.36 | 0.52 | 0.1 | 0.96 | 0.91 | 0.53 | 0.76 | 0.73 | 0.28 | 0.82 | 0.72 | 0.92 | 0.59 | 0.92 | 0.97 |
| Gypsum x CC x depth | | |  | 0.74 | 0.81 | 0.45 | 0.73 | 0.29 | 0.27 | 0.81 | 0.6 | 0.73 | 0.77 | 0.93 | 0.12 | 0.61 | 0.96 | 0.41 | 0.15 | 0.97 |
| CR x CC x depth | | |  | 0.62 | 0.09 | 0.45 | 0.64 | 0.85 | 0.64 | 0.57 | 0.37 | 0.71 | 0.08 | 0.98 | 0.43 | 0.1 | 0.49 | 0.74 | 0.97 | 0.56 |
| Gypsum x CR x CC x depth | | | | 0.93 | 0.69 | 0.87 | 0.76 | 0.44 | 0.78 | 0.93 | 0.74 | 0.76 | 0.66 | 0.48 | 0.62 | 0.65 | 0.87 | 0.41 | 0.93 | 0.91 |
