# Supplementary material for: Gypsum, crop rotation, and cover crop impacts on soil organic carbon and biological dynamics in rainfed transitional no-till corn-soybean systems
Source: PLoS One. 2022 Sep 27;17(9):e0275198. doi: 10.1371/journal.pone.0275198 (PMC9514652; doi:10.1371/journal.pone.0275198)
Supplement: S5 Table — (DOCX) [file pone.0275198.s006.docx]

**S5 Table.** Interactive effects of gypsum, crop rotation, and cover crop on normalized values of on total soil organic C (SOC), total nitrogen (TN), microbial biomass (SBM), metabolic quotient (qR), active carbon (AC), cold (CWC) and hot (HWC) salt water extractable carbon, carbon pool index (CPI), nitrogen pool index (NPI), carbon lability index (CLI) and carbon management index (CMI) under a rainfed transitioning no-till soybean-corn rotation, averaged across sites (2012 to 2016).

| Gypsum | Crop | Cover | Depth | SOC | TN | SMBC | SMBC: | AC | CWC | HWC | CPI | NPI | CLI | | | | CMI | | | |
| --- | --- | --- | --- | --- | --- | --- | --- | --- | --- | --- | --- | --- | --- | --- | --- | --- | --- | --- | --- | --- |
| (Mg/ha) | rotation | crop | (cm) | (g/kg) | | (mg/kg) | SOC(%) | (mg/kg) | | |  |  | SMBC | AC | CWC | HWC | SMBC | AC | CWC | HWC |
| 0 | CS | No | 0 | 1.35 | 1.21 | 1.09 | 0.77 | 1.27 | 1.19 | 1.13 | 1.12 | 1.08 | 0.76 | 0.95 | 0.85 | 0.8 | 0.91 | 1.04 | 0.98 | 0.95 |
|  |  |  | 15 | 0.87 | 0.87 | 0.57 | 0.65 | 0.88 | 0.91 | 0.69 | 1.1 | 0.99 | 0.64 | 1.01 | 1 | 0.76 | 0.71 | 1.11 | 1.13 | 0.86 |
|  |  | Rye | 0 | 1.4 | 1.25 | 0.93 | 0.64 | 1.32 | 1.24 | 1.02 | 1.16 | 1.11 | 0.64 | 0.94 | 0.88 | 0.72 | 0.78 | 1.09 | 1.04 | 0.86 |
|  |  |  | 15 | 0.92 | 0.86 | 0.65 | 0.68 | 0.98 | 0.75 | 0.71 | 1.15 | 0.99 | 0.67 | 1.09 | 0.78 | 0.73 | 0.81 | 1.23 | 0.95 | 0.9 |
|  | SC | No | 0 | 1.16 | 1.11 | 1.15 | 0.91 | 1.23 | 1.15 | 1.2 | 0.98 | 0.99 | 0.91 | 1.04 | 0.94 | 0.95 | 0.96 | 1.02 | 0.95 | 0.99 |
|  |  |  | 15 | 0.89 | 0.87 | 0.67 | 0.7 | 0.93 | 0.77 | 0.72 | 1.12 | 1.01 | 0.7 | 1.02 | 0.82 | 0.77 | 0.84 | 1.18 | 0.95 | 0.9 |
|  |  | Rye | 0 | 1.13 | 1.11 | 1.06 | 0.87 | 1.19 | 1.29 | 1.14 | 0.95 | 0.99 | 0.87 | 1.03 | 1.09 | 0.95 | 0.88 | 0.98 | 1.09 | 0.95 |
|  |  |  | 15 | 0.74 | 0.78 | 0.69 | 0.94 | 0.91 | 0.88 | 0.72 | 0.92 | 0.89 | 0.93 | 1.29 | 1.18 | 0.98 | 0.87 | 1.16 | 1.1 | 0.9 |
|  | SS | No | 0 | 1.09 | 1.11 | 1.53 | 1.35 | 1.03 | 1.18 | 1.36 | 0.92 | 0.98 | 1.37 | 0.95 | 1.04 | 1.2 | 1.32 | 0.88 | 0.99 | 1.15 |
|  |  |  | 15 | 0.64 | 0.82 | 0.99 | 1.61 | 0.67 | 0.8 | 0.9 | 0.78 | 0.95 | 1.63 | 1.03 | 1.38 | 1.52 | 1.26 | 0.8 | 1.01 | 1.15 |
|  |  | Rye | 0 | 1.16 | 1.19 | 1.3 | 1.09 | 1.07 | 1.28 | 1.24 | 0.97 | 1.05 | 1.1 | 0.94 | 1.06 | 1.04 | 1.12 | 0.9 | 1.07 | 1.05 |
|  |  |  | 15 | 0.76 | 0.78 | 0.89 | 1.36 | 0.75 | 0.77 | 0.8 | 0.93 | 0.9 | 1.38 | 1.06 | 1.11 | 1.21 | 1.1 | 0.91 | 0.96 | 0.99 |
| 1.1 | CS | No | 0 | 1.43 | 1.25 | 1.55 | 1.08 | 1.43 | 1.24 | 1.38 | 1.19 | 1.1 | 1.09 | 0.98 | 0.84 | 0.95 | 1.33 | 1.17 | 1.02 | 1.17 |
|  |  |  | 15 | 0.89 | 0.87 | 0.99 | 1.05 | 0.95 | 0.95 | 0.95 | 1.1 | 1 | 1.04 | 1.14 | 1.1 | 1.03 | 1.21 | 1.2 | 1.18 | 1.17 |
|  |  | Rye | 0 | 1.42 | 1.24 | 1.22 | 0.82 | 1.47 | 1.41 | 1.26 | 1.18 | 1.1 | 0.81 | 1.02 | 0.96 | 0.84 | 1.01 | 1.2 | 1.17 | 1.05 |
|  |  |  | 15 | 0.85 | 0.86 | 0.81 | 1.01 | 1 | 0.97 | 0.83 | 1.06 | 0.99 | 1.02 | 1.22 | 1.13 | 1.01 | 1.03 | 1.28 | 1.19 | 1.03 |
|  | SC | No | 0 | 1.18 | 1.06 | 1.41 | 1.19 | 1.2 | 1.26 | 1.3 | 0.98 | 0.94 | 1.19 | 1.03 | 1.04 | 1.08 | 1.18 | 0.99 | 1.05 | 1.08 |
|  |  |  | 15 | 0.78 | 0.77 | 0.89 | 1.28 | 0.87 | 0.98 | 0.86 | 1 | 0.89 | 1.29 | 1.15 | 1.19 | 1.13 | 1.13 | 1.12 | 1.23 | 1.08 |
|  |  | Rye | 0 | 1.25 | 1.15 | 1.37 | 1.04 | 1.23 | 1.27 | 1.33 | 1.04 | 1.02 | 1.03 | 0.97 | 1.01 | 1.02 | 1.16 | 1.02 | 1.07 | 1.13 |
|  |  |  | 15 | 0.8 | 0.8 | 0.77 | 1.02 | 0.87 | 0.82 | 0.77 | 0.99 | 0.92 | 1.04 | 1.07 | 1.07 | 1.04 | 0.99 | 1.09 | 1.02 | 0.98 |
|  | SS | No | 0 | 1.19 | 1.15 | 1.32 | 1.08 | 1.13 | 1.25 | 1.23 | 1 | 1.02 | 1.07 | 0.95 | 1.02 | 1 | 1.14 | 0.94 | 1.05 | 1.05 |
|  |  |  | 15 | 0.73 | 0.84 | 0.83 | 1.23 | 0.68 | 0.91 | 0.83 | 0.88 | 0.96 | 1.24 | 0.95 | 1.38 | 1.24 | 1.06 | 0.82 | 1.12 | 1.04 |
|  |  | Rye | 0 | 1.08 | 1.08 | 1.41 | 1.22 | 1.17 | 1.3 | 1.26 | 0.91 | 0.96 | 1.22 | 1.05 | 1.12 | 1.09 | 1.21 | 0.98 | 1.09 | 1.07 |
|  |  |  | 15 | 0.62 | 0.75 | 1.02 | 1.91 | 0.72 | 0.92 | 0.93 | 0.74 | 0.86 | 1.98 | 1.32 | 1.8 | 1.8 | 1.28 | 0.87 | 1.14 | 1.15 |
| 2.2 | CS | No | 0 | 1.32 | 1.16 | 1.67 | 1.24 | 1.54 | 1.4 | 1.56 | 1.1 | 1.03 | 1.25 | 1.15 | 1.07 | 1.17 | 1.44 | 1.25 | 1.19 | 1.33 |
|  |  |  | 15 | 0.89 | 0.88 | 1.1 | 1.23 | 1 | 0.94 | 0.99 | 1.12 | 1.01 | 1.25 | 1.15 | 1 | 1.08 | 1.35 | 1.29 | 1.15 | 1.2 |
|  |  | Rye | 0 | 1.43 | 1.33 | 1.8 | 1.21 | 1.65 | 1.24 | 1.48 | 1.18 | 1.18 | 1.21 | 1.13 | 0.89 | 1.02 | 1.53 | 1.34 | 1.05 | 1.25 |
|  |  |  | 15 | 0.85 | 0.86 | 1.09 | 1.15 | 1.08 | 0.88 | 0.95 | 1.07 | 0.99 | 1.16 | 1.29 | 0.99 | 1.03 | 1.36 | 1.39 | 1.08 | 1.18 |
|  | SC | No | 0 | 1.16 | 1.09 | 1.67 | 1.36 | 1.21 | 1.23 | 1.47 | 0.98 | 0.97 | 1.38 | 1.02 | 1 | 1.2 | 1.43 | 1 | 1.02 | 1.23 |
|  |  |  | 15 | 0.81 | 0.83 | 1.01 | 1.16 | 0.96 | 0.88 | 0.9 | 1.01 | 0.95 | 1.15 | 1.22 | 1.09 | 1.08 | 1.23 | 1.23 | 1.1 | 1.11 |
|  |  | Rye | 0 | 1.29 | 1.39 | 1.6 | 1.22 | 1.29 | 1.24 | 1.35 | 1.07 | 1.22 | 1.22 | 1.01 | 0.97 | 1.02 | 1.36 | 1.07 | 1.06 | 1.14 |
|  |  |  | 15 | 0.86 | 0.91 | 1.09 | 1.26 | 0.98 | 0.77 | 0.86 | 1.07 | 1.06 | 1.27 | 1.26 | 0.98 | 1.01 | 1.33 | 1.24 | 0.97 | 1.06 |
|  | SS | No | 0 | 1.09 | 1.14 | 1.61 | 1.49 | 1.18 | 1.26 | 1.4 | 0.92 | 1.01 | 1.5 | 1.08 | 1.14 | 1.29 | 1.4 | 0.98 | 1.04 | 1.19 |
|  |  |  | 15 | 0.73 | 0.82 | 1.1 | 1.57 | 0.82 | 0.8 | 0.94 | 0.9 | 0.94 | 1.6 | 1.18 | 1.15 | 1.34 | 1.37 | 1.03 | 1.01 | 1.17 |
|  |  | Rye | 0 | 1.16 | 1.33 | 1.71 | 1.47 | 1.22 | 1.48 | 1.51 | 0.97 | 1.17 | 1.48 | 1.04 | 1.26 | 1.3 | 1.48 | 1.01 | 1.23 | 1.28 |
|  |  |  | 15 | 0.74 | 0.88 | 0.97 | 1.43 | 0.82 | 1.07 | 0.93 | 0.91 | 1.02 | 1.46 | 1.22 | 1.64 | 1.42 | 1.22 | 1.02 | 1.34 | 1.17 |
| Probability > F | | |  |  |  |  |  |  |  |  |  |  |  |  |  |  |  |  |  |  |
| Gypsum | | |  | 0.92 | 0.21 | <.001 | 0.002 | 0.02 | 0.03 | <.001 | 0.76 | 0.03 | 0.001 | 0.03 | 0.17 | 0.02 | <.001 | 0.02 | 0.13 | <.001 |
| Crop rotation (CR) | | |  | <.001 | 0.18 | 0.38 | <.001 | <.001 | 0.35 | 0.38 | <.001 | 0.03 | <.001 | 0.78 | <.001 | <.001 | 0.23 | <.001 | 0.65 | 0.2 |
| Cover crop (CC) | | |  | 0.73 | 0.22 | 0.54 | 0.67 | 0.27 | 0.34 | 0.26 | 0.83 | 0.16 | 0.72 | 0.16 | 0.38 | 0.71 | 0.56 | 0.26 | 0.57 | 0.26 |
| Soil depth | | |  | <.001 | <.001 | <.001 | 0.42 | <.001 | <.001 | <.001 | 0.06 | <.001 | 0.39 | 0 | 0.01 | 0.12 | 0.24 | 0.11 | 0.53 | 0.14 |
| Gypsum x CR | | |  | 0.97 | 0.82 | 0.28 | 0.56 | 0.68 | 0.53 | 0.16 | 0.88 | 0.56 | 0.58 | 0.62 | 0.93 | 0.7 | 0.25 | 0.72 | 0.85 | 0.15 |
| Gypsum x CC | | |  | 0.66 | 0.13 | 0.84 | 0.89 | 0.97 | 0.96 | 0.9 | 0.35 | 0.02 | 0.87 | 0.86 | 0.86 | 0.59 | 0.87 | 0.99 | 0.96 | 0.92 |
| Gypsum x depth | | |  | 0.73 | 0.82 | 0.55 | 0.57 | 0.59 | 0.73 | 0.39 | 0.33 | 0.87 | 0.56 | 0.88 | 0.25 | 0.26 | 0.89 | 0.68 | 0.76 | 0.69 |
| CR x CC | | |  | 0.99 | 0.81 | 0.93 | 0.85 | 0.8 | 0.18 | 0.71 | 0.97 | 0.7 | 0.84 | 0.81 | 0.41 | 0.68 | 0.96 | 0.73 | 0.43 | 0.78 |
| CR x depth | | |  | 0.35 | 0.8 | 1 | 0.34 | 0.23 | 0.94 | 0.86 | 0.13 | 0.75 | 0.32 | 0.92 | 0.14 | 0.06 | 0.98 | 0.1 | 0.98 | 0.76 |
| CC x depth | | |  | 0.51 | 0.13 | 0.72 | 0.42 | 0.94 | 0.19 | 0.8 | 0.3 | 0.05 | 0.4 | 0.14 | 0.8 | 0.35 | 0.74 | 0.88 | 0.35 | 0.96 |
| Gypsum x CR x CC | | |  | 0.63 | 0.78 | 0.74 | 0.34 | 0.98 | 0.05 | 0.74 | 0.08 | 0.41 | 0.33 | 0.36 | 0.16 | 0.22 | 0.7 | 0.99 | 0.28 | 0.62 |
| Gypsum x CR x depth | | |  | 0.99 | 0.99 | 0.99 | 0.97 | 0.91 | 1 | 0.93 | 0.95 | 0.99 | 0.97 | 0.9 | 0.95 | 0.93 | 1 | 0.97 | 0.99 | 0.99 |
| Gypsum x CC x depth | | |  | 0.9 | 0.63 | 0.79 | 0.9 | 0.89 | 0.57 | 0.87 | 0.91 | 0.67 | 0.88 | 0.96 | 0.64 | 0.87 | 0.87 | 0.9 | 0.73 | 0.95 |
| CR x CC x depth | | |  | 1 | 1 | 0.99 | 0.97 | 1 | 0.94 | 0.98 | 0.86 | 1 | 0.97 | 0.93 | 0.78 | 0.93 | 0.99 | 1 | 0.95 | 0.97 |
