# Supplementary material for: Gypsum, crop rotation, and cover crop impacts on soil organic carbon and biological dynamics in rainfed transitional no-till corn-soybean systems
Source: PLoS One. 2022 Sep 27;17(9):e0275198. doi: 10.1371/journal.pone.0275198 (PMC9514652; doi:10.1371/journal.pone.0275198)
Supplement: Attachment — Submitted filename: Response to Reviewers.docx [file pone.0275198.s007.docx]

**Response to Reviewers' comments:**

**1.** Is the manuscript technically sound, and do the data support the conclusions?

The manuscript must describe a technically sound piece of scientific research with data that supports the conclusions. Experiments must have been conducted rigorously, with appropriate controls, replication, and sample sizes. The conclusions must be drawn appropriately based on the data presented. Reviewer #1: No__________________________________

**2.** Has the statistical analysis been performed appropriately and rigorously? Reviewer #1: No

**3.** Have the authors made all data underlying the findings in their manuscript fully available?

The PLOS Data policy requires authors to make all data underlying the findings described in their manuscript fully available without restriction, with rare exception (please refer to the Data Availability Statement in the manuscript PDF file). The data should be provided as part of the manuscript or its supporting information, or deposited to a public repository. For example, in addition to summary statistics, the data points behind means, medians and variance measures should be available. If there are restrictions on publicly sharing data—e.g. participant privacy or use of data from a third party—those must be specified. Reviewer #1: No

**4.** Is the manuscript presented in an intelligible fashion and written in standard English?

PLOS ONE does not copyedit accepted manuscripts, so the language in submitted articles must be clear, correct, and unambiguous. Any typographical or grammatical errors should be corrected at revision, so please note any specific errors here. Reviewer #1: No

**Response:**

A per reviewers’ comments/suggestions, the manuscript was thoroughly revised for better flow, understanding, interpretation, and conclusions presented in simplified English language. All the raw data and their in-depth statistical analysis using SAS and treatment interactions were presented with supporting information (S1 File and S1 Table – S5 Table). Anybody will be able see and use the data, if necessary (with permission). The manuscript was revised and edited by several authors and a professional editor (Bradford Sherman, The Ohio State University Communications).

**_________________________________**

**Response to Specific comments**

**Reviewer #1:** The manuscript “Gypsum, crop rotation and cover crop impacts on soil organic carbon and biological dynamics in rainfed transitional no-till corn-soybean systems” has been reviewed. The MS provides information on SOC and biological properties under different gypsum rate, crop rotation and cover crop. After going through, the MS is having many fundamental issues which are appended below:

(i) Line 47-49: “SOC is a mixture of diverse and progressively decomposing organic compounds with different complexities and thermodynamic stabilities that greatly affects soil and environmental functions [1-4]”. This sentence is akward. The ‘mixture’ is not best fit.

**Response:** The manuscript was thoroughly revised as per suggestion. The sentence in lines 47-49 was simplified to avoid confusion and for easier understanding.

(ii) Line 49: “It is a composite core indicator” needs modification. It does not sound good.

**Response:** Simplified as suggested “It is one of the core indicators……….”

(iii) Introduction section is large. It needs to concise and simplification (for instance line 129-135 is not needed in Introduction). Also, many sentences are very complex that reduces readability.

**Response:** Totally agreed. The introduction has been shortened. The long and complex sentences were simplified to improve flow and readability. As suggested, lines 125 through 135 have been removed.

(iv) I don’t get the point in MS of adding gypsum in the studied soil. In general, gypsum is added in sodic degraded soil for reclamation. Author must highlight the background for soil status and suggested management. It is also important to clarify whether studied region is having any sodicity problem or not. Only, for carbon sequestration gypsum is added, this statement is not correct. The crop preference and soil geochemical properties drive the management practices in a cropping ecology.

**Response:** Thank you for the comment. For our United Soybean Board-funded project (2012 to 2016), gypsum (flue gas desulfurized gypsum) was applied to improve marginal soil quality to support economic crop productivity, especially corn and soybeans. The soils we have selected to conduct our long-term (still on-going) experiment at all sites were marginal soils (low fertility, compacted, etc.) except Hoytville-Ohio. None of our selected sites have experienced any sodicity or salinity problems. Details of the cropping diversity, cover crops, and soils information at all sites were described in our earlier published papers (Ref. 21, 32, 33, 52). Again, gypsum was not intended to apply for sequestering carbon, but to improve marginal soil quality. A sequence of papers has been published, and more has been submitted/will be submitted for publication with information on soil fertility and chemistry including heavy metals, soil compaction and aggregate stability, greenhouse gas emissions, and inductive and deductive soil quality.

Gypsum application has gained increasing use by farmers around the world, especially in relationship to remediating/reclaiming sodic and degraded soils. While soil carbon sequestration is a topic that has gained much attention in helping alleviate climate change, the information in the manuscript focuses on how gypsum, an agricultural soil amendment, impacts soil carbon fractions and is much needed.

(iv) Line 177-178: Authors mentioned that ‘days under shade at room temperature, processed, and analyzed for chemical and physical analysis (Table 1). However, Table 1 is related to the ‘List of abbreviations and definitions’. The title of table of table needs modification as per text (or vice versa).

**Response:** Table 1 was removed to avoid confusion of widespread use of abbreviations and/or acronyms. As suggested, a large number of abbreviations were removed and explained accordingly for better flow and readability.

(v) Table 1. List of abbreviations and definitions: It is not a format of table (without any column and row): modify accordingly.

**Response:** Table 1 has been removed.

(vi) The major fundamental issue is the MS devoid of soil physico-chemical and biological properties of studied soil under ‘Site descriptions’ in MM section. The initial soil properties of different sites are vital for a better idea on studied soil.

**Response**: Data on selected initial (2012) soil biological, chemical, and physical properties at 0-15 and 15-30 cm depths were briefly described under site description. The information was also listed in our earlier published papers (Ref. 21).

(vii) I understand authors must have the yield data of crops. The yield data could be correlated with CPI, NPI for a better clarity on impact of soil properties on yield sustainability.

**Response:** Thank you for the comment. Information associated with gypsum, crop rotation, and cover crop impact on crop yields was published in our earlier papers (Ref. 32 and 33). The relationship of CPI and NPI with crop yield will be presented in our upcoming “Inductive and deductive soil quality” papers (manuscript under preparation).

(vii) Excessive use of abbreviations in MS reduces the readability and clarity in MS.

**Response**: As suggested, the use of abbreviations has been minimized.

(viii) In conclusion, it is mentioned that “Gypsum, crop rotation, and cover crop variably affected the depth distribution of SOC pools, TN, 607 and SOC lability without any consistent interactions within- and across the sites”. However, no table is having any mention on depth on which data presented. Only, in MM section it is mentioned 0-15 and 15-30 cm soil sampling depth (line 173). Then, where is depth wise data and how this inference was drawn.

**Response:** We did point out that under no-till there will be a stratification effect on soil properties in response to lack of plowing, which was an expected outcome. That is why we did not present the soil depth information in the main tables. However, detailed information on statistical analysis associated with all the main treatments and their interactions, including soil depth, was presented in support information (S1 File and S1 Table – S5 Table).
