# Supplementary material for: Gypsum, crop rotation, and cover crop impacts on soil organic carbon and biological dynamics in rainfed transitional no-till corn-soybean systems
Source: PLoS One. 2022 Sep 27;17(9):e0275198. doi: 10.1371/journal.pone.0275198 (PMC9514652; doi:10.1371/journal.pone.0275198)
Supplement: Attachment — Submitted filename: Response to Review Comments-3.docx [file pone.0275198.s008.docx]

**Response to Review Comments**

**Reviewer # 1:** (No Response)

**Reviewer # 2:** This article is a well written manuscript based on an extensive study including field experiments at four different locations. The statistical analyses performed in the study also appears sufficient. However, I could find some issues which the authors should address before considering the article for publication.

**Response:** The author(s) acknowledged reviewer’s proactive comments and/or contribution to improve the quality of the manuscript. Same credits for the academic editor.

______________________________________________________________________________

**Comment:** L 31: Write 0-15 and 15-30 cm instead of 0-15 vs. 15-30 cm

**Response**: Thanks. Corrected accordingly.

**Comment:** L 46: Nitrogen pool index is mentioned in the keywords, but there is no mention of it in the abstract. At least one sentence in the abstract should mention NPI.

**Response**: The NPI information was added in the abstract.

**Comment:** The authors have responded to a query of an earlier reviewer that ‘gypsum was applied to improve marginal soil quality to support economic crop productivity.’ None of the soils in this study was sodic. Then gypsum would only have supplied Ca and SO_4_^2-^. First of all, if these two were not deficient in the soil (whether sufficient or deficient in the soil was not mentioned in the article), what was the original intent to apply the gypsum. Also, I could not understand how gypsum could improve different labile C pools in soil by only supplying Ca and SO_4_^2-^ if they were not deficient to limit microbial activity. Observing the pH, I don’t think Ca would be deficient in these soils. S may be deficient I don’t know as there is no data on that. If gypsum is not amending any inherent problem like sodicity (which is not the case here as no soil was sodic) or supplying any nutrient which is deficient, then how it has a significant effect on improving soil quality and support economic crop productivity?

**Response:** While none of the soils were sodic, they were less productive soils, especially Marvyn loamy sand (Shorter, Alabama) and Omulga silt loam (Piketon, Ohio) soils were marginal or degraded soils. While the Marvyn ***loamy sand*** is a low water holding capacity, poor aggregate stability, and low SOM content marginal sandy soil, the ***Omulga silt loam*** is a compacted fragipan and low SOM content degraded soil.

While most of our studied soil nutrient content data associated with FGD gypsum have already been published (*Gonzalez JM, Dick WA, Islam KR, Watts DB, Fausey NR, Flanagan DC, VanToai TT, Batte MT, Reeder RC, Kost D, Shedekar VS. (2022) Gypsum and cereal rye cover crops affect soil chemistry: Trace metals and plant nutrients. Soil Science Society of America Journal.* [*https://wileyonlinelibrary.com/journal/saj2*](https://wileyonlinelibrary.com/journal/saj2)), the exchangeable Ca and S concentration of all the soils were included in the manuscript, as per suggested.

FGD gypsum not only provides Ca and S, but it also provides Mg, K, and micronutrients. The cationic bridging effects of Ca promote flocculation of clay and SOC to form soil macroaggregate stability (≥250 µm size aggregates) that lead to accumulated, macroaggregate protected organic C (POC) as particulate organic matter (POM), which is one of the labile C pools. Moreover, gypsum decreases surface crusts formation, acts as an electrolyte to facilitate water infiltration due to its higher solubility than lime, and reduce soluble reactive phosphorus via runoff and tile drainage. All these small but proactive effects of FGD gypsum are expected to improve microbial activity, influence SOC lability and accumulation, and affect crop productivity in conjunction with no-till and cropping diversity with cover crops.

**Comment**: L 88-90: Mention the type of soil where such results were found

**Response:** As suggested, a brief description on soil type was added. “Recently, a few studies have reported that gypsum application favors soil biology and enzymatic activity in *Hagerstown silt loam* (pH 6.2) in Pennsylvania, USA [16] and in alkaline-saline clay soils in northwest China [24]. Gypsum amendments provide several nutrients essential for crop growth in *Marvyn loamy sand* (marginal soil with poor aggregate stability and low SOM content) in Alabama (Shorter), *Blount silt loam* in Indiana (Farmland), *Hoytville clay* in northwestern Ohio, and *Omulga silt loam* (degraded soil with compaction and low SOM content) in southern Ohio, respectively [21]”.

**Comment**: L 99: Rewrite the sentence to avoid repetition of similar words

**Response:** Thanks. Revised accordingly.

**Comment**: L 189: Write ‘physical properties’ instead of ‘physical analysis’

**Response**: Thanks. Corrected, as per suggestion.

**Comment**: L 196: Put the starting parenthesis ‘(‘before ‘SMBC:SOC’)

**Response:** Edited accordingly.

**Comment:** L 217-218: Authors have mentioned that “The labile C pool was considered as a portion of the SOC that was comprised of the combined pools of active C, SMBC, and cold and hot-water C.” However, active C, SMBC, and cold- and hot- water C were not extracted sequentially from the same sample; they were extracted from separate samples with different methods. Each of them may a part of the labile C; but they are overlapping themselves. As they are not mutually exclusive, adding them up clearly overestimates the CL, and at the same time underestimates the non-labile C as the later was derived by subtracting CL from total SOC. Hence, the parameters derived from CL are also questionable. The authors should find a way to rectify this issue or give an appropriate rebuttal.

**Response:** Current statement about the “labile C pool” written in lines 217 - 218 has been corrected. The conceptually defined labile C pools such as active C, SMBC, and cold- and hot- water C were determined by KMnO_4_ oxidation, extraction with distilled deionized water, or K_2_SO_4_ solution using different methods. Separate soil samples were used to measure each C pool. It is not a combined C pool, each C pool was determined separately from SOC. That’s why we have different CL, CLI, and CMI values associated with active C, SMBC, and cold- and hot- water C pools, respectively (Table 1 to 5).

**Comment:** Table 1, last row: This value of SMBC (1228 mg kg^-1^) seems too high, might be a typo, please check; If not a typo, then how come so huge difference is not significant?

**Response:** Thanks. It’s a typo. Corrected accordingly.

**Comment:** L 280-281: 2.2 kg or Mg ha^-1^?

**Response:** Corrected accordingly (2.2 Mg/ha)

**Comment:** L 317-318: The authors have seen some benefits of 2.2 Mg/ha gypsum application on SMBC, SMBC:SOC, and CMI; but, without the mention of its effect on crop yield, the information remains incomplete. They should at least include the average yield of four years under different treatments.

**Response**: Thank you for the comment. Detailed information associated with gypsum, crop rotation, and cover crop impact on crop yields was published in our earlier papers (Cited ref # 32 and 33 in the manuscript reference list). As the crop yield data were already published, we have tried to minimize the data overlapping and repetitions in incoming and future papers except soil quality indexing and evaluation.

*Cited ref. # 32:* Batte MT, Dick WA, Fausey NR, Flanagan DC, Gonzalez JM, Islam R, Reeder R, VanToai T, Watts DB. (2018) Cover crops and gypsum applications: Soybean and corn yield and profitability impacts. American Society of Farm Manager and Rural Appraisers 8:47-71.

*Cited ref. # 33:* Raut Y, Shedekar V, Islam K, Gonzalez J, Watts D, Dick W, Flanagan D, Fausey N, Batte M, Reeder R, VanToai T. (2020) Soybean yield response to gypsum soil amendment, cover crop and rotation. Agricultural and Environmental Letters. https://doi.org/10.1002/ael2.20020.
